# Direct esterification of amides by the dimethylsulfate-mediated activation of amide C–N bonds

**DOI:** 10.1038/s42004-024-01180-9

**Published:** 2024-04-27

**Authors:** Hongjian Qin, Zijian Han, Emmanuel Mintah Bonku, Haiguo Sun, Abdullajon Odilov, Fuqiang Zhu, Safomuddin Abduahadi, Weiliang Zhu, Jingshan Shen, Haji A. Aisa

**Affiliations:** 1 Key Laboratory of Plant Resources and Chemistry in Arid Regions, Xinjiang Technical Institute of Physics and Chemistry, Chinese Academy of Sciences, Urumqi, Xinjiang PR China; 2 University of Chinese Academy of Sciences, Beijing, PR China; 3 CAS Key Laboratory of Receptor Research, State Key Laboratory of Drug Research, Drug Discovery and Design Center, Shanghai Institute of Materia Medica, Chinese Academy of Sciences, Shanghai, PR China; 4 State Key Laboratory of Drug Research, Shanghai Institute of Materia Medica, Chinese Academy of Sciences, Shanghai, PR China; 5 Topharman Shanghai Co., Ltd., Shanghai, PR China

**Keywords:** Synthetic chemistry methodology, Synthetic chemistry methodology, Homogeneous catalysis, Organocatalysis

## Abstract

Amides are important intermediates in organic chemistry and the pharmaceutical industry, but their low reactivity requires catalysts and/or severe reaction conditions for esterification. Here, a novel approach was devised to convert amides into esters without the use of transition metals. The method effectively overcomes the inherent low reactivity of amides by employing dimethylsulfate-mediated reaction to activate the C-N bonds. To confirm the proposed reaction mechanism, control experiments and density functional theory (DFT) calculations were conducted. The method demonstrates a wide array of substrates, including amides with typical H/alkyl/aryl substitutions, *N,N*-disubstituted amides, amides derived from alkyl, aryl, or vinyl carboxylic acids, and even amino acid substrates with stereocentres. Furthermore, we have shown the effectiveness of dimethylsulfate in removing acyl protective groups in amino derivatives. This study presents a method that offers efficiency and cost-effectiveness in broadening the esterification capabilities of amides, thereby facilitating their increased utilization as synthetic compounds in diverse transformations.

## Abstract (web summary)

Amides are important intermediates in organic chemistry and the pharmaceutical industry, but their low reactivity requires catalysts and/or harsh reaction conditions to esterify them. Here, the authors report a versatile and mild dimethyl sulfate-mediated reaction to activate and esterify C—N bonds, achieving up to 95% amide bond cleavage.

## Introduction

**Fig. 1:**
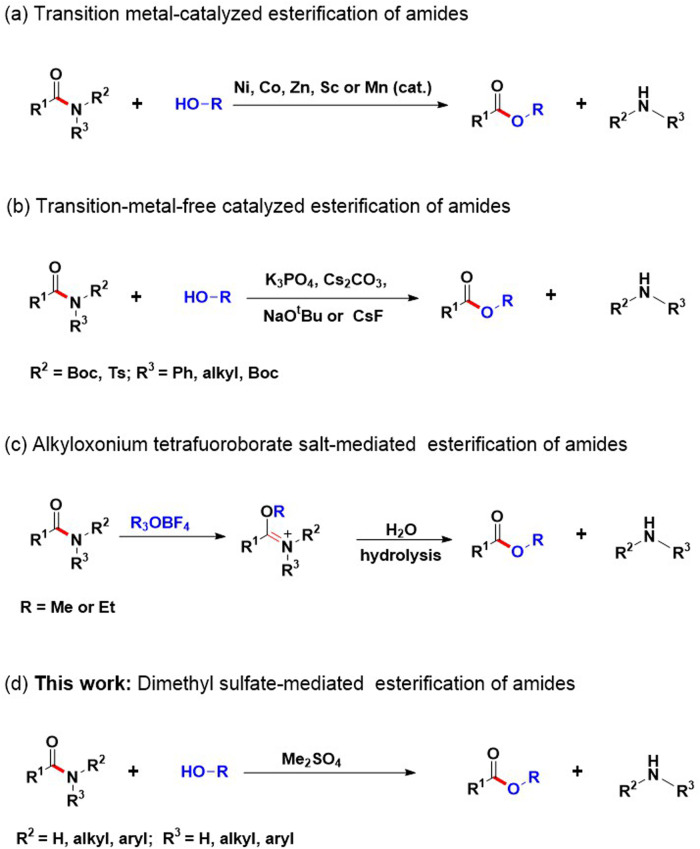
Methods for esterification reactions of amides. **a** Transition metal-catalyzed esterification of amides. **b** Transition-metal-free catalyzed esterification of amides. **c** Alkyloxonium tetrafuoroborate salt-mediated esterification of amides. **d** This method for dimethyl sulfate-mediated esterification of amides.

Amides play significant role as functional groups and important structural patterns in organic molecules.^1–4^ They serve as the fundamental units of proteins and can be found in various natural and synthetic compounds.^5,6^ They are generally considered to be weak electrophiles, which is mainly attributed to the resonance stability of the amide bond^7^. The stability and reactivity of the amide bond are associated with the planar resonance of the bond (15–20 kcal mol^-1^)^8–13^. However, any distortion of the amide bond, disrupting the planar conjugation, leads to notable changes in the physical and chemical properties of the amide. Additionally, this implies that the conversion of amides to other functional groups typically necessitates demanding experimental conditions and extended reaction durations. Specifically, the direct formation of esters from amides necessitates the application of highly acidic or alkaline conditions, which are unsuitable for the delicate functional groups. To address this limitation, various methods have been devised for the esterification of amides. Transition metal-catalyzed reactions can be considered as one of the most effective methods for achieving this goal^14–19^, as shown in Fig. 1a. Amides have been esterified by Garg and colleagues utilizing a catalyst consisting of Ni (0) /NHC^20,21^. Additionally, Danoun and colleagues^22^ have expanded upon this method by applying a cobalt system. Metal-free conditions are commonly used for amide esterification, with several protocols^23–26^ involving amide alcoholysis in basic conditions or Boc activation/base elimination (as shown in Fig. 1b). The conversion of amides to esters using alkyloxonium tetrafuoroborate salts has been previously reported in several studies^27–32^, involving the formation of imidate esters followed by subsequent hydrolysis to obtain amines and esters (as shown in Fig. 1c).The limited scope of the substrate still remains despite these advances. Developing a novel protocol without transition metals that can be applied to amides with normal H/alkyl/aryl substitutions remains a challenging and attractive task.

To overcome the longstanding issue related to the limited reactivity of amides and their limited application in C-N bond cleavage processes, we present the initial direct esterification of amides mediated by dimethyl sulfate (Fig. 1d). Notable aspects of our research comprise (a) the demonstration of a novel conceptual pathway for amide esterification utilizing dimethyl sulfate-mediated amide esterification; (b) the establishment of a single versatile dimethyl sulfate-mediated system capable of esterifying various amides; (c) the pioneering use of the easily accessible, stable, and cost-effective dimethyl sulfate, eliminating the need for expensive transition metal catalysts and ligands. Overall, this work demonstrates that dimethyl sulfate can significantly improve the esterification range of amides, which may contribute to the increasing use of amides as synthetics in various transformations. However, dimethyl sulfate is a known hazardous chemical^33^, highly toxic, seemingly harmless chemical commonly used in industry as a methylating agent. The substance can be readily absorbed through the skin or respiratory tract, potentially leading to poisoning or fatal outcomes during use, handling, or transportation^33,34^. To reduce potential hazards, the process should be confined to a controlled environment with adequate local exhaust ventilation. Operators should receive specialized training, adhere strictly to established procedures, and wear closed-circuit filter respirators (half-mask style), chemical safety goggles, enclosed gas suits, and rubber gloves to prevent exposure. It’s important to note that, empty containers or post-treatment reaction solutions may contain residual hazardous substances that necessitate proper disposal, which can be achieved by degrading residual hazardous materials with solutions of sodium hydroxide (1 mol/L), sodium carbonate (1 mol/L), or ammonia (1.5 mol/L)^33,35^.

## Results and discussion

### Optimization of reaction conditions

Amide **1a** was chosen as a model substrate, dimethyl sulfate and methanol were mixed in a solvent, and the product methyl benzoate (**2a**) was observed to investigate our proposed transformation. We screened several solvents for coordination with amide **1a** using dimethyl sulfate as a catalyst and found that only a trace of **2a** was observed in acetonitrile (MeCN), tetrahydrofuran (THF), toluene, dioxane, and chlorobenzene (Table 1, entries 1–5). To our delight, after surveying various solvents, methanol was determined to be the most suitable reaction medium (Table 1, entries 6–7). It was evident that the esterification progressed at a sluggish pace when conducted at a temperature of 25 °C (Table 1, entry 6).

**Table 1:** Optimization of the reaction conditions^a^


Entry	Solvent	Temp. (°C)	Yield (%)^b^
1	THF	66	trace
2	MeCN	82	trace
3	Toluene	111	15
4	Dioxane	101	13
5	Chlorobenzene	132	20
6	Methanol	25	40
7	Methanol	65	95

^a^ Reaction was carried out with amide **1a** (1.0 mmol, 1.0 equiv), dimethyl sulfate (1.0 mmol, 1.0 equiv), methanol (2.0 eq), solvent (0.2 M), and heated for 12 h. ^b^ Calibrated GC yield.

### Reaction scope of alcohols for amide esterification

**Fig. 2:**
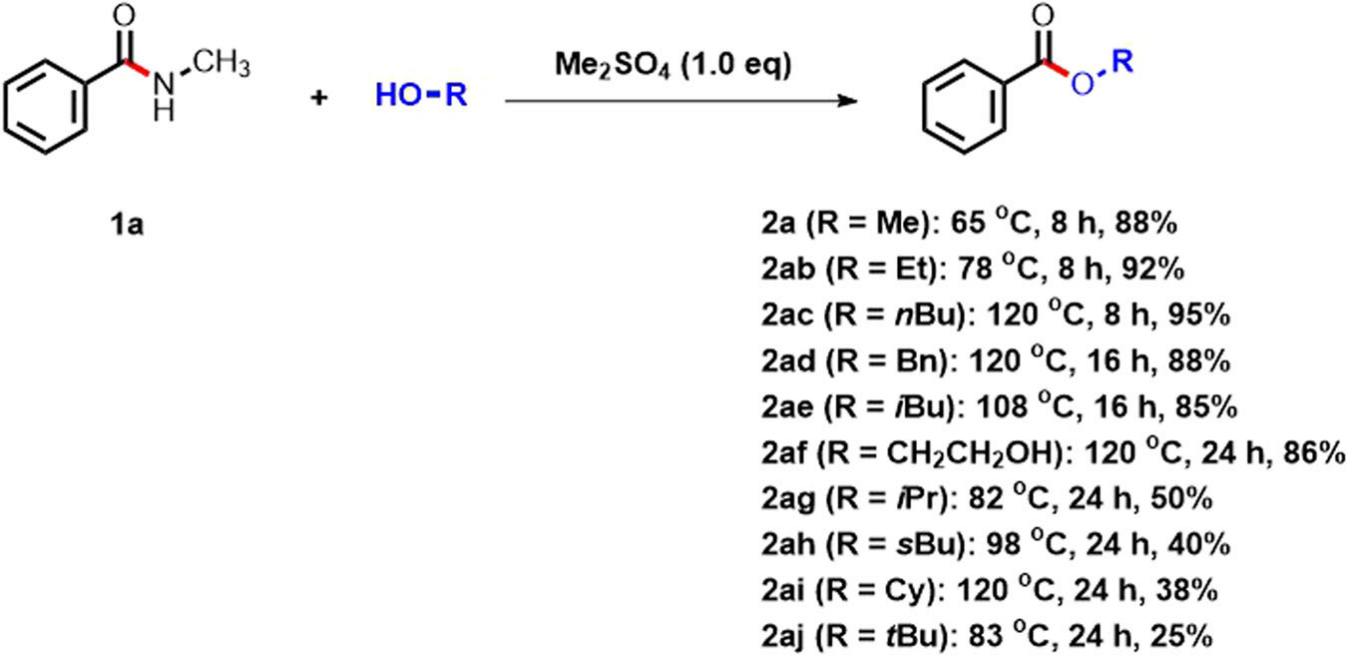
Scope of alcohols for amide esterification. Reaction conditions: amide **1a** (1.0 mmol, 1.0 equiv), dimethyl sulfate (1.0 mmol, 1.0 equiv), alcohols as solvent (0.2 M), and heated for 8 − 12 h in a sealed vial under an atmosphere of N_2_. Isolated yields are provided.

After optimizing the reaction conditions, we proceeded to assess the esterification of amides using the dimethyl sulfate mediated method. The scope of alcohols was investigated (Fig. 2). Under similar reaction conditions, esters **2a,** **2ab,** **2ac,** **2ad,** **2ae**, and **2af** were obtained in high yields (85–95%) from primary alcohols including methanol, ethanol, 1-butanol (n-BuOH), benzyl alcohol, isobutanol, and ethylene glycol. Nevertheless, when secondary alcohols (such as i-PrOH, s-BuOH or CyOH) and tertiary alcohols (like t-BuOH) were employed in accordance with our standard reaction procedure, the resulting esters **2ag,** **2ah,** **2ai**, and **2aj** exhibited diminished yields ranging from 25% to 50%.

### Reaction scope of amides for esterification

In order to further investigate the extent and constraints of this direct esterification method, a diverse range of amides were employed to produce the intended outcomes using the standard reaction conditions. This protocol was found to be compatible with a diverse array of amides, as indicated in Table 2. Significantly, primary and secondary amides derived from substituted phenyl carboxylic acids could be esterified smoothly in comparable yields (55 − 93%). Furthermore, apart from the primary benzamide (**1ab** and **1ac**), other compounds with the electron-withdrawing the nitroxyl, chloride or fluoride substituents (**1c,** **1cb,** **1d,** **1db,** **1j** and **1jb**) or the electron-donating methoxy or methyl substituents (**1b,** **1bb,** **1** **h** and **1hb**) exhibited good compatibility. In addition to phenyl derivatives, we explored substrates of naphthyl and heterocyclic nature. Esterification of naphthyl compounds (**1i** and **1ib**) and pyridine substrates (**1** **g** and **1gb**) resulted in high yields ranging from 83% to 93%. Additionally, amides derived from alkyl carboxylic acid substrates (**1e,** **1eb,** **1** **f** and **1fb**) were also amenable to the reaction. Motivated by the investigation of the esterification process of amides derived from alkyl carboxylic acids, our exploration commenced on 8-aminoquinoline amides. These amides serve as a remarkably efficient and extensively employed directing group amide, functioning as a bidentate auxiliary in diverse metal-catalyzed C − H functionalization reactions^36–43^. 8-Aminoquinoline amide derived from 3-phenylpropanoic acid (**1k**) reacted to give the corresponding ester (**2k**) in 92% yield. Among them, the chiral compounds, amides derived from amino acids (**1** **m,** **1n** and **1nn**) were esterified to the corresponding ester products (**2** **m,** **2** **mb,** **2n,** **2nb,** **2nn** and **2nnb**) in 80 − 93% yields. Additionally, it is worth mentioning that there was no racemization of ester products (Supplementary Figs. S1−S12) observed, which emphasizes the gentle reaction conditions that effectively hinder significant epimerization of the α stereocenters. We also found that the methodology tolerates α,β-unsaturated 8-aminoquinoline amide (**1** **l**), *N*-acyl-succinicimide (**1of**), *N*-acyl glutarimide (**1og**), as well as some substrates (**1pp,** **1qq,** **1rr**) containing nucleophilic sites, such as amino or hydroxyl groups. This resulted in the formation of **2** **l,** **2of,** **2og,** **2pp,** **2qq** and **2rr**, respectively, in good yields (85 − 93%). However, tertiary amide substrates (**1ad,** **1ae,** **1af,** **1ag,** **1bc,** **1cc,** **1dc,** **1ec,** **1** **fc,** **1gc,** **1hc,** **1ic** and **1jc**) undergo the dimethyl sulphate-mediated esterification under our reaction conditions failed to give ester products in desirable yields

**Table 2:**
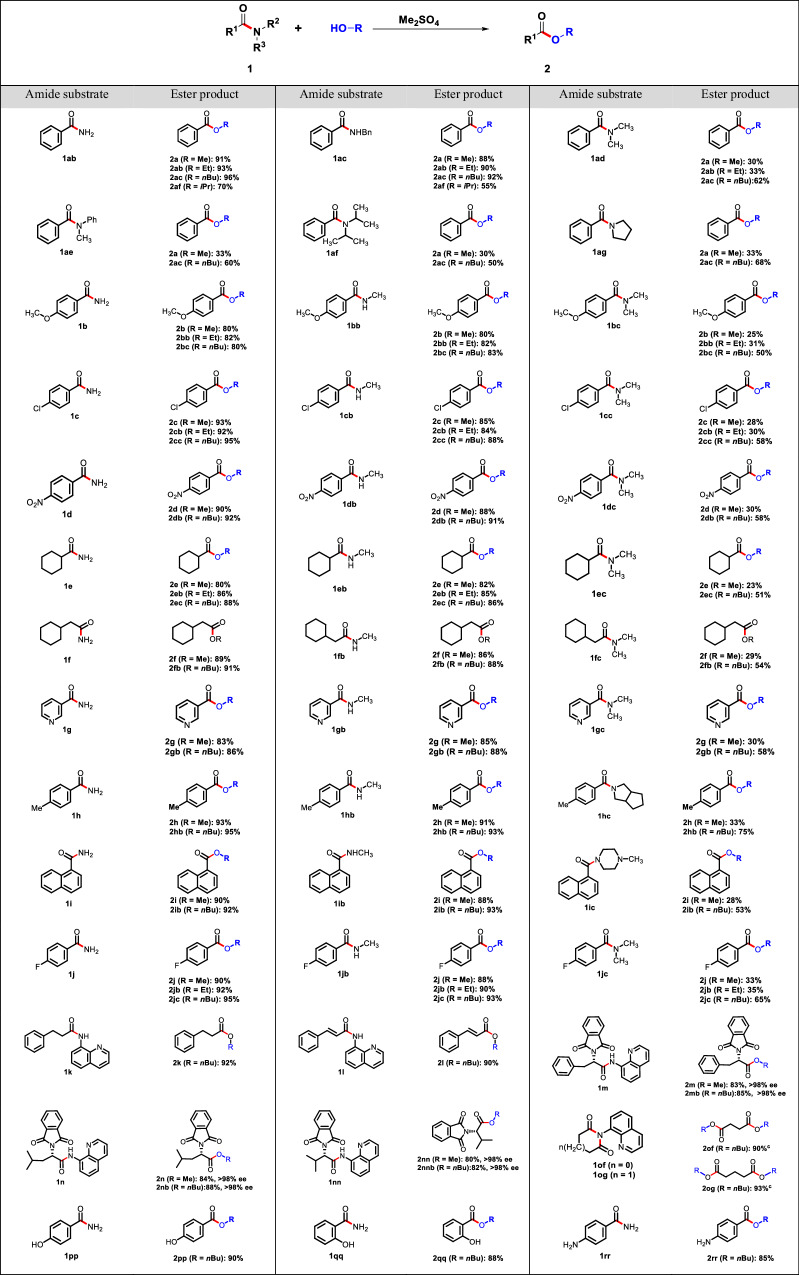
Scope of amides for esterification^a,b^

^a^ Reaction conditions: amides (1 mmol, 1.0 equiv), dimethyl sulfate (1.0 mmol, 1.0 equiv), alcohols as solvent (0.2 M), and heated at 100 °C for 8 − 12 h in a sealed vial under an atmosphere of N_2_. ^b^ Isolated yields. ^c^ Amides (1 mmol, 1.0 equiv), dimethyl sulfate (2.0 mmol, 2.0 equiv) alcohols as solvent (0.2 M), and heated at 120 °C for 24 h in a sealed vial under an atmosphere of N_2_.

### Cleavage of acyl protective group on amines with dimethyl sulphate

We next applied this methodology to the cleavage of acyl protective group on amines with dimethyl sulphate. In order to explore the scope and limitations of our method, we investigated a series of amino derivatives containing several acyl protective groups, for example acetyl group, benzoyl group, pivaloyl group, succinic acyl group, Boc and Cbz (Table 3). We began to investigate primary aromatic amino derivatives substrates, such as 8-aminoquinoline amides (**1o,** **1ob,** **1oc,** **1od,** **1oe,** **1of** and **1og**), 1-naphthylamine amide (**1p,** **1pb,** **1** **pc,** **1pd** and **1pe**). We found that acetyl, benzoyl, pivaloyl, Boc and Cbz protective groups were easily removed under our cleavage conditions, leading to the corresponding amines (**3o** and **3p**) in 85−95% yields; while the *N*-acyl-succinicimide **1of** or *N*-acyl-glutarimide **1og** counterparts required doubled-loading of dimethyl sulphate mediate and extended reaction time (24 hours) to achieve satisfactory reaction out comes in 85 − 88% yields. In addition, treatment of primary aliphatic amino derivatives (**1q,** **1qb,** **1qc,** **1qd** and **1qe**) using the current conditions provided the desired product **3q** in 84 − 95% yields. Gratifyingly, the protocol was compatible with secondary amino derivatives (**1r−1re,** **1** **s−1se,** **1t−1td,** **1** **u−1ue** and **1** **v−1ve**) containing acyl protective groups, and all of these reactions proceeded successfully, resulting in the formation of products **3r** − **3** **v** with yields ranging from 60% to 88%. Afterwards, we examined the reaction’s tolerance by screening the chiral amino acids derivatives with either the Boc protective group (**1w,** **1x**, and **1** **y**) or the Cbz protective group (**1wb,** **1xb**, and **1yb**).To our delight, the transformation also proceeded smoothly, in that the corresponding products (**3w,** **3x** and **3** **y**) were furnished in 82 − 90% yields with >98% ee (Supplementary Figs. S13−S18).

**Table 3:**
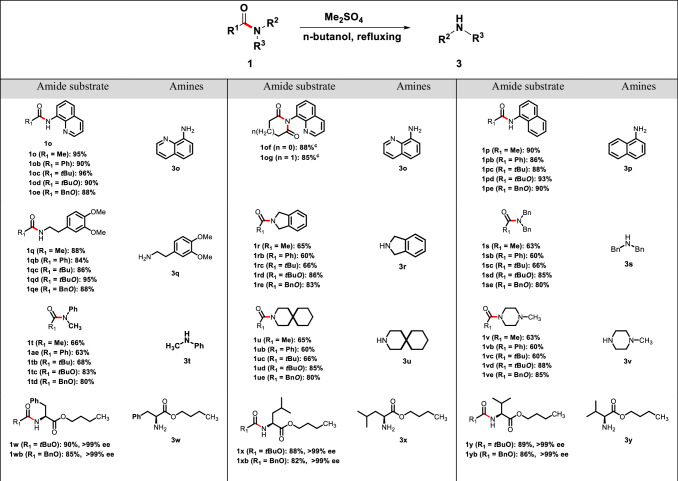
Cleavage of acyl protective group on amines with dimethyl sulphate^a,b^

^a^ Reaction conditions: amides (1 mmol, 1.0 equiv), dimethyl sulfate (1.0 mmol, 1.0 equiv), n-butanol as solvent (0.2 M), and heated at 120 °C for 8 h in a sealed vial under an atmosphere of N_2_. ^b^ Isolated yields. ^c^Dimethyl sulfate (2.0 mmol, 2.0 equiv) and heated at 120 °C for 24 h.

### Mechanistic studies

**Fig. 3:**
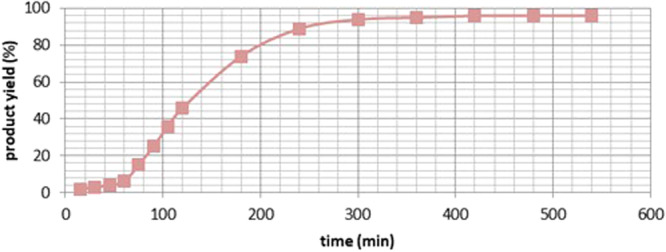
Yield-time curves of the esterification of 1a. Representative time course of the esterification of 1a, monitored by GC analysis against an internal standard.

**Fig. 4:**
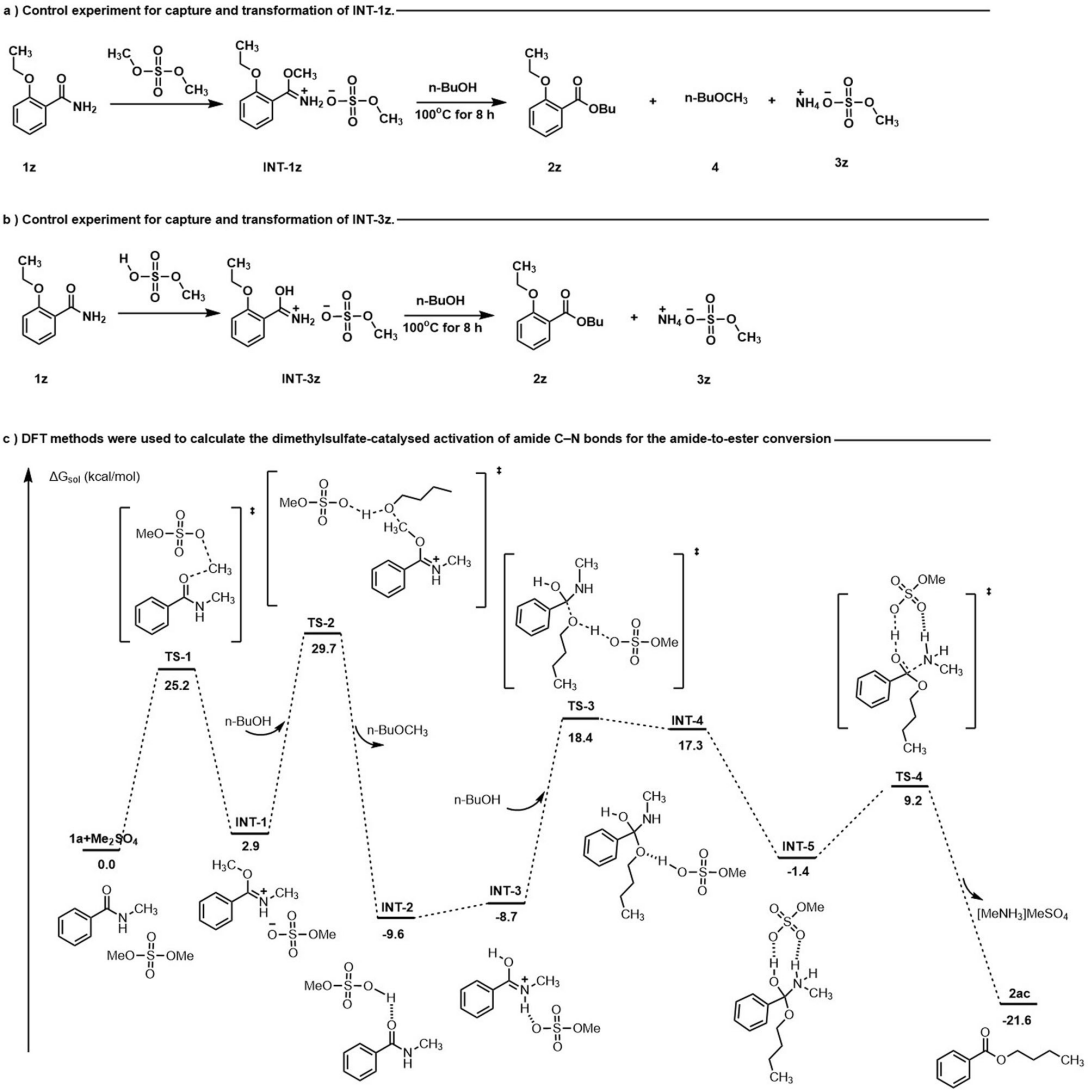
Control experiments and possible mechanism. **a** Control experiment for capture and transformation of **INT-1z**. **b** Control experiment for capture and transformation of **INT-3z**. **c** DFT methods were used to calculate the dimethylsulfate-mediated activation of amide C–N bonds for the amide-to-ester conversion.The calculation is performed at the M06-2X SMD/6-311 + + G(d,p)//M06-2X PCM/6-31 G(d) level of theory. The relative Gibbs free energies (ΔG_sol_) are represented in kcal/mol.

In order to gain insight into the esterification mechanism of amides mediated by dimethylsulfate, representative time course of the esterification of **1a** and a series of control experiments were conducted. The kinetic behavior of the esterification reaction with dimethyl sulfate-mediattion was studied using GC. As depicted in Table 4 (Fig. 3), the esterification reaction was observed to occur in three stages: an induction period (Fig. 3, 0 − 60 min), an active period characterized by rapid formation of product **2ac** (60 − 240 min), and a final period with significantly reduced reaction rate ( > 240 min). These experiments aimed to capture and isolate any potential reaction intermediates, as depicted in Fig. 4a. Specifically, the reaction between amide **1z** and dimethylsulfate was conducted at a temperature of 75 °C for an extended duration in the absence of alcohols (Fig. 4a). It is worth mentioning that a yield of 95% was achieved for **INT-1z** (Supplementary Data 4 and Supplementary Table S2−S8), with no detection of **2z**, which is believed to be a potential intermediate for the esterification process. Fortunately, **INT-1z** could be readily converted to ester **2z** with a high yield of 93% by heating it at 100 °C in n-BuOH for a few hours (Fig. 4a). A similar outcome was observed when amide **1z** was reacted with methyl hydrogen sulfate in MeOH at a temperature of 50 °C for a duration of 30 min, or under ambient conditions for a duration of 24 hours. It is worth noting that the compound **INT-3z**, which is O-protonated amide, was successfully isolated (Fig. 4b). This compound serves as a possible intermediate for the esterification process. Upon heating compound **INT-3z** at 100 °C in n-BuOH for several hours, ester **2z** was obtained with a high yield of 88% (Fig. 4b). The isolation and conversion of **INT-1z** and **INT-3z** demonstrate that dimethylsulfate plays a significant role in the formation of these intermediates, which are essential for the esterification reaction.

**Table 4:** Representative time course of the esterification of 1a ^a^


Time (minutes)	Yield (%)^b^	Time (minutes)	Yield (%)^b^
15	2	180	74
30	3	240	89
45	4	300	94
60	6	360	95
75	15	420	96
90	25	480	96
105	36	540	96
120	46		

^a^ Reaction was carried out with amide **1a** (1.0 mmol, 1.0 equiv), dimethyl sulfate (1.0 mmol, 1.0 equiv), n-butanol as solvent (0.2 M), and heated at 120 ^o^C, monitored by GC analysis against an internal standard. ^b^ Calibrated GC yield.

To gain a deeper understanding of this novel amide esterification pathway, density functional theory (DFT) calculations were conducted at M06-2X SMD/6-311 + + G(d,p)//M06-2X PCM/6-31 G(d) level. Drawing upon both experimental and theoretical evidence, the mechanism of the reaction was proposed (Fig. 4c and Supplementary Table S1). **1a** and dimethyl sulfate was selected as the zero-point of the potential surface (**1a** + **Me**_**2**_**SO**_**4**_, 0.0 kcal/mol). Firstly, the methylation of carbonyl oxygen by dimethyl sulfate occurs through transition state **TS1** (25.2 kcal/mol) to form imidoesterification intermediate **INT-1**. Then the oxygen atom of n-butanol attacks the carbon atom of **INT-1** through **TS-2** (26.8 kcal/mol), yielding **INT-2**. **INT-1** has been observed in the experiment, probably because of the stability of this amide salt and a relatively high energy barrier. Next, **INT-2** transfers into **INT-3**, where the hydrogen bond species is converted into an O-protonated amide species. The formation of **INT-3** has been proved by control experiment and it sets the stage for the next step. **INT-3** undergoes a transformation to yield **INT-4** via **TS-3** (27.1 kcal/mol), where a second molecule of n-butanol attacks the carbocation. Afterwards, a proton transfer happens, giving rise to two new hydrogen bonds within **INT-5**. Ultimately, **INT-5** undergoes an amino group departure process **TS-4** (10.6 kcal/mol) to form the desired product **2ac**.

In summary, a novel and efficient transition-metal-free method has been devised for the direct esterification of amides through the activation of C-N bonds, employing dimethyl sulfate-mediated reaction. This approach successfully addresses the inherent reactivity limitations of amides as functional groups and offers a diverse array of substrates amenable to different transformations. Control experiments and Density Functional Theory (DFT) calculations have contributed to a more comprehension of the proposed mechanism governing the amide esterification pathway. This mechanism entails an initial imidoesterification step, succeeded by n-butanol attacks resulting in the formation of an O-protonated amide and the subsequent departure of an amino group. The protocol presented in this study exhibits remarkable versatility and operates under mild conditions. Furthermore, its metal-free composition supplements the conventional transition-metal-catalyzed esterification of amides. Consequently, this protocol represents a promising and viable new pathway for achieving catalytic cleavage of amide C-N bonds, yielding up to 95%. The findings of this research significantly broaden the scope of esterification of amides, thereby facilitating their increased utilization as synthetic intermediates in the fields of organic chemistry and the pharmaceutical industry.

## Methods

### General procedure for amide esterification (Supplementary Note 1)

To a solution of amides **1** (1.0 equiv) in alcohols (0.2 M) was added dimethyl sulfate (1.0 equiv), and heated for 8 − 24 h at 65 − 120 °C in a sealed vial under an atmosphere of N_2_ (monitored by TLC). The resulting mixture concentrated in vacuo to give residues. Then the residues were dissolved in ethyl acetate and washed with brine, dried over Na_2_SO_4_, filtered, and concentrated in vacuo. The crude product was purified by silica-gel column chromatography to give ester products **2**.

### General procedure for cleavage of acyl protective group on amines with dimethyl sulphate

To a solution of amides **1** (1.0 equiv) in n-butanol (0.2 M) was added dimethyl sulfate (1.0 equiv), and heated for 8 h at 120 °C in a sealed vial under an atmosphere of N_2_ (monitored by TLC). The resulting mixture concentrated in vacuo to give residues. Then the residues were dissolved in ethyl acetate and washed with sodium bicarbonate saturated solution, brine, dried over Na_2_SO_4_, filtered, and concentrated in vacuo. The crude product was purified by silica-gel column chromatography to give amine products **3**.

### Supplementary information

Supplementary Information file

Description of Additional Supplementary Files

Supplementary Data 1 file

Supplementary Data 2 file

Supplementary Data 3 file

Supplementary Data 4 file

## Data Availability

All data supporting the findings of this study are available within this article and its Supplementary Information file. The copies of ^1^H NMR and ^13^C NMR spectra of the compounds obtained in this manuscript are available in Supplementary Data 1. Source Data for Supplementary Tables S1−S8 are available within Supplementary Data 2. Source Data for Supplementary Figs. S1-S18 for copies of compound chromatograms obtained in this manuscript are provided in Supplementary Data 3. Cif (crystallographic data) for compound INT-1z as Supplementary Data 4. The X-ray crystallographic coordinates for structures reported in this study have been deposited at the Cambridge Crystallographic Data Centre (CCDC), under deposition number 2347148 (for **INT-1z**). These data can be obtained free of charge from The Cambridge Crystallographic Data Centre via www.ccdc.cam.ac.uk/data_request/cif. The data are also available from the corresponding author upon reasonable request.
