# Supplementary material for: Direct esterification of amides by the dimethylsulfate-mediated activation of amide C–N bonds
Source: Commun Chem. 2024 Apr 27;7:93. doi: 10.1038/s42004-024-01180-9 (PMC11055851; doi:10.1038/s42004-024-01180-9)
Supplement: Supplementary file 1 — Supplementary Information file [file 42004_2024_1180_MOESM1_ESM.pdf]

Supplementary Information

Direct esterification of amides by the dimethylsulfate-mediated activation of amide C–N bonds

Contents

General Information 1
Experimental section 2
Synthesis of (hexahydrocyclopenta[c]pyrrol-2(1H)-yl)(p-tolyl)-methanone 1hc 2
General procedure for preparation of 8-aminoquinoline amide substrates 2
General procedure for preparation of 3-Azaspiro[5.5]undecane amide substrates 5
General procedure for preparation of amino acids derivative with protective group substrates 7
General procedure for amide esterification 8
Synthesis of n-butyl 2-ethoxybenzoate 18
General procedure for cleavage of acyl protective group on amines with dimethyl sulphate 20
Computational details 23
DFT Calculations 23
Supplementary References: 30

Supplementary Note 1

General Information

All glassware was thoroughly oven-dried. Chemicals and solvents were either purchased from commercial suppliers or purified by standard techniques. Thin-layer chromatography plates were visualized by exposure to ultraviolet light and/or staining with ninhydrin hydrate followed by heating on a hot plate. Flash chromatography was carried out using silica gel (200−300 mesh). ^{1}H NMR and ^{13}C NMR spectra were recorded on a Brucker 400 Hz, 500 Hz, or 600 Hz instrument. Data for ^{1}H NMR was presented as the chemical shift in ppm, and multiplicities were denoted as follows: s, singlet; d, doublet; t, triplet; q, quartet; m, multiplet; br, broad. Data for ^{13}C NMR were reported as chemical shift. The ESI mass spectra were determined on a Thermo Fisher FINNIGAN LTQ instrument. All high-resolution mass spectra (HRMS) results were obtained on an Agilent 1290-6545 UHPLC-QTOF LC/MS spectrometer or Thermo Scientific Orbitrap Exploris GC/MS spectrometer. Thin-layer chromatography (TLC) inspections were performed on silica gel plates (GF-254). All commercially available chemicals and solvents were directly used without further purification unless otherwise noted.

Experimental section

Synthesis of

(hexahydrocyclopenta[c]pyrrol-2(1H)-yl)(p-tolyl)-methanone 1hc

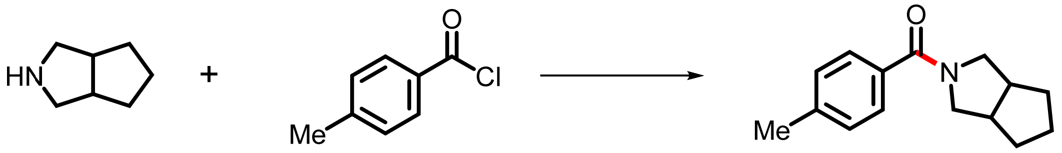

To a solution of octahydrocyclopenta[c]pyrrole (111 mg, 1.0 mmol, 1.0 equiv.) in dicholoromethane (5.0 mL) was slowly added 4-methylbenzoyl chloride (186 mg, 1.2 mmol, 1.2 equiv.) followed by triethylamine (152 mg, 1.5 mmol, 1.5 equiv.) at 0 °C. After complete addition, the reaction was allowed to stir continuously until all the starting material was consumed completely (monitored by TLC, approx. 3-5 h). After completion, the reaction mixture was quenched with water and extracted with dichloromethane. The combined organic layer was washed with brine solution, dried over Na_2SO_4. The product was purified by column chromatography on silica gel to afford pure compound **1hc** (202 mg, 88% yield) as a colorless oil; ^{1}H NMR (400 MHz, $CDCl_3$) δ 7.38 (d, J = 8.1 Hz, 2H), 7.19 (d, J = 7.9 Hz, 2H), 3.84 (s, 1H), 3.71 – 3.40 (m, 2H), 3.22 (s, 1H), 2.66 (d, J = 15.6 Hz, 2H), 2.37 (s, 3H), 1.94 – 1.67 (m, 3H), 1.67 – 1.45 (m, 2H), 1.35 (s, 1H). ^{13}C{^{1}H}NMR (100 MHz, $CDCl_3$) δ 169.63, 139.79, 134.21, 128.82, 127.22, 55.36, 51.83, 43.82, 41.91, 32.32, 31.72, 25.60, 21.38. HRMS (EI): calcd for $C_{15}H_{19}NO$: 229.1467, found: 229.1465.

General procedure for preparation of 8-aminoquinoline amide substrates

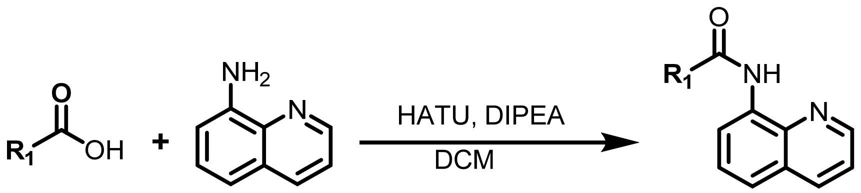

To a mixture of substituted carboxylic acids (1 mmol, 1.0 equiv) in dichloromethane (5 mL) was added 8-aminoquinoline (1.0 mmol, 1.0 equiv), HATU (1.0 mmol, 1.0 equiv) and followed by DIPEA (1.5 mmol, 1.5 equiv). After complete addition, the reaction was allowed to stir continuously until all the starting material was consumed completely (monitored by TLC, approx. 2-5 h). After completion, the reaction mixture was quenched with water and extracted with dichloromethane. The combined organic layer was washed with brine solution, dried over Na_2SO_4. The product was purified by column chromatography on silica gel to afford pure compounds**.**

3-phenyl-N-(quinolin-8-yl)propanamide

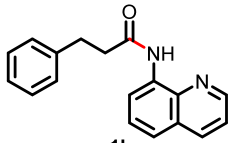

1k

Following the general procedure compound **1k** was obtained from 3-phenylpropanoic acid (150 mg, 1.0 mmol). The crude product was purified by silica-gel column chromatography (DCM/MeOH = 100/1 to 30/1) to afford the title compound **1k**[1] (248 mg, 90%) as a white solid; m.p. 64–66 °C. ^{1}H NMR (400 MHz, $CDCl_3$) δ 10.01 (s, 1H), 8.91 (dd, J = 7.5, 1.1 Hz, 1H), 8.82 (dd, J = 4.2, 1.5 Hz, 1H), 8.16 (dd, J = 8.3, 1.5 Hz, 1H), 7.82 (d, J = 15.6 Hz, 1H), 7.64 – 7.49 (m, 4H), 7.48 – 7.35 (m, 4H), 6.79 (d, J = 15.6 Hz, 1H). $^{13}C\{^1H\}$ NMR (100 MHz, $CDCl_3$) δ 170.74, 148.11, 140.81, 138.33, 136.36, 134.47, 128.58, 128.43, 127.94, 127.44, 126.26, 121.60, 121.46, 116.49, 39.74, 31.50. HRMS (ESI): m/z $[M + H]^+$ calcd for $C_{18}H_{17}N_2O$ 277.1136, found: 277.1134.

N-(quinolin-8-yl)cinnamamide

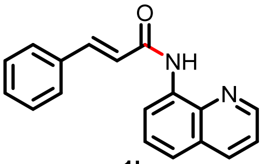

1l

Following the general procedure compound **1l** was obtained from cinnamic acid (148 mg, 1.0 mmol). The crude product was purified by silica-gel column chromatography (DCM/MeOH = 100/1 to 30/1) to afford the title compound **1l**[2] (250 mg, 91%) as a white solid; m.p. 110-113 °C. ^{1}H NMR (400 MHz, $CDCl_3$) δ 10.01 (s, 1H), 8.91 (dd, J = 7.5, 1.1 Hz, 1H), 8.82 (dd, J = 4.2, 1.5 Hz, 1H), 8.16 (dd, J = 8.3, 1.5 Hz, 1H), 7.82 (d, J = 15.6 Hz, 1H), 7.64 – 7.49 (m, 4H), 7.48 – 7.35 (m, 4H), 6.79 (d, J = 15.6 Hz, 1H). $^{13}C\{^1H\}$ NMR (100 MHz, $CDCl_3$) δ 164.15, 148.13, 142.11, 138.41, 136.51, 134.83, 134.65, 129.93, 128.89, 128.07, 128.00, 127.54, 121.69, 121.68, 121.58, 116.93. HRMS (ESI): m/z $[M + H]^+$ calcd for $C_{18}H_{15}N_2O$ 275.1179, found: 275.1176.

(S)-2-(1,3-dioxoisoindolin-2-yl)-3-phenyl-N-(quinolin-8-yl)propanamide

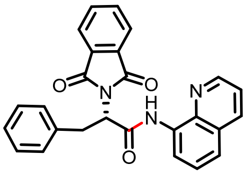

1m

Following the general procedure compound **1m** was obtained from (S)-2-(1,3-dioxoisoindolin-2-yl)-3-phenylpropanoic acid (295 mg, 1.0 mmol). The crude product was purified by silica-gel column chromatography (DCM/MeOH = 100/1 to 30/1) to afford the title compound **1m**[3] (358 mg, 85%) as a white solid; m.p. 122 – 123 °C. ^{1}H NMR (400 MHz, $CDCl_3$) δ 10.25 (s, 1H), 8.66 (dd, J = 6.4, 2.5 Hz, 1H), 8.53 (dd, J = 4.2, 1.6 Hz, 1H), 8.04 (dd, J = 8.3, 1.6 Hz, 1H), 7.74 (dd, J = 5.5, 3.1 Hz, 2H), 7.63 (dd, J = 5.5, 3.1 Hz, 2H), 7.49 – 7.38 (m, 2H), 7.31 (dd, J = 8.3, 4.2 Hz, 1H), 7.22 – 7.06 (m, 5H), 5.38 (dd, J = 9.8, 6.9 Hz, 1H), 3.78 – 3.69 (m, 2H). $^{13}C\{^1H\}$ NMR (100 MHz, $CDCl_3$) δ 166.85, 165.38, 147.24, 137.43, 135.69, 135.22, 133.14, 132.82, 130.60, 127.98, 127.66, 126.80, 126.27, 125.92, 122.51, 120.96, 120.58, 115.74, 55.20, 33.71. HRMS (ESI): m/z $[M + H]^+$ calcd for $C_{26}H_{20}N_3O_3$ 422.1499, found: 422.145.

(S)-2-(1,3-dioxoisoindolin-2-yl)-4-methyl-N-(quinolin-8-yl)pentanamide

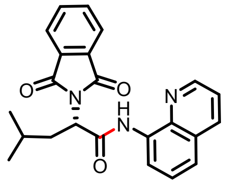

1n

Following the general procedure compound **1n** was obtained from

(S)-2-(1,3-dioxoisoindolin-2-yl)-4-methylpentanoic acid (261 mg, 1.0 mmol). The crude product was purified by silica-gel column chromatography (DCM/MeOH = 100/1 to 30/1) to afford the title compound **1n**[4] (340 mg, 88%) as a white solid; m.p. 103 – 106 °C. ^{1}H NMR (400 MHz, $CDCl_3$) δ 10.37 (s, 1H), 8.82 – 8.68 (m, 2H), 8.17 (dd, J = 8.3, 1.6 Hz, 1H), 7.93 (dd, J = 5.5, 3.1 Hz, 2H), 7.79 (dd, J = 5.5, 3.1 Hz, 2H), 7.59 – 7.50 (m, 2H), 7.45 (dd, J = 8.3, 4.3 Hz, 1H), 5.26 (dd, J = 11.3, 5.0 Hz, 1H), 2.68 (ddd, J = 13.9, 11.4, 4.3 Hz, 1H), 2.20 – 2.08 (m, 1H), 1.67 – 1.58 (m, 1H), 1.07 (dd, J = 10.7, 6.6 Hz, 6H). ^{13}C{^{1}H} NMR (100 MHz, $CDCl_3$) δ 168.17, 167.39, 148.27, 138.42, 136.44, 134.21, 133.93, 131.92, 127.90, 127.38, 123.61, 121.90, 121.60, 116.88, 53.66, 37.42, 25.59, 23.25, 21.32. HRMS (ESI): m/z [M + H]$^+$ calcd for $C_{23}H_{22}N_3O_3$ 388.1656, found: 388.1654.

(S)-2-(1,3-dioxoisoindolin-2-yl)-3-methyl-N-(quinolin-8-yl)butanamide

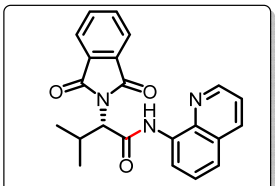

1nn

Following the general procedure compound **1nn** was obtained from (S)-2-(1,3-dioxoisoindolin-2-yl)-3-methylbutanoic acid (247 mg, 1.0 mmol). The crude product was purified by silica-gel column chromatography (DCM/MeOH = 100/1 to 30/1) to afford the title compound **1nn**[3] (317g, 85%) as a white solid; m.p. 112 – 114 °C. ^{1}H NMR (400 MHz, $CDCl_3$) δ 10.58 (s, 1H), 8.93 – 8.68 (m, 2H), 8.13 (dd, J = 8.3, 1.6 Hz, 1H), 7.89 (dd, J = 5.4, 3.1 Hz, 2H), 7.73 (dd, J = 5.5, 3.0 Hz, 2H), 7.58 – 7.36 (m, 3H), 4.70 (d, J = 10.8 Hz, 1H), 3.36 – 3.11 (m, 1H), 1.23 (d, J = 6.6 Hz, 3H), 1.00 (d, J = 6.6 Hz, 3H).^{13}C {^{1}H}NMR (100 MHz, $CDCl_3$) δ 168.14, 166.84, 148.53, 138.72, 136.19, 134.27, 131.63, 127.91, 127.25, 123.66, 121.98, 121.63, 117.00, 77.37, 77.06, 76.74, 63.22, 27.34, 20.48, 19.62. HRMS (ESI): m/z [M + H]$^+$ calcd for $C_{22}H_{20}N_3O_3$ 374.1499, found: 374.1496.

1-(quinolin-8-yl)pyrrolidine-2,5-dione

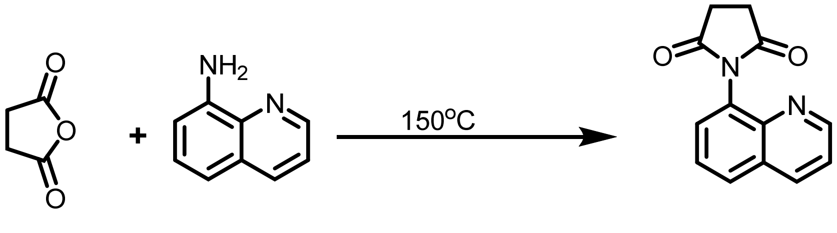

Succinic anhydride (118 mg, 1.18 mmol) and 8-aminoquinoline 5 (190 mg, 1.30 mmol) were dissolved in tetrahydrofuran (5 mL). The solvent was removed in vacuo and the residue was heated at 150 °C for 5 hours. The crude product was purified by silica-gel column chromatography (DCM/MeOH = 150/1 to 50/1) to afford the title compound **1of**[5] (253 mg, 95%) as an off-white solid; m.p. 186-188 °C. 1 H NMR (400 MHz, $CDCl_3$) δ 8.88 (dd, J = 1.6 Hz, J = 4.2 Hz, 1 H), 8.20 (dd, J = 1.6 Hz, J = 8.3 Hz, 1 H), 7.93 (dd, J = 2.1 Hz, J = 7.4 Hz, 1 H), 7.66 – 7.60 (m, 2 H), 7.46 – 7.42 (m, 1 H), 3.20 – 2.91 (m, 4 H). ^{13}C{^{1}H}NMR (100 MHz, $CDCl_3$) δ 176.90, 150.99, 143.47, 136.24, 130.15, 129.78, 129.46, 129.28, 126.14, 122.02, 28.95. HRMS (EI): calcd for $C_{13}H_{10}N_2O_2$: 226.0742, found: 226.0740

1-(quinolin-8-yl)piperidine-2,6-dione

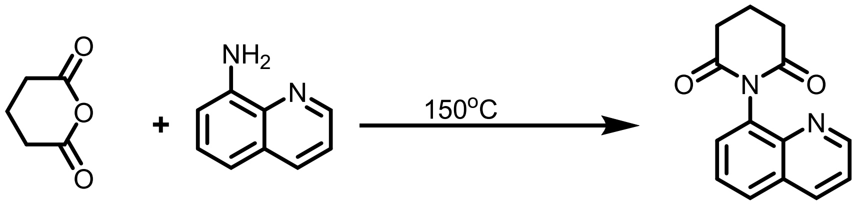

Glutaric anhydride (135 mg, 1.18 mmol) and 8-aminoquinoline 5 (190 mg, 1.30 mmol) were dissolved in tetrahydrofuran (5 mL). The solvent was removed in vacuo and the residue was heated at 150 °C for 5 hours. The crude product was purified by silica-gel column chromatography (DCM/MeOH = 150/1 to 50/1) to afford the title compound **1og**[6] (260 mg, 92%) as a foamy solid. ^{1}H NMR (400 MHz, $CDCl_3$) δ 8.84 (dd, J = 4.1, 1.4 Hz, 1H), 8.19 (d, J = 8.1 Hz, 1H), 7.89 (dd, J = 8.1, 1.1 Hz, 1H), 7.67 – 7.51 (m, 2H), 7.41 (dd, J = 8.2, 4.1 Hz, 1H), 2.99 (dd, J = 15.1, 7.0 Hz, 2H), 2.88 (ddd, J = 17.2, 7.6, 4.9 Hz, 2H), 2.31 (dd, J = 7.9, 5.6 Hz, 1H), 2.24 – 2.09 (m, 1H). ^{13}C{^{1}H}NMR (100 MHz, $CDCl_3$) δ 173.03, 150.77, 143.70, 136.45, 136.32, 133.48, 129.79, 129.22, 129.08, 126.18, 121.69, 33.06, 17.45.
HRMS (EI): calcd for $C_{14}H_{12}N_2O_2$: 240.2620, found: 240.2617.

General procedure for preparation of 3-Azaspiro[5.5]undecane amide substrates

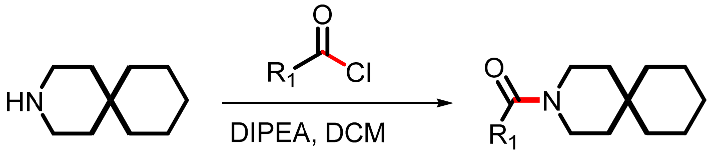

To a solution of 3-azaspiro[5.5]undecane (2.0 mmol, 1.0 equiv.) in dicholoromethane (5.0 mL) was slowly added respective acid chlorides (2.4 mmol, 1.2 equiv.) followed by triethylamine (3.0 mmol, 1.5 equiv.) at 0 °C. After complete addition, the reaction was allowed to stir continuously until all the starting material was consumed completely (monitored by TLC, approx. 3-5 h). After completion, the reaction mixture was quenched with water and extracted with dichloromethane. The combined organic layer was washed with brine solution, dried over Na_2SO_4. The product was purified by column chromatography on silica gel to afford pure compounds.

1-(3-Azaspiro[5.5]undecan-3-yl)ethan-1-one

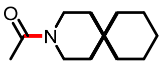

1u

Following the general procedure compound **1u** was obtained from 3-azaspiro[5.5]undecane (306 mg, 2.0 mmol). The crude product was purified by silica-gel column chromatography (EA/PE = 10/1 to 4/1) to afford the title compound **1u** (277 mg, 80%) as a colorless oil;. ^{1}H NMR (400 MHz, $CDCl_3$) δ 3.63 – 3.47 (m, 2H), 3.45 – 3.29 (m, 2H), 2.08 (s, 3H), 1.52 – 1.30 (m, 14H). ^{13}C{^{1}H}NMR (100 MHz, $CDCl_3$) δ 168.86, 42.37, 37.36, 36.11, 31.27, 26.64, 21.51, 21.39. HRMS (EI): calcd for $C_{12}H_{21}NO$: 195.1623, found: 195.1621.

Phenyl(3-azaspiro[5.5]undecan-3-yl)methanone

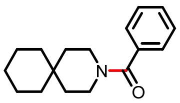

1ub

Following the general procedure compound **1ub** was obtained from 3-azaspiro[5.5]undecane (306 mg, 2.0 mmol). The crude product was purified by silica-gel column chromatography (EA/PE = 10/1 to 4/1) to afford the title compound **1ub** (437 mg, 85%) as a foamy solid. ^{1}H NMR (400 MHz, $CDCl_3$) δ 7.09 (d, J = 8.9 Hz, 2H), 6.90 – 6.79 (m, 2H), 6.78 (s, 1H), 4.15 (q, J = 7.1 Hz, 2H), 3.79 (s, 3H), 1.33 (t, J = 7.1 Hz, 3H). ^{13}C{^{1}H}NMR (100 MHz, $CDCl_3$) δ 170.29, 136.53, 129.35, 128.39, 126.84, 36.22, 31.57, 26.67, 21.42. HRMS (EI): calcd for $C_{17}H_{33}NO$: 257.178, found: 257.176.

2,2-Dimethyl-1-(3-azaspiro[5.5]undecan-3-yl)propan-1-one

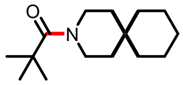

1uc

Following the general procedure compound **1uc** was obtained from 3-azaspiro[5.5]undecane (306 mg, 2.0 mmol). The crude product was purified by silica-gel column chromatography (EA/PE = 10/1 to 4/1) to afford the title compound **1uc** (394 mg, 83%) as a foamy solid. ^{1}H NMR (400 MHz, $CDCl_3$) δ 3.61 – 3.47 (m, 4H), 1.40 (dd, J = 14.9, 9.0 Hz, 14H), 1.27 (s, 9H). ^{13}C{^{1}H}NMR (100 MHz, $CDCl_3$) δ 176.16, 41.02, 38.65, 36.50, 36.39, 31.42, 28.46, 26.71, 21.46. HRMS (EI): calcd for $C_{15}H_{27}NO$: 237.2093, found: 237.2090.

Tert-butyl 3-azaspiro[5.5]undecane-3-carboxylate

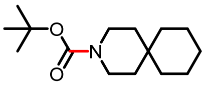

1ud

Following the general procedure compound **1ud** was obtained from 3-azaspiro[5.5]undecane (306 mg, 2.0 mmol). The crude product was purified by silica-gel column chromatography (EA/PE = 10/1 to 4/1) to afford the title compound **1ud** (428 mg, 89%) as a colorless oil;. ^{1}H NMR (400 MHz, $CDCl_3$) δ 3.44 – 3.27 (m, 4H), 1.50 – 1.30 (m, 23H). ^{13}C{^{1}H}NMR (100 MHz, $CDCl_3$) δ 155.05, 79.03, 39.40, 36.12, 31.06, 28.46, 26.73, 21.39. HRMS (EI): calcd for $C_{15}H_{27}NO_2$: 253.2042, found: 253.2040.

Benzyl 3-azaspiro[5.5]undecane-3-carboxylate

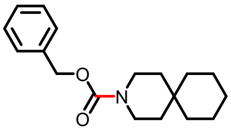

1ue

Following the general procedure compound **1ue** was obtained from 3-azaspiro[5.5]undecane (306 mg, 2.0 mmol). The crude product was purified by silica-gel column chromatography

(EA/PE = 10/1 to 4/1) to afford the title compound **1ue** (436 mg, 89%) as a colorless oil;. ^{1}H NMR (400 MHz, $CDCl_3$) δ 7.43 – 7.20 (m, 5H), 5.12 (s, 2H), 3.50 – 3.37 (m, 4H), 1.51 – 1.26 (m, 14H). ^{13}C{^{1}H}NMR (100 MHz, $CDCl_3$) δ 155.46, 137.08, 128.46, 127.80, 66.88, 39.73, 36.10, 31.11, 26.71, 21.40. HRMS (EI): calcd for $C_{18}H_{25}NO_2$: 287.1885, found: 287.1882.

General procedure for preparation of amino acids derivative with protective group substrates

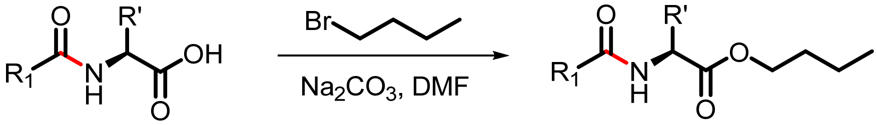

To a solution of amino acids (1.0 equiv.) derivatives in DMF (0.2 M) was added Na_2CO_3 (1.0 equiv.), n-bromobutane (1.2 equiv.) and heated for 2−3h at 65°C in a sealed vial under an atmosphere of N_2 (monitored by TLC). Then the mixture was cooled to room temperature, quenched water, extracted with ethyl acetate, and washed with brine. The organic phase was dried over anhydrous Na_2SO_4 and concentrated. The residue was purified by column chromatography on silica gel to give pure products.

Butyl (tert-butoxycarbonyl)-L-phenylalaninate

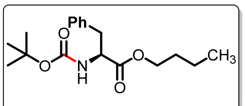

1W

Following the general procedure compound **1w** was obtained from (tert-butoxycarbonyl)-L-phenylalanine (265 mg, 1.0 mmol). The crude product was purified by silica-gel column chromatography (EA/PE = 6/1 to 2/1) to afford the title compound **1w**[7] (290 mg, 91%) as a foamy solid. ^{1}H NMR (400 MHz, $CDCl_3$) δ 7.33 – 7.25 (m, 3H), 7.16 (d, J = 6.9 Hz, 2H), 5.00 (d, J = 7.6 Hz, 1H), 4.59 (d, J = 7.4 Hz, 1H), 4.17 – 4.11 (m, 2H), 3.10 (t, J = 6.9 Hz, 2H), 1.63 – 1.55 (m, 2H), 1.44 (s, 9H), 1.29 (dd, J = 14.2, 7.0 Hz, 2H), 0.94 (t, J = 7.4 Hz, 3H). ^{13}C{^{1}H}NMR (100 MHz, $CDCl_3$) δ 171.97, 155.08, 136.12, 129.34, 128.49, 126.95, 79.82, 65.20, 54.48, 38.47, 30.51, 28.30, 19.04, 13.64.
HRMS (ESI): m/z $[M + H]^+$ calcd for $C_{18}H_{28}NO_4$ 322.2013, found: 322.2011.

Butyl ((benzyloxy)carbonyl)-L-phenylalaninate

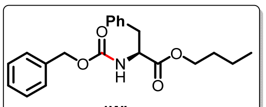

1Wb

Following the general procedure compound **1wb** was obtained from ((benzyloxy)carbonyl)-L-phenylalanine (300 mg, 1.0 mmol). The crude product was purified by silica-gel column

chromatography (EA/PE = 6/1 to 2/1) to afford the title compound **1wb**[8] (327 mg, 92%) as a foamy solid. ^{1}H NMR (400 MHz, $CDCl_3$) δ 7.42 – 7.19 (m, 8H), 7.17 – 7.04 (m, 2H), 5.24 (d, J = 8.0 Hz, 1H), 5.09 (s, 2H), 4.65 (dd, J = 14.0, 6.0 Hz, 1H), 4.20 – 4.00 (m, 2H), 3.10 (t, J = 6.1 Hz, 2H), 1.56 (dd, J = 14.5, 7.0 Hz, 2H), 1.31 (dd, J = 15.0, 7.4 Hz, 2H), 0.91 (t, J = 7.4 Hz, 3H). ^{13}C{^{1}H}NMR (100 MHz, $CDCl_3$) δ 171.61, 155.63, 136.32, 135.80, 129.33, 128.58, 128.54, 128.19, 128.10, 127.10, 66.95, 65.39, 54.88, 38.36, 30.48, 19.05, 13.66. HRMS (ESI): m/z [M + H]$^+$ calcd for $C_{21}H_{26}NO_4$ 356.1856, found: 356.1852.

Butyl (tert-butoxycarbonyl)-L-leucinate

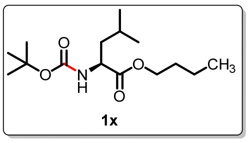

Following the general procedure compound **1x** was obtained from (tert-butoxycarbonyl)-L-leucine (231 mg, 1.0 mmol). The crude product was purified by silica-gel column chromatography (EA/PE = 6/1 to 2/1) to afford the title compound **1x** (256 mg, 90%) as a foamy solid. ^{1}H NMR (400 MHz, $CDCl_3$) δ 4.88 (d, J = 7.7 Hz, 1H), 4.30 (d, J = 5.4 Hz, 1H), 4.12 (td, J = 6.7, 2.8 Hz, 2H), 1.71 (dd, J = 14.1, 6.6 Hz, 1H), 1.62 (dt, J = 8.5, 6.7 Hz, 3H), 1.53 – 1.47 (m, 1H), 1.44 (s, 9H), 1.40 – 1.33 (m, 2H), 0.96 – 0.93 (m, 9H). ^{13}C{^{1}H}NMR (100 MHz, $CDCl_3$) δ 173.45, 155.17, 79.67, 65.04, 52.17, 41.98, 30.59, 28.33, 24.81, 22.81, 22.00, 19.08, 13.67. HRMS (ESI): m/z [M + H]$^+$ calcd for $C_{15}H_{30}NO_4$ 288.2169, found: 288.2165.

Butyl ((benzyloxy)carbonyl)-L-leucinate

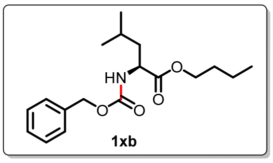

Following the general procedure compound **1xb** was obtained from ((benzyloxy)carbonyl)-L-leucine (265 mg, 1.0 mmol). The crude product was purified by silica-gel column chromatography (EA/PE = 6/1 to 2/1) to afford the title compound **1xb**[8] (282 mg, 88%) as a foamy solid. ^{1}H NMR (400 MHz, $CDCl_3$) δ 7.38 – 7.30 (m, 5H), 5.13 (d, J = 13.1 Hz, 3H), 4.38 (dd, J = 14.0, 8.7 Hz, 1H), 4.13 (t, J = 6.6 Hz, 2H), 1.74 – 1.58 (m, 4H), 1.55 – 1.47 (m, 1H), 1.38 (dd, J = 14.9, 7.4 Hz, 2H), 0.94 (q, J = 6.7 Hz, 9H). ^{13}C{^{1}H}NMR (100 MHz, $CDCl_3$) δ 173.20, 155.94, 136.31, 128.53, 128.17, 128.09, 66.97, 65.22, 52.61, 41.98, 30.55, 24.78, 22.80, 21.95, 19.07, 13.67.
HRMS (ESI): m/z [M + H]$^+$ calcd for $C_{18}H_{28}NO_4$ 322.2013, found: 322.2010.

General procedure for amide esterification

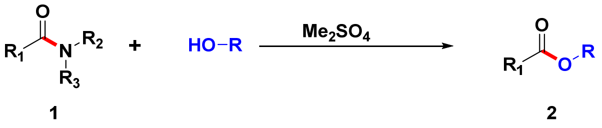

To a solution of amide **1** (1.0 equiv) in alcohols (0.2 M) was added dimethyl sulfate (1.0 equiv), and heated for 8−24h at 65−120°C in a sealed vial under an atmosphere of N_2

(monitored by TLC). The resulting mixture concentrated in vacuo to give residues. Then the residues were dissolved in ethyl acetate (30 V) and washed with brine, dried over Na_2SO_4, filtered, and concentrated in vacuo. The crude product was purified by silica-gel column chromatography to give ester product **2.**

Methyl benzoate[9]

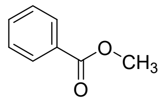

2a

Colorless oil; ^{1}H NMR (400 MHz, $CDCl_3$) δ 8.03 (dd, J = 8.3, 1.2 Hz, 2H), 7.52 (t, J = 7.4 Hz, 1H), 7.41 (t, J = 7.7 Hz, 2H), 3.89 (s, 3H). ^{13}C{^{1}H}NMR (100 MHz, $CDCl_3$) δ 167.03, 132.87, 130.18, 129.55, 128.33, 52.01. HRMS (EI): m/z [M] calcd for $C_8H_8O_2$ 136.0524, found: 136.0522.

Ethyl benzoate[10]

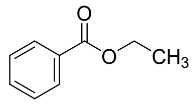

2ab

Colorless oil; ^{1}H NMR (400 MHz, $CDCl_3$) δ 8.05 (dt, J = 8.5, 1.6 Hz, 2H), 7.60 – 7.50 (m, 1H), 7.49 – 7.36 (m, 2H), 4.38 (q, J = 7.1 Hz, 2H), 1.39 (t, J = 7.1 Hz, 3H). ^{13}C{^{1}H} NMR (100 MHz, $CDCl_3$) δ 166.64, 132.80, 130.52, 129.54, 128.32, 60.95, 14.34. HRMS (EI): m/z [M] calcd for $C_9H_{10}O_2$ 150.0681, found: 150.0678.

Butyl benzoate[10]

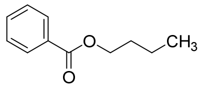

2ac

Colorless oil; ^{1}H NMR (400 MHz, $CDCl_3$) δ 8.13 – 7.95 (m, 2H), 7.61 – 7.51 (m, 1H), 7.49 – 7.38 (m, 2H), 4.33 (t, J = 6.6 Hz, 2H), 1.76 (dt, J = 14.5, 6.7 Hz, 2H), 1.58 – 1.40 (m, 2H), 0.98 (t, J = 7.4 Hz, 3H). ^{13}C{^{1}H} NMR (100 MHz, $CDCl_3$) δ 166.71, 132.79, 130.56, 129.54, 128.32, 64.84, 30.80, 19.29, 13.77. HRMS (EI): m/z [M] calcd for $C_{11}H_{14}O_2$ 178.0994, found: 178.0992.

Benzyl benzoate[11]

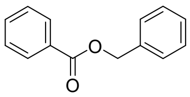

2ad

Colorless oil; ^{1}H NMR (400 MHz, $CDCl_3$) δ 8.16 – 8.01 (m, 2H), 7.65 – 7.50 (m, 1H), 7.49 – 7.29 (m, 7H), 5.37 (s, 2H). ^{13}C{^{1}H} NMR (100 MHz, $CDCl_3$) δ 166.46, 136.10, 133.05, 130.18, 129.73, 128.62, 128.40, 128.26, 128.19, 66.71. HRMS (EI): m/z [M] calcd for $C_{14}H_{12}O_2$ 212.0837, found: 212.0835.

Isobutyl benzoate[12]

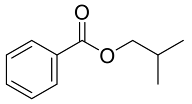

2ae

Colorless oil; ^{1}H NMR (400 MHz, $CDCl_3$) δ 8.11 – 8.00 (m, 2H), 7.61 – 7.51 (m, 1H), 7.51 – 7.36 (m, 2H), 4.11 (d, J = 6.6 Hz, 2H), 2.09 (dt, J = 13.4, 6.7 Hz, 1H), 1.03 (d, J = 6.7 Hz, 6H). ^{13}C{^{1}H} NMR (100 MHz, $CDCl_3$) δ 166.65, 132.82, 130.55, 129.55, 128.34, 71.02, 27.93, 19.21. HRMS (EI): m/z [M] calcd

for $C_{11}H_{14}O_2$ 178.0994, found: 178.0991.

2-hydroxyethyl benzoate[13]

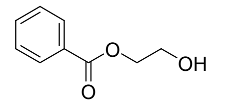

2af

Colorless oil; ^{1}H NMR (400 MHz, $CDCl_3$) δ 8.05 (dd, J = 5.2, 3.3 Hz, 2H), 7.65 – 7.49 (m, 1H), 7.44 (dd, J = 10.7, 4.7 Hz, 2H), 4.50 – 4.39 (m, 2H), 4.04 – 3.88 (m, 2H), 2.29 (brs, 1H). $^{13}C\{^1H\}$ NMR (100 MHz, $CDCl_3$) δ 166.99, 133.19, 129.87, 129.69, 128.42, 66.67, 61.37. HRMS (EI): m/z [M] calcd for $C_9H_{10}O_3$ 166.0630, found: 166.0628.

Isopropyl benzoate[10]

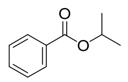

2ag

Colorless oil; ^{1}H NMR (400 MHz, $CDCl_3$) δ 8.04 (dd, J = 5.2, 3.4 Hz, 2H), 7.63 – 7.49 (m, 1H), 7.42 (dd, J = 10.6, 4.6 Hz, 2H), 5.26 (dt, J = 12.5, 6.3 Hz, 1H), 1.37 (d, J = 6.3 Hz, 6H). $^{13}C\{^1H\}$ NMR (100 MHz, $CDCl_3$) δ 166.12, 132.69, 130.93, 129.51, 128.26, 68.33, 21.96. HRMS (EI): m/z [M] calcd for $C_{10}H_{12}O_3$ 164.0837, found: 164.0835.

Sec-butyl benzoate[14]

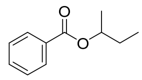

2ah

Colorless oil; ^{1}H NMR (400 MHz, $CDCl_3$) δ 8.04 (dd, J = 5.2, 3.3 Hz, 2H), 7.62 – 7.48 (m, 1H), 7.48 – 7.35 (m, 2H), 5.10 (dd, J = 12.5, 6.3 Hz, 1H), 1.83 – 1.57 (m, 2H), 1.34 (d, J = 6.3 Hz, 3H), 0.98 (t, J = 7.5 Hz, 3H). $^{13}C\{^1H\}$ NMR (100 MHz, $CDCl_3$) δ 166.25, 132.68, 130.95, 129.51, 128.28, 72.85, 28.97, 19.57, 9.74. HRMS (EI): m/z [M] calcd for $C_{11}H_{14}O_2$ 178.0994, found: 178.0993.

Cyclohexyl benzoate[15]

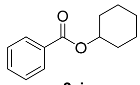

2ai

Colorless oil; ^{1}H NMR (400 MHz, $CDCl_3$) δ 8.11 – 7.99 (m, 2H), 7.54 (dd, J = 10.5, 4.3 Hz, 1H), 7.43 (t, J = 7.6 Hz, 2H), 5.12 – 4.92 (m, 1H), 2.04 – 1.87 (m, 2H), 1.85 – 1.72 (m, 2H), 1.59 (ddd, J = 12.6, 8.1, 3.4 Hz, 3H), 1.42 (ddd, J = 31.0, 9.5, 3.1 Hz, 3H). $^{13}C\{^1H\}$ NMR (100 MHz, $CDCl_3$) δ 166.00, 132.67, 131.05, 129.54, 128.26, 73.03, 31.65, 25.50, 23.67. HRMS (EI): m/z [M] calcd for $C_{13}H_{16}O_2$ 204.1150, found: 204.1148.

Tert-butyl benzoate[10]

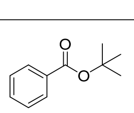

2aj

Colorless oil; ^{1}H NMR (400 MHz, $CDCl_3$) δ 7.99 (dd, J = 5.2, 3.3 Hz, 2H), 7.51 (dd, J = 5.0, 3.7 Hz, 1H), 7.46 – 7.37 (m, 2H), 1.60 (s, 9H). $^{13}C\{^1H\}$ NMR (100 MHz, $CDCl_3$) δ 165.79, 132.40, 132.04, 129.41, 128.17, 80.96, 28.21. HRMS (EI): m/z [M] calcd for $C_{11}H_{14}O_2$ 178.0994, found: 178.0992.

Methyl 4-methoxybenzoate[10]

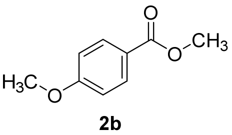

2b

Colorless oil; ^{1}H NMR (400 MHz, $CDCl_3$) δ 8.05 – 7.93 (m, 2H), 7.01 – 6.81 (m, 2H), 3.88 (s, 3H), 3.85 (s, 3H). $^{13}C\{^1H\}$NMR

(100 MHz, $CDCl_3$) δ 166.85, 163.34, 131.58, 122.63, 113.60, 55.40, 51.83. HRMS (EI): m/z [M] calcd for $C_9H_{10}O_3$ 166.0630, found: 166.0627.

Ethyl 4-methoxybenzoate[16]

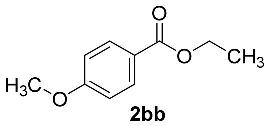
2bb

Colorless oil; ^1H NMR (400 MHz, $CDCl_3$) δ 8.10 – 7.92 (m, 2H), 7.01 – 6.82 (m, 2H), 4.35 (q, J = 7.1 Hz, 2H), 3.85 (s, 3H), 1.38 (t, J = 7.1 Hz, 3H). $^{13}C\{^1H\}$ NMR (100 MHz, $CDCl_3$) δ 166.40, 163.26, 131.54, 122.98, 113.55, 60.63, 55.41, 14.39. HRMS (EI): m/z [M] calcd for $C_{10}H_{12}O_3$ 180.0786, found: 180.0785.

Butyl 4-methoxybenzoate[17]

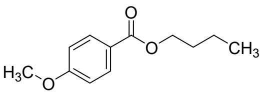
2bc

Colorless oil; ^1H NMR (400 MHz, $CDCl_3$) δ 8.07 – 7.93 (m, 2H), 7.00 – 6.83 (m, 2H), 4.29 (t, J = 6.6 Hz, 2H), 3.85 (s, 3H), 1.74 (dt, J = 14.6, 6.7 Hz, 2H), 1.47 (dd, J = 15.0, 7.5 Hz, 2H), 0.98 (t, J = 7.4 Hz, 3H). $^{13}C\{^1H\}$ NMR (100 MHz, $CDCl_3$) δ 166.46, 163.25, 131.54, 123.00, 113.56, 64.53, 55.41, 30.85, 19.31, 13.79. HRMS (EI): m/z [M] calcd for $C_{12}H_{16}O_3$ 208.1099, found: 208.1096.

Methyl 4-chlorobenzoate[10]

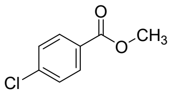
2c

Colorless oil; ^1H NMR (400 MHz, $CDCl_3$) δ 7.09 (d, J = 8.9 Hz, 2H), 6.90 – 6.79 (m, 2H), 6.78 (s, 1H), 4.15 (q, J = 7.1 Hz, 2H), 3.79 (s, 3H), 1.33 (t, J = 7.1 Hz, 3H). $^{13}C\{^1H\}$NMR (100 MHz, $CDCl_3$) δ 156.35, 155.88, 131.09, 122.72, 114.14, 62.98, 55.50, 14.44.

HRMS (EI): m/z [M] calcd for $C_8H_7ClO_2$ 170.0135, found: 170.0133.

Ethyl 4-chlorobenzoate[18]

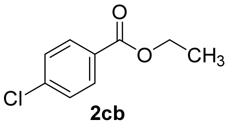
2cb

Colorless oil; ^1H NMR (400 MHz, $CDCl_3$) δ 8.06 – 7.90 (m, 2H), 7.49 – 7.33 (m, 2H), 4.37 (q, J = 7.1 Hz, 2H), 1.39 (t, J = 7.1 Hz, 3H). $^{13}C\{^1H\}$ NMR (100 MHz, $CDCl_3$) δ 165.75, 139.24, 130.94, 128.96, 128.65, 61.21, 14.29. HRMS (EI): m/z [M] calcd for $C_9H_9ClO_2$ 184.0291, found: 184.0290.

Butyl 4-chlorobenzoate[19]

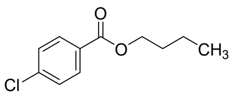
2cc

Colorless oil; ^1H NMR (400 MHz, $CDCl_3$) δ 8.05 – 7.89 (m, 2H), 7.51 – 7.34 (m, 2H), 4.32 (t, J = 6.6 Hz, 2H), 1.75 (dt, J = 14.6, 6.7 Hz, 2H), 1.47 (dd, J = 15.0, 7.5 Hz, 2H), 0.98 (t, J = 7.4 Hz, 3H). $^{13}C\{^1H\}$ NMR (100 MHz, $CDCl_3$) δ 165.82, 139.24, 130.94, 128.99, 128.67, 65.10, 30.74, 19.26, 13.75. HRMS (EI): m/z [M] calcd for $C_{11}H_{13}ClO_2$ 212.0604, found: 212.0601.

Methyl 4-nitrobenzoate[20]

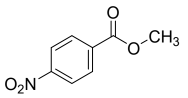
2d

As a foamy solid; ^1H NMR (400 MHz, $CDCl_3$) δ 8.30 (d, J =

8.9 Hz, 2H), 8.22 (d, *J* = 8.9 Hz, 2H), 3.99 (s, 3H). $^{13}C\{^1H\}$NMR (100 MHz, $CDCl_3$) *δ* 165.19, 150.57, 135.51, 130.73, 123.57, 52.85.
HRMS (EI): m/z [M] calcd for $C_8H_7NO_4$ 181.0375, found: 181.0373.

Butyl 4-nitrobenzoate[17]

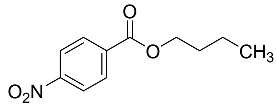

2db

As a foamy solid; ^1H NMR (400 MHz, $CDCl_3$) *δ* 8.29 (d, *J* = 9.0 Hz, 2H), 8.21 (d, *J* = 9.0 Hz, 2H), 4.38 (t, *J* = 6.6 Hz, 2H), 1.77 (dd, *J* = 14.8, 6.9 Hz, 2H), 1.54 – 1.43 (m, 2H), 0.99 (t, *J* = 7.4 Hz, 3H). $^{13}C\{^1H\}$ NMR (100 MHz, $CDCl_3$) *δ* 164.78, 150.50, 135.90, 130.67, 123.53, 65.84, 30.65, 19.23, 13.73. HRMS (EI): m/z [M] calcd for $C_{11}H_{13}NO_4$ 223.0845, found: 223.0841.

Methyl cyclohexanecarboxylate[10]

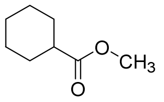

2e

Colorless oil; ^1H NMR (400 MHz, $CDCl_3$) *δ* 3.66 (s, 3H), 2.30 (ddd, *J* = 11.3, 7.6, 3.6 Hz, 1H), 1.90 (dd, *J* = 13.0, 2.6 Hz, 2H), 1.81 – 1.69 (m, 2H), 1.68 – 1.57 (m, 1H), 1.43 (td, *J* = 11.7, 5.8 Hz, 2H), 1.35 – 1.13 (m, 3H). $^{13}C\{^1H\}$NMR (100 MHz, $CDCl_3$) *δ* 176.58, 51.43, 43.11, 29.02, 25.75, 25.45.
HRMS (EI): m/z [M] calcd for $C_8H_{14}O_2$ 142.0994, found: 142.0992.

Ethyl cyclohexanecarboxylate[21]

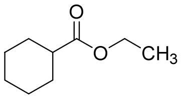

2eb

Colorless oil; ^1H NMR (400 MHz, $CDCl_3$) *δ* 4.11 (q, *J* = 7.1 Hz, 2H), 2.28 (tt, *J* = 11.3, 3.6 Hz, 1H), 1.90 (dd, *J* = 13.0, 2.5 Hz, 2H), 1.81 – 1.69 (m, 2H), 1.69 – 1.57 (m, 1H), 1.53 – 1.37 (m, 2H), 1.35 – 1.15 (m, 6H). $^{13}C\{^1H\}$NMR (100 MHz, $CDCl_3$) *δ* 176.15, 60.01, 43.24, 29.02, 25.78, 25.46, 14.23.
HRMS (EI): m/z [M] calcd for $C_9H1_6O_2$ 156.1150, found: 156.1147.

Butyl cyclohexanecarboxylate[22]

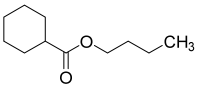

2ec

Colorless oil; ^1H NMR (400 MHz, $CDCl_3$) *δ* 4.06 (t, *J* = 6.6 Hz, 2H), 2.36 – 2.20 (m, 1H), 1.90 (d, *J* = 12.9 Hz, 2H), 1.79 – 1.71 (m, 2H), 1.61 (dd, *J* = 14.2, 7.4 Hz, 3H), 1.48 – 1.32 (m, 4H), 1.32 – 1.20 (m, 3H), 0.93 (t, *J* = 7.4 Hz, 3H). $^{13}C\{^1H\}$ NMR (100 MHz, $CDCl_3$) *δ* 176.23, 63.95, 43.29, 30.74, 29.05, 25.79, 25.47, 19.16, 13.72. HRMS (EI): m/z [M] calcd for $C_{11}H_{14}O_2$ 184.1463, found: 184.1460.

Methyl 2-cyclohexylacetate[23]

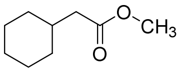

2f

Colorless oil; ^1H NMR (400 MHz, $CDCl_3$) *δ* 3.66 (s, 3H), 2.19 (d, *J* = 7.0 Hz, 2H), 1.84 – 1.57 (m, 6H), 1.40 – 1.06 (m, 3H), 1.05 – 0.85 (m, 2H). $^{13}C\{^1H\}$NMR (100 MHz, $CDCl_3$) *δ* 173.61, 51.33, 41.98, 34.89, 33.03, 26.14, 26.02. HRMS (EI): m/z [M] calcd for

$C_9H_{16}O_2$ 156.1150, found: 56.1149.

Butyl 2-cyclohexylacetate

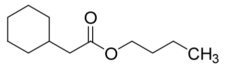

2fb

Colorless oil; ^{1}H NMR (400 MHz, $CDCl_3$) δ 4.06 (t, J = 6.7 Hz, 2H), 2.17 (d, J = 7.0 Hz, 2H), 1.82 – 1.58 (m, 8H), 1.43 – 1.09 (m, 5H), 0.94 (dd, J = 9.7, 5.0 Hz, 5H). $^{13}C\{^1H\}$ NMR (100 MHz, $CDCl_3$) δ 173.29, 64.01, 42.27, 34.95, 33.03, 30.73, 26.17, 26.04, 19.17, 13.70. HRMS (EI): m/z [M] calcd for $C_{12}H_{16}O_2$ 198.1620, found: 198.1616.

Methyl nicotinate[24]

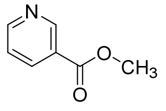

2g

As a foamy solid; ^{1}H NMR (400 MHz, $CDCl_3$) δ 9.23 (d, J = 0.6 Hz, 1H), 8.90 – 8.66 (m, 1H), 8.43 – 8.20 (m, 1H), 7.40 (dt, J = 8.0, 4.1 Hz, 1H), 3.97 (d, J = 4.5 Hz, 3H). $^{13}C\{^1H\}$NMR (100 MHz, $CDCl_3$) δ 165.71, 153.41, 150.89, 136.99, 126.00, 123.26, 52.38. HRMS (EI): m/z [M] calcd for $C_7H_7NO_2$ 137.0477, found: 137.0475.

Butyl nicotinate[22]

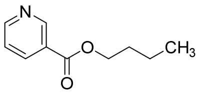

2gb

Colorless oil; ^{1}H NMR (400 MHz, $CDCl_3$) δ 9.23 (d, J = 1.6 Hz, 1H), 8.78 (dd, J = 4.8, 1.7 Hz, 1H), 8.30 (dt, J = 7.9, 1.9 Hz, 1H), 7.40 (ddd, J = 7.9, 4.9, 0.6 Hz, 1H), 4.37 (t, J = 6.6 Hz, 2H), 1.78 (dt, J = 14.5, 6.7 Hz, 2H), 1.57 – 1.40 (m, 2H), 0.99 (t, J = 7.4 Hz, 3H). $^{13}C\{^1H\}$ NMR (100 MHz, $CDCl_3$) δ 165.31, 153.27, 150.86, 137.03, 126.38, 123.26, 65.30, 30.67, 19.21, 13.71. HRMS (EI): m/z [M] calcd for $C_{11}H_{14}O_2$ 179.0946, found: 179.0943.

Methyl 4-methylbenzoate[10]

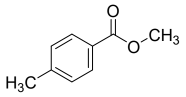

2h

As a foamy solid; ^{1}H NMR (400 MHz, $CDCl_3$) δ .93 (d, J = 8.2 Hz, 2H), 7.23 (d, J = 8.3 Hz, 2H), 3.89 (s, 3H), 2.40 (s, 3H). $^{13}C\{^1H\}$NMR (100 MHz, $CDCl_3$) δ 167.18, 143.55, 129.60, 129.08, 127.45, 77.37, 77.05, 76.73, 51.93, 21.64. HRMS (EI): m/z [M] calcd for $C_9H_{10}O_2$ 150.0681, found: 150.0680.

Butyl 4-methylbenzoate[25]

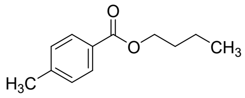

2hb

Colorless oil; ^{1}H NMR (400 MHz, $CDCl_3$) δ 7.93 (d, J = 8.2 Hz, 2H), 7.31 – 7.17 (m, 2H), 4.31 (t, J = 6.6 Hz, 2H), 2.40 (s, 3H), 1.74 (dd, J = 14.8, 6.9 Hz, 2H), 1.48 (dd, J = 15.0, 7.5 Hz, 2H), 0.98 (t, J = 7.4 Hz, 3H). $^{13}C\{^1H\}$ NMR (100 MHz, $CDCl_3$) δ 166.78, 143.40, 129.57, 129.03, 127.83, 64.65, 30.82, 21.64, 19.30, 13.78. HRMS (EI): m/z [M] calcd for $C_{12}H_{16}O_2$ 192.1150, found: 192.1147.

Methyl 1-naphthoate[10]

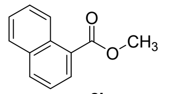

2i

As a foamy solid; ^{1}H NMR (400 MHz, $CDCl_3$) δ 8.91 (d, J = 8.0 Hz, 1H), 8.18 (d, J = 8.0 Hz, 1H), 8.01 (d, J = 8.0 Hz, 1H), 7.87 (d, J = 8.0 Hz, 1H), 7.64−7.58 (m, 1H), 7.56− 7.45 (m, 2H), 3.99 (s, 3H). $^{13}C\{^1H\}$NMR (100 MHz, $CDCl_3$) δ 167.29, 135.53, 132.51, 131.09, 129.38, 128.26, 128.17, 127.78, 127.41, 126.66, 125.25, 52.27. HRMS (EI): m/z [M] calcd for $C_{12}H_{10}O_2$ 186.0681, found: 150.0678.

Butyl 1-naphthoate[25]

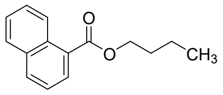

2ib

Colorless oil; ^{1}H NMR (400 MHz, $CDCl_3$) δ 8.60 (s, 1H), 8.07 (dd, J = 8.6, 1.7 Hz, 1H), 7.96 (d, J = 8.0 Hz, 1H), 7.88 (d, J = 8.6 Hz, 2H), 7.56 (dtd, J = 16.1, 6.9, 1.3 Hz, 2H), 4.39 (t, J = 6.6 Hz, 2H), 1.81 (dt, J = 14.5, 6.7 Hz, 2H), 1.52 (dt, J = 16.6, 7.5 Hz, 2H), 1.01 (t, J = 7.4 Hz, 3H). $^{13}C\{^1H\}$ NMR (100 MHz, $CDCl_3$) δ 166.87, 135.50, 132.53, 130.94, 129.36, 128.16, 128.10, 127.81, 127.77, 126.60, 125.28, 65.01, 30.86, 19.34, 13.81. HRMS (EI): m/z [M] calcd for $C_{15}H_{16}O_2$ 228.1150, found: 228.1148.

Methyl 4-fluorobenzoate[10]

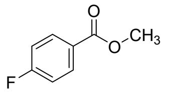

2j

As a foamy solid; ^{1}H NMR (400 MHz, $CDCl_3$) δ 8.16 – 7.94 (m, 2H), 7.13 (t, J = 8.7 Hz, 2H), 3.94 (s, 3H). $^{13}C\{^1H\}$NMR (100 MHz, $CDCl_3$) δ 166.14, 165.76 (d, J = 253.7 Hz), 132.11 (d, J = 9.2 Hz), 126.43 (d, J = 3.0 Hz), 115.50 (d, J = 22.0 Hz), 52.17. HRMS (EI): m/z [M] calcd for $C_8H_7FO_2$ 154.0430, found: 154.0428.

Ethyl 4-fluorobenzoate[26]

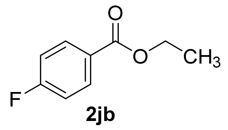

2jb

Colorless oil; ^{1}H NMR (400 MHz, $CDCl_3$) δ 8.13 – 8.01 (m, 2H), 7.17 – 7.02 (m, 2H), 4.38 (q, J = 7.1 Hz, 2H), 1.40 (t, J = 7.1 Hz, 3H). $^{13}C\{^1H\}$ NMR (100 MHz, $CDCl_3$) δ 165.68 (d, J = 253.5 Hz), 165.61, 132.04 (d, J = 9.4 Hz), 126.76 (d, J = 3.0 Hz), 115.39 (d, J = 22.0 Hz), 61.05, 14.27. HRMS (EI): m/z [M] calcd for $C_9H_9FO_2$ 168.0587, found: 168.0585.

Butyl 4-fluorobenzoate[27]

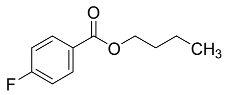

2jc

Colorless oil; ^{1}H NMR (400 MHz, $CDCl_3$) δ 8.15 – 7.98 (m, 2H), 7.10 (t, J = 8.7 Hz, 2H), 4.32 (t, J = 6.6 Hz, 2H), 1.74 (dd, J = 14.8, 6.8 Hz, 2H), 1.47 (dd, J = 15.0, 7.5 Hz, 2H), 0.98 (t, J = 7.4 Hz, 3H). $^{13}C\{^1H\}$ NMR (100 MHz, $CDCl_3$) δ 165.69 (d, J = 253.3 Hz), 165.57, 132.04 (d, J = 9.4 Hz), 126.77 (d, J = 3.0 Hz), 115.42 (d, J = 21.9 Hz), 64.96, 30.76, 19.26, 13.73. HRMS (EI): m/z [M] calcd for $C_{11}H_{13}FO_2$ 196.0900, found: 196.0897.

Butyl 3-phenylpropanoate[28]

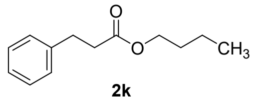

2k

Colorless oil; ^{1}H NMR (400 MHz, $CDCl_3$) δ 7.37 – 7.18 (m, 5H), 4.10 (t, J = 6.7 Hz, 2H), 2.98 (t, J = 7.8 Hz, 2H), 2.65 (t, J = 7.8 Hz, 2H), 1.61 (dt, J = 14.6, 6.8 Hz, 2H), 1.37 (dd, J = 15.0, 7.5 Hz, 2H), 0.94 (t, J = 7.4 Hz, 3H). $^{13}C\{^1H\}$ NMR (100 MHz, $CDCl_3$) δ 173.04, 140.60, 128.49, 128.31, 126.24, 64.37, 35.96, 31.03, 30.67, 19.12, 13.72. HRMS (EI): m/z [M] calcd for $C_{13}H_{18}O_2$ 206.1307, found: 206.1305.

Butyl cinnamate[29]

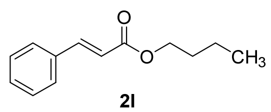

2l

As a white solid; m.p. 140–143 °C. ^{1}H NMR (400 MHz, $CDCl_3$) δ 7.68 (d, J = 16.0 Hz, 1H), 7.53 (dd, J = 6.8, 2.8 Hz, 2H), 7.42 – 7.32 (m, 3H), 6.44 (d, J = 16.0 Hz, 1H), 4.21 (t, J = 6.7 Hz, 2H), 1.70 (dt, J = 14.6, 6.8 Hz, 2H), 1.51 – 1.37 (m, 2H), 0.97 (t, J = 7.4 Hz, 3H). $^{13}C\{^1H\}$ NMR (100 MHz, $CDCl_3$) δ 167.13, 144.57, 134.50, 130.22, 128.89, 128.07, 118.32, 64.46, 30.80, 19.22, 13.78. HRMS (EI): m/z [M] calcd for $C_{13}H_{16}O_2$ 204.1150, found: 204.1146.

Methyl (S)-2-(1,3-dioxoisoindolin-2-yl)-3-phenylpropanoate[30]

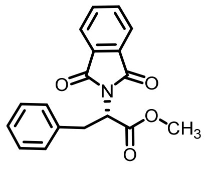

2m

As a white solid; m.p. 115–118 °C. ^{1}H NMR (400 MHz, $CDCl_3$) δ 7.77 (dt, J = 7.0, 3.5 Hz, 2H), 7.73 – 7.63 (m, 2H), 7.24 – 7.06 (m, 5H), 5.16 (dd, J = 11.2, 5.3 Hz, 1H), 3.78 (s, 3H), 3.57 (qd, J = 14.3, 8.3 Hz, 2H). $^{13}C\{^1H\}$NMR (100 MHz, $CDCl_3$) δ 169.36, 167.44, 136.71, 134.09, 131.59, 128.84, 128.56, 126.85, 123.47, 53.27, 52.90, 34.66. HRMS (EI): m/z [M] calcd for $C_{18}H_{15}NO_4$ 309.1001, found: 309.1000.

Butyl (S)-2-(1,3-dioxoisoindolin-2-yl)-3-phenylpropanoate

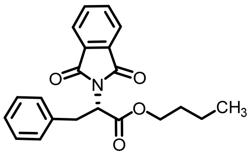

2mb

As a foamy solid; ^{1}H NMR (400 MHz, $CDCl_3$) δ 7.77 (td, J = 5.2, 2.0 Hz, 2H), 7.69 (td, J = 5.2, 2.0 Hz, 2H), 7.27 – 7.00 (m, 5H), 5.15 (dd, J = 11.1, 5.4 Hz, 1H), 4.19 (td, J = 6.6, 1.5 Hz, 2H), 3.57 (qd, J = 14.3, 8.3 Hz, 2H), 1.67 – 1.53 (m, 2H), 1.31 (dd, J = 15.0, 7.5 Hz, 2H), 0.88 (t, J = 7.4 Hz, 3H). $^{13}C\{^1H\}$ NMR (100 MHz, $CDCl_3$) δ 168.91, 167.52, 136.85, 134.07, 131.63, 128.85, 128.55, 126.81, 123.43, 65.87, 53.46, 34.67, 30.46, 19.01, 13.61. HRMS (EI): m/z [M] calcd for $C_{21}H_{21}NO_4$ 351.1471, found: 351.1470.

Methyl (S)-2-(1,3-dioxoisoindolin-2-yl)-4-methylpentanoate[31]

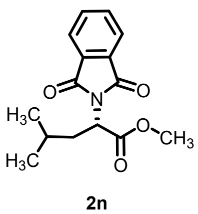

2n

As a foamy solid; ^{1}H NMR (400 MHz, $CDCl_3$) δ 7.87 (dd, J = 5.4, 3.1 Hz, 2H), 7.75 (dd, J = 5.5, 3.0 Hz, 2H), 4.96 (dd, J = 11.5, 4.4 Hz, 1H), 3.73 (s, 3H), 2.38 – 2.29 (m, 1H), 2.00 –

1.94 (m, 1H), 1.55 – 1.42 (m, 1H), 0.94 (dd, *J* = 10.8, 6.6 Hz, 6H). $^{13}C\{^1H\}$NMR (100 MHz, $CDCl_3$) δ 170.28, 167.75, 134.17, 131.87, 123.53, 52.72, 50.64, 37.29, 25.08, 23.16, 21.03. HRMS (EI): m/z [M] calcd for $C_{15}H_{17}NO_4$ 275.1158, found: 275.1156.

Butyl (S)-2-(1,3-dioxoisoindolin-2-yl)-4-methylpentanoate

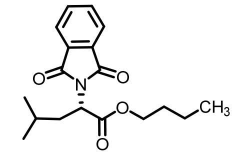

2nb

Colorless oil; ^1H NMR (400 MHz, $CDCl_3$) δ 7.87 (dd, *J* = 5.5, 3.0 Hz, 2H), 7.74 (dd, *J* = 5.5, 3.0 Hz, 2H), 4.94 (dd, *J* = 11.6, 4.4 Hz, 1H), 4.13 (td, *J* = 6.7, 1.5 Hz, 2H), 2.37 – 2.30 (m, 1H), 1.99 – 1.92 (m, 1H), 1.60 – 1.53 (m, 2H), 1.53 – 1.49 (m, 1H), 1.30 (dt, *J* = 15.0, 7.4 Hz, 2H), 0.94 (dd, *J* = 9.9, 6.6 Hz, 6H), 0.86 (t, *J* = 7.4 Hz, 3H). $^{13}C\{^1H\}$ NMR (100 MHz, $CDCl_3$) δ 169.85, 167.83, 134.14, 131.88, 123.48, 65.65, 50.84, 37.26, 30.46, 25.13, 23.19, 21.05, 18.99, 13.60. HRMS (EI): m/z [M] calcd for $C_{18}H_{23}NO_4$ 317.1627, found: 317.1624.

Methyl (S)-2-(1,3-dioxoisoindolin-2-yl)-3-methylbutanoate[31]

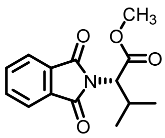

2nn

As a foamy solid; ^1H NMR (400 MHz, $CDCl_3$) δ 7.88 (dd, *J* = 5.4, 3.1 Hz, 2H), 7.75 (dd, *J* = 5.5, 3.1 Hz, 2H), 4.58 (d, *J* = 8.3 Hz, 1H), 3.71 (s, 3H), 2.87 – 2.67 (m, 1H), 1.15 (d, *J* = 6.7 Hz, 3H), 0.91 (d, *J* = 6.8 Hz, 3H). $^{13}C\{^1H\}$NMR (100 MHz, $CDCl_3$) δ 169.32, 167.77, 134.20, 131.74, 123.58, 57.56, 52.42, 28.57, 20.94, 19.39.

HRMS (EI): m/z [M] calcd for $C_{14}H_{15}NO_4$ 261.1001, found: 261.1000.

Butyl (S)-2-(1,3-dioxoisoindolin-2-yl)-3-methylbutanoate

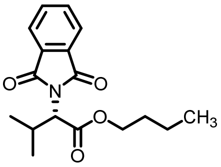

2nnb

Colorless oil; ^1H NMR (400 MHz, $CDCl_3$) δ 7.87 (dd, *J* = 5.5, 3.1 Hz, 2H), 7.75 (dd, *J* = 5.5, 3.0 Hz, 2H), 4.57 (d, *J* = 8.3 Hz, 1H), 4.13 (td, *J* = 6.7, 2.5 Hz, 2H), 2.90 – 2.66 (m, 1H), 1.56 (ddt, *J* = 13.8, 8.9, 7.1 Hz, 2H), 1.28 (dt, *J* = 15.0, 7.4 Hz, 2H), 1.16 (d, *J* = 6.7 Hz, 3H), 0.92 (d, *J* = 6.8 Hz, 3H), 0.85 (t, *J* = 7.4 Hz, 3H). $^{13}C\{^1H\}$ NMR (100 MHz, $CDCl_3$) δ 168.90, 167.83, 134.17, 131.75, 123.51, 65.33, 57.82, 30.41, 28.54, 21.00, 19.50, 19.02, 13.56. HRMS (EI): m/z [M] calcd for $C_{17}H_{21}NO_4$ 304.1471, found: 304.1470.

Dibutyl succinate and 8-aminoquinoline

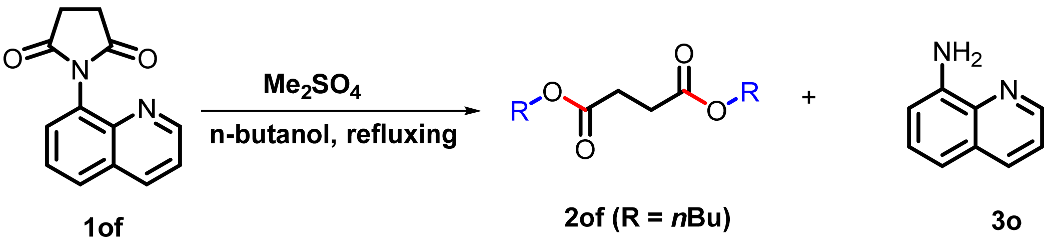

To a solution of amide **1of** (226 mg, 1mmol, 1.0 equiv) in n-butanol (5 mL) was

added dimethyl sulfate (252 mg, 2mmol, 2.0 equiv), and heated for 24h at 120°C in a sealed vial under an atmosphere of N_2 (monitored by TLC). The resulting mixture concentrated in vacuo to give residues. Then the residues were dissolved in ethyl acetate (30 V) and washed with sodium bicarbonate saturated solution, brine, dried over Na_2SO_4, filtered, and concentrated in vacuo. The crude product was purified by silica-gel column chromatography (5% EtOAc in *n*-hexane as the eluent) to give ester product **2of**[25] (207 mg, 90% yield); and silica-gel column chromatography (15% EtOAc in *n*-hexane as the eluent) to afford 8-aminoquinoline 3**o**[33] (126 mg, 88% yield).

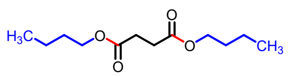

2of

Colorless oil; ^{1}H NMR (400 MHz, $CDCl_3$) δ 4.09 (t, J = 6.7 Hz, 4H), 2.62 (s, 4H), 1.61 (dt, J = 14.7, 6.8 Hz, 4H), 1.38 (dd, J = 15.0, 7.5 Hz, 4H), 0.93 (t, J = 7.4 Hz, 6H). ^{13}C{^{1}H}NMR (100 MHz, $CDCl_3$) δ 172.34, 64.54, 30.60, 29.17, 19.07, 13.65.
HRMS (EI): m/z [M] calcd for $C_{12}H_{22}O_4$ 230.1518, found: 230.1516.

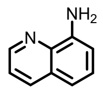

3o

Brown solid; m.p. 63–65 °C. ^{1}H NMR (400 MHz, $CDCl_3$) δ 8.79 (dd, J = 4.2, 1.6 Hz, 1H), 8.10 (dd, J = 8.3, 1.6 Hz, 1H), 7.45 – 7.33 (m, 2H), 7.18 (dd, J = 8.1, 0.8 Hz, 1H), 6.96 (dd, J = 7.5, 1.0 Hz, 1H), 4.98 (brs, 2H). ^{13}C{^{1}H} NMR (100 MHz, $CDCl_3$) δ 147.42, 143.96, 138.41, 136.04, 128.88, 127.41, 121.35, 116.05, 110.07. HRMS (ESI): m/z $[M + H]^+$ calcd for $C_9H_9N_2$ 145.0760, found: 145.0758.

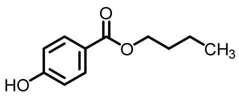

2pp

Colorless oil; ^{1}H NMR (400 MHz, $CDCl_3$) δ 7.95 (d, J = 8.7 Hz, 2H), 7.15 (s, 1H), 6.91 (d, J = 8.7 Hz, 2H), 4.31 (t, J = 6.6 Hz, 2H), 1.85 – 1.64 (m, 12H), 1.47 (dd, J = 15.0, 7.5 Hz, 2H), 0.97 (t, J = 7.4 Hz, 3H). ^{13}C{^{1}H} NMR (100 MHz, $CDCl_3$) δ 167.46, 160.64, 131.95, 122.26, 115.36, 65.02, 30.76, 19.28, 13.77. HRMS (ESI): m/z $[M - H]^-$ calcd for $C_{11}H_{13}O_3$ 193.0870, found: 193.0864.

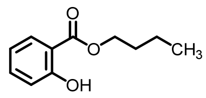

2qq

Colorless oil; ^{1}H NMR (400 MHz, $CDCl_3$) 10.85 (s, 1H), 7.85 (dd, J = 8.0, 1.7 Hz, 1H), 7.56 – 7.37 (m, 1H), 6.98 (dd, J = 8.4, 0.8 Hz, 1H), 6.94 – 6.76 (m, 1H), 4.36 (t, J = 6.6 Hz, 2H), 1.77 (dt, J = 14.5, 6.6 Hz, 2H), 1.61 – 1.31 (m, 2H), 0.99 (t, J = 7.4 Hz, 3H).^{13}C{^{1}H} NMR (100 MHz, $CDCl_3$) δ 170.27, 161.66, 135.58, 129.88, 119.10, 117.57, 112.66, 65.23, 30.61, 19.22, 13.74. HRMS (ESI): m/z $[M - H]^-$ calcd for $C_{11}H_{13}O_3$ 193.0870, found: 193.0867.

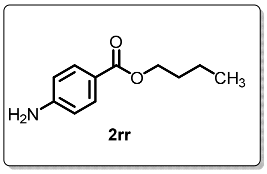

White solid; m.p. 56–58 °C. ^{1}H NMR (400 MHz, $CDCl_3$) δ 7.98 – 7.70 (m, 2H), 6.82 – 6.51 (m, 2H), 4.26 (t, J = 6.6 Hz, 2H), 4.19 – 3.90 (brs, 2H), 1.72 (dt, J = 14.5, 6.6 Hz, 2H), 1.47 (dt, J = 14.9, 7.4 Hz, 2H), 0.97 (t, J = 7.4 Hz, 3H). $^{13}C\{^{1}H\}$ NMR (100 MHz, $CDCl_3$) δ 166.79, 150.66, 131.56, 120.19, 113.85, 77.38, 77.06, 76.74, 64.24, 30.91, 19.33, 13.82. HRMS (ESI): m/z $[M + H]^+$ calcd for $C_{11}H_{15}NO_2$ 193.1103, found: 193.1098.**Dibutyl glutarate**

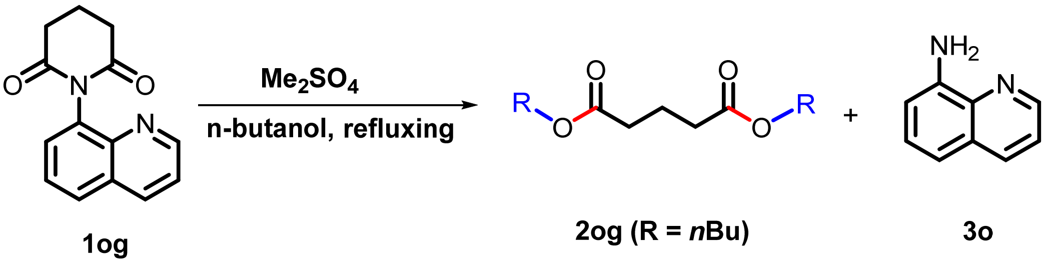

To a solution of amide **1og** (230 mg, 1mmol, 1.0 equiv) in n-butanol (5 mL) was added dimethyl sulfate (252 mg, 2mmol, 2.0 equiv), and heated for 24h at 120°C in a sealed vial under an atmosphere of N_2 (monitored by TLC). The resulting mixture concentrated in vacuo to give residues. Then the residues were dissolved in ethyl acetate (30 V) and washed with sodium bicarbonate saturated solution, brine, dried over Na_2SO_4, filtered, and concentrated in vacuo. The crude product was purified by silica-gel column chromatography (5% EtOAc in *n*-hexane as the eluent) to give ester product **2og**[32] (227 mg, 93% yield); and silica-gel column chromatography (15% EtOAc in *n*-hexane as the eluent) to afford 8-aminoquinoline (122 mg, 85% yield).

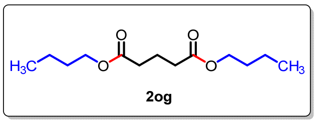

Colorless oil; ^{1}H NMR (400 MHz, $CDCl_3$) δ 4.08 (t, J = 6.7 Hz, 4H), 2.37 (t, J = 7.4 Hz, 4H), 2.01 – 1.88 (m, 2H), 1.61 (dt, J = 14.7, 6.8 Hz, 4H), 1.38 (dd, J = 15.1, 7.5 Hz, 4H), 0.94 (t, J = 7.4 Hz, 6H). $^{13}C\{^{1}H\}$NMR (100 MHz, $CDCl_3$) δ 172.94, 64.21, 33.28, 30.62, 20.19, 19.08, 13.62.
HRMS (EI): m/z [M] calcd for $C_{13}H_{24}O_4$ 244.1675, found: 244.1673.

Synthesis of n-butyl 2-ethoxybenzoate

Method A

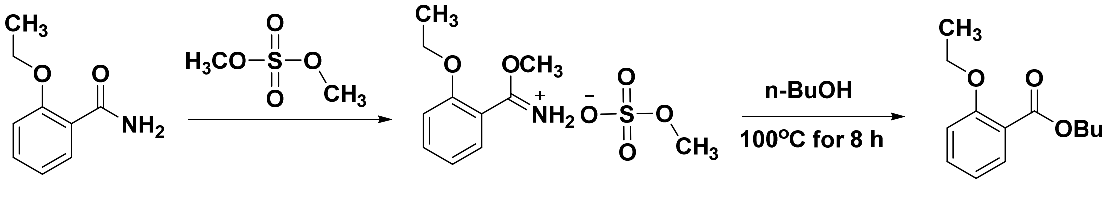

1z INT-1z 2z

Methyl 2- ethoxybenzimidate Methyl Hydrogen Sulfate (**INT-1z**).

To a 10 mL flask were charged **1z** (1.0g, 5.58 mmol) and dimethyl sulfate (2.11 g, 16.73 mmol). A clear solution formed when the temperature was raised to 70 °C, and the mixture was heated for an additional 8 h. The stirred reaction mixture was cooled to ambient temperature (15−20 °C), and methyl tert-butyl ether (4 mL) was added over 5 min. After an additional stirring for 1h, the resulting precipitate was collected by filtration, and the filter cake was washed with MTBE (0.50 mL × 3) and dried invacuum at 40 °C, giving **INT-1z** (1.40 g, 82%) as a white solid with slightly hygroscopic properties. ^{1}H NMR (500 MHz, chloroform-d): δ 12.08 (s, 1H), 10.24 (s, 1H), 8.04 (dd, J = 8.1, 1.5 Hz, 1H), 7.82 – 7.62 (m, 1H), 7.18 (dd, J = 13.7, 8.1 Hz, 2H), 4.49 (s, 3H), 4.40 (q, J = 7.0 Hz, 2H), 3.80 (s, 3H), 1.62 (t, J = 7.0 Hz, 3H). ^{13}C{^{1}H}NMR (125 MHz, $CDCl_3$) δ 169.99, 159.46, 138.21, 131.49, 121.81, 113.24, 111.65, 66.35, 60.01, 54.89, 14.48. HRMS (ESI): m/z [M – ($^-OSO_3Me$)]$^+$ calcd for $C_{10}H_{14}NO_2^+$ 180.1019, found: 180.1016. Elem. Anal. Calcd for $C_{11}H_{17}NO_6S$: C, 45.35; H, 5.88; N, 4.81; S, 11.01. Found: C, 45.43; H, 5.85; N, 4.87; S, 11.10.

A solution of **INT-1z** (291 mg, 1mmol, 1.0 equiv) in n-butanol (3 mL) was heated for 8 h at 100°C in a sealed vial under an atmosphere of N_2 (monitored by TLC). The resulting mixture concentrated in vacuo to give residues. Then the residues were dissolved in ethyl acetate (30 mL) and washed with sodium bicarbonate saturated solution, brine, dried over Na_2SO_4, filtered, and concentrated in vacuo. The crude product was purified by silica-gel column chromatography (5% EtOAc in *n*-hexane as the eluent) to give ester product **2z** (227 mg, 93% yield), as a colorless oil; ^{1}H NMR (400 MHz, $CDCl_3$) δ 7.77 (dd, J = 8.0, 1.8 Hz, 1H), 7.51 – 7.33 (m, 1H), 6.96 (t, J = 7.3 Hz, 2H), 4.31 (t, J = 6.6 Hz, 2H), 4.11 (q, J = 7.0 Hz, 2H), 1.74 (dt, J = 14.5, 6.6 Hz, 2H), 1.56 – 1.38 (m, 5H), 0.97 (t, J = 7.4 Hz, 3H). ^{13}C{^{1}H}NMR (100 MHz, $CDCl_3$) δ 166.76, 158.42, 133.12, 131.51, 121.01, 120.06, 113.25, 64.67, 64.52, 30.80, 19.28, 14.75, 13.73.

HRMS (EI): m/z [M] calcd for $C_{13}H_{18}O_3$ 222.1256, found: 222.1253.

Method B

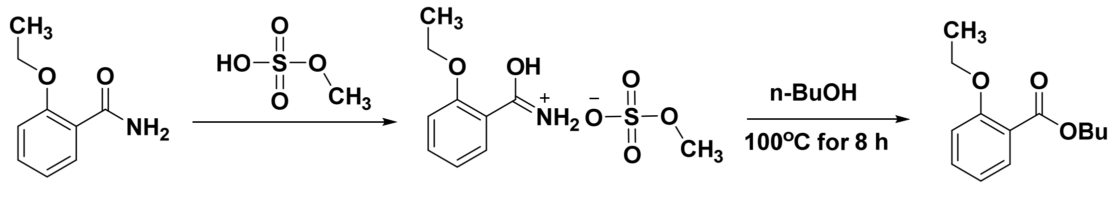

1z **INT-3z** **2z**

2- ethoxybenzimidate Methyl Hydrogen Sulfate (**INT-3z**).

To a solution of amide **1z** (165 mg, 1 mmol) 5 mL of methanol was added methyl hydrogen sulfate (336 mg, 3 mmol), and then heating for 15 min. The resultant solutions were air cooled to room temperature and allowed in open vial for slow evaporation. Solid was obtained within two days.

^{1}H NMR (400 MHz, CD_3OD) δ 7.96 (dd, J = 7.8, 1.8 Hz, 1H), 7.56 – 7.41 (m, 1H), 7.13 (d, J = 8.2 Hz, 1H), 7.09 – 6.96 (m, 1H), 4.24 (q, J = 7.0 Hz, 2H), 3.68 (s, 3H), 1.49 (t, J = 7.0 Hz, 3H). ^{13}C{^{1}H}NMR (100 MHz, CD_3OD) δ 168.98, 157.42, 133.32, 131.06, 120.74, 120.43, 112.55, 64.57, 53.72, 13.58. HRMS (ESI): m/z [M – ($^-OSO_3Me$)]$^+$ calcd for $C_9H_{12}NO_2^+$ 166.0863, found: 166.0855. Elem. Anal. Calcd for $C_{10}H_{15}NO_6S$: C, 43.32; H, 5.45; N, 5.05; S, 11.56. Found: C, 43.25; H, 5.48; N, 5.09; S, 11.64.

n-Butyl 2-ethoxybenzoate.

A solution of **INT-3z** (277 mg, 1mmol, 1.0 equiv) in n-butanol (3 mL) was heated for 8 h at 100°C in a sealed vial under an atmosphere of N_2 (monitored by TLC). The resulting mixture concentrated in vacuo to give residues. Then the residues were dissolved in ethyl acetate (30 mL) and washed with sodium bicarbonate saturated solution, brine, dried over Na_2SO_4, filtered, and concentrated in vacuo. The crude product was purified by silica-gel column chromatography (5% EtOAc in *n*-hexane as the eluent) to give ester product **2z** (218 mg, 88% yield), as a colorless oil;

General procedure for cleavage of acyl protective group on amines with dimethyl sulphate

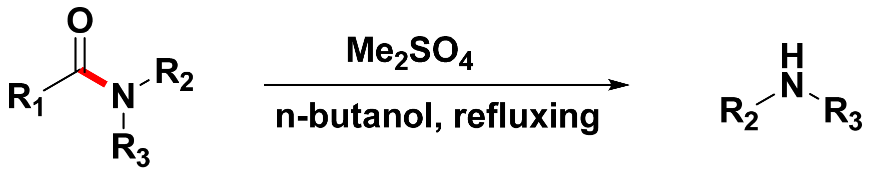

1 **3**

To a solution of amide **1** (1.0 equiv) in n-butanol (0.2 M) was added dimethyl sulfate (1.0 equiv), and heated for 8h at 120°C in a sealed vial under an atmosphere of N_2 (monitored by TLC). The resulting mixture concentrated in vacuo to give residues. Then the residues were dissolved in ethyl acetate (30 V) and washed with sodium bicarbonate saturated solution, brine, dried over Na_2SO_4, filtered, and concentrated in

vacuo. The crude product was purified by silica-gel column chromatography to give amine product **3.**

2-(3,4-Dimethoxyphenyl)ethan-1-amine[33]

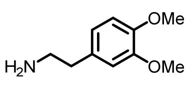

3q

Brown solid; m.p. 153–155 °C. ^{1}H NMR (400 MHz, $CDCl_3$) δ 6.77 (d, J = 7.8 Hz, 1H), 6.72 – 6.67 (m, 2H), 3.83 (s, 3H), 3.81 (s, 3H), 2.89 (d, J = 6.8 Hz, 2H), 2.65 (s, 2H), 1.18 (s, 2H). ^{13}C{^{1}H} NMR (100 MHz, $CDCl_3$) δ 148.92, 147.45, 132.46, 120.71, 112.08, 111.34, 55.91, 55.81, 43.65, 39.63. HRMS (ESI): m/z [M + H]$^+$ calcd for $C_{10}H_{16}NO_2$ 182.1176, found: 182.1175.

Isoindoline[34]

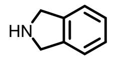

3r

Colorless oil; ^{1}H NMR (400 MHz, CD_3OD) δ 7.59 – 7.22 (m, 4H), 4.63 (s, 4H). ^{13}C{^{1}H} NMR (100 MHz, CD_3OD) δ 134.16, 128.52, 122.65, 50.54. HRMS (ESI): m/z [M + H]$^+$ calcd for $C_8H_{10}N$ 120.0808, found: 182.1175.

Dibenzylamine[35]

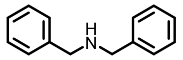

3s

Colorless oil; ^{1}H NMR (400 MHz, $CDCl_3$) δ 7.38 – 7.21 (m, 10H), 3.81 (s, 4H), 1.74 (s, 1H). ^{13}C{^{1}H} NMR (100 MHz, $CDCl_3$) δ 140.28, 128.43, 128.19, 126.99, 53.16. HRMS (ESI): m/z [M + H]$^+$ calcd for $C_{14}H_{16}N$ 198.1277, found: 192.1275.

N-Methylaniline[36]

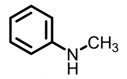

3t

Colorless oil; ^{1}H NMR (400 MHz, $CDCl_3$) δ 7.23 – 7.14 (m, 2H), 6.71 (t, J = 7.3 Hz, 1H), 6.61 (dd, J = 8.5, 0.8 Hz, 2H), 2.83 (s, 3H). ^{13}C{^{1}H} NMR (100 MHz, $CDCl_3$) δ 149.34, 129.23, 117.29, 112.45, 30.77. HRMS (ESI): m/z [M + H]$^+$ calcd for $C_7H_{10}N$ 108.0808, found: 108.0805.

3-Azaspiro[5.5]undecane

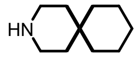

3u

As a foamy solid; ^{1}H NMR (400 MHz, CD_3OD) δ 3.21 – 3.12 (m, 4H), 1.75 – 1.66 (m, 4H), 1.57 – 1. 44 (m, 10H). ^{13}C{^{1}H} NMR (100 MHz, CD_3OD) δ 39.68, 35.33, 32.39, 29.98, 26.05, 20.82. HRMS (ESI): HRMS (EI): m/z [M] calcd for $C_{10}H_{19}N$ 153.1517, found: 153.1514.

1-Methylpiperazine[37]

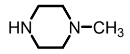

3v

Colorless oil; ^{1}H NMR (400 MHz, $CDCl_3$) δ 2.60 (dd, J = 8.6, 4.3 Hz, 4H), 2.08 (s, 3H), 2.05 – 1.86 (m, 4H), 1.71 (s, 1H). $^{13}C\{^1H\}$ NMR (100 MHz, $CDCl_3$) δ 56.10, 46.44, 45.75. HRMS (ESI): m/z $[M + H]^+$ calcd for $C_5H_{13}N_2$ 101.1073, found: 101.1070.

Butyl L-phenylalaninate[38]

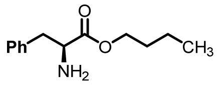

3w

Colorless oil; ^{1}H NMR (400 MHz, CD_3OD) δ 7.45 – 7.21 (m, 5H), 4.29 (t, J = 7.0 Hz, 1H), 4.17 (t, J = 6.5 Hz, 2H), 3.26 – 3.13 (m, 2H), 1.68 – 1.43 (m, 2H), 1.29 (dd, J = 15.1, 7.5 Hz, 2H), 0.90 (t, J = 7.4 Hz, 3H). $^{13}C\{^1H\}$ NMR (100 MHz, CD_3OD) δ 170.33, 135.40, 130.48, 130.19, 128.99, 67.46, 55.28, 37.59, 31.46, 19.98, 13.99. HRMS (ESI): m/z $[M + H]^+$ calcd for $C_{13}H_{20}NO_2$ 222.1489, found: 222.1487.

Butyl L-leucinate

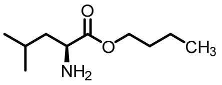

3x

Colorless oil; ^{1}H NMR (400 MHz, CD_3OD) δ 4.26 (t, J = 6.5 Hz, 2H), 4.01 (t, J = 6.8 Hz, 1H), 1.87 – 1.61 (m, 5H), 1.43 (dd, J = 15.1, 7.5 Hz, 2H), 1.08 – 0.86 (m, 9H). $^{13}C\{^1H\}$ NMR (100 MHz, $CDCl_3$) δ 171.21, 67.41, 52.59, 40.80, 31.59, 25.70, 22.53, 22.51, 20.09, 13.99. HRMS (ESI): m/z $[M + H]^+$ calcd for $C_{10}H_{22}NO_2$ 188.1645, found: 188.1643.

Butyl L-valinate

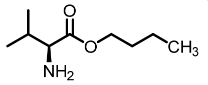

3y

Colorless oil; ^{1}H NMR (400 MHz, CD_3OD) δ 4.29 (td, J = 6.6, 2.6 Hz, 2H), 3.94 (d, J = 4.6 Hz, 1H), 2.39 – 2.23 (m, 1H), 1.71 (dt, J = 8.7, 6.7 Hz, 2H), 1.56 – 1.35 (m, 2H), 1.10 (dd, J = 7.0, 3.5 Hz, 6H), 0.99 (t, J = 7.4 Hz, 3H). $^{13}C\{^1H\}$ NMR (100 MHz, CD_3OD) δ 170.10, 67.28, 59.47, 31.66, 31.02, 20.12, 18.36, 18.23, 13.91. HRMS (ESI): m/z $[M + H]^+$ calcd for $C_9H_{20}NO_2$ 174.1489, found: 188. 174.1486.

Butyl D-phenylalaninate

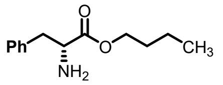

3wD

Colorless oil; ^{1}H NMR (400 MHz, CD_3OD) δ 7.49 – 7.21 (m, 1H), 4.31 (t, J = 7.0 Hz, 1H), 4.19 (t, J = 6.5 Hz, 1H), 3.29 – 3.13 (m, 1H), 1.66 – 1.49 (m, 1H), 1.30 (dd, J = 15.1, 7.5 Hz, 1H), 0.92 (t, J = 7.4 Hz, 1H). $^{13}C\{^1H\}$ NMR (100 MHz, CD_3OD) δ 171.17, 67.36, 52.54, 40.76, 31.54, 25.65, 22.48, 22.46, 20.04, 13.94.HRMS (ESI): m/z $[M + H]^+$ calcd for $C_{13}H_{20}NO_2$ 222.1489, found: 222.1488.

Butyl D-leucinate

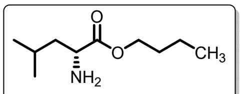

3xD

Colorless oil; ^{1}H NMR (400 MHz, CD_3OD) δ 4.26 (t, J = 6.5 Hz, 2H), 4.01 (t, J = 6.8 Hz, 1H), 1.87 – 1.61(m, 5H), 1.49 – 1.39 (m, 2H), 1.16 – 0.79 (m, 9H). $^{13}C\{^1H\}$ NMR (100 MHz, $CDCl_3$) δ 171.21, 67.41, 52.59, 40.80, 31.59, 25.70, 22.53, 22.51, 20.09,

13.99. HRMS (ESI): m/z $[M + H]^+$ calcd for $C_{10}H_{22}NO_2$ 188.1645, found: 188.1642.

Butyl D-valinate

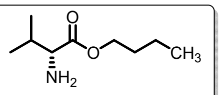

3yD

Colorless oil; ^{1}H NMR (400 MHz, CD_3OD) δ 4.27 (td, J = 6.6, 2.6 Hz, 2H), 3.92 (d, J = 4.6 Hz, 1H), 2.43 – 2.15 (m, 1H), 1.68 (dt, J = 8.7, 6.7 Hz, 1H), 1.53 – 1.30 (m, 2H), 1.07 (dd, J = 7.0, 3.5 Hz, 6H), 0.97 (t, J = 7.4 Hz, 3H). ^{13}C{^{1}H} NMR (100 MHz, CD_3OD) δ 170.13, 67.31, 59.50, 31.69, 31.05, 20.15, 18.39, 18.26, 13.95. HRMS (ESI): m/z $[M + H]^+$ calcd for $C_9H_{20}NO_2$ 174.1489, found: 188. 174.1488.

Computational details

Density functional theory (DFT) calculations were performed with Gaussian16[39] package. Geometry optimizations were carried out using the M06-2X functional[40] and 6-31G(d) basis set[41] in solution (1-butanol, ε=17.332) by the continuum method PCM[42]. Frequency analysis was performed at the same level under standard conditions to either a minimum (i.e., no imaginary frequency) or a transition state (i.e., only one imaginary frequency) and to obtain thermodynamic energy corrections. Intrinsic reaction coordinate (IRC) calculations[43] were conducted to verify that all transition state structures connected the corresponding reactants and products. Single-point calculations with the M06-2X/6-311++G(d,p) level of theory[44] and SMD[45] solvent model were performed using the geometries obtained at optimization step. The free energies presented in this work represent M06-2X/6-311++G(d,p) calculated single-point energies with M06-2X/6-31G(d) calculated thermodynamic corrections, denoted as ΔG_{sol}(M06-2X SMD/6-311++G(d,p) // M06-2X PCM/6-31G(d)) or simply as ΔG_{sol} for the sake of clarity.

DFT Calculations

Energy values for the reported species and imaginary frequencies for the transition states

Optimized cartesian coordinates of the calculated structures

1a+Me_2SO_4

C	-4.64759000	-1.42448500	0.84004300
C	-3.38086800	-0.86463000	0.96432200
C	-2.96173300	0.13002100	0.07804900
C	-3.81195500	0.54308700	-0.95127300
C	-5.07703700	-0.02406000	-1.07950000
C	-5.49809600	-1.00288200	-0.18147200
H	-4.97192200	-2.19029100	1.53735700
H	-2.70111100	-1.18539800	1.74684600
H	-3.48454900	1.28305000	-1.67600300
H	-5.73037900	0.29371700	-1.88558200
H	-6.48574900	-1.44183000	-0.28194200
C	-1.58450100	0.69330600	0.26997600
O	-0.70646100	0.05139000	0.84995200
N	-1.35739900	1.92839100	-0.22296100
H	-2.14014300	2.49080400	-0.52315800

C	-0.06681400	2.56979300	-0.04882300
H	-0.06865800	3.51081400	-0.59797500
H	0.13628700	2.77012800	1.00908900
H	0.72836600	1.92827700	-0.43733300
S	3.56979800	-0.27511500	-0.35458900
O	4.96204100	-0.53656700	-0.60480200
O	2.93033600	0.91926500	-0.86242400
O	2.80446900	-1.56421400	-0.87348400
O	3.33463500	-0.36367800	1.21489100
C	1.37287000	-1.58910200	-0.65802100
C	2.43749700	0.59897100	1.83293700
H	1.02136700	-2.45665300	-1.21182400
H	1.16121800	-1.69886900	0.40635700
H	0.91935400	-0.67427300	-1.04108400
H	2.48505000	0.35822900	2.89305000
H	2.80403100	1.60969200	1.65339600
H	1.42104800	0.47602600	1.45247000

TS-1

C	4.87305300	0.95337700	-1.08860700
C	5.47029800	1.12494200	0.15906600
C	4.82705600	0.67226400	1.30964100
C	3.59464900	0.03455300	1.21494600
C	2.99865000	-0.14698200	-0.03723000
C	3.63551500	0.32773900	-1.18694000
C	1.67850000	-0.81941500	-0.20734900
O	1.01481400	-0.60450400	-1.25636800
O	-2.70746200	0.35455200	-1.24626600
S	-3.54765100	0.01493800	-0.04080900
O	-4.79503700	-0.64516100	-0.38423400
O	-2.73742500	-0.59645400	1.01554100
O	-4.03404800	1.45627900	0.51358900
C	-2.97766100	2.33660000	0.91625300
H	5.36987500	1.31004600	-1.98471600
H	6.43361300	1.61873400	0.23647200
H	5.28069600	0.82154900	2.28364200
H	3.09519600	-0.27961500	2.12683100
H	3.15322300	0.19419500	-2.14897700
H	-3.46490200	3.25002900	1.25359400
H	-2.40562800	1.89110900	1.73354600
H	-2.32379000	2.55293700	0.06713200
C	-0.83430700	-0.17932200	-1.19868900
H	-0.80260200	0.02753200	-0.13995900
H	-1.11849300	-1.15676800	-1.54941700
H	-0.64094900	0.60557200	-1.91104700
C	0.08996400	-2.51012700	0.62369000
H	0.00212700	-2.84441900	-0.41229900
H	-0.82670600	-1.99624900	0.92388700
H	0.24478900	-3.37698800	1.26468000
N	1.26122900	-1.64660000	0.74465500
H	1.87632200	-1.80993300	1.53022500

INT-1

C	5.20737600	-1.29638400	-0.01886100
C	5.97023600	-0.13113000	0.04998600
C	5.35127200	1.11844200	0.06900600
C	3.96583500	1.20737500	0.02878500
C	3.20019400	0.03630500	-0.02776300
C	3.82143600	-1.21742800	-0.06370300
C	1.73080000	0.09635700	-0.05709500
O	1.14684800	-0.89414900	-0.66325900
N	1.09221300	1.09628900	0.49302500
C	-0.30381000	1.51228500	0.28137000
C	-0.19374400	-1.33838400	-0.31356000
H	5.69256100	-2.26606300	-0.03630100
H	7.05294700	-0.19613400	0.08146800
H	5.94780400	2.02325100	0.10320900
H	3.49202400	2.18419800	0.00255900
H	3.21609400	-2.11557500	-0.11411400
H	1.66765100	1.72113800	1.04756300
H	-0.36412500	2.55726500	0.57899300
H	-0.55258700	1.42624400	-0.77602000
H	-1.00186000	0.91394300	0.86931700
H	-0.21218500	-2.38371900	-0.61280900
H	-0.34585700	-1.24137100	0.76028600
H	-0.94557200	-0.76720400	-0.85512700
S	-3.75814400	-0.26276500	0.46263900
O	-4.43977800	-1.42535900	-0.11872000
O	-4.66133700	0.76437500	0.99526600
O	-2.61884200	-0.59017800	1.33202400
O	-2.98180300	0.45925200	-0.80263500
C	-3.84546600	0.87833000	-1.85788900
H	-3.21129900	1.36449800	-2.59945700
H	-4.34813000	0.01610600	-2.30423000
H	-4.58812400	1.58751300	-1.48181500

1-BuOH

C	1.31007700	0.47287500	0.00000900

H	1.33472600	1.12358300	0.88680800
H	1.33473300	1.12358100	-0.88679100
O	2.40924400	-0.42520900	0.00001400
C	0.03046100	-0.34321800	0.00000500
H	0.03021600	-0.99697400	0.88142500
H	0.03023800	-0.99699600	-0.88139900
C	-1.22123700	0.53163000	-0.00002200
H	-1.20517100	1.18877800	-0.87878900
H	-1.20518300	1.18881700	0.87871600
C	-2.50530700	-0.29410600	-0.00001200
H	-2.55327700	-0.93832800	0.88417600
H	-3.39279900	0.34496600	-0.00003800
H	-2.55326000	-0.93837600	-0.88416500
H	3.22186600	0.09954100	0.00006200

TS-2

C	5.60132700	0.62261900	1.07237300
C	6.44650700	0.10273600	0.09256300
C	5.92671900	-0.67320200	-0.94215500
C	4.55952800	-0.92115700	-1.00722100
C	3.70990900	-0.38982900	-0.03212600
C	4.23558100	0.37082400	1.01589800
C	2.24028900	-0.62418300	-0.05422700
O	1.61159200	-0.62582400	1.03772400
N	1.65477900	-0.82095500	-1.23085400
C	0.29422100	-1.32036700	-1.41537400
C	-0.07516100	0.20158200	1.22862800
H	6.00748800	1.22221200	1.88019700
H	7.51345800	0.29577300	0.13982000
H	6.58641400	-1.09371600	-1.69361600
H	4.16842400	-1.55624500	-1.79678900
H	3.56417500	0.76175900	1.77294100
H	2.21848300	-0.68470100	-2.06007100
H	0.25791900	-1.87805800	-2.35003700
H	0.03795900	-1.99291500	-0.59542800
H	-0.44148800	-0.51392000	-1.46007000
H	0.23142500	0.50275800	2.21784600
H	0.11518500	0.85610900	0.38987300
H	-0.60657400	-0.72720000	1.08524800
S	-3.13137500	-1.68338500	-0.52788000
O	-4.50943400	-2.08091000	-0.24602500
O	-2.50045200	-2.30506500	-1.68772200
O	-2.92257600	-0.20956300	-0.46798700
O	-2.22936500	-2.26933800	0.71404100
C	-2.80212100	-2.10975200	2.01682800
H	-2.06072000	-2.49651900	2.71586400
H	-2.99182400	-1.05365800	2.23421100
H	-3.73003200	-2.67865300	2.09428200
C	-1.79415300	2.47569300	1.36332800
H	-2.64834200	2.87510700	1.92134200
H	-0.87956000	2.84832300	1.83733600
O	-1.78249600	1.05146300	1.53105100
C	-1.86657100	2.89558500	-0.09667300
H	-2.76731000	2.46295000	-0.54996100
H	-1.00929700	2.48642600	-0.64770400
C	-1.88884600	4.41651900	-0.24385100
H	-0.98770600	4.83925400	0.21757500
H	-2.74346000	4.82048800	0.31276200
C	-1.97343800	4.85153300	-1.70456800
H	-2.88114100	4.45968600	-2.17476000
H	-1.98957700	5.94113800	-1.79446000
H	-1.11506000	4.47822100	-2.27263800
H	-2.35119300	0.62851900	0.82910600

$BuOCH_3$

C	-0.71362300	0.37855500	-0.00000700
H	-0.77800600	1.03074700	-0.88722700
H	-0.77804300	1.03070200	0.88724200
O	-1.78142900	-0.54375400	-0.00005300
C	0.59764900	-0.38508300	0.00000200
H	0.62587700	-1.03751900	-0.88163200
H	0.62583300	-1.03757100	0.88159900
C	1.81049000	0.54309700	0.00006000
H	1.76647700	1.19871600	0.87894200
H	1.76651500	1.19877600	-0.87878000
C	3.12816700	-0.22786500	0.00006200
H	3.20340500	-0.86930500	-0.88426400
H	3.98754400	0.44856000	0.00010600
H	3.20336400	-0.86936900	0.88434500
C	-3.03201200	0.10149700	-0.00006600
H	-3.15631100	0.73322700	-0.89134100
H	-3.80289500	-0.67130800	-0.00011000
H	-3.15635700	0.73317200	0.89124200

INT-2

C	3.56922200	-1.70569200	-0.07896000
C	4.57586000	-0.95733700	0.53086400
C	4.39259500	0.40385600	0.76810700

C	3.19815700	1.01853400	0.40520500
C	2.18802600	0.26699600	-0.20120500
C	2.37808100	-1.09407200	-0.45257100
C	0.89158600	0.86818500	-0.61104600
O	0.28167700	0.41368100	-1.61031300
N	0.42159900	1.88527200	0.10837500
C	-0.77280100	2.64071200	-0.23993300
H	3.71390900	-2.76526500	-0.26234100
H	5.50743300	-1.43502500	0.81738200
H	5.18190000	0.98884200	1.22828900
H	3.07249900	2.08576800	0.56486800
H	1.57886800	-1.65663000	-0.92308400
H	0.90114300	2.12327800	0.96681600
H	-0.93582900	2.56118000	-1.31513100
H	-1.64721900	2.25686700	0.29102700
H	-0.61629900	3.68641800	0.02533200
S	-2.19563800	-0.92249700	0.06560200
O	-3.23970500	-1.91240800	-0.07969900
O	-0.88249300	-1.30441500	0.53875300
O	-2.08914400	-0.11819200	-1.26165400
O	-2.68880300	0.18658900	1.11257900
C	-4.01593200	0.70651900	0.89307000
H	-4.16149300	1.45409600	1.67012600
H	-4.07520200	1.16911100	-0.09491800
H	-4.74880200	-0.09542700	0.98862500
H	-1.07290400	0.18160100	-1.44374900

INT-3

C	2.29785600	-2.14621700	0.21959100
C	3.68983600	-2.10003600	0.15636700
C	4.34980100	-0.87662700	0.04508200
C	3.61934000	0.30353600	-0.00532900
C	2.22115300	0.25166700	0.04457000
C	1.55419000	-0.97367500	0.16359900
C	1.46674300	1.50906800	-0.02446100
O	2.13161200	2.58524400	0.32973700
N	0.22877400	1.56731900	-0.40753000
C	-0.56265800	2.79520300	-0.44759200
H	1.78650900	-3.09730400	0.32120500
H	4.26327100	-3.02040500	0.19992100
H	5.43274700	-0.84218000	-0.00220600
H	4.12218100	1.26008900	-0.09425900
H	0.47178600	-1.02565000	0.24288800
H	-0.25170800	0.70861500	-0.75318700
H	-1.57306500	2.51163300	-0.73374100
H	-0.59699500	3.26060100	0.54099900
H	-0.15646300	3.49255600	-1.18566300
S	-2.34350300	-0.83504600	-0.31594300
O	-3.60084100	-1.36442600	-0.83954300
O	-1.72168300	-1.63759200	0.74353900
O	-1.38975000	-0.39380600	-1.36199700
O	-2.69591100	0.60954500	0.37179700
C	-3.57570200	0.53051700	1.49678500
H	-3.73915100	1.55608700	1.82578200
H	-4.52580800	0.07743100	1.20163400
H	-3.11423100	-0.05253700	2.29756000
H	1.62478600	3.40822600	0.20692900

TS-3

C	-3.21542000	-1.61731700	-0.68781700
C	-2.22580900	-0.83277900	-0.10564600
C	-2.38299300	0.55339000	-0.03515700
C	-3.54523500	1.14424200	-0.52668200
C	-4.53460600	0.35508600	-1.11138100
C	-4.37002200	-1.02404000	-1.19748800
C	-1.31717000	1.41015500	0.62022400
N	-1.21711200	1.16276700	1.99290000
O	0.00883000	1.00132300	-0.10366700
O	-1.45126100	2.75710800	0.41094000
C	0.97206000	2.03714200	-0.43534800
C	2.30802800	1.38388300	-0.73093800
C	3.34070800	2.41651800	-1.18226100
C	4.69228000	1.76883900	-1.47220700
H	-3.08149300	-2.69315800	-0.74287400
H	-1.33236100	-1.30811400	0.28937200
H	-3.69459600	2.21567800	-0.43937600
H	-5.43617300	0.82254300	-1.49353500
H	-5.14087600	-1.63684500	-1.65348700
H	-1.28848500	0.17021400	2.19377200
H	0.49755000	0.07849500	0.45964000
H	0.59204000	2.56623600	-1.31329500
H	1.04658400	2.74295000	0.39512200
H	2.67087100	0.86299100	0.16253400
H	2.17976800	0.62110600	-1.50875500
H	2.97496000	2.93124100	-2.07912500
H	3.45478400	3.18206500	-0.40505900
H	5.42855600	2.51190400	-1.79028400
H	5.08085500	1.26542300	-0.58111800

H	4.60176700	1.01975000	-2.26546100
C	-0.11945500	1.80049100	2.71687000
H	-0.25834800	1.61300800	3.78220700
H	0.86754300	1.41979000	2.42238300
H	-0.15813800	2.87820000	2.54941400
S	1.56565600	-2.08702900	0.60616700
O	2.74570100	-1.72411300	-0.16123600
O	1.68799900	-3.08698200	1.64515800
O	0.85492400	-0.85128100	1.16745800
O	0.49235900	-2.71070900	-0.42722300
C	0.42998900	-2.08992100	-1.72337300
H	-0.38193900	-2.59501400	-2.24442100
H	0.19718900	-1.02448200	-1.63086400
H	1.37486400	-2.22899500	-2.24994300
H	-1.62816100	2.92120300	-0.53003200

INT-4

C	-2.89874500	-1.53275600	-1.80737100
C	-2.00706400	-0.72600900	-1.09981100
C	-2.48523800	0.12924800	-0.10740200
C	-3.85639600	0.18296200	0.16288700
C	-4.74094100	-0.62098200	-0.54712500
C	-4.26214200	-1.48364800	-1.53360800
C	-1.57327900	1.06427500	0.67629200
N	-1.85396800	0.96636800	2.07119200
O	-0.20646900	0.67180800	0.34852200
O	-1.75360600	2.39596200	0.31334800
C	0.86167800	1.63695500	0.34233300
C	1.39955300	1.79222300	-1.06828100
C	2.67471400	2.63170900	-1.09783200
C	3.20951600	2.81706700	-2.51545200
H	-2.52065000	-2.20133100	-2.57442400
H	-0.94590500	-0.76927900	-1.32033500
H	-4.21657000	0.85747400	0.93308700
H	-5.80381400	-0.57523700	-0.33164600
H	-4.95191200	-2.11359100	-2.08666200
H	-1.91836400	-0.01412700	2.33059400
H	0.28112500	-0.66139300	0.81766400
H	0.50162900	2.58677300	0.74078100
H	1.63834800	1.25135000	1.01370000
H	1.59956200	0.79499300	-1.47879200
H	0.62716700	2.25312700	-1.69860500
H	2.48206200	3.61159100	-0.64280200
H	3.43608200	2.14230300	-0.47656600
H	4.12730700	3.41154800	-2.52125400
H	3.43063300	1.84857100	-2.97579400
H	2.47314400	3.32625500	-3.14583100
C	-0.94358400	1.68301500	2.95975500
H	-1.31638400	1.59310500	3.98161800
H	0.08743600	1.30294900	2.93216600
H	-0.93790500	2.74141300	2.69096800
S	1.78448600	-2.00588100	0.05275800
O	2.29376900	-3.28380200	0.48920600
O	1.26483600	-1.83232100	-1.28420600
O	0.74087800	-1.53699300	1.12324100
O	2.93329200	-0.90059500	0.17747100
C	3.67732300	-0.90090000	1.41525500
H	4.36858900	-0.06514900	1.32982800
H	2.99804400	-0.74874000	2.25716400
H	4.21862000	-1.84153100	1.52018200
H	-1.74621000	2.44702500	-0.65601500

INT-5

C	4.90766300	1.13547500	0.15534000
C	3.51600600	1.08105000	0.17697600
C	2.86139100	-0.06609600	-0.27144200
C	3.59710100	-1.15596000	-0.73954700
C	4.98754000	-1.09682700	-0.75760800
C	5.64387900	0.04825500	-0.30987700
C	1.34939800	-0.16686900	-0.22222200
N	0.97603700	-0.94873700	1.08313900
O	0.81949600	1.09533500	-0.11023700
O	0.90085500	-0.92803200	-1.26022900
C	-0.60728100	1.22047400	-0.17733700
C	-0.94999800	2.69046800	-0.05072700
C	-2.45731700	2.92041900	-0.15335500
C	-2.82553900	4.39260900	0.00987400
H	5.41535800	2.03024000	0.50052900
H	2.93653400	1.92674900	0.53072200
H	3.07997700	-2.03840300	-1.10335500
H	5.55742500	-1.94321000	-1.12681800
H	6.72815900	0.09376800	-0.32635600
H	-0.03450600	-1.23270400	1.06430600
H	-0.96974900	0.81595600	-1.12939000
H	-1.07334600	0.65202500	0.63730800
H	-0.57689700	3.06395900	0.91108800
H	-0.42871700	3.24715700	-0.83864000
H	-2.81066400	2.55539100	-1.12651600

H	-2.96946800	2.31839000	0.60755900
H	-3.90523000	4.54435900	-0.07330500
H	-2.50643800	4.76709100	0.98806200
H	-2.33820700	5.00354700	-0.75705700
C	1.22723500	-0.22069200	2.35280900
H	0.92832600	-0.86328400	3.17951000
H	2.28707300	0.02132300	2.42217000
H	0.63307200	0.69171800	2.34901000
H	1.51345400	-1.82160400	1.06361400
S	-2.49524400	-1.76399400	0.12853100
O	-3.45997100	-2.85536900	0.13612400
O	-1.68691500	-1.62806800	1.35926000
O	-1.66925400	-1.70123100	-1.10044600
O	-3.34142500	-0.37788900	0.15109700
C	-4.18622000	-0.17196400	-0.98831500
H	-4.72771600	0.75257600	-0.79238400
H	-3.58009300	-0.06996300	-1.89227000
H	-4.88785900	-1.00319900	-1.09329300
H	-0.06429100	-1.14682700	-1.18492400

TS-4

C	4.92533500	1.05179300	-0.16928000
C	3.53651200	1.07607300	-0.23546300
C	2.83864600	-0.11082000	-0.47632000
C	3.52362500	-1.31624900	-0.65095900
C	4.91142700	-1.33200200	-0.58276400
C	5.61137600	-0.14958100	-0.34225400
C	1.36486100	-0.10895300	-0.54400100
N	1.10167000	-0.49840600	1.85872900
O	0.80733100	1.06320200	-0.45369400
O	0.76682700	-1.16303300	-0.89639500
C	-0.63373800	1.15826700	-0.39374500
C	-0.99072500	2.62209300	-0.26092500
C	-2.50644300	2.81835500	-0.23754400
C	-2.88725700	4.28670500	-0.06921100
H	5.47229600	1.97008300	0.01592200
H	2.99302500	2.00555300	-0.10578700
H	2.96424000	-2.22694700	-0.83506600
H	5.44793600	-2.26523800	-0.71699000
H	6.69536300	-0.16477900	-0.28977400
H	0.18908700	-0.91753300	2.03520200
H	-1.05440200	0.72210400	-1.30494100
H	-0.96366700	0.57507100	0.47280800
H	-0.54455800	3.02127900	0.65793200
H	-0.55430300	3.17469900	-1.10126400
H	-2.93213600	2.42991700	-1.17163900
H	-2.93915300	2.22141500	0.57437100
H	-3.97285800	4.41613500	-0.06302100
H	-2.49416200	4.68511900	0.87171400
H	-2.47906500	4.89132900	-0.88565700
C	1.27269900	0.73938900	2.61314900
H	1.23503700	0.61501400	3.70283900
H	2.23669000	1.18770300	2.35161900
H	0.48893200	1.44648500	2.32440000
H	1.80253700	-1.18234900	2.13637400
S	-2.45160000	-1.80702700	0.18424600
O	-3.38331800	-2.91731600	0.08026100
O	-1.69054700	-1.66885800	1.41958000
O	-1.57390600	-1.71470600	-1.04475100
O	-3.31627800	-0.43971700	0.14446600
C	-4.19581300	-0.30317000	-0.98168900
H	-4.72436900	0.63650200	-0.82849300
H	-3.61651100	-0.26389900	-1.90805700
H	-4.90260500	-1.13492000	-1.00924100
H	-0.33734900	-1.30943600	-0.89198600

[$MeNH_3$] $MeSO_4$

N	2.46555100	-0.90460800	-0.20055300
H	1.64436200	-1.06743200	0.42044600
C	3.25083900	0.24697600	0.31947600
H	3.61731600	-0.00036500	1.31351400
H	4.08062700	0.44596000	-0.35526700
H	2.57939800	1.10252300	0.36095000
H	3.02587200	-1.75399400	-0.29463400
S	-0.53781200	0.38100600	-0.03572600
O	0.36529600	0.57756500	-1.18059000
O	-1.20842200	1.58310500	0.45448800
O	-1.69459200	-0.56486100	-0.68121100
O	0.07991500	-0.43535400	1.03965300
C	-2.74294400	-0.92276200	0.22493500
H	-3.42702100	-1.55300500	-0.34188700
H	-2.33612600	-1.47961100	1.07314200
H	-3.26134000	-0.02753900	0.57679100
H	2.03810400	-0.65930600	-1.10276200

3ac

C	-4.13259100	0.37810700	0.00039700
C	-2.88470600	0.99086200	0.00021000

C	-1.72579300	0.21147500	-0.00014100
C	-1.81560700	-1.18274500	-0.00029300
C	-3.06646700	-1.79224100	-0.00010600
C	-4.22322300	-1.01371800	0.00023900
H	-5.03359900	0.98286300	0.00066500
H	-2.79011500	2.07180700	0.00032600
H	-0.91083100	-1.78029400	-0.00056100
H	-3.13936800	-2.87496600	-0.00022900
H	-5.19750200	-1.49258200	0.00038500
C	-0.41195600	0.91673300	-0.00034900
O	-0.28799300	2.12295200	0.00004300
C	1.92832200	0.68166500	-0.00023200
H	2.02061400	1.31899100	-0.88574200
H	2.02032800	1.31910600	0.88522100
O	0.62658900	0.07399200	-0.00041300
C	2.95654500	-0.43069400	-0.00000400
H	2.80068000	-1.06327400	-0.88227600
H	2.80044900	-1.06312800	0.88233200
C	4.38170400	0.12052900	0.00013700
H	4.52359400	0.76138600	0.87920700
H	4.52382200	0.76123700	-0.87900600
C	5.42659500	-0.99237000	0.00036700
H	5.31744700	-1.62846600	-0.88413400
H	6.44155300	-0.58553100	0.00046200
H	5.31722000	-1.62831200	0.88495100

Supplementary References:

(1) Pan, F.; Shen, P.; Zhang, L.; Wang, X.; Shi, Z. *Org. Lett.* **2013**, *15*, 4758 – 4761.

(2) Ano, Y.; Tobisu, M.; Chatani, N. *Org. Lett.* **2012**, *14*, 354 – 357.

(3) Wang, H.; Park, Y.; Bai, Z.; Chang, S.; He, G.; Chen, G. *J. Am. Chem. Soc.* **2019**, *141*, 7194 – 7201.

(4) Reddy, B. V.; Reddy, L. R.; Corey. *Org. Lett.* **2006**, *8*, 3391 – 3394.

(5) Zeng, L.; Tang, S.; Wang, D.; Deng, Y.; Chen, J.; Lee, J.; Lei, A. *Org. Lett.* **2017**, *19*, 2170 – 2173.

(6) Govindan, K.; Chen, N.; Chuang, Y.; Lin, W. *Org. Lett.* **2021**, *23*, 9419 – 9424.

(7) Ulatowski, F.; Jurczak, J. *Tetrahedron Asymmetry*, **2014**, 25, 962 – 968.

(8) Maegawa, Y.; Agura, K.; Hayashi, Y.; Ohshima, T.; Mashima, K. *Synlett*, **2012**, 13, 137 – 141.

(9) Weinhold, T.; Reece, N..; Ribeiro, K.; Lopez Ocasio, M.; Watson, N.; Hanson, K.; Longstreet, A. R. *J. Org. Chem.* **2022**, *87*, 16928 – 16936.

(10) Ganesan, V.; Moon, Seokyeong; Yoon, S. *J. Org. Chem.* **2023**, *88*, 5127 – 5134.

(11) Liu, H.; Shi, G.; Pan, S.; Jiang, Y.; Zhang, Y. *Org. Lett.* **2013**, *15*, 4098 – 4101.

(12) Tang, Z.; Jiang, Q.; Peng, L.; Xu, X.; Li, J.; Qiu, R.; Au, C. *Green Chem.* **2017**, *19*, 5396 – 5402.

(13) Wakita, N.; Hara, S. *Tetrahedron* **2010**, *66*, 7939 – 7945.

(14) Gaspa, S.; Porcheddu, A.; De Luca, L. *Org. Lett.* **2015**, *17*, 3666 – 3669.

(15) Gan, S.; Shi, L.; Song, L.; Yin, J.; Yu, Z. *Green Chem.* **2022**, *24*, 2232 – 2239.

(16) Zhou, Y.; Yang, D.; Luo, G.; Zhao, Y.; Luo, Y.; Xue, N.; Qu, J. *Tetrahedron* **2014**, *70*, 4668 – 4674.

(17) Yang, X.; Guo, Y.; Tong, H.; Liu, R.; Zhou, R. *Green Chem.* **2023**, *25*, 1672 – 1678.

(18) Cao, Z.; Chen, X.; Chen, Y.; Kong, X.; Lu, Z.; Ni, Sh.; Wang, W. *Org. Lett.* **2022**, *24*, 2137 – 2142.

(19) Chakraborti, A.; Singh, B.; Chankeshwara, S.; Patel, A. *J. Org. Chem.* **2009**, *74*, 5967 – 5974.

(20) Zhu, Y.; Yan, H.; Lu, L.; Liu, D.; Rong, G.; Mao, J. *J. Org. Chem.* **2013**, *78*, 9898 – 9905.

(21) Wang, Y.; Chang, Z.; Hu, Y.; Lin, X.; Dou, X. *Org. Lett.* **2021**, *23*, 1910 – 1914.

(22) Pandey, G.; Koley, S.; Talukdar, R.; Sahani, P. *Org. Lett.* **2018**, *20*, 5861 – 5865.

(23) Tsukamoto, Y.; Itoh, S.; Kobayashi, M.; Obora, Y. *Org. Lett.* **2019**, *21*, 3299 – 3303.

(24) Ma, C.; Zhao, C.; Xu, X.; Li, Z.; Wang, X.; Zhang, K.; Mei, T. *Org. Lett.* **2019**, *21*, p. 2464 – 2467.

(25) Sonam, N.; Shinde, V.; Kumar, A. *J. Org. Chem.* **2022**, *87*, 2651 – 2661.

(26) Shang, R.; Fu, Y.; Li, J.; Zhang, S.; Guo, Q.; Liu, L. *J. Am. Chem. Soc.* **2009**, *131*, 5738 – 5739.

(27) Mašek, T.; Jahn, U. *J. Org. Chem.* **2021**, *86*, 11608 – 11632.

(28) Nishii, Y.; Akiyama, S.; Kita, Y.; Mashima, K. *Synlett*, **2015**, *26*, 1831 – 1834.

(29) Fernández, E.; Rivero-Crespo, M.; Domínguez, I.; Rubio-Marqués, P.; Oliver-Meseguer, J.; Liu, L.; Cabrero-Antonino, M.; Leyva-Pérez, A.; Corma, A. *J. Am. Chem. Soc.* **2019**, *141*, 1928 – 1940.

(30) Shendage, D.; Froehlich, R.; Haufe, G. *Org. Lett.* **2004**, *6*, 3675 – 3678.

(31)Nathanael, J.; Wille, U. *J. Org. Chem.* **2019**, *84*, 3405 – 3418.
(32)Britton, J.; Dalziel, S.; Raston, C. L. *Green Chem.* **2016**, 18, 2193 – 2200.
(33)Goyal, V.; Bhatt, T.; Dewangan, C.; Narani, Anand; N., Ganesh; B., Ekambaram; Natte, K.; Jagadeesh, R. *J. Org. Chem.* **2023**, *88*, 2245 – 2259.
(34)Xu, F.; Simmons, B.; Reamer, R..; Corley, E.; Murry, J.; Tschaen, D. *J. Org. Chem.* **2008**, 73, 312 – 315.
(35)Tien, C.; Adams, M..; Ferguson, M..; Johnson, E..; Speed, Al.H. *Org. Lett.* **2017**, 19, 5565 – 5568.
(36)Mondal, A.; Karattil Suresh, A.; Sivakumar, G.; Balaraman, E. *Org. Lett.* **2022**, 24, 8990 – 8995.
(37)Vignaroli, G.; Zamperini, C.; Dreassi, E.; Radi, M.; Angelucci, A.; Sanita, P.; Crespan, E.; Musumeci, F.; Botta, M. *ACS Med. Chem. Lett.* **2013**, 4, 622 – 626.
(38)Kapitanov, I.; Jordan, A.; Karpichev, Y.; Spulak, M.; Perez, L.; Kellett, A.; Kümmerer, K.; Gathergood, N. *Green Chem.* **2019**, 21, 1777 – 1794.
(39)Gaussian 16, Revision B.01,M. J. Frisch, G. W. Trucks, H. B. Schlegel, G. E. Scuseria, M. A. Robb, J. R. Cheeseman, G. Scalmani, V. Barone, G. A. Petersson, H. Nakatsuji, X. Li, M. Caricato, A. V. Marenich, J. Bloino, B. G. Janesko, R. Gomperts, B. Mennucci, H. P. Hratchian, J. V. Ortiz, A. F. Izmaylov, J. L. Sonnenberg, D. Williams-Young, F. Ding, F. Lipparini, F. Egidi, J. Goings, B. Peng, A. Petrone, T. Henderson, D. Ranasinghe, V. G. Zakrzewski, J. Gao, N. Rega, G. Zheng, W. Liang, M. Hada, M. Ehara, K. Toyota, R. Fukuda, J. Hasegawa, M. Ishida, T. Nakajima, Y. Honda, O. Kitao, H. Nakai, T. Vreven, K. Throssell, J. A. Montgomery, Jr., J. E. Peralta, F. Ogliaro, M. J. Bearpark, J. J. Heyd, E. N. Brothers, K. N. Kudin, V. N. Staroverov, T. A. Keith, R. Kobayashi, J. Normand, K. Raghavachari, A. P. Rendell, J. C. Burant, S. S. Iyengar, J. Tomasi, M. Cossi, J. M. Millam, M. Klene, C. Adamo, R. Cammi, J. W. Ochterski, R. L. Martin, K. Morokuma, O. Farkas, J. B. Foresman, and D. J. Fox, Gaussian, Inc., Wallingford CT, 2016.
(40)Zhao, Y., Truhlar, D.G. The M06 suite of density functionals for main group thermochemistry, thermochemical kinetics, noncovalent interactions, excited states, and transition elements: two new functionals and systematic testing of four M06-class functionals and 12 other functionals. Theor Chem Account 2008, 120, 215–241.
(41)(a) Ditchfield, R., Hehre, W. J., Pople, J. A. Self-Consistent Molecular-Orbital Methods. IX. An Extended Gaussian-Type Basis for Molecular-Orbital Studies of Organic Molecules. J. Chem. Phys. 1971, 54, 724-728. (b) Hariharan, P. C., Pople, J. A. The influence of polarization functions on molecular orbital. hydrogenation energies. Theor. Chim. Acta 1973, 28, 213-222. (c) Hehre, W. J., Ditchfield, R., Pople, J. A. Self-Consistent Molecular Orbital Methods. XII. Further Extensions of Gaussian-Type Basis Sets for Use in Molecular Orbital Studies of Organic Molecules. J. Chem. Phys. 1972, 56, 2257-2261.
(42)G. Scalmani and M. J. Frisch, Continuous surface charge polarizable continuum models of solvation. I. General formalism, J. Chem. Phys., 2010,132, 114110.
(43)(a) Gonzalez, C.; Schlegel, H. B. An improved algorithm for reaction path following. J. Chem. Phys. 1989, 90, 2154. (b) Gonzalez, C.; Schlegel, H. B. Reaction Path Following in Mass-Weighted Internal Coordinates. J. Phys. Chem. 1990, 94, 5523.
(44)(a) Krishnan, R., Binkley, J. S., Seeger, R., Pople, J. A. Self-consistent molecular orbital methods. XX. A basis set for correlated wave functions. J. Chem. Phys. 1980, 72, 650-654. (b)

Clark, Timothy, Chandrasekhar, Jayaraman, Spitznagel, Günther W., Schleyer, Paul Von Ragué. Efficient diffuse function-augmented basis sets for anion calculations. III. The 3-21+G basis set for first-row elements, Li-F. J. Comput. Chem. 1983, 4, 294-301. (c) Francl, Michelle M., Pietro, William J., Hehre, Warren J., Binkley, J. Stephen, Gordon, Mark S., DeFrees, Douglas J., Pople, John A. Self-consistent molecular orbital methods. XXIII. A polarization-type basis set for second-row elements. J. Chem. Phys. 1982, 77, 3654-3665. (d) Spitznagel, Günther W., Clark, Timothy, Schleyer, Paul von Ragué, Hehre, Warren J. An evaluation of the performance of diffuse function-augmented basis sets for second row elements, Na-Cl. J. Comput. Chem. 1987, 8, 1109-1116.

(45) Marenich AV, Cramer CJ, Truhlar DG. Universal solvation model based on solute electron density and on a continuum model of the solvent defined by the bulk dielectric constant and atomic surface tensions. J Phys Chem B. 2009 May 7;113(18):6378-96.
