# Supplementary material for: Direct esterification of amides by the dimethylsulfate-mediated activation of amide C–N bonds
Source: Commun Chem. 2024 Apr 27;7:93. doi: 10.1038/s42004-024-01180-9 (PMC11055851; doi:10.1038/s42004-024-01180-9)
Supplement: Supplementary file 2 — Description of Additional Supplementary Files [file 42004_2024_1180_MOESM2_ESM.pdf]

Description of Additional Supplementary Files

File name: Supplementary Data 1

Description: ^{1}H NMR and ^{13}C NMR spectra

File name: Supplementary Data 2

Description: Source Data for Supplementary Tables S1−S8

File name: Supplementary Data 3

Description: Source Data for Supplementary Figures S1-S18

File name: Supplementary Data 4

Description: CIF (small molecule crystallographic data) of the X-ray crystallographic coordinates for structures for compound **INT-1z**.
