# Supplementary material for: Direct esterification of amides by the dimethylsulfate-mediated activation of amide C–N bonds
Source: Commun Chem. 2024 Apr 27;7:93. doi: 10.1038/s42004-024-01180-9 (PMC11055851; doi:10.1038/s42004-024-01180-9)
Supplement: Supplementary file 3 — Supplementary Data 1 file [file 42004_2024_1180_MOESM3_ESM.docx]

**Supplementary Data 1**

**Direct esterification of amides by the dimethylsulfate-mediated activation of amide C–N bonds**

# ^1^H and ^13^C NMR spectra

**Compound** **1hc** ^1^H NMR (400 MHz, CDCl_3_)


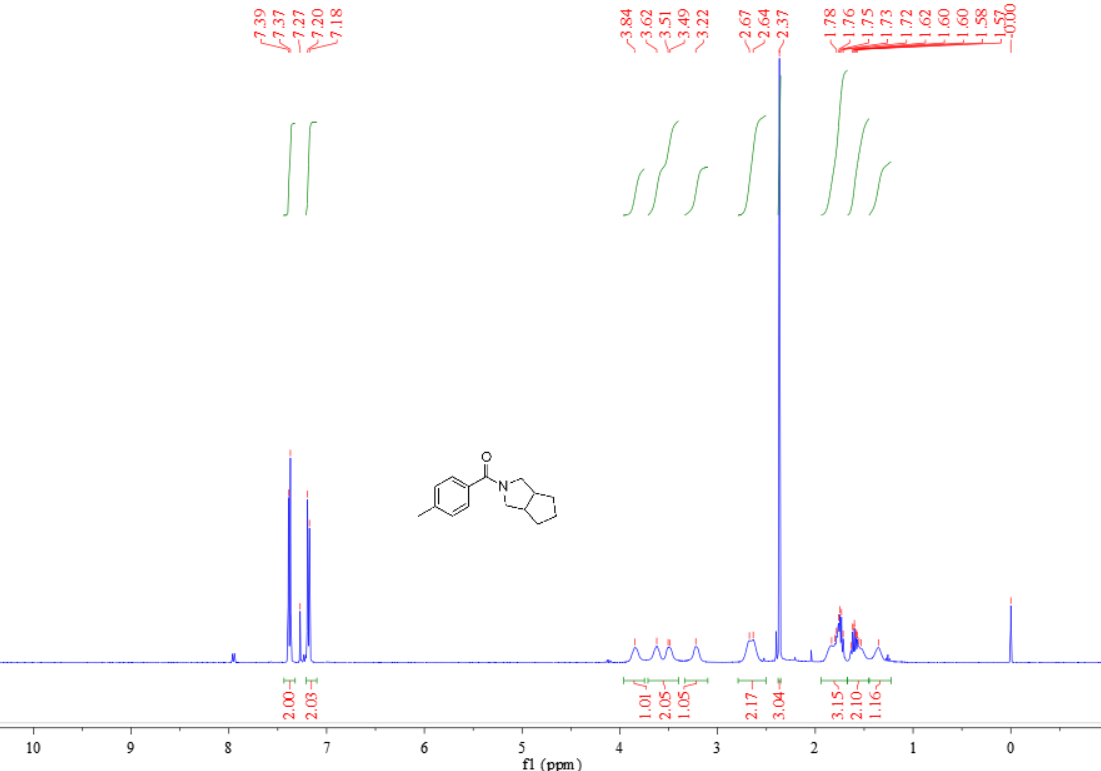


**Compound** **hc** ^13^C{^1^H}NMR (100 MHz, CDCl_3_)


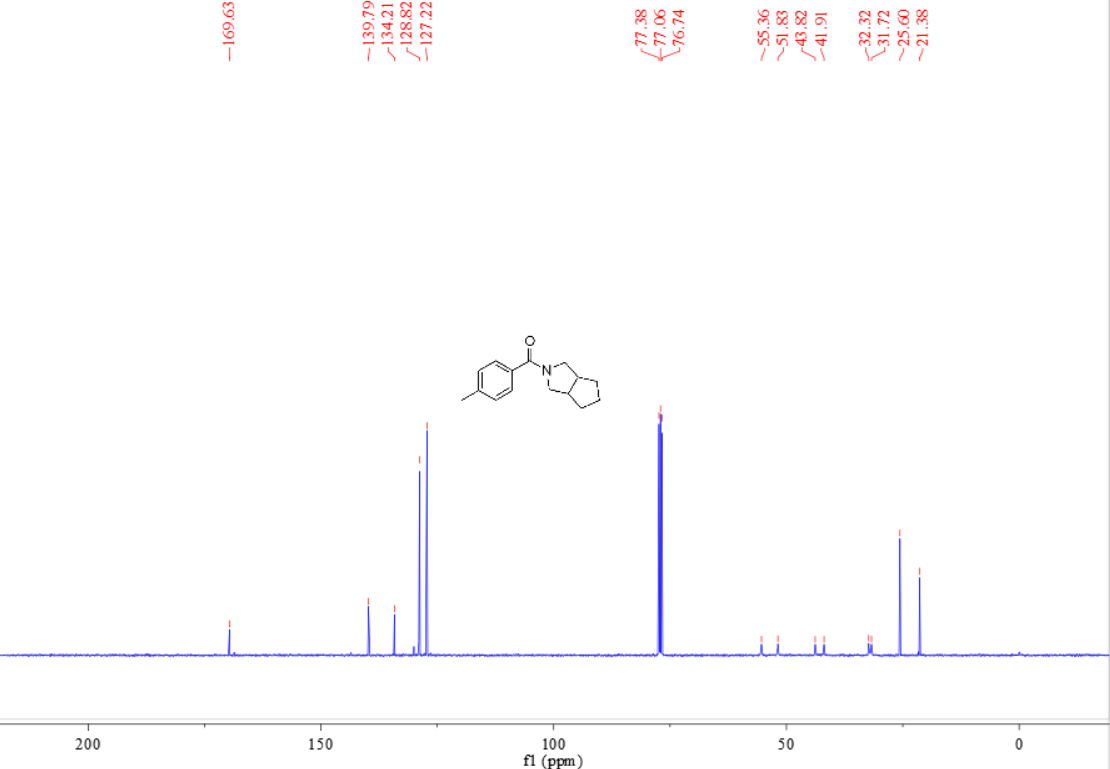


**Compound** **1k** ^1^H NMR (400 MHz, CDCl_3_)


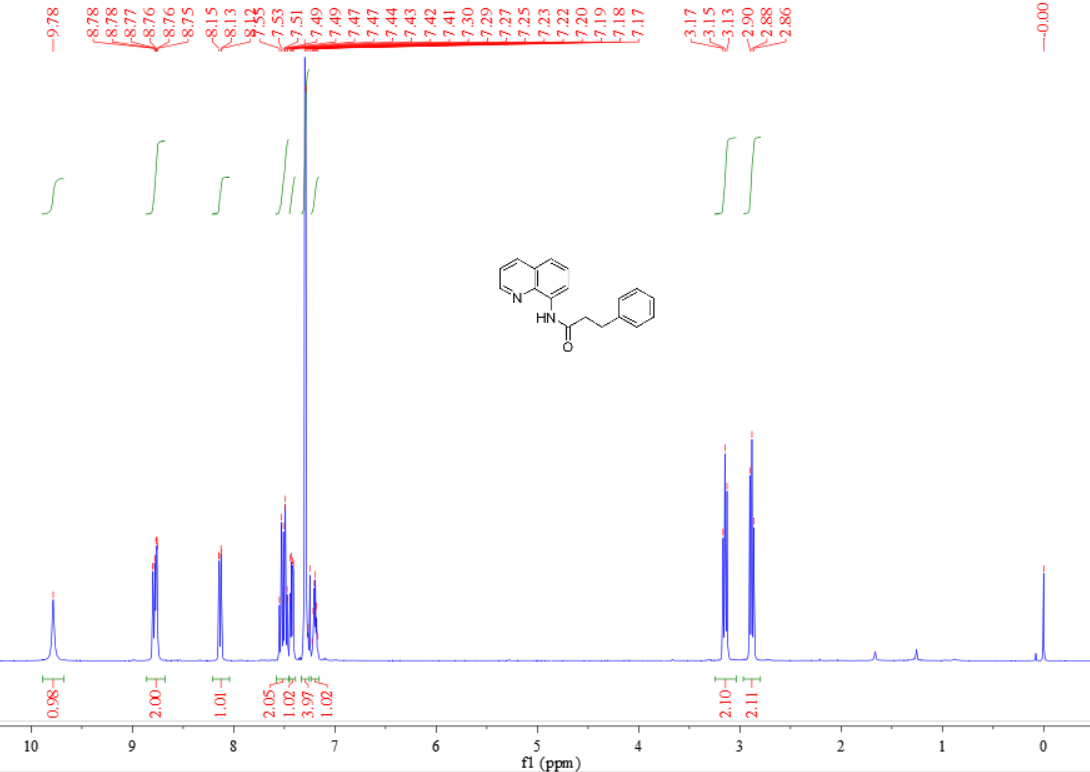


**Compound** **1k** ^13^C{^1^H}NMR (100 MHz, CDCl_3_)


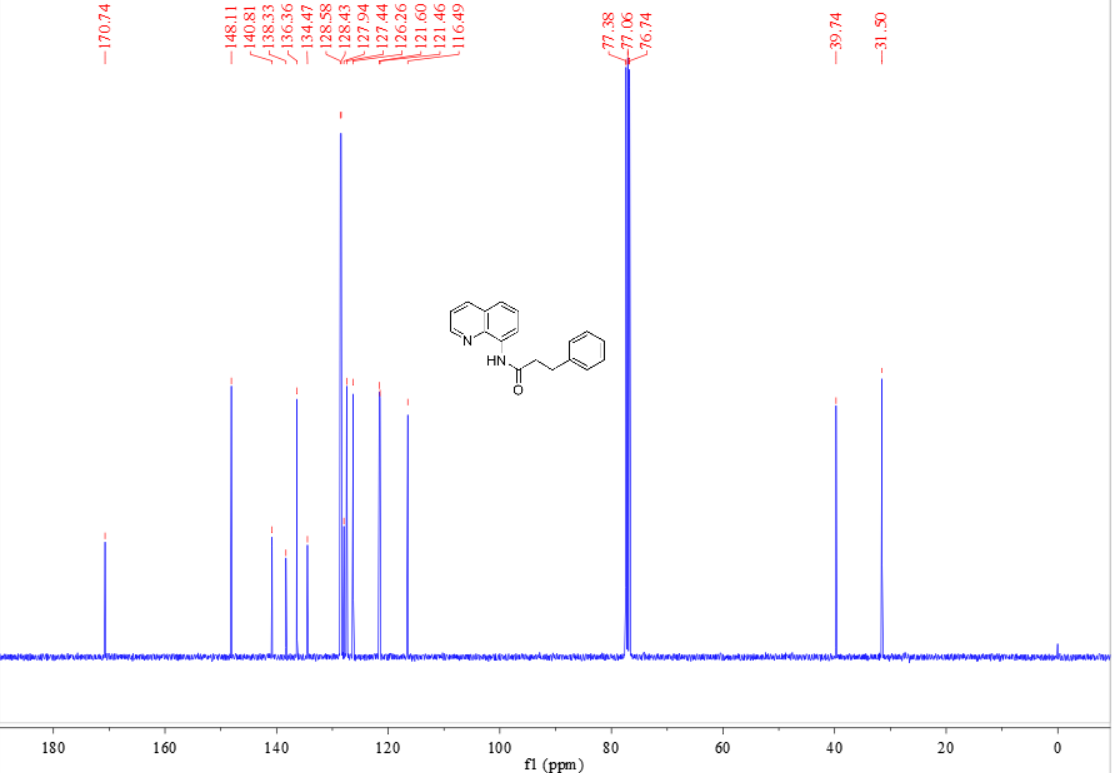


**Compound** **1l** ^1^H NMR (400 MHz, CDCl_3_)


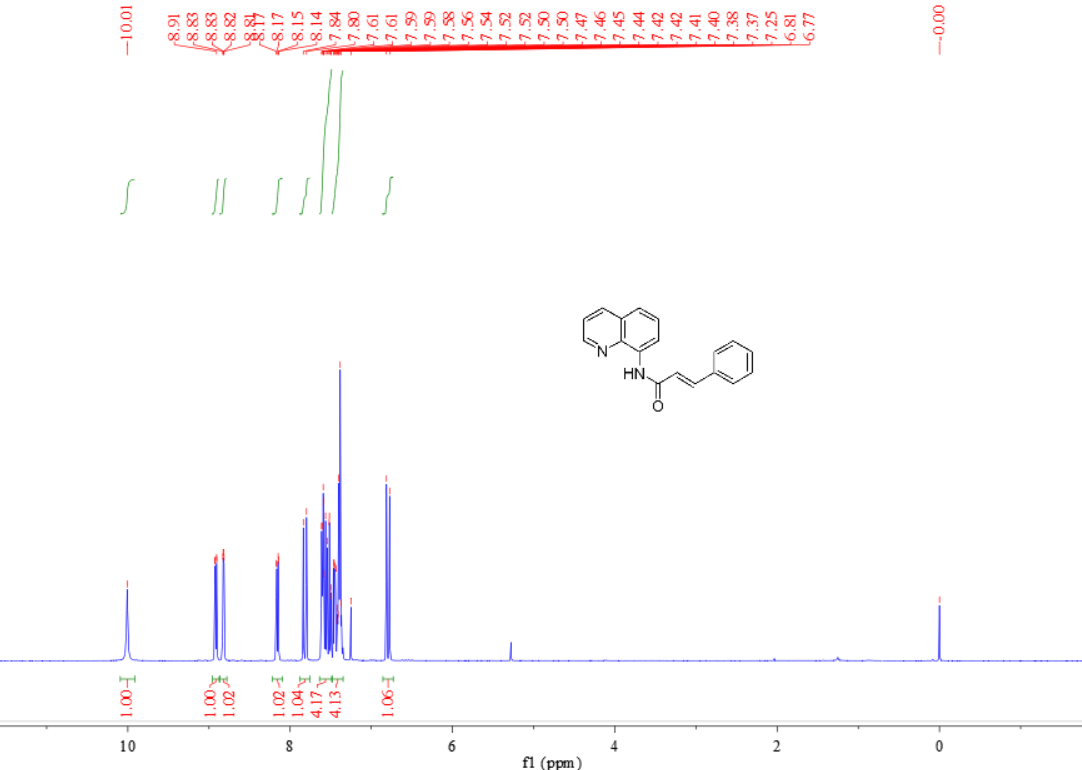


**Compound** **1l** ^13^C{^1^H}NMR (100 MHz, CDCl_3_)


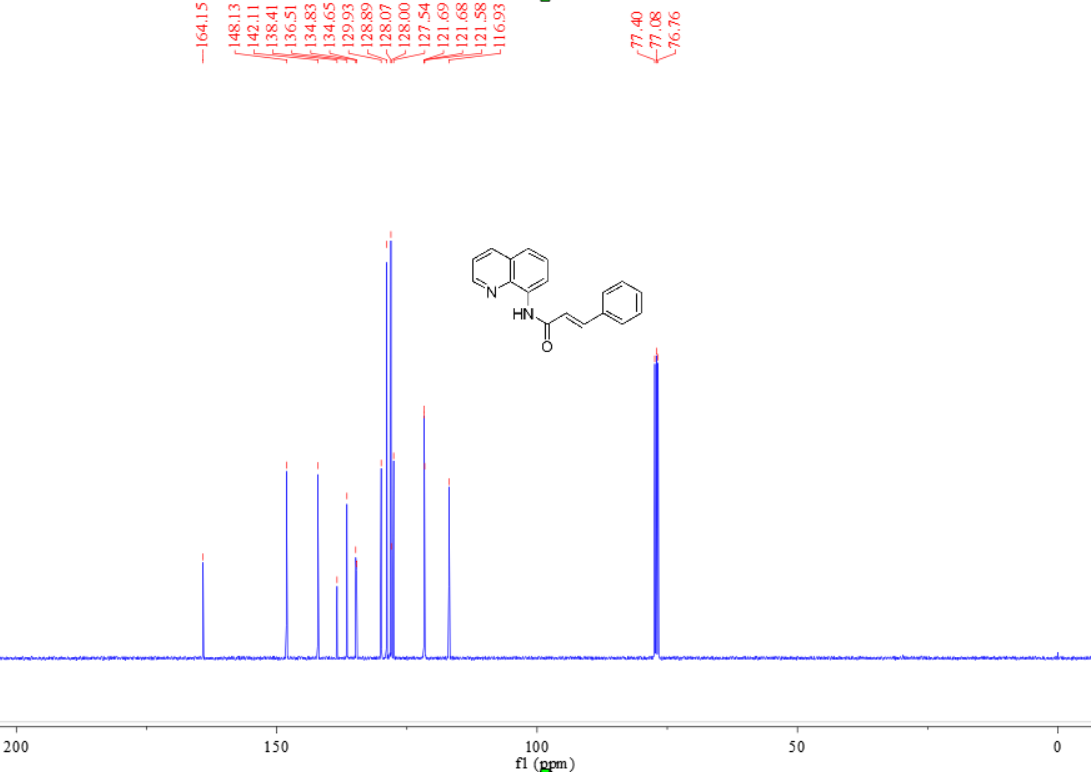


**Compound** **1m** ^1^H NMR (400 MHz, CDCl_3_)


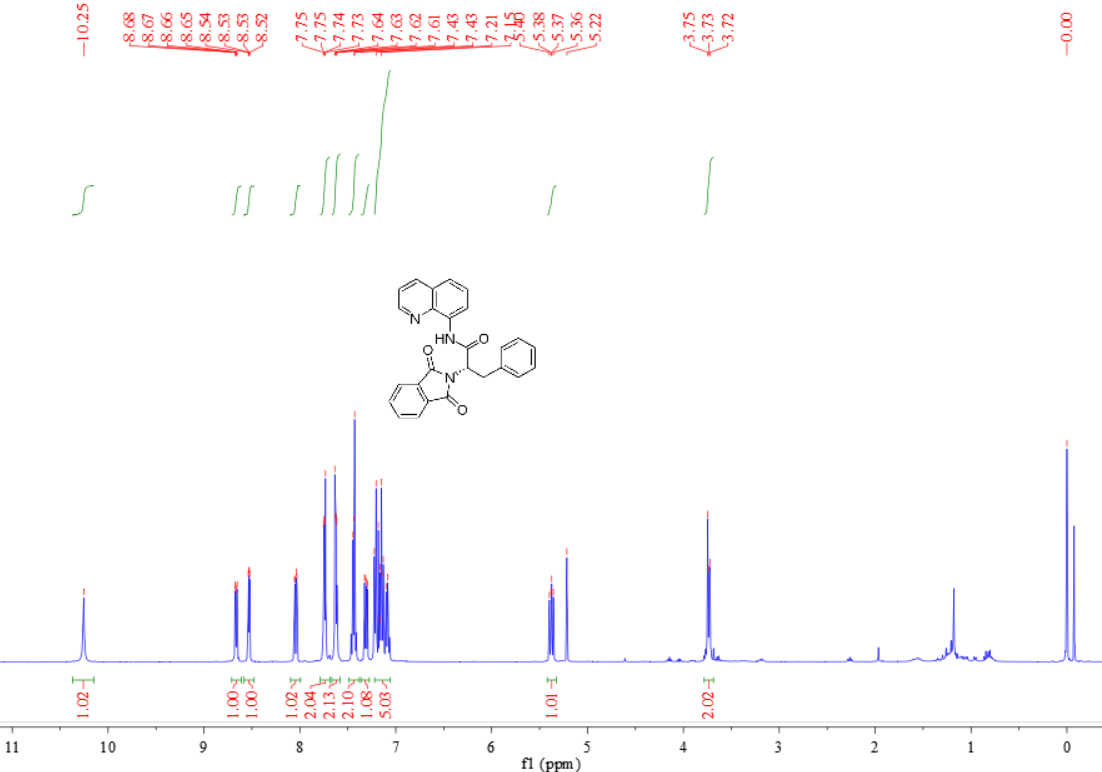


**Compound** **1m** ^13^C{^1^H}NMR (100 MHz, CDCl_3_)


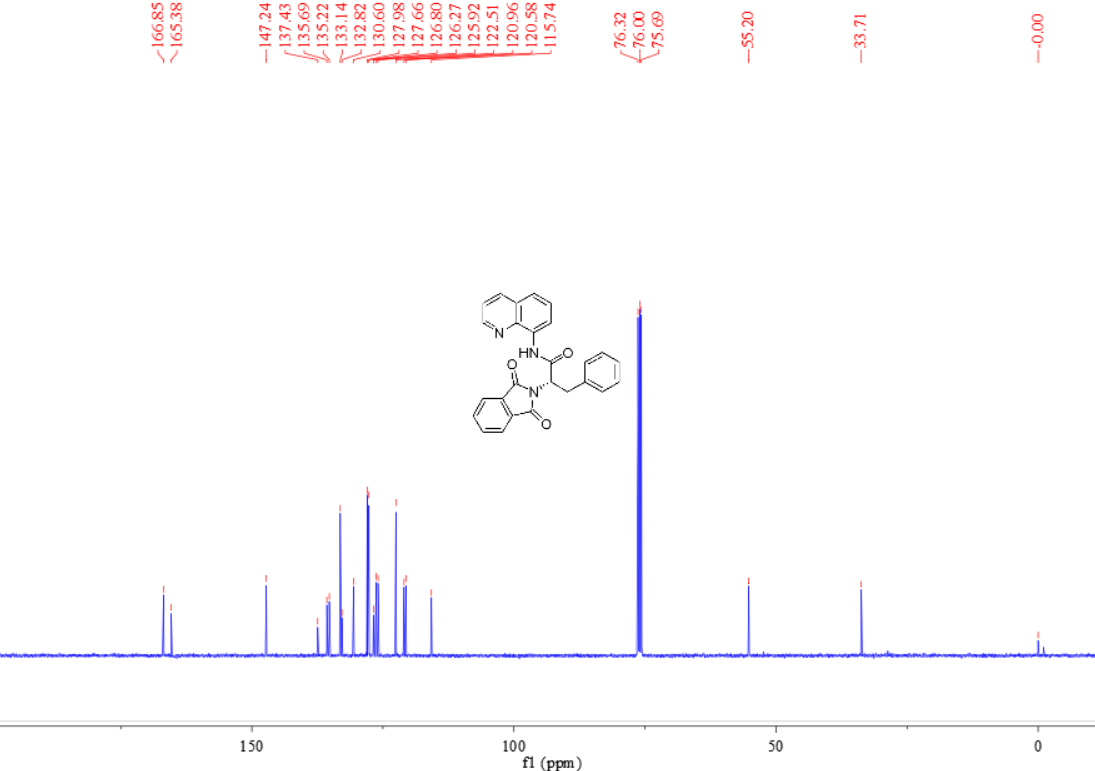


**Compound** **1n** ^1^H NMR (400 MHz, CDCl_3_)


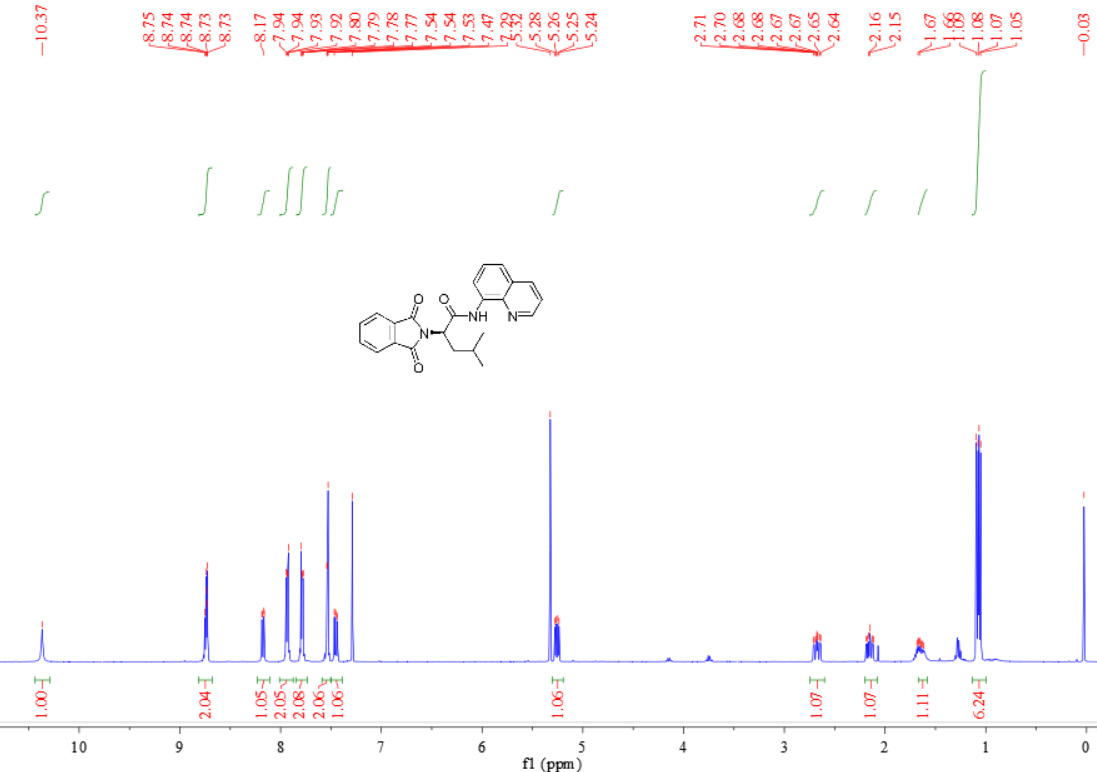


**Compound** **1n** ^13^C{^1^H}NMR (100 MHz, CDCl_3_)


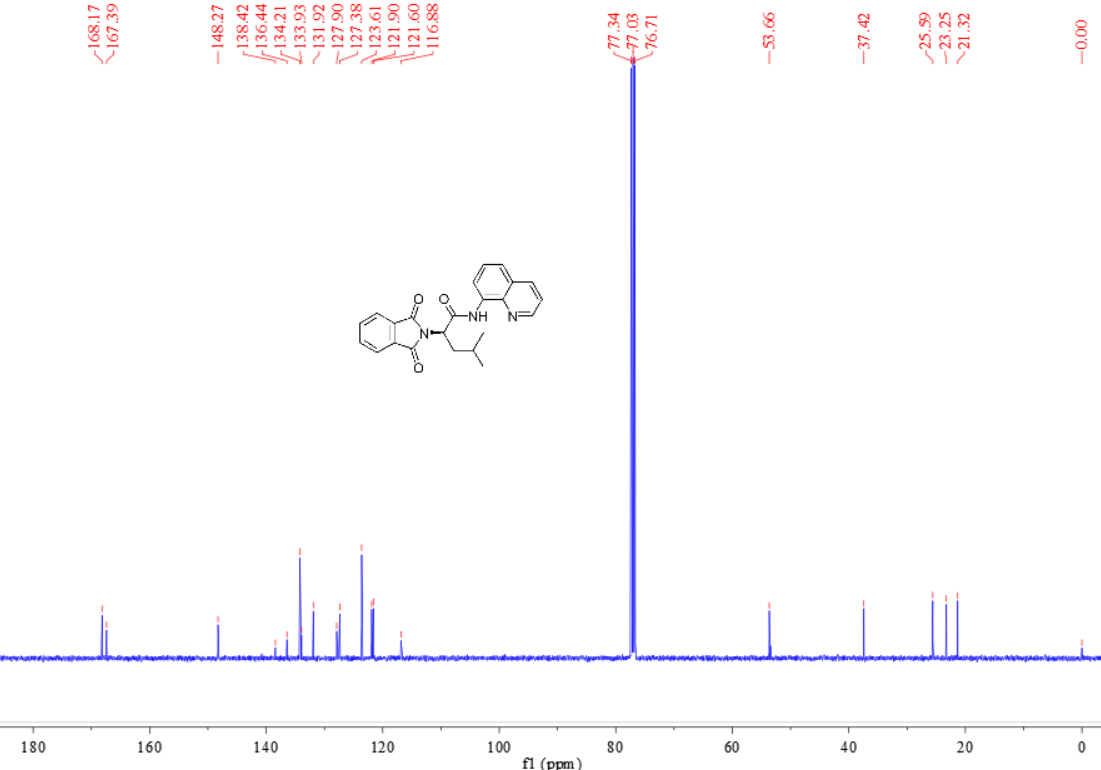


**Compound** **1nn** ^1^H NMR (400 MHz, CDCl_3_)


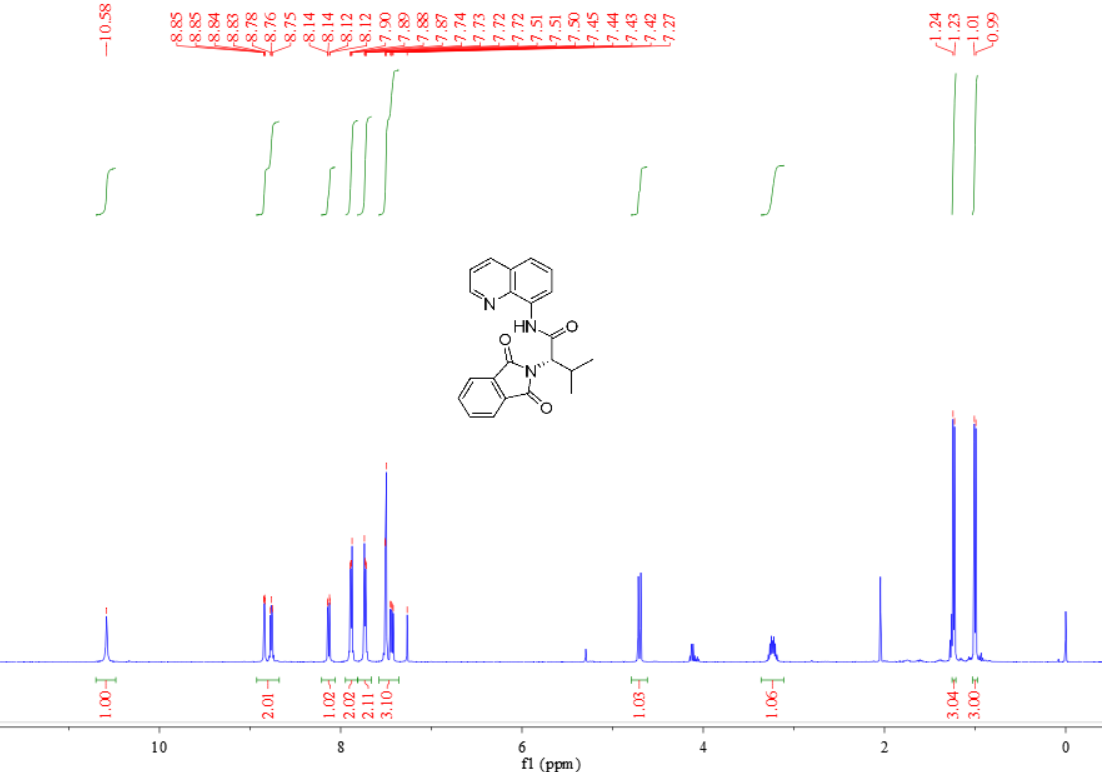


**Compound** **1nn** ^13^C{^1^H}NMR (100 MHz, CDCl_3_)


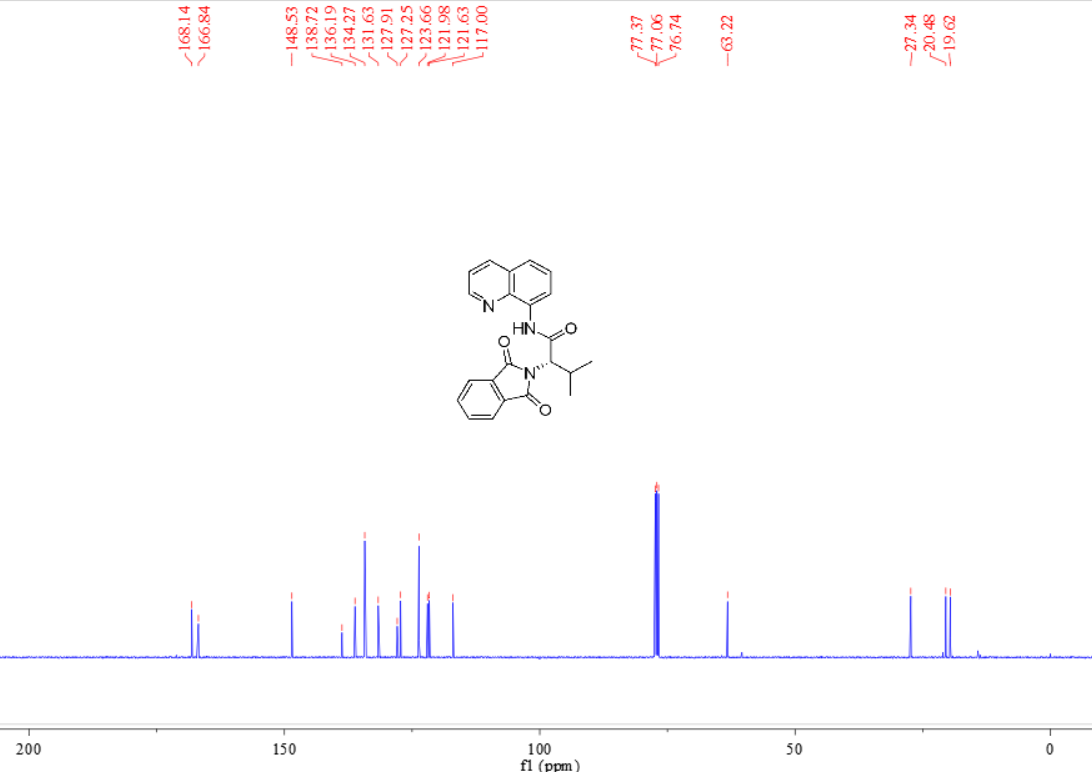


**Compound** **1of** ^1^H NMR (400 MHz, CDCl_3_)


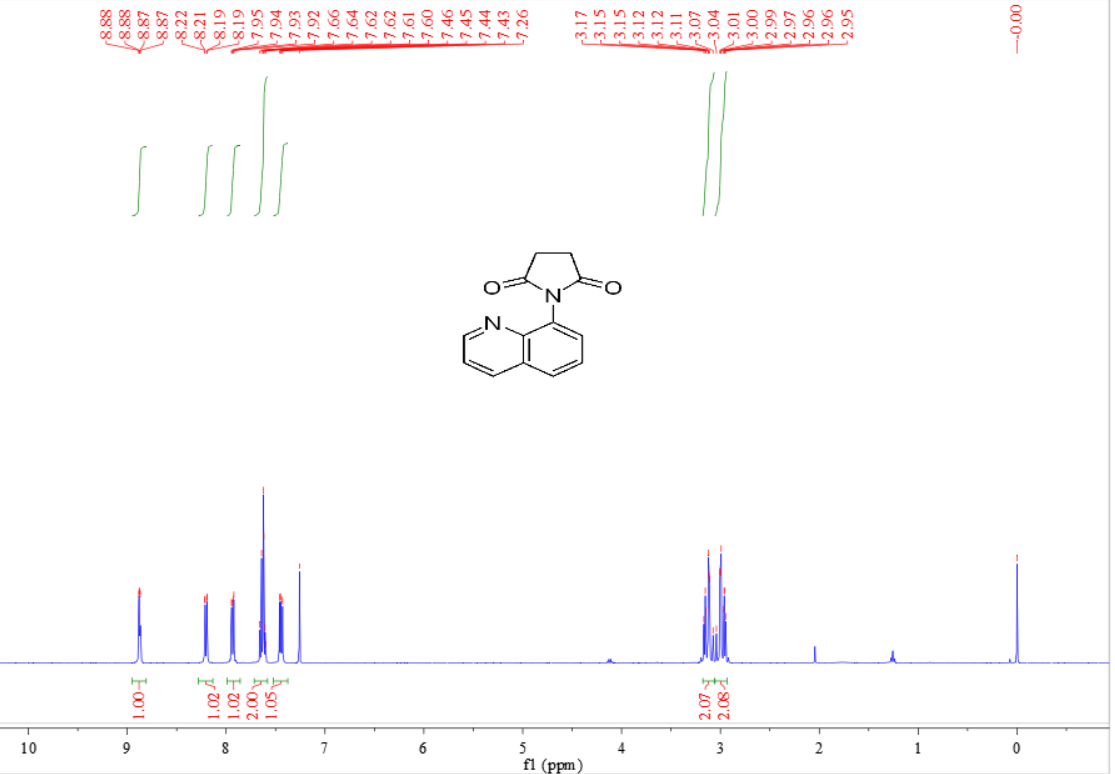


**Compound** **1of** ^13^C{^1^H}NMR (100 MHz, CDCl_3_)


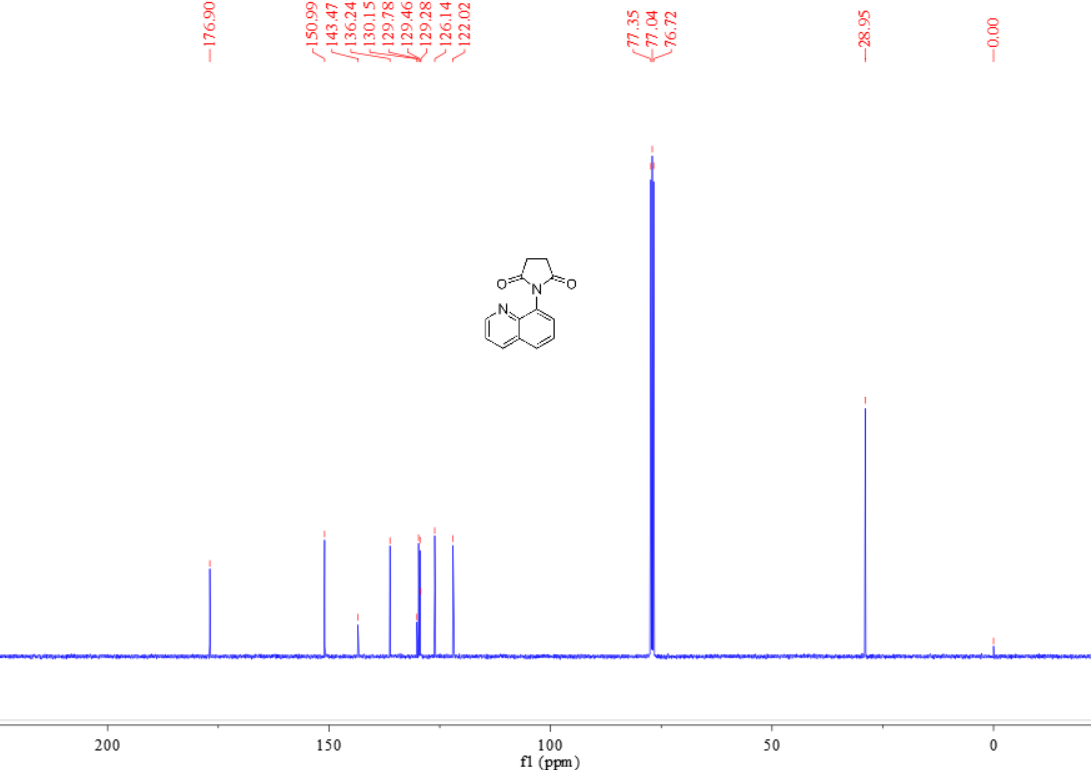


**Compound** **1og** ^1^H NMR (400 MHz, CDCl_3_)


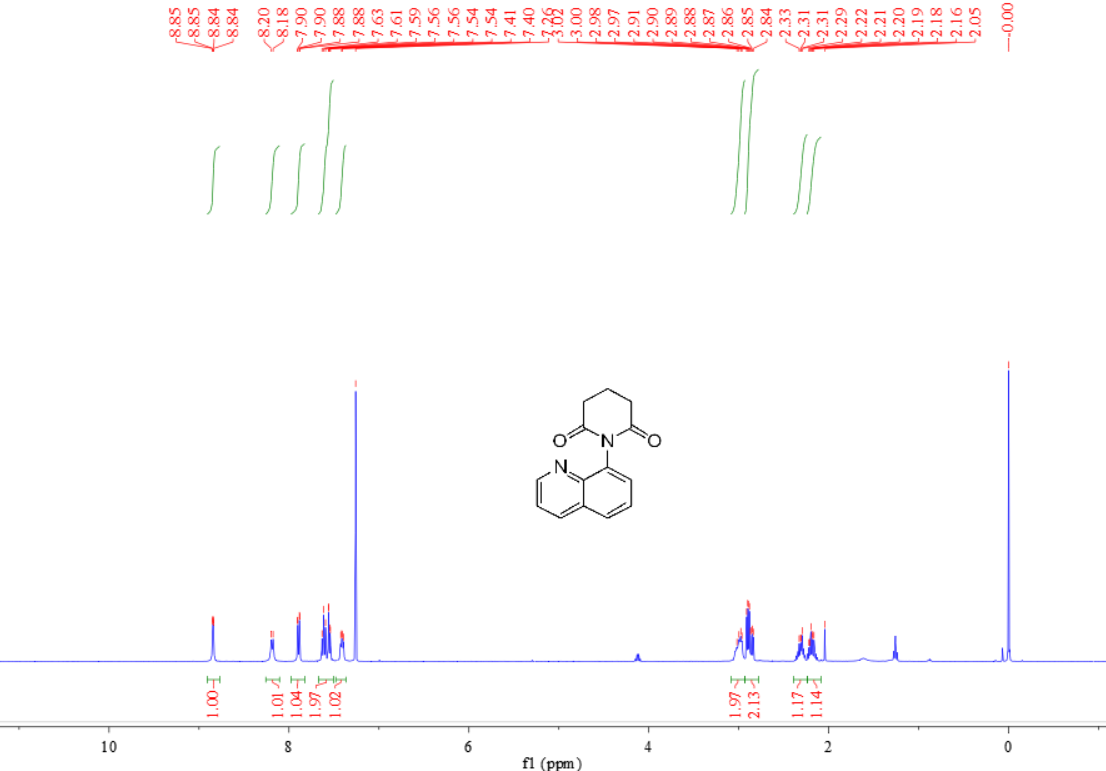


**Compound** **1og** ^13^C{^1^H}NMR (100 MHz, CDCl_3_)


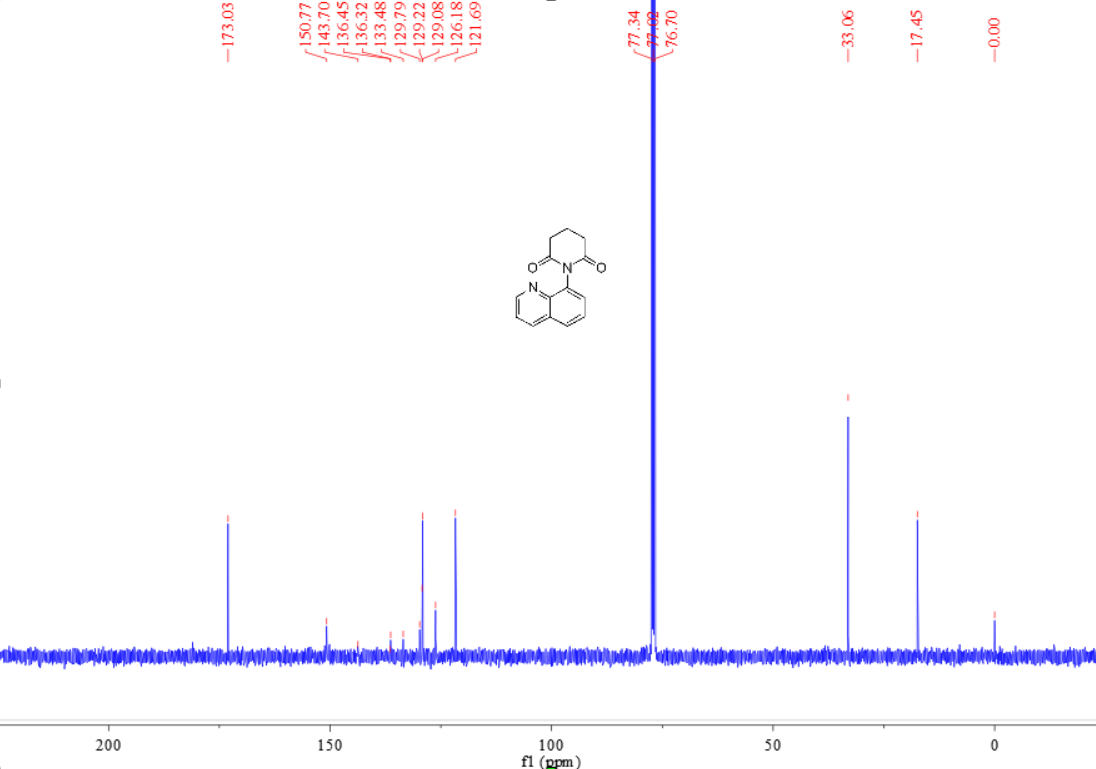


**Compound** **1u** ^1^H NMR (400 MHz, CDCl_3_)


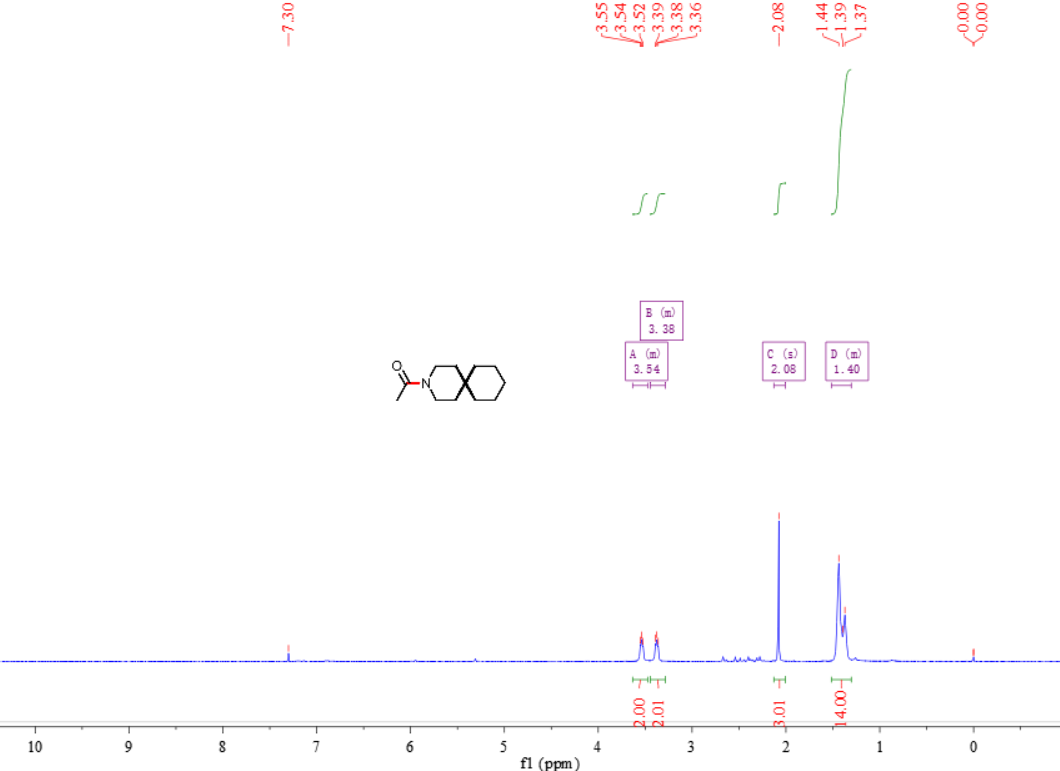


**Compound** **1u** ^13^C{^1^H}NMR (100 MHz, CDCl_3_)


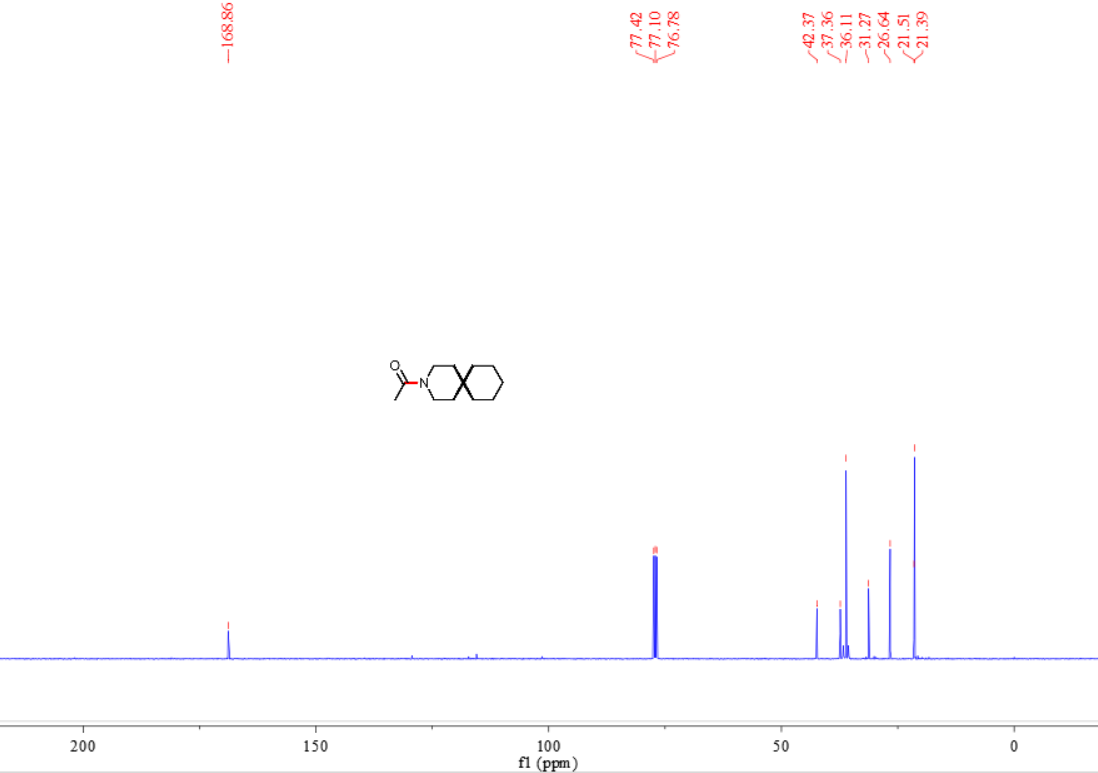


**Compound** **1ub** ^1^H NMR (400 MHz, CDCl_3_)


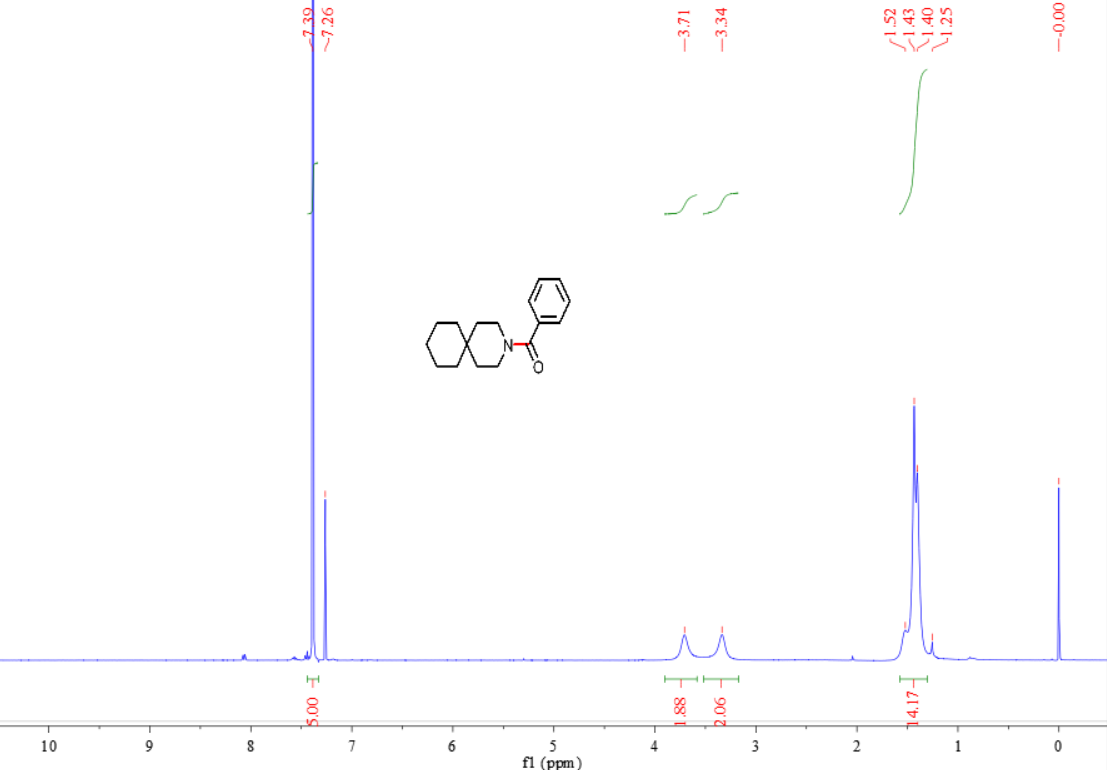


**Compound** **1ub** ^13^C{^1^H}NMR (100 MHz, CDCl_3_)


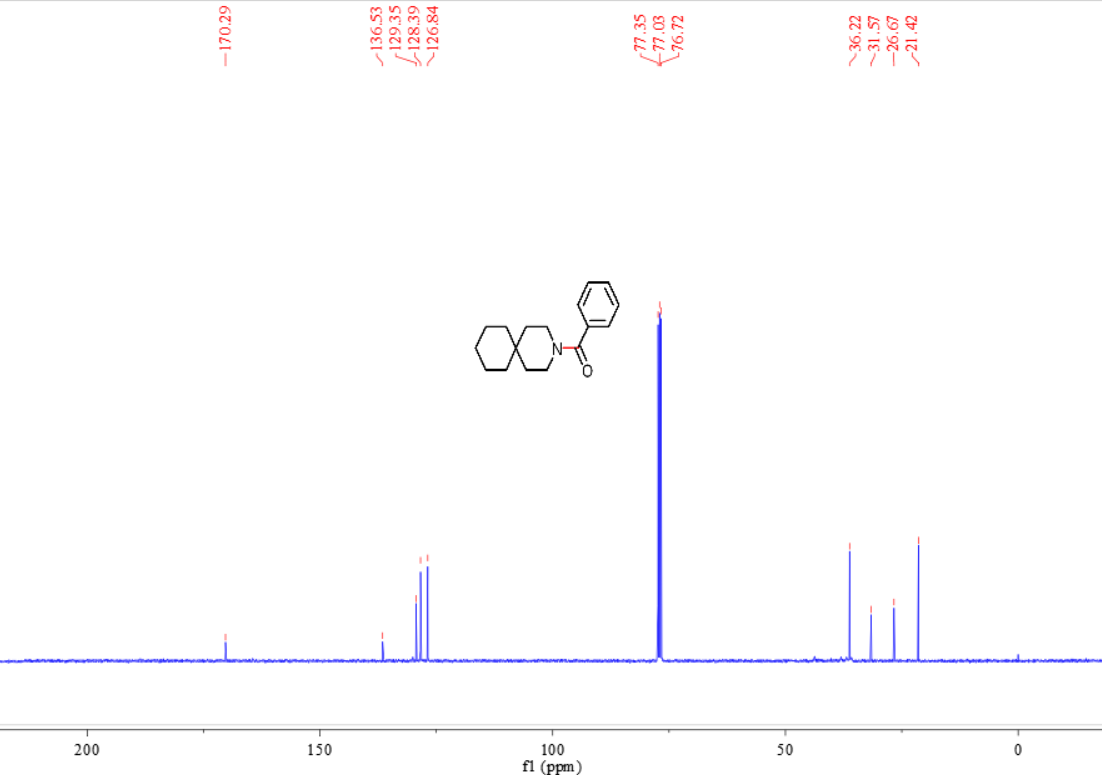


**Compound** **1uc** ^1^H NMR (400 MHz, CDCl_3_)


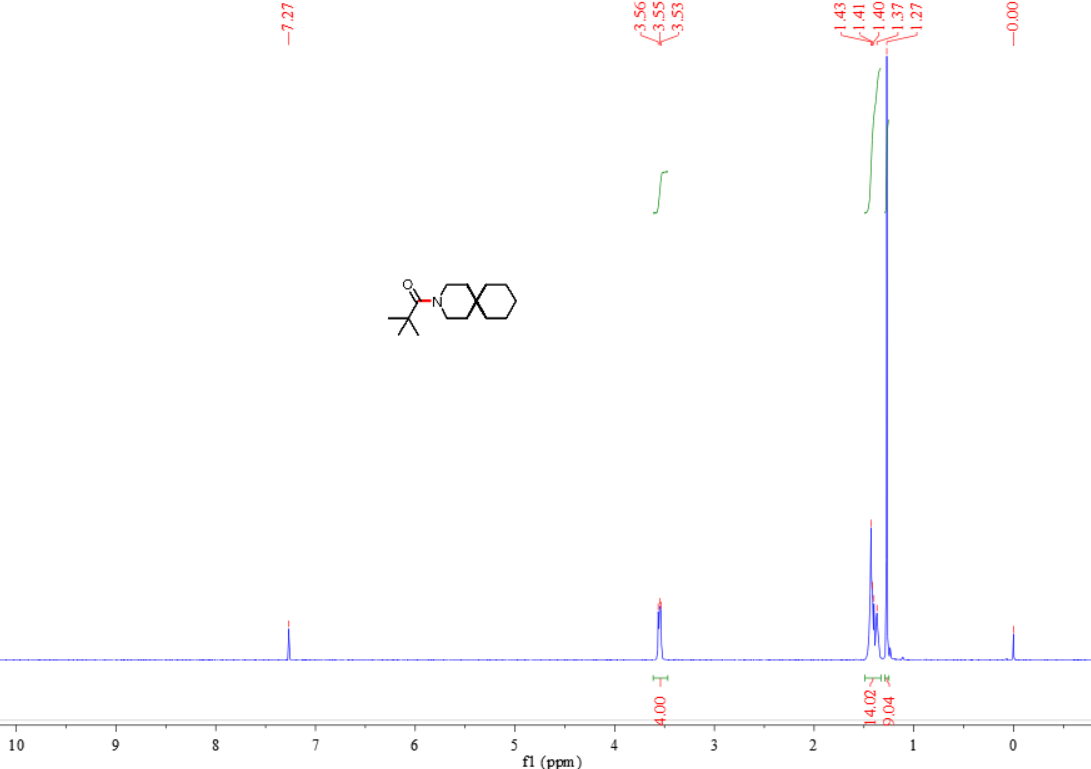


**Compound** **1uc** ^13^C{^1^H}NMR (100 MHz, CDCl_3_)


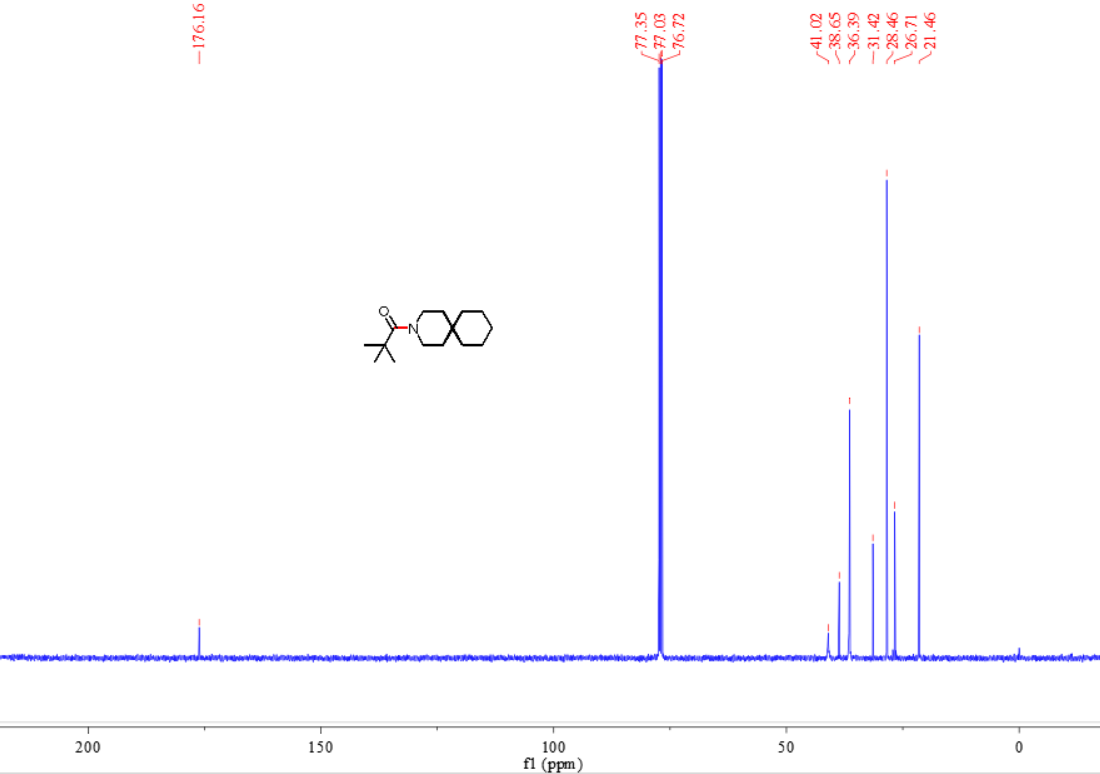


**Compound** **1ud** ^1^H NMR (400 MHz, CDCl_3_)


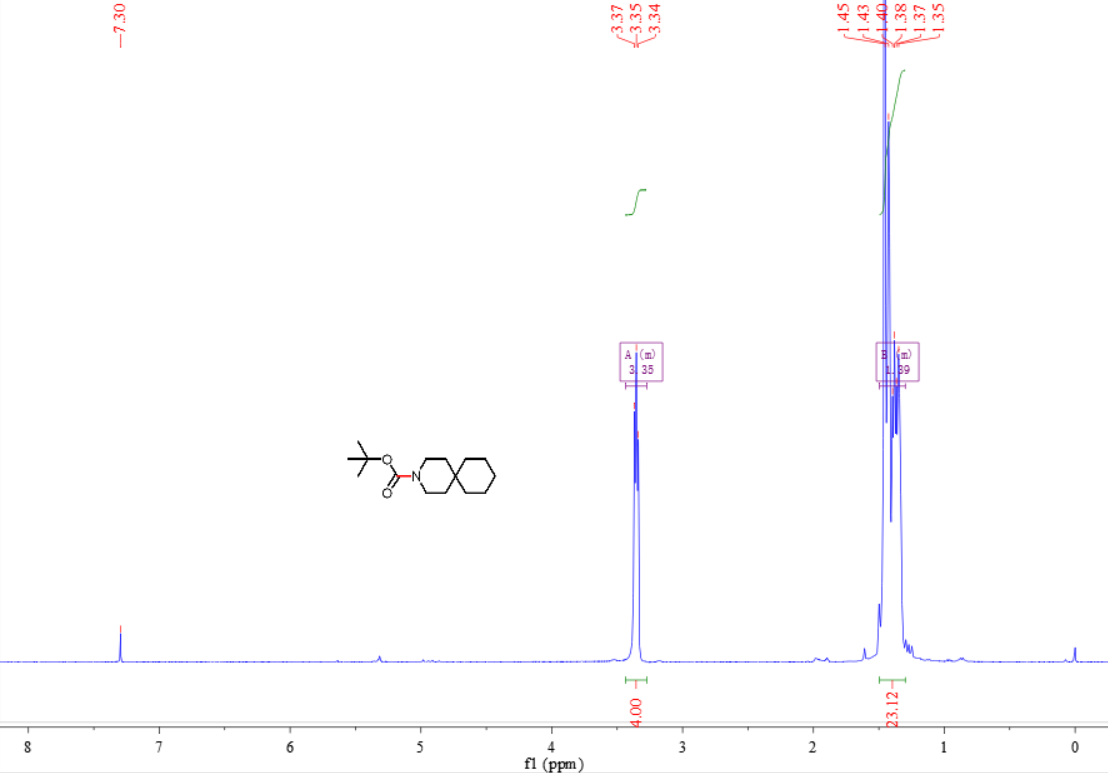


**Compound** **1ud** ^13^C{^1^H}NMR (100 MHz, CDCl_3_)


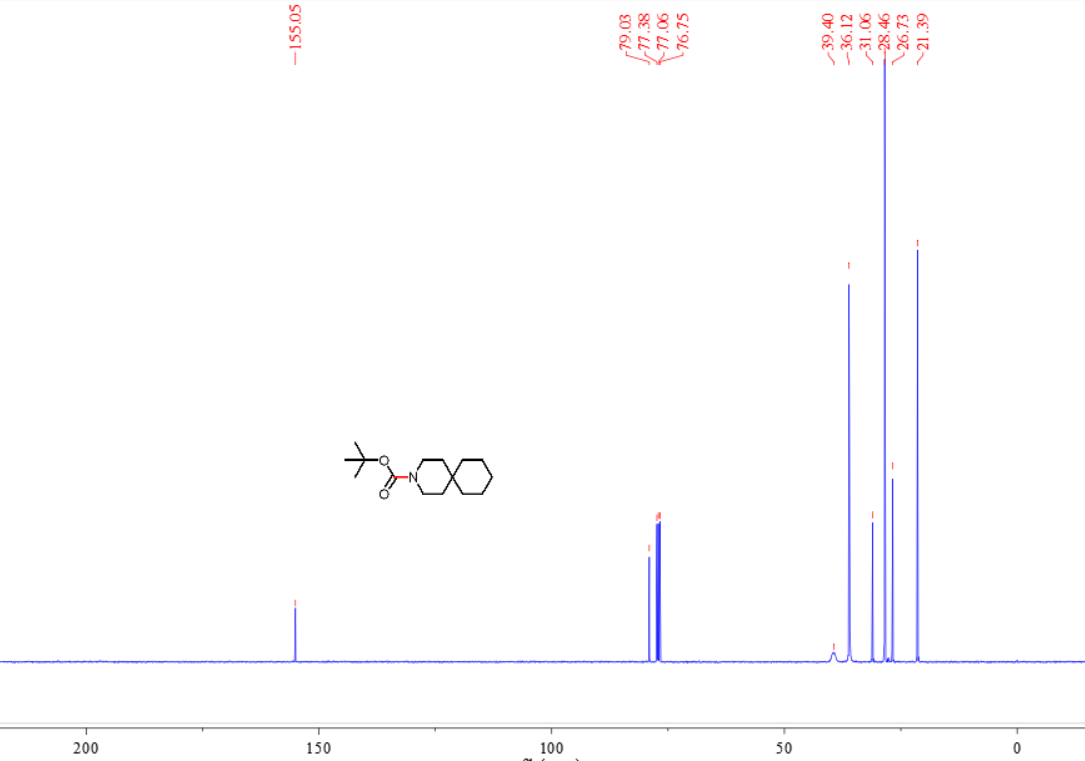


**Compound** **1ue** ^1^H NMR (400 MHz, CDCl_3_)


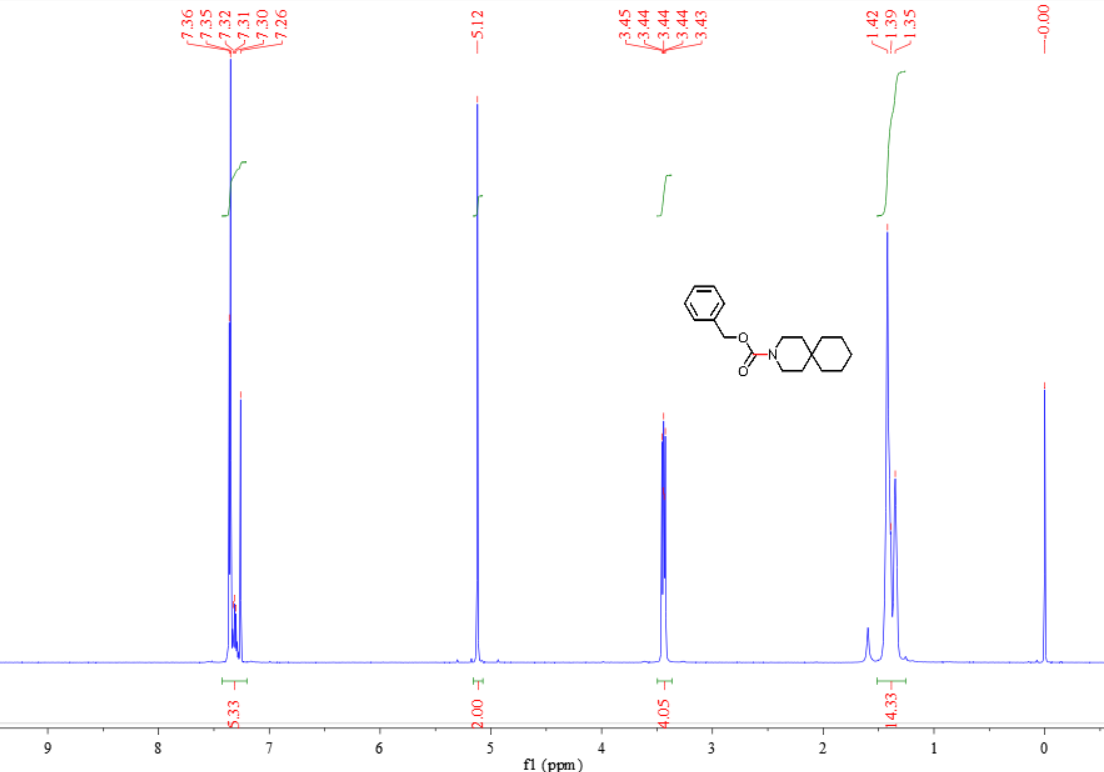


**Compound** **1ue** ^13^C{^1^H}NMR (100 MHz, CDCl_3_)


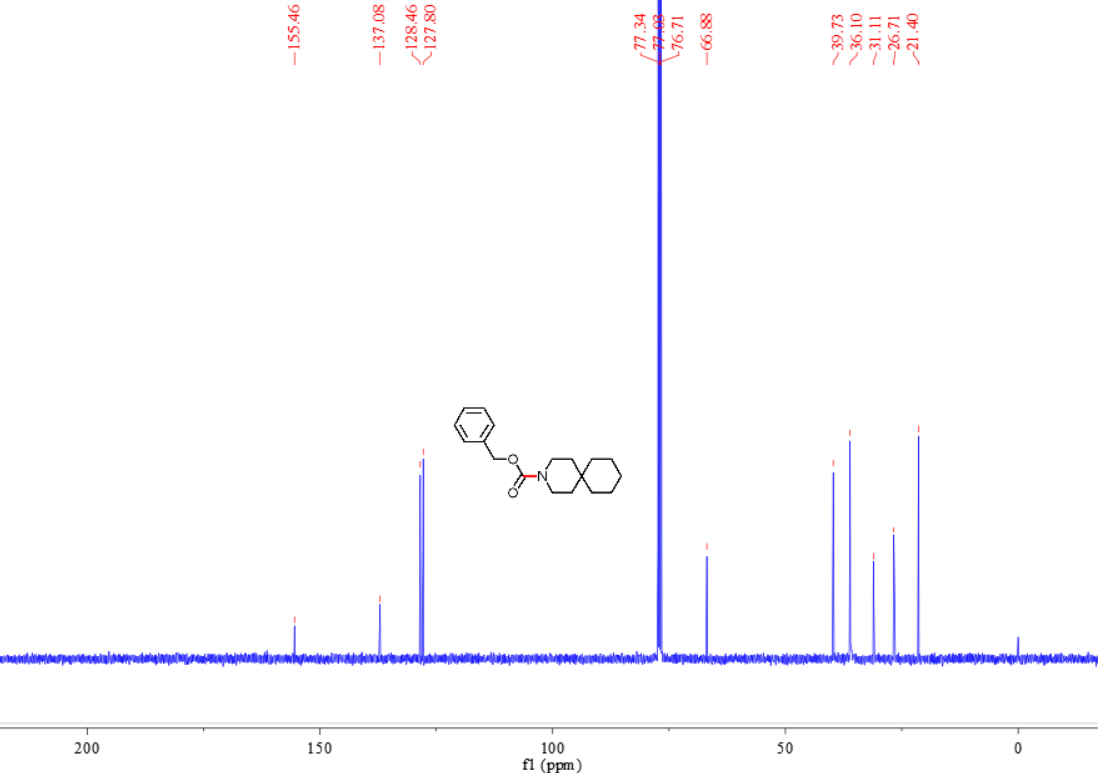


**Compound** **1w** ^1^H NMR (400 MHz, CDCl_3_)


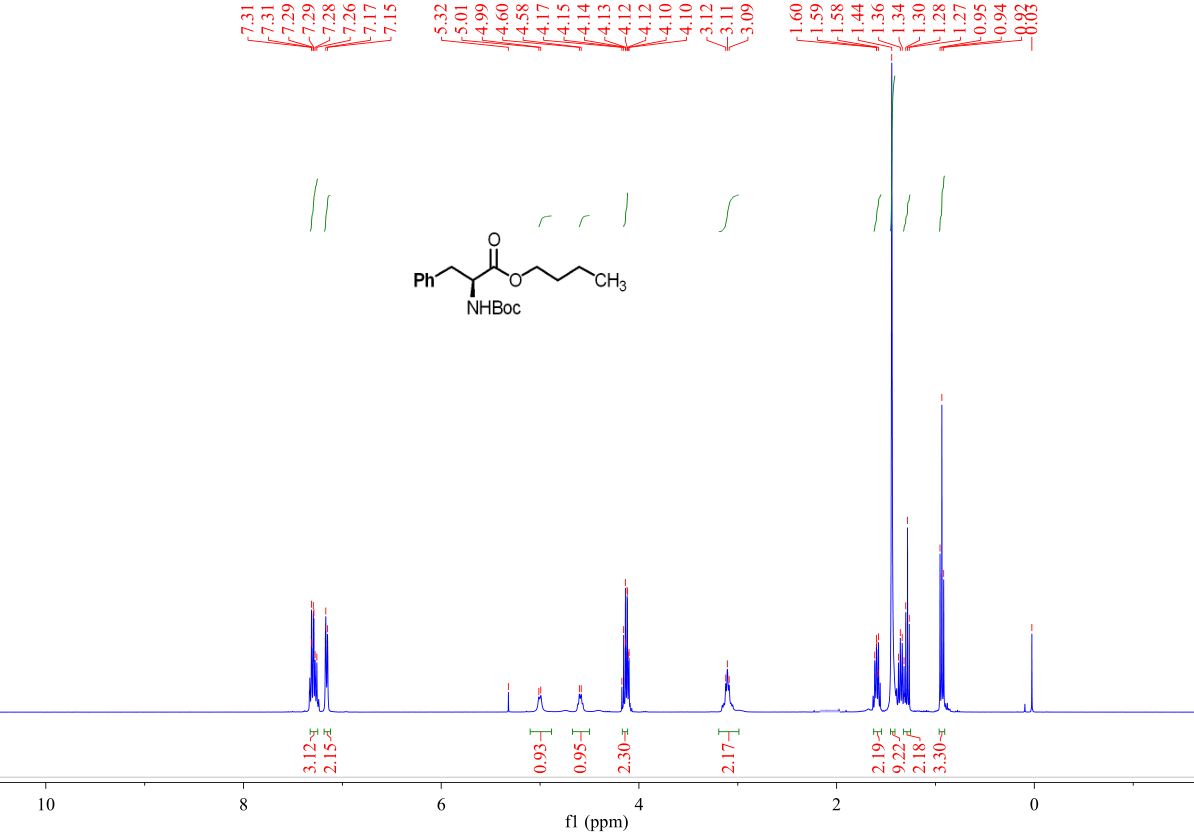


**Compound** **1w** ^13^C{^1^H}NMR (100 MHz, CDCl_3_)


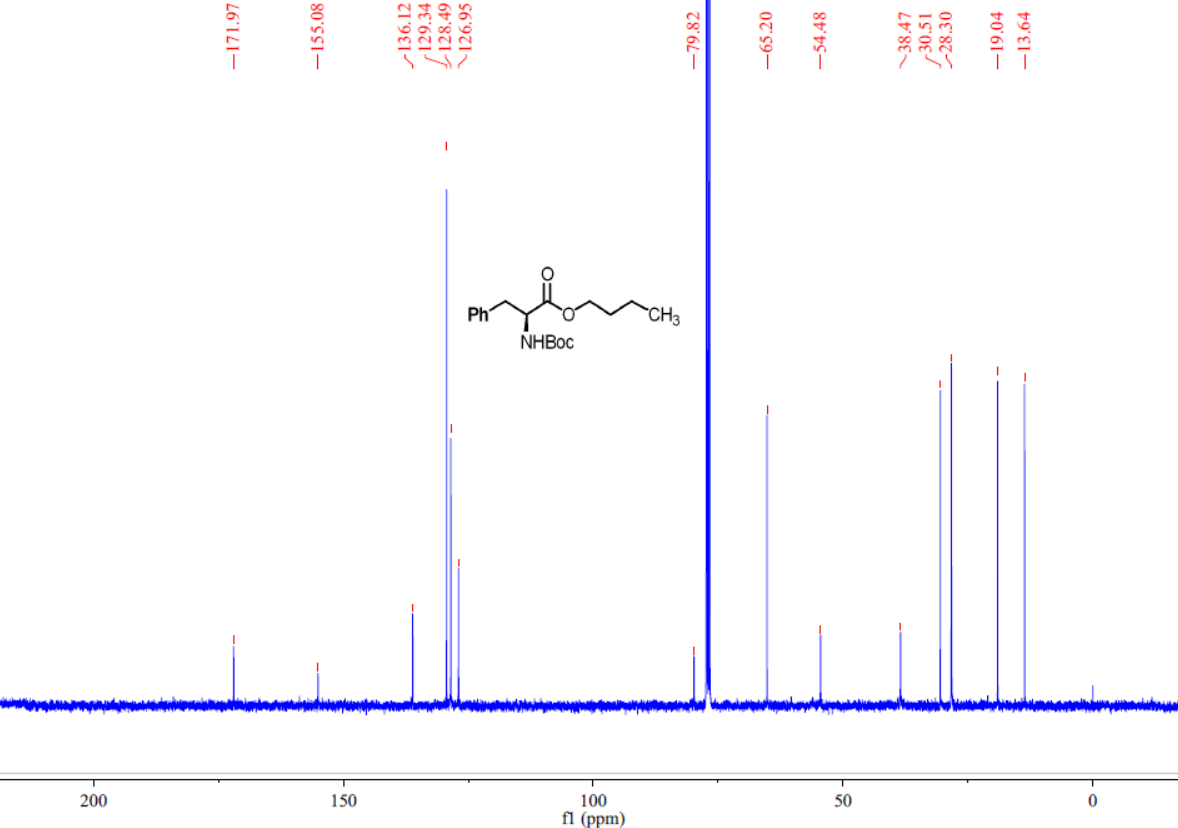


**Compound** **1wb** ^1^H NMR (400 MHz, CDCl_3_)


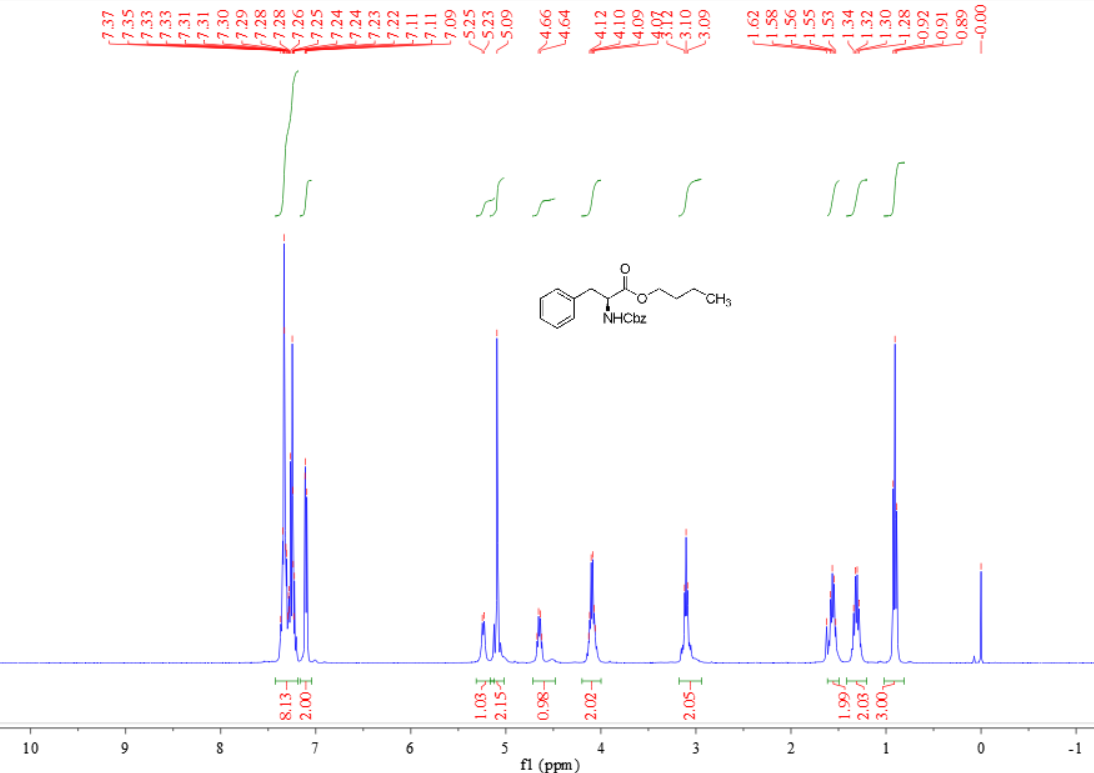


**Compound** **1wb** ^13^C{^1^H}NMR (100 MHz, CDCl_3_)


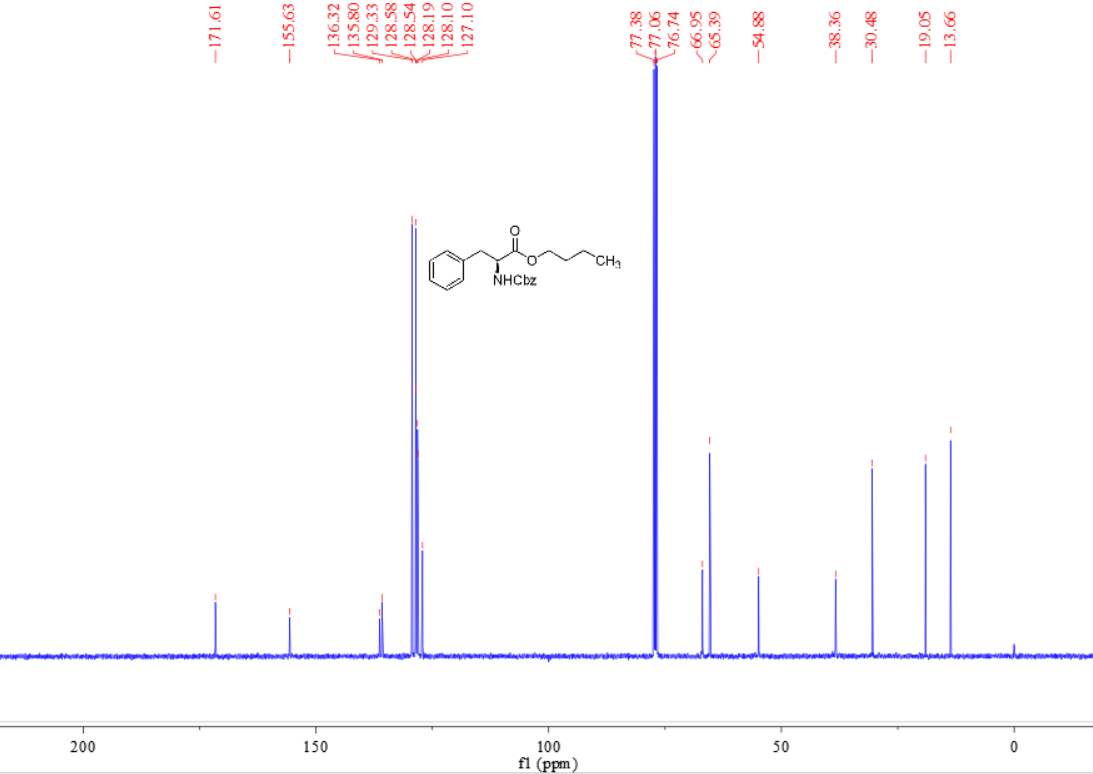


**Compound** **1x** ^1^H NMR (400 MHz, CDCl_3_)


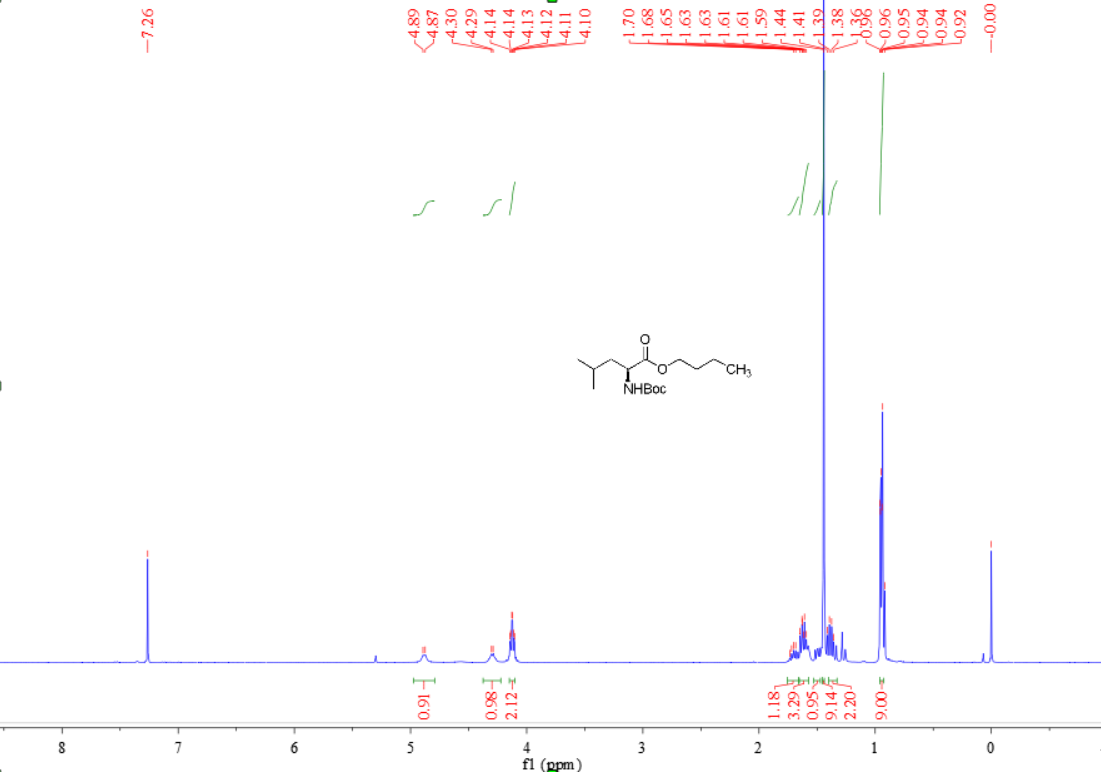


**Compound** **1x** ^13^C{^1^H}NMR (100 MHz, CDCl_3_)


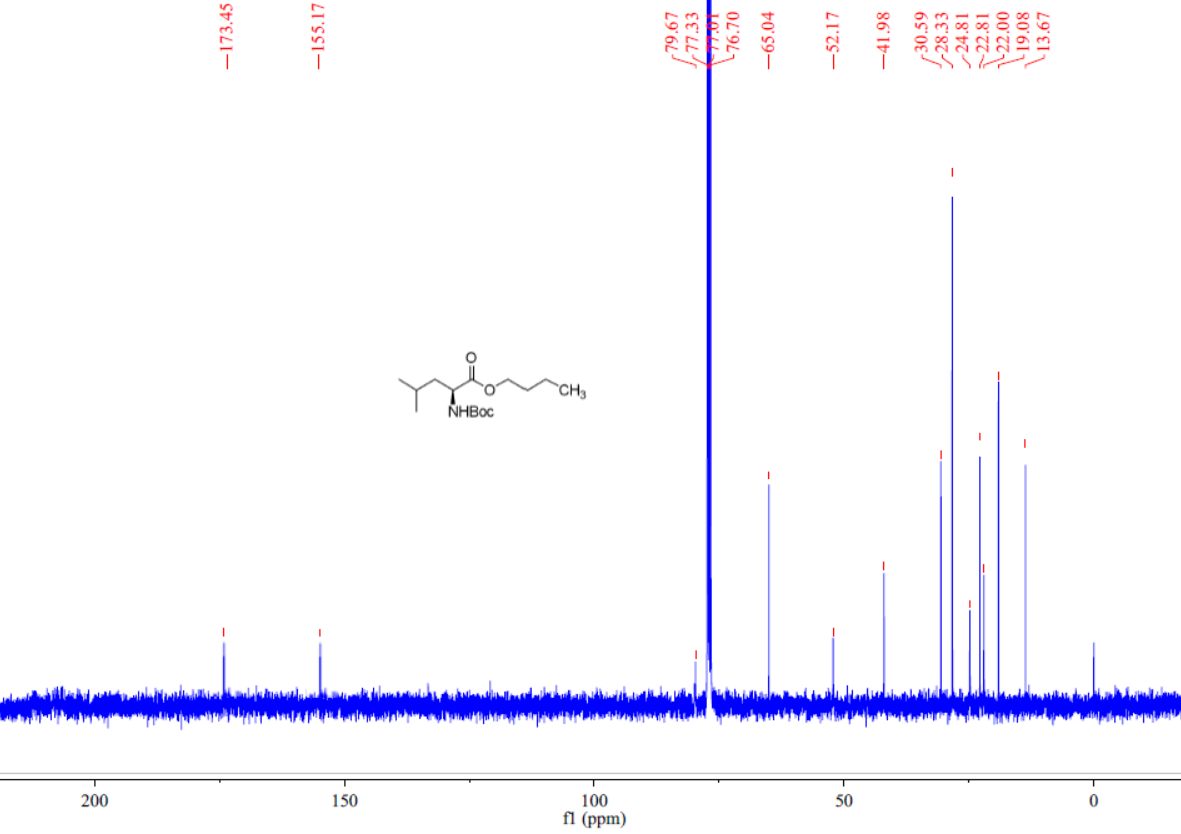


**Compound** **1xb** ^1^H NMR (400 MHz, CDCl_3_)


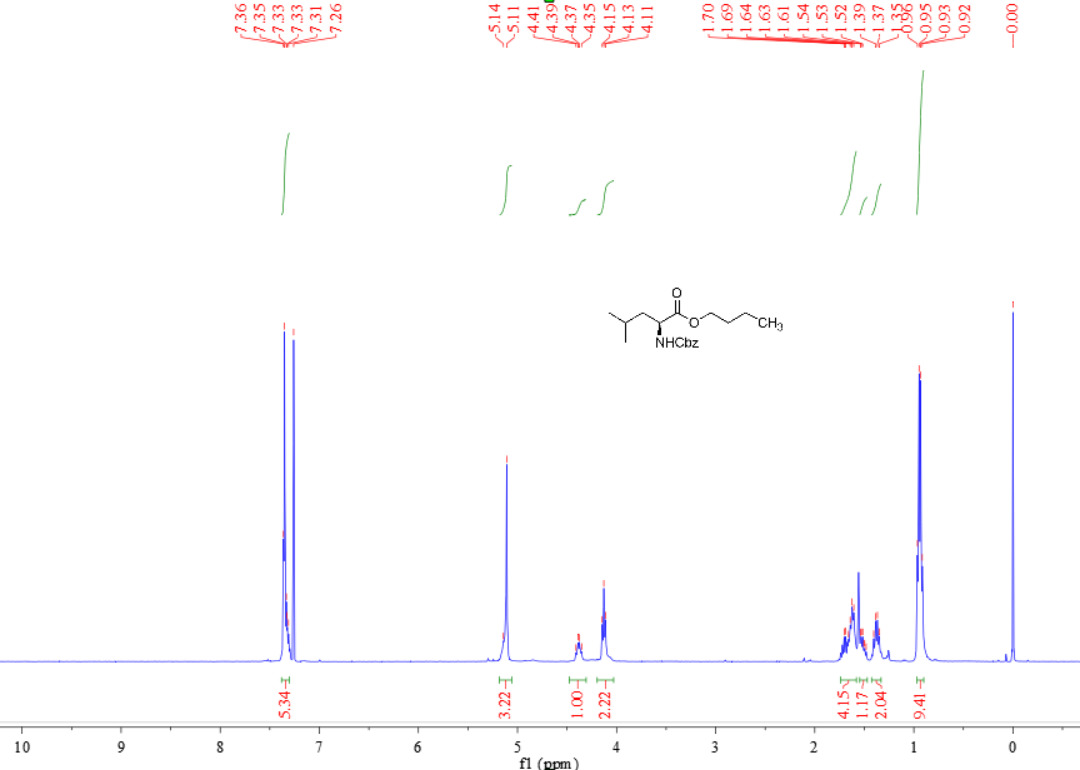


**Compound** **1xb** ^13^C{^1^H}NMR (100 MHz, CDCl_3_)


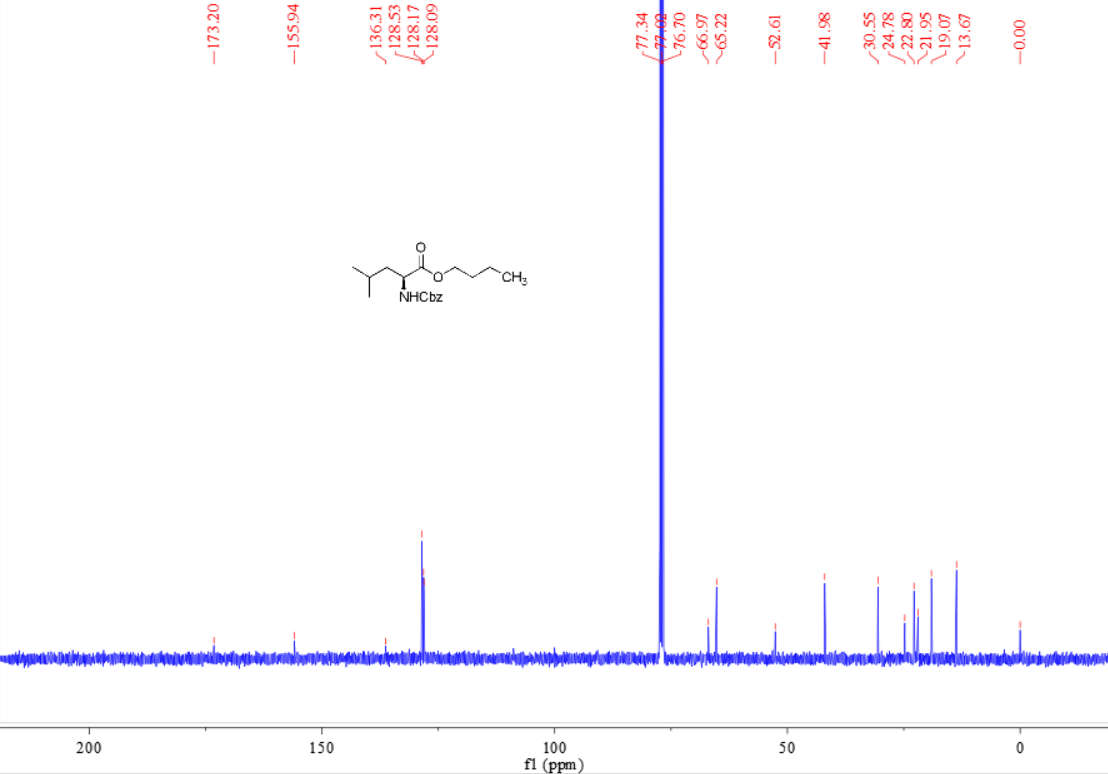


**Compound** **2a** ^1^H NMR (400 MHz, CDCl_3_)


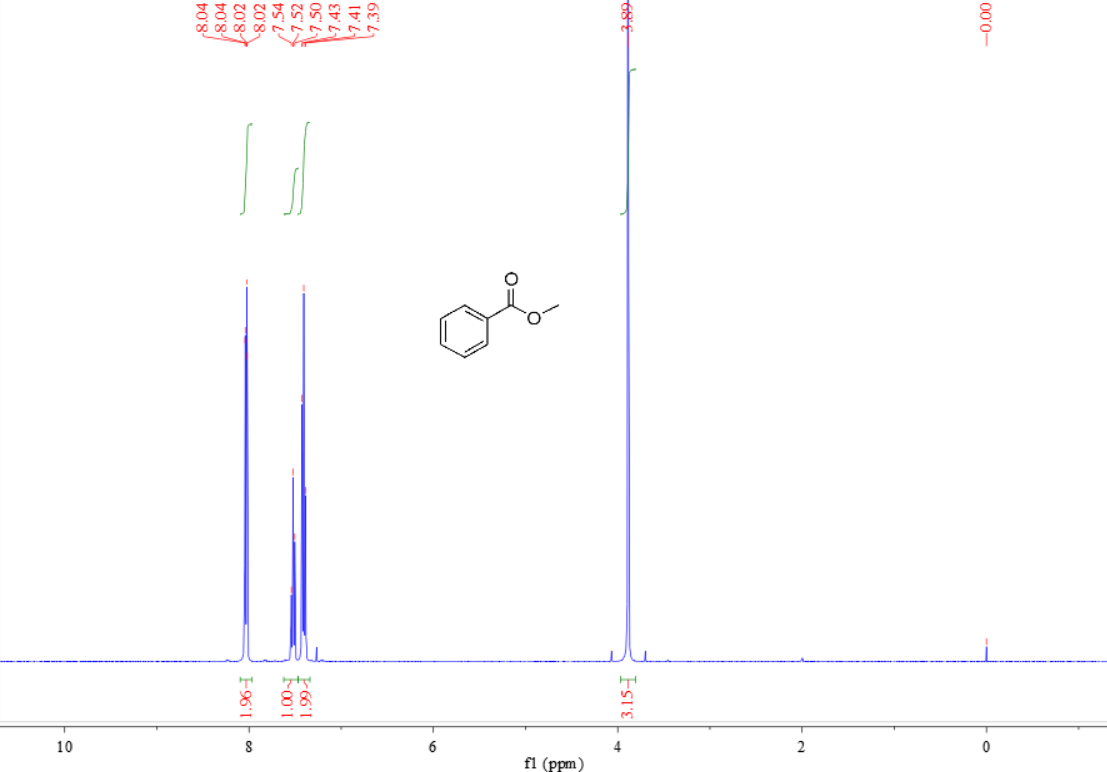


**Compound** **2a** ^13^C{^1^H}NMR (100 MHz, CDCl_3_)


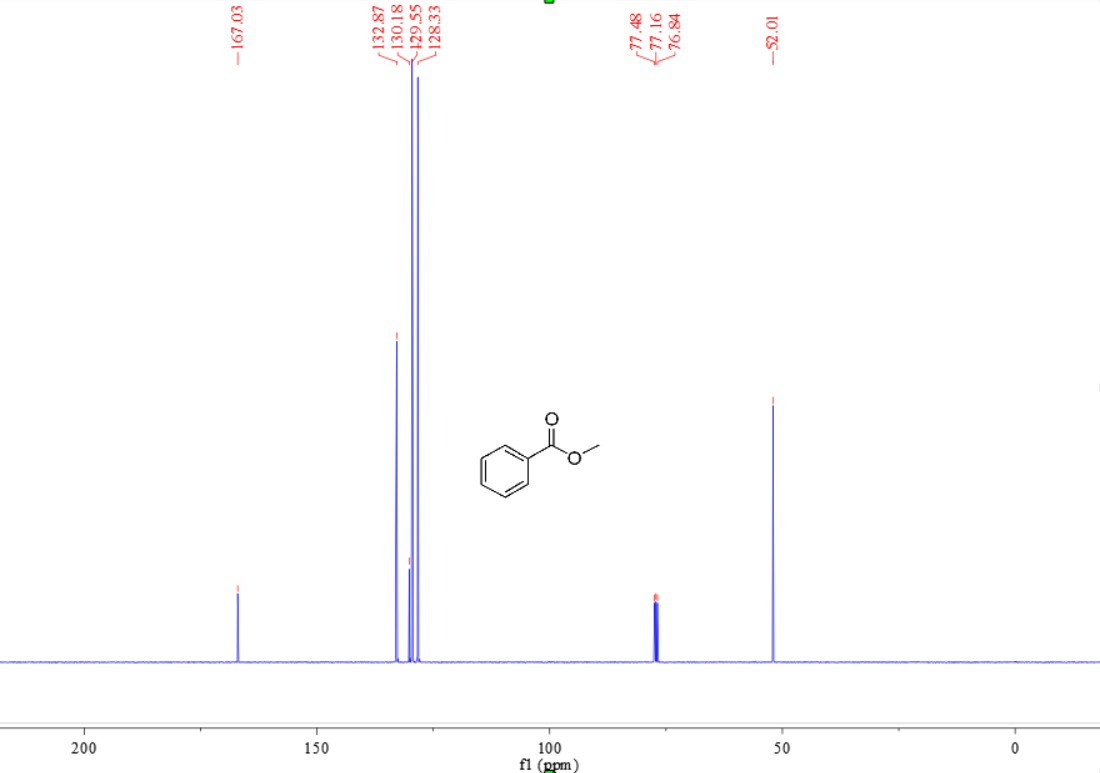


**Compound** **2ab** ^1^H NMR (400 MHz, CDCl_3_)


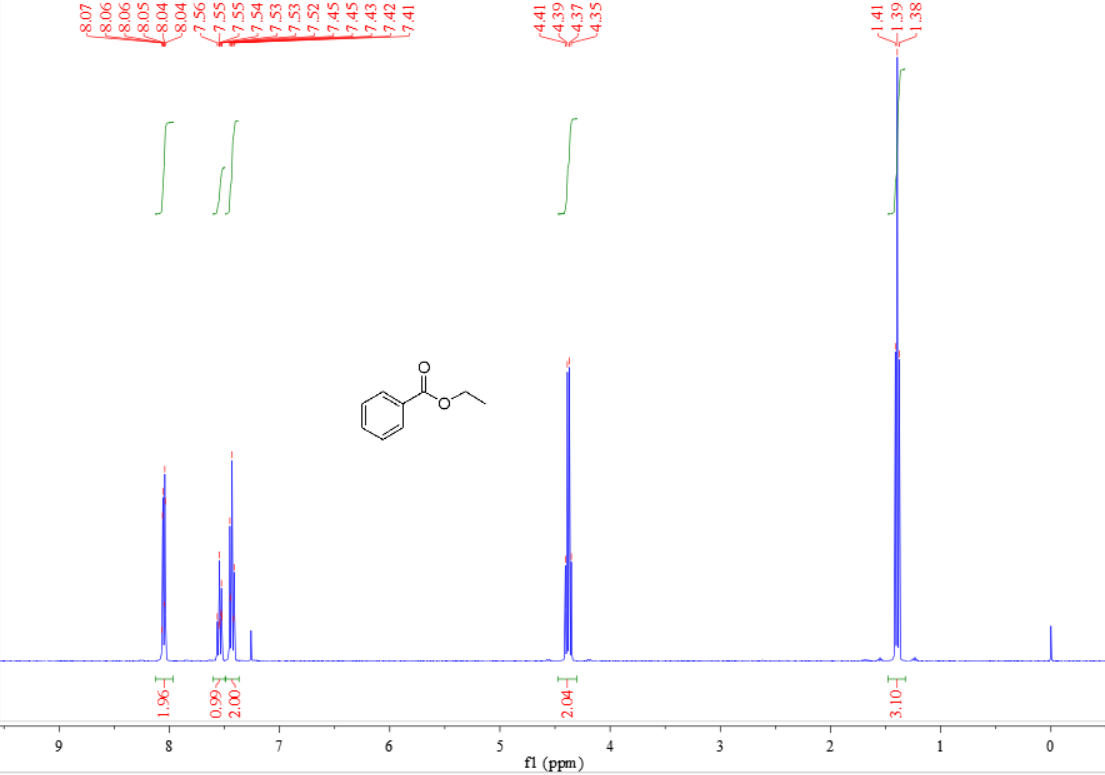


**Compound** **2ab** ^13^C{^1^H}NMR (100 MHz, CDCl_3_)


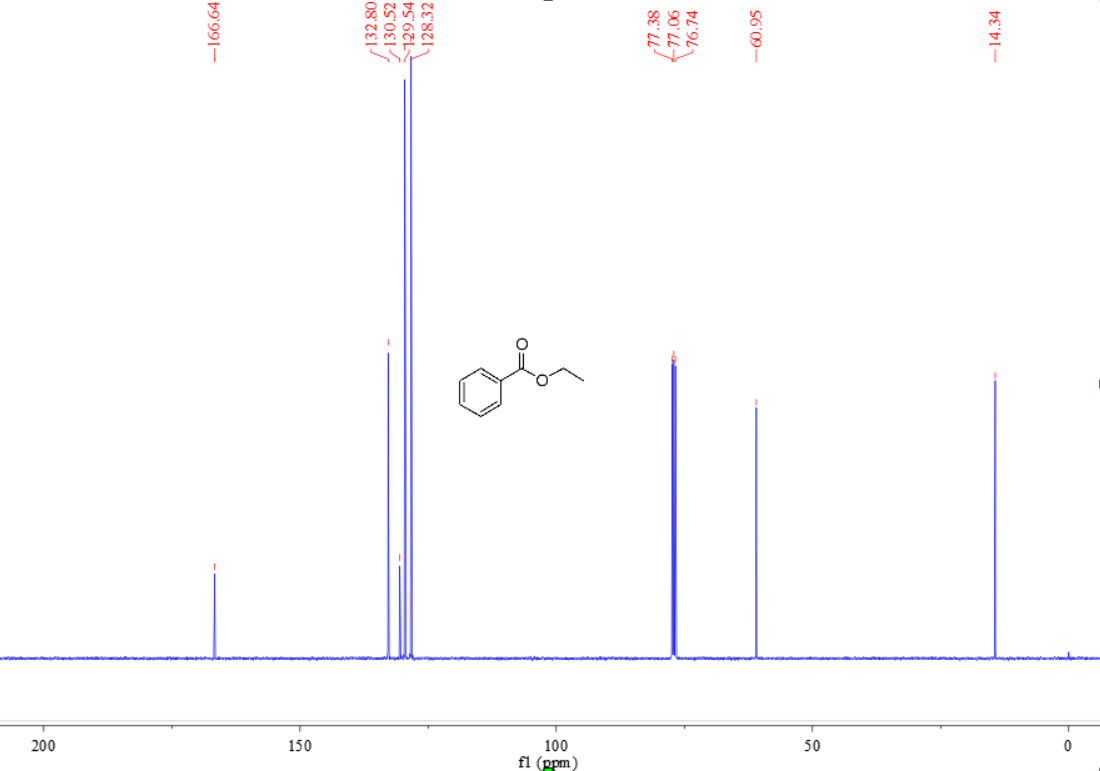


**Compound** **2ac**  ^1^H NMR (400 MHz, CDCl_3_)


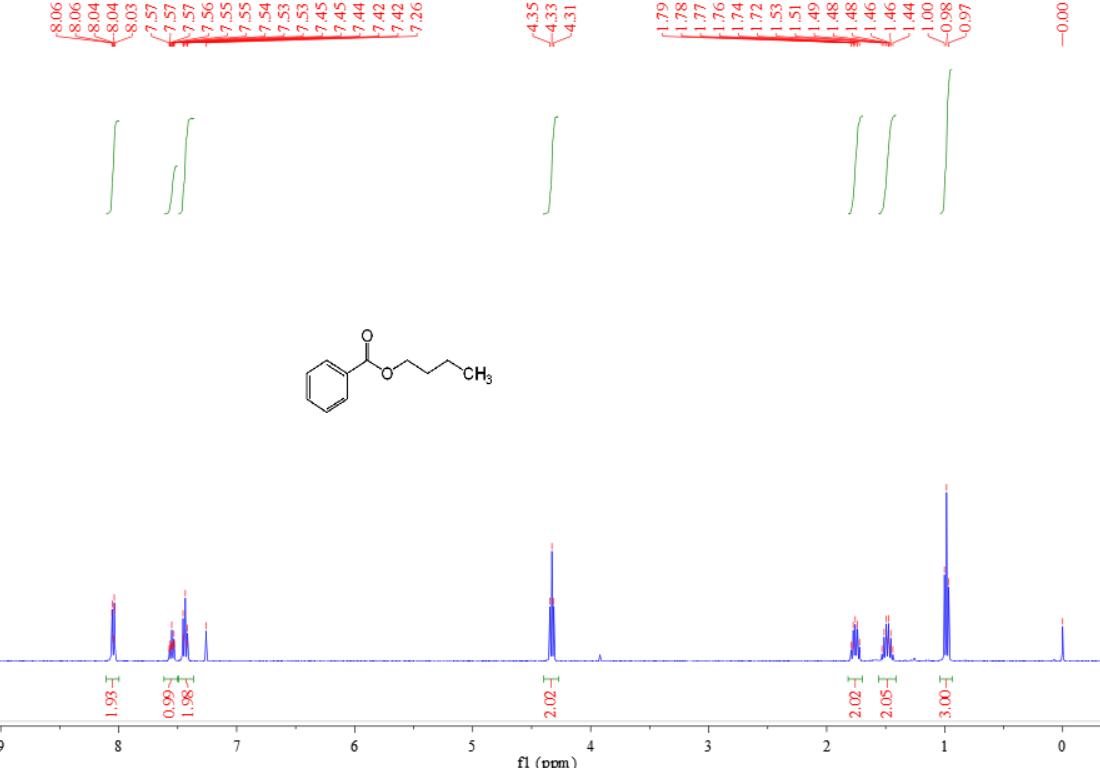


**Compound** **2ac** ^13^C{^1^H}NMR (100 MHz, CDCl_3_)


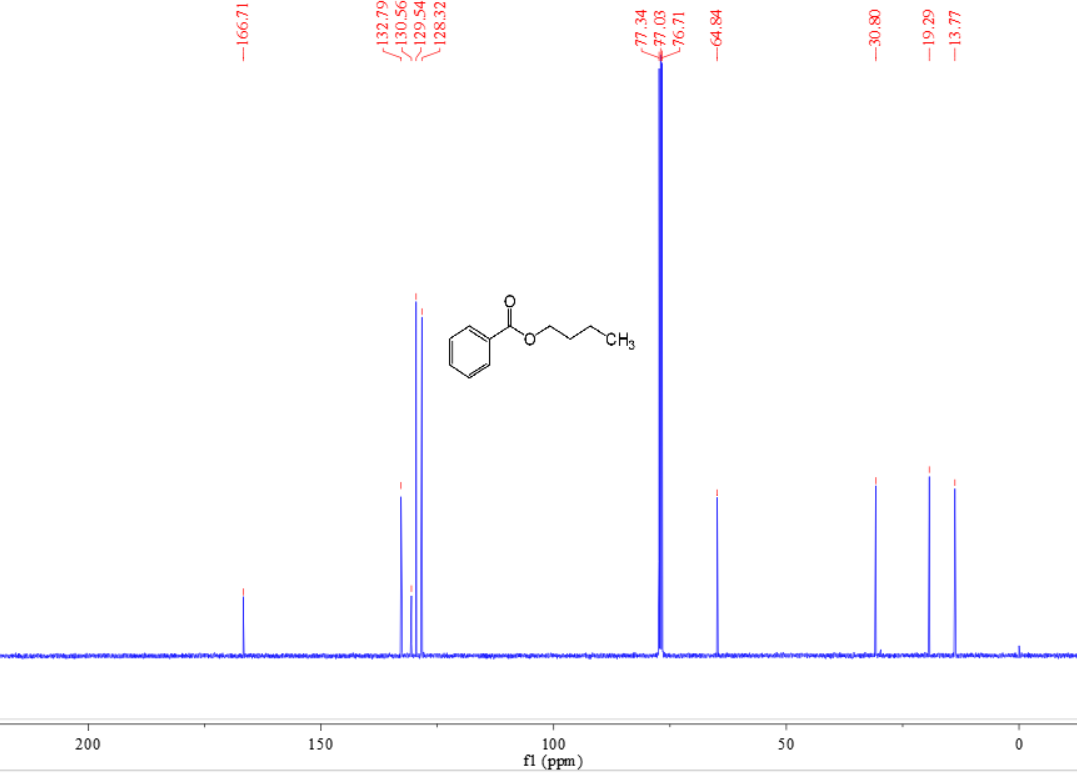


**Compound 2ad** ^1^H NMR (400 MHz, CDCl_3_)


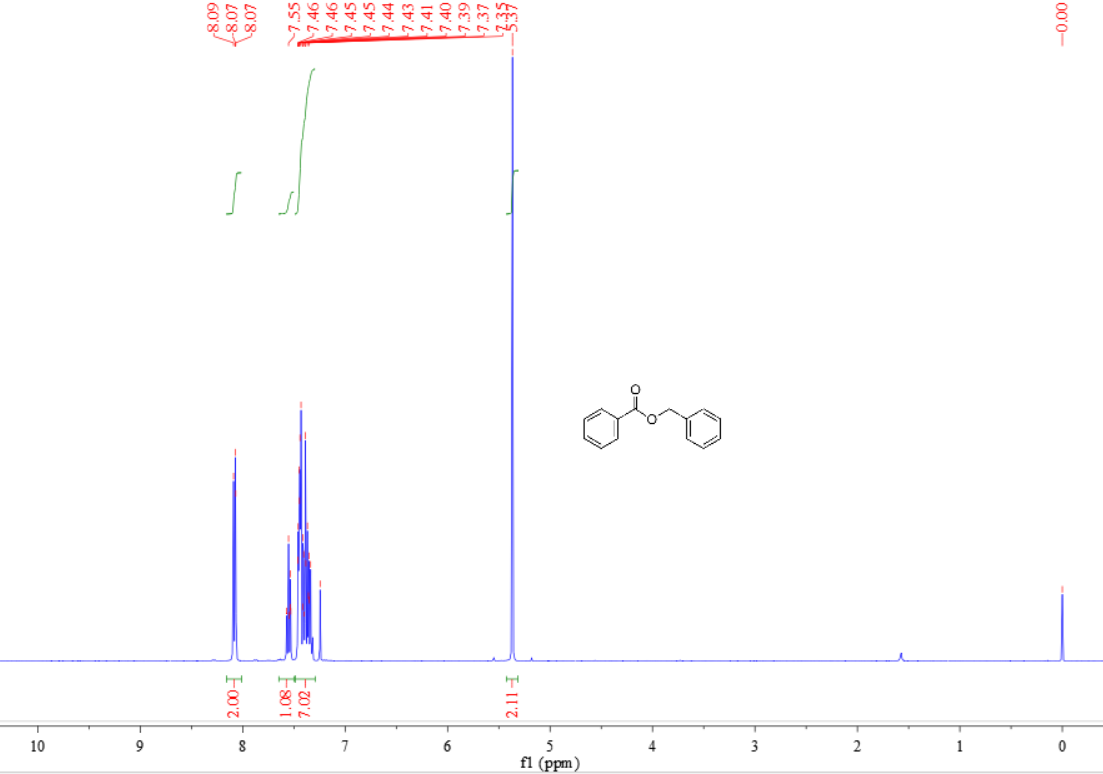


**Compound** **2ad** ^13^C{^1^H}NMR (100 MHz, CDCl_3_)


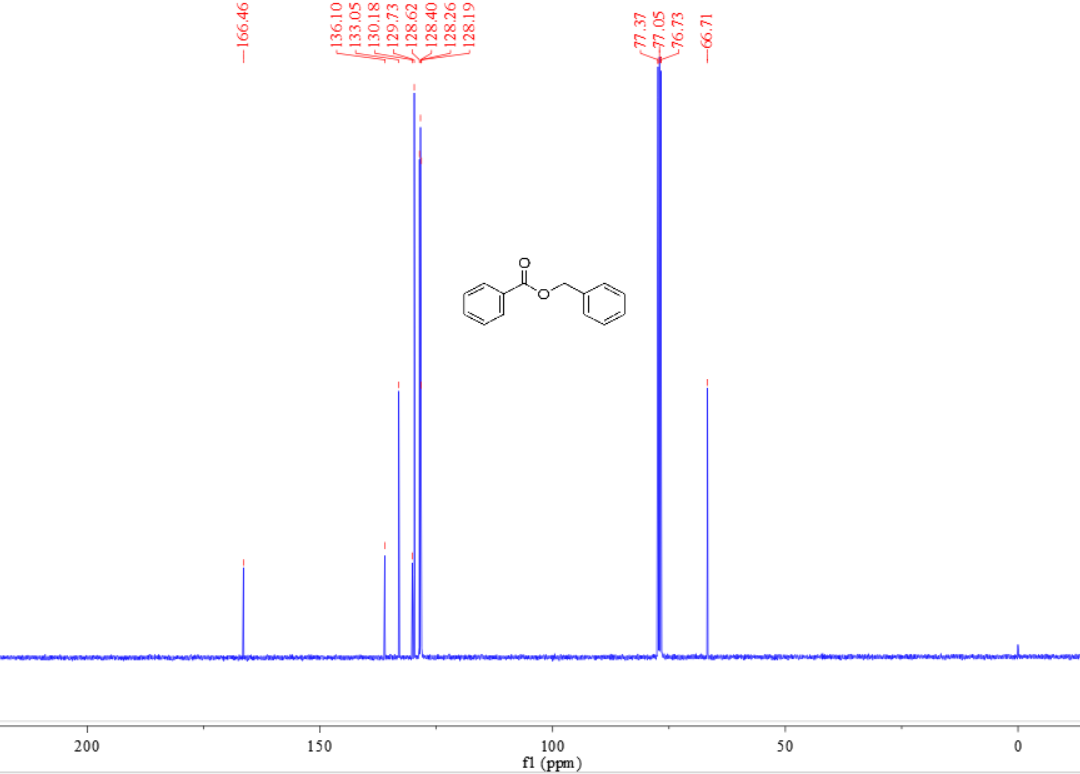


**Compound** **2ae** ^1^H NMR (400 MHz, CDCl_3_)


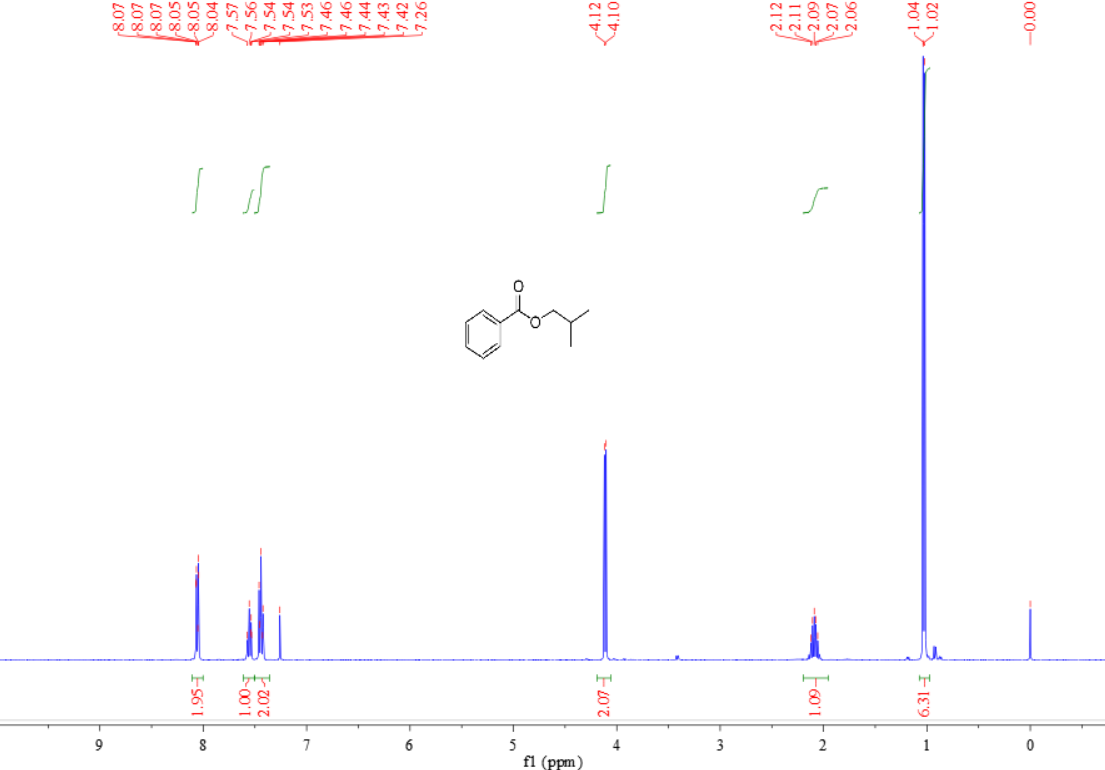


**Compound** **2ae** ^13^C{^1^H}NMR (100 MHz, CDCl_3_)


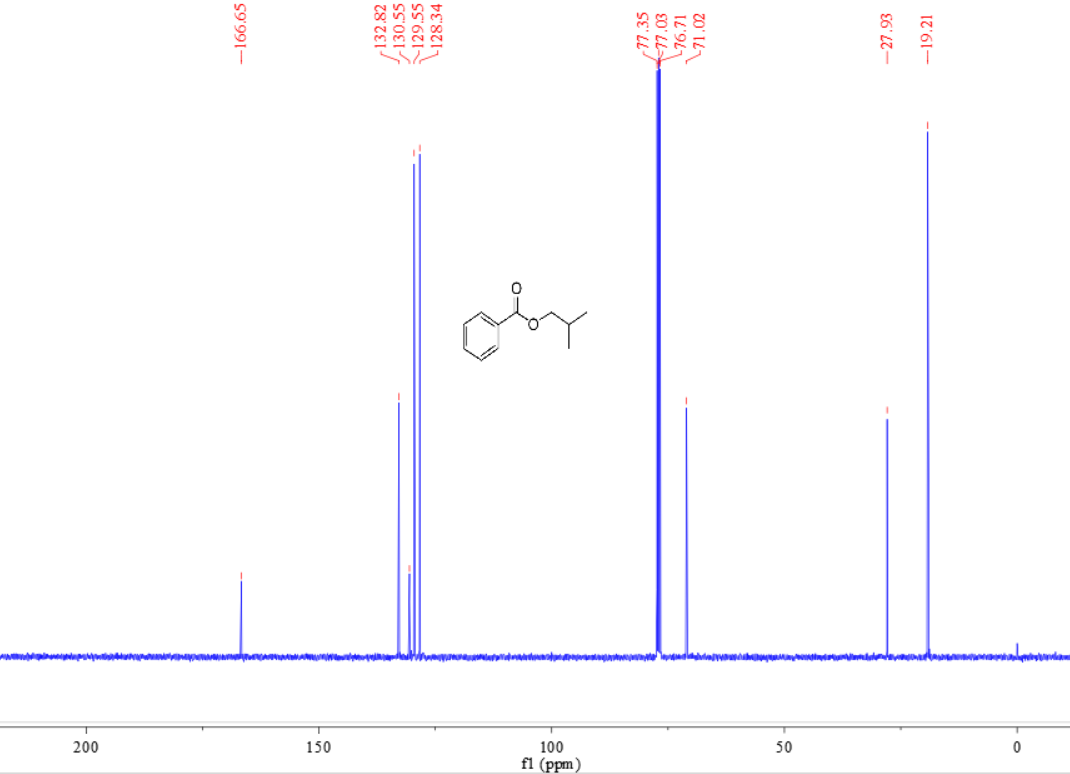


**Compound** **2af** ^1^H NMR (400 MHz, CDCl_3_)


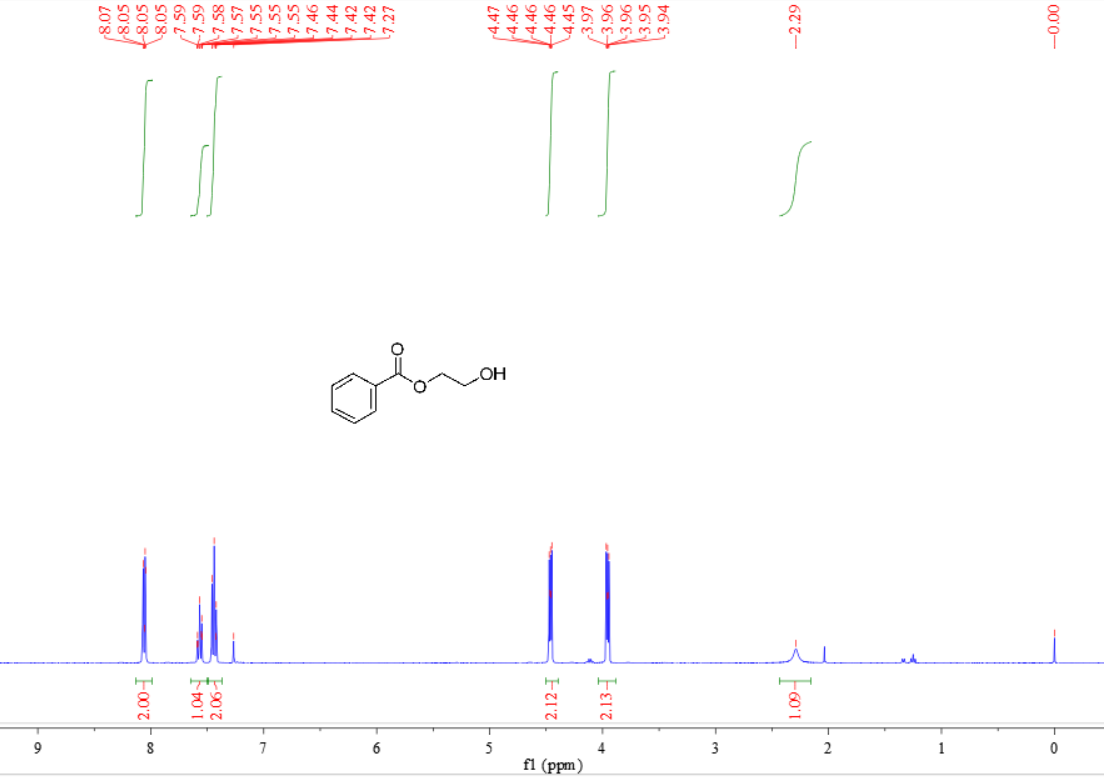


**Compound** **2af** ^13^C{^1^H}NMR (100 MHz, CDCl_3_)


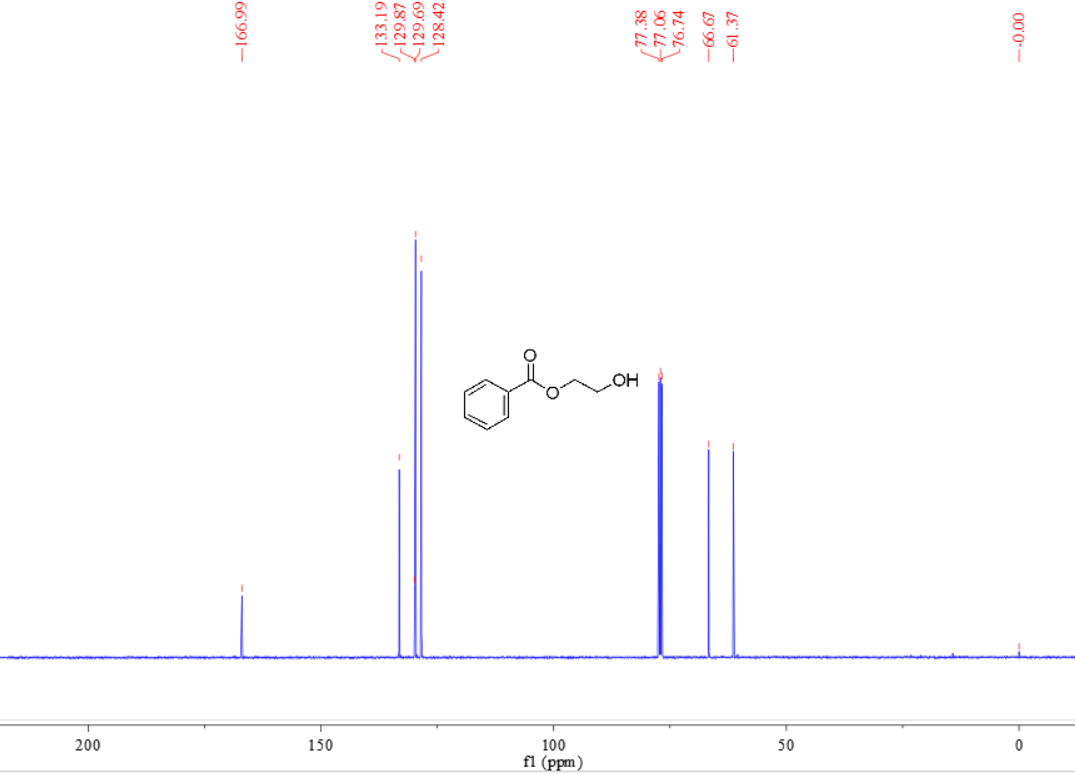


**Compound** **2ag** ^1^H NMR (400 MHz, CDCl_3_)


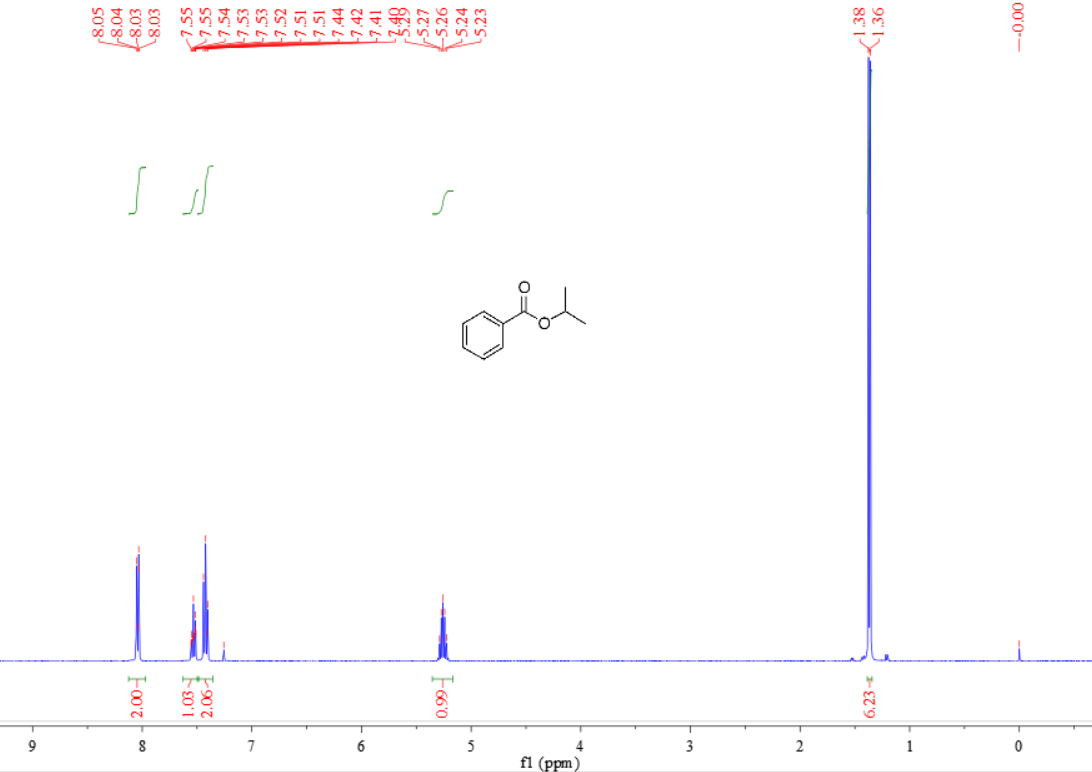


**Compound** **2ag** ^13^C{^1^H}NMR (100 MHz, CDCl_3_)


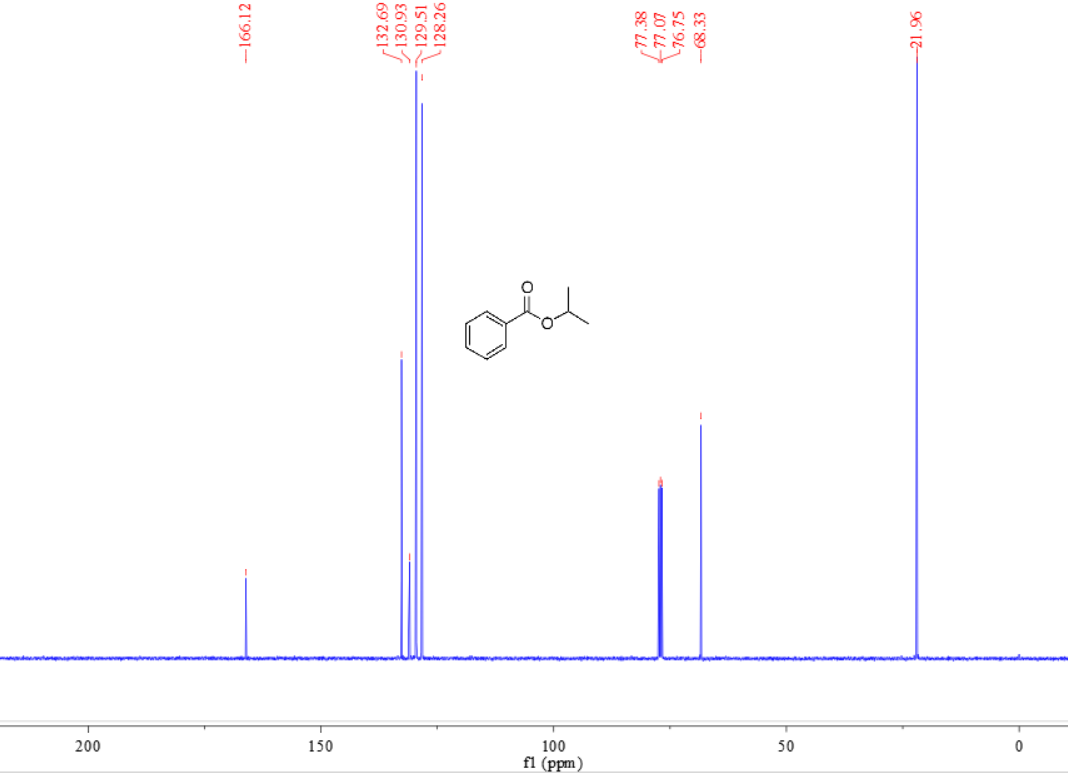


**Compound** **2ah** ^1^H NMR (400 MHz, CDCl_3_)


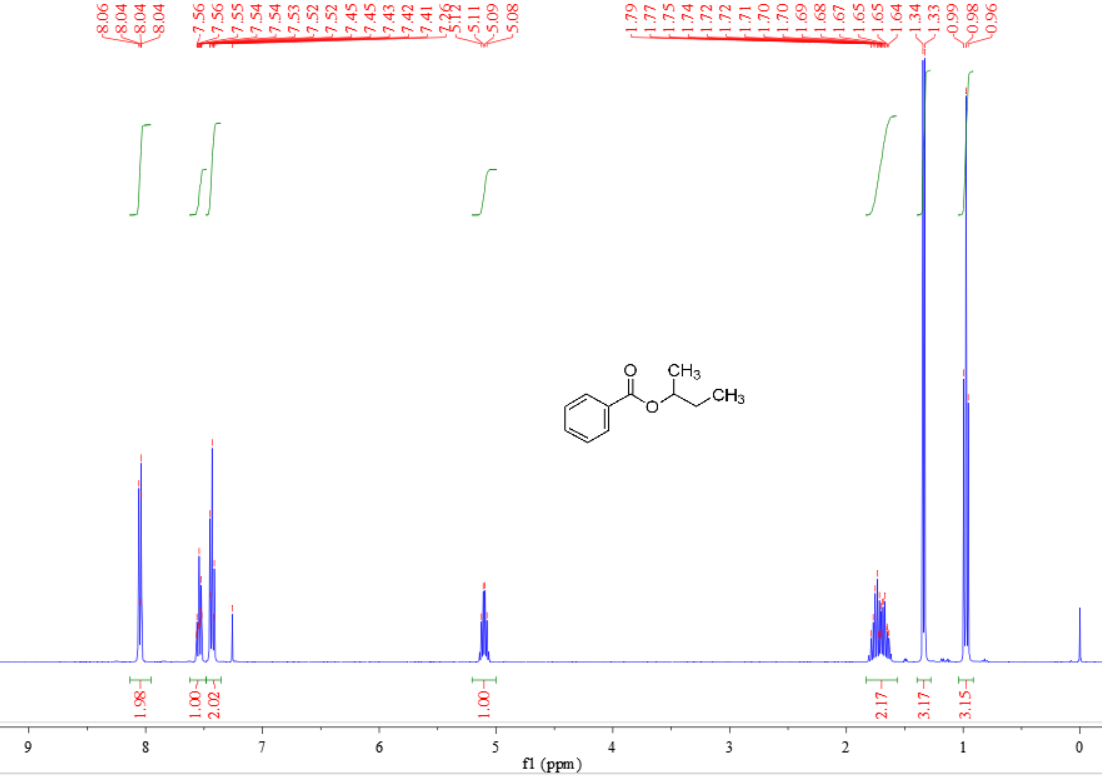


**Compound** **2ah** ^13^C{^1^H}NMR (100 MHz, CDCl_3_)


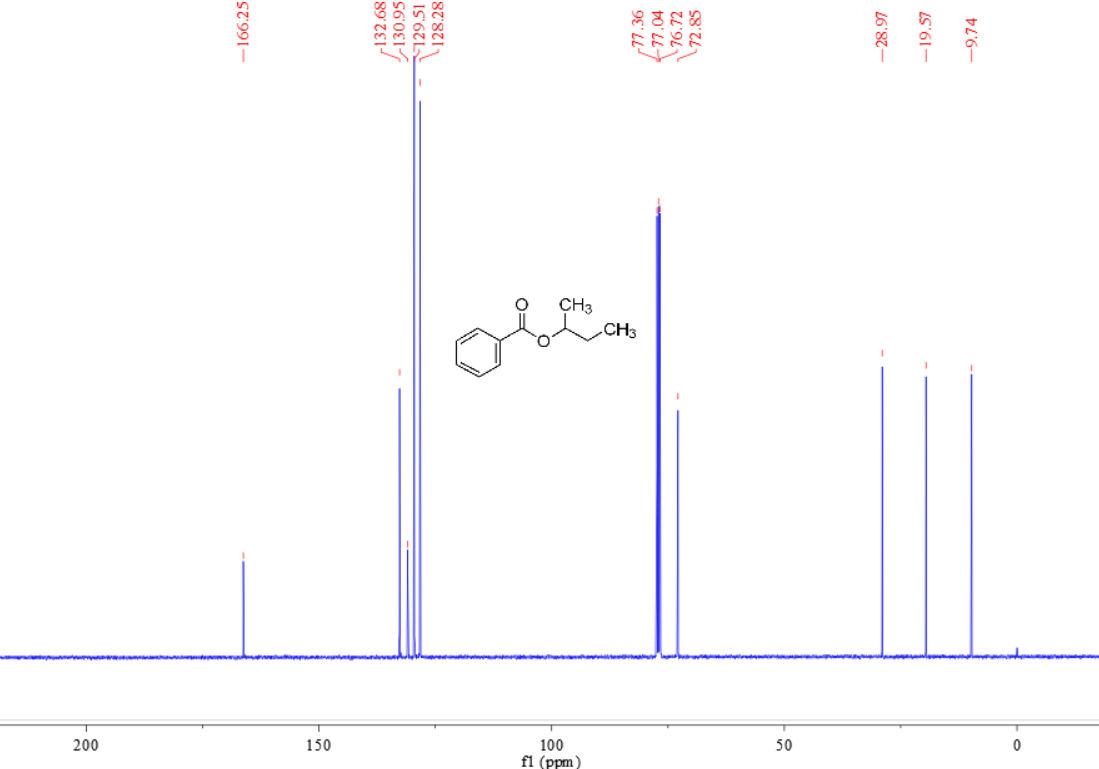


**Compound** **2ai** ^1^H NMR (400 MHz, CDCl_3_)


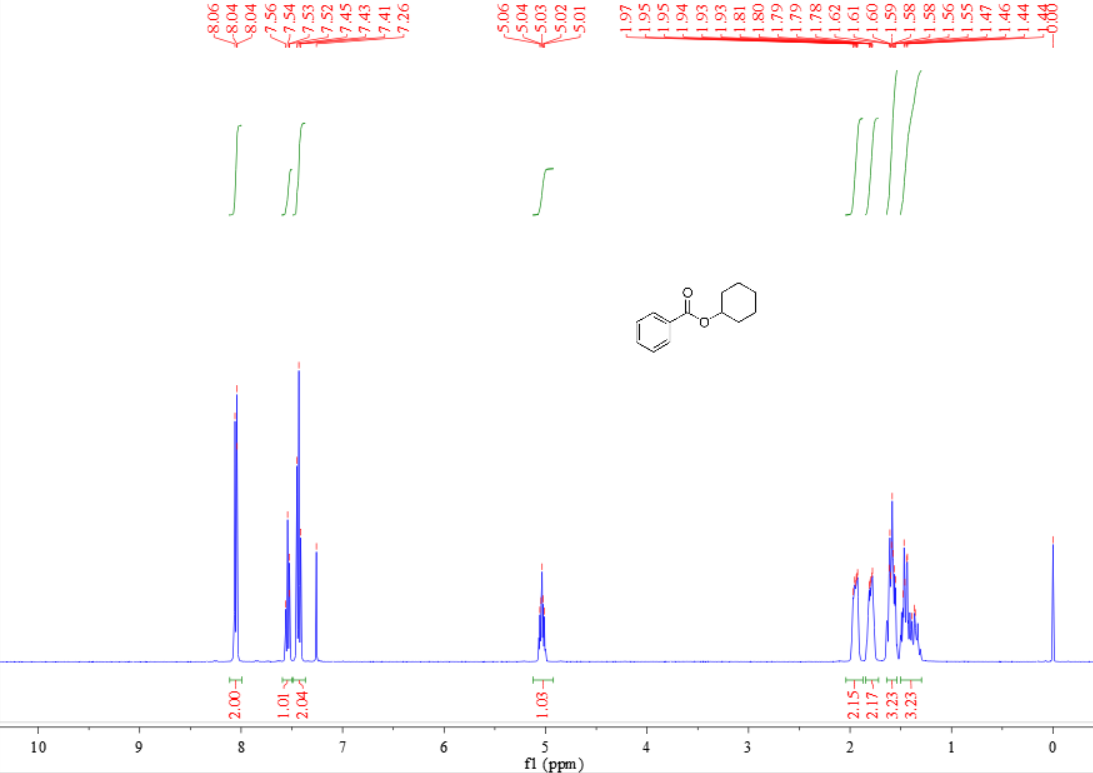


**Compound** **1g** ^13^C{^1^H}NMR (100 MHz, CDCl_3_)


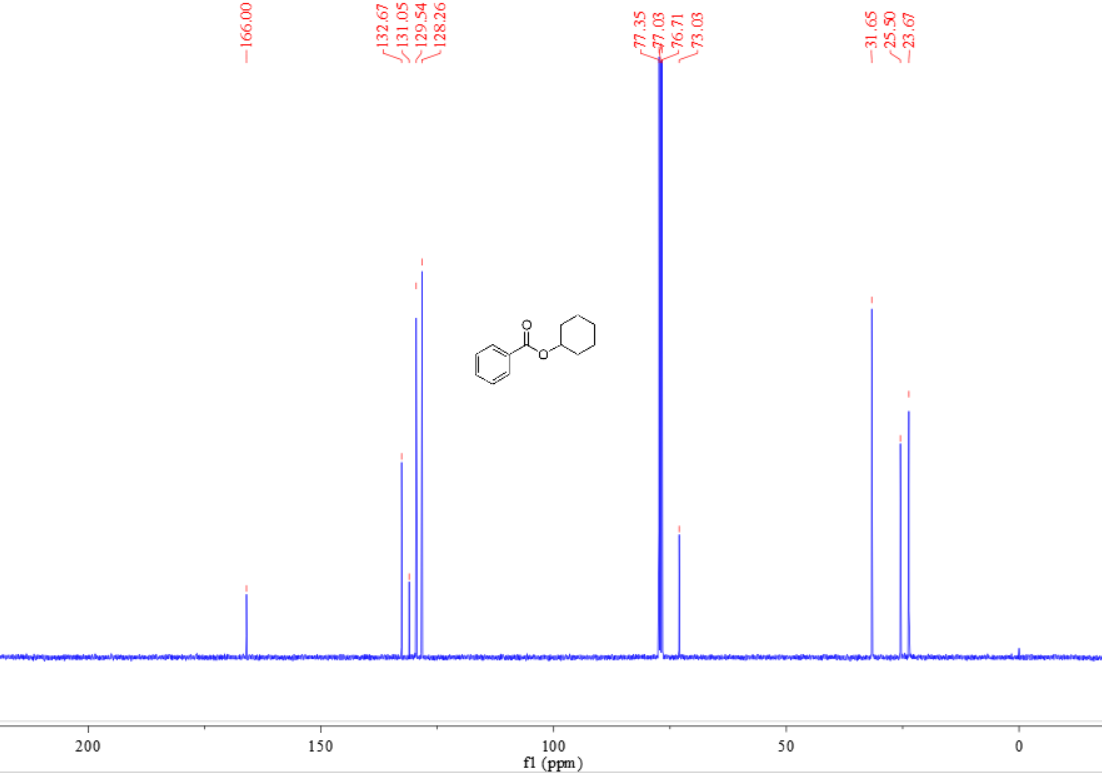


**Compound** **2aj** ^1^H NMR (400 MHz, CDCl_3_)


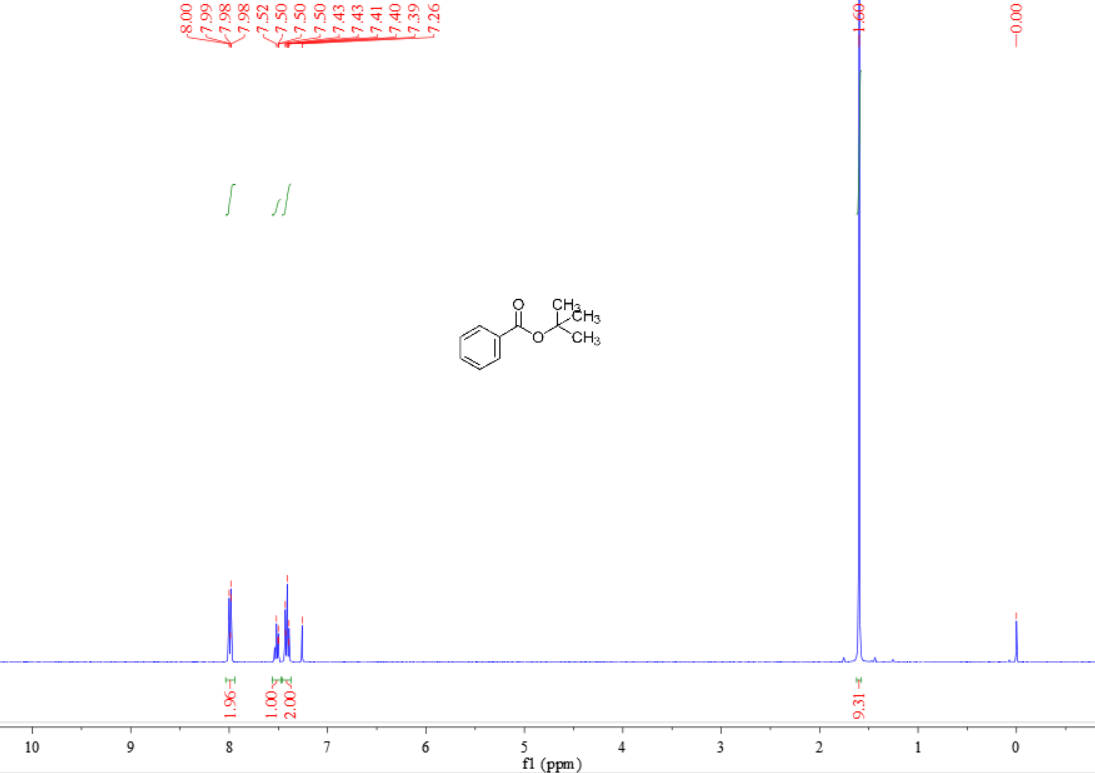


**Compound** **2aj** ^13^C{^1^H}NMR (100 MHz, CDCl_3_)


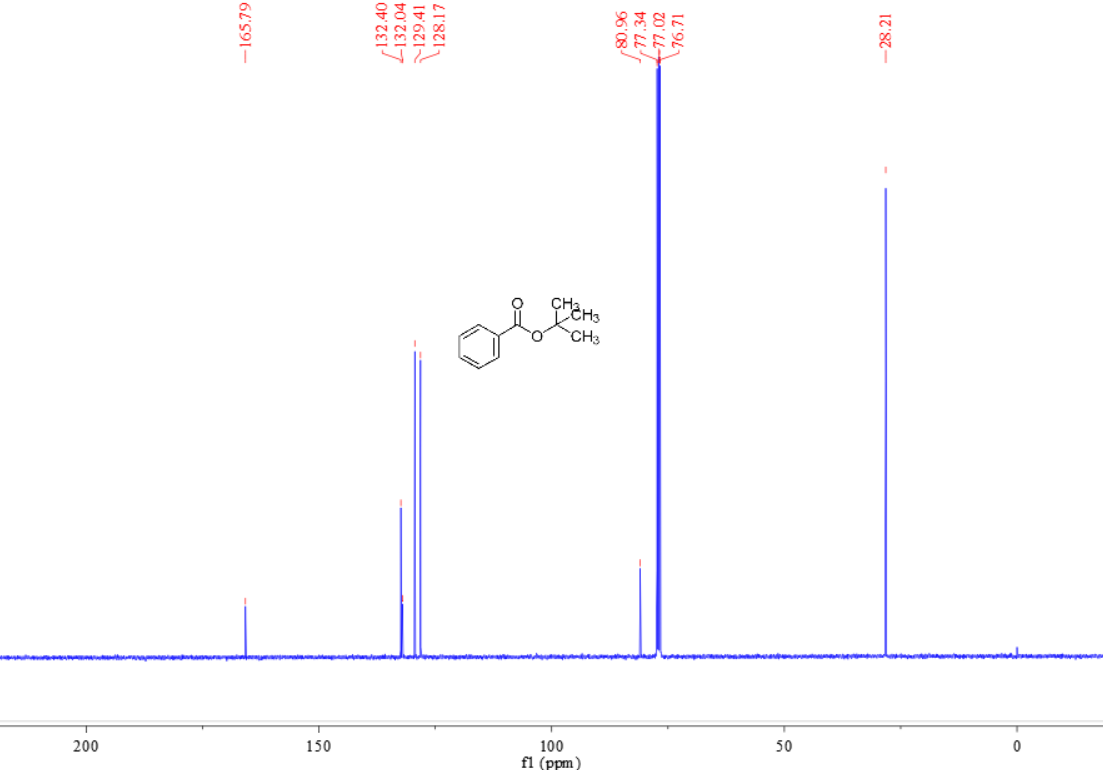


**Compound** **2b** ^1^H NMR (400 MHz, CDCl_3_)


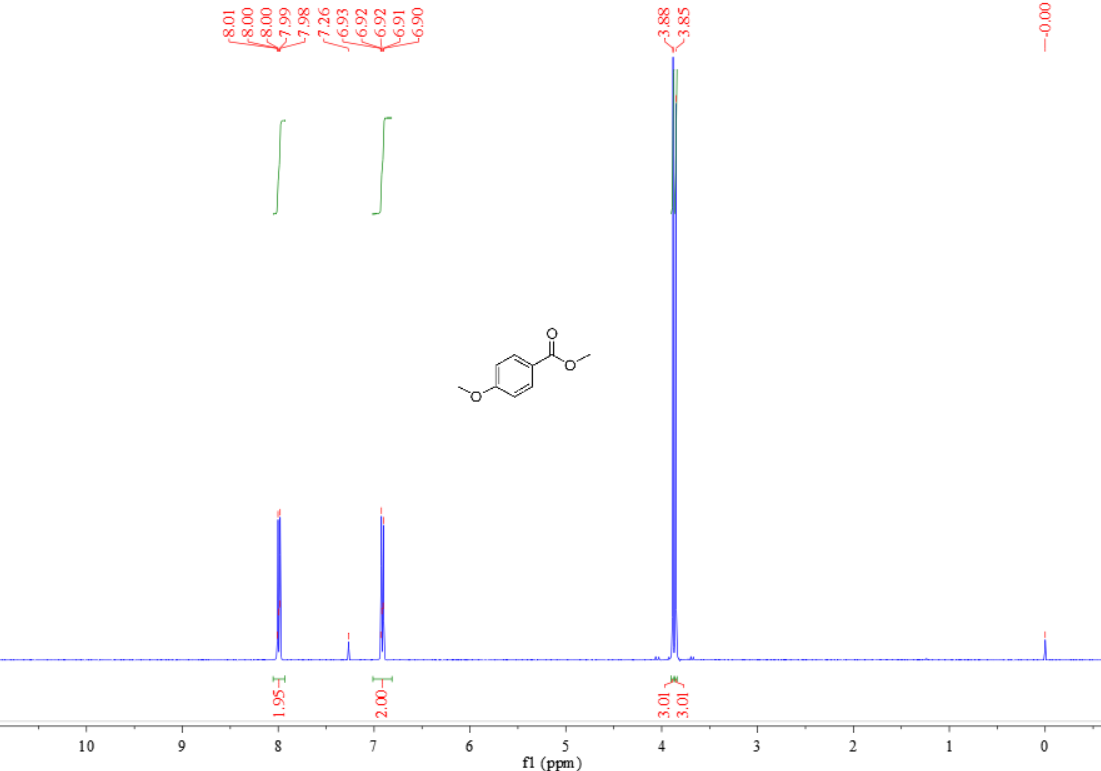


**Compound** **2b** ^13^C{^1^H}NMR (100 MHz, CDCl_3_)


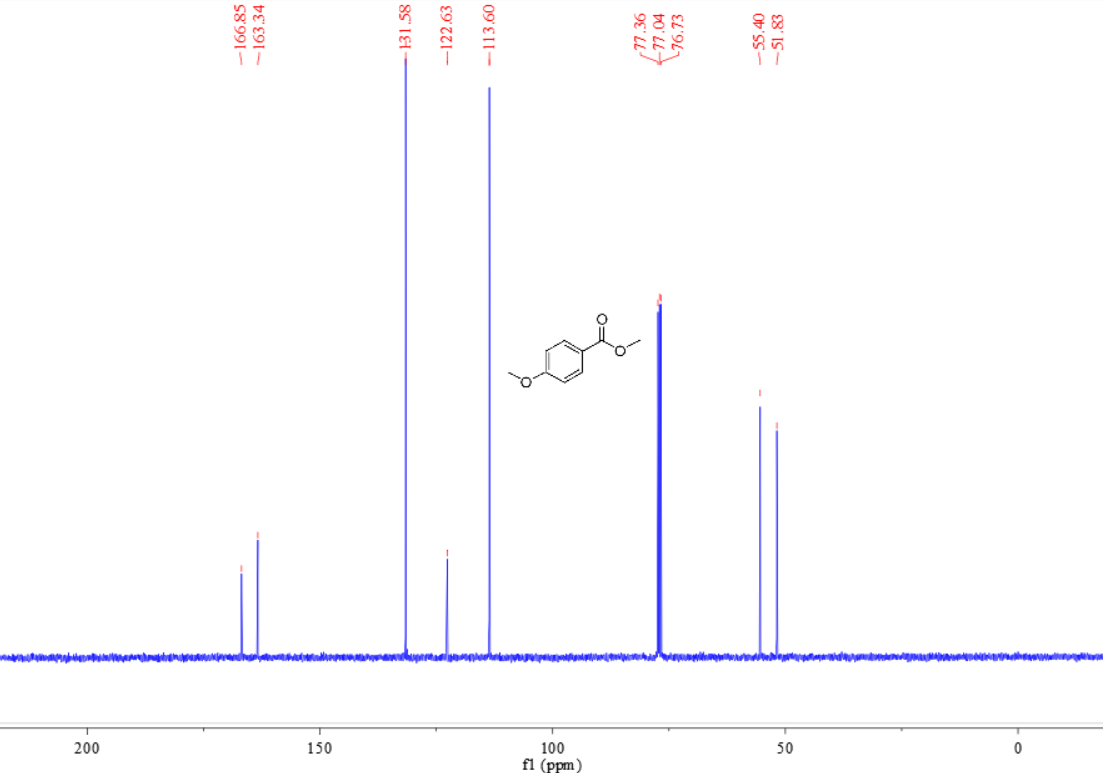


**Compound** **2bb** ^1^H NMR (400 MHz, CDCl_3_)


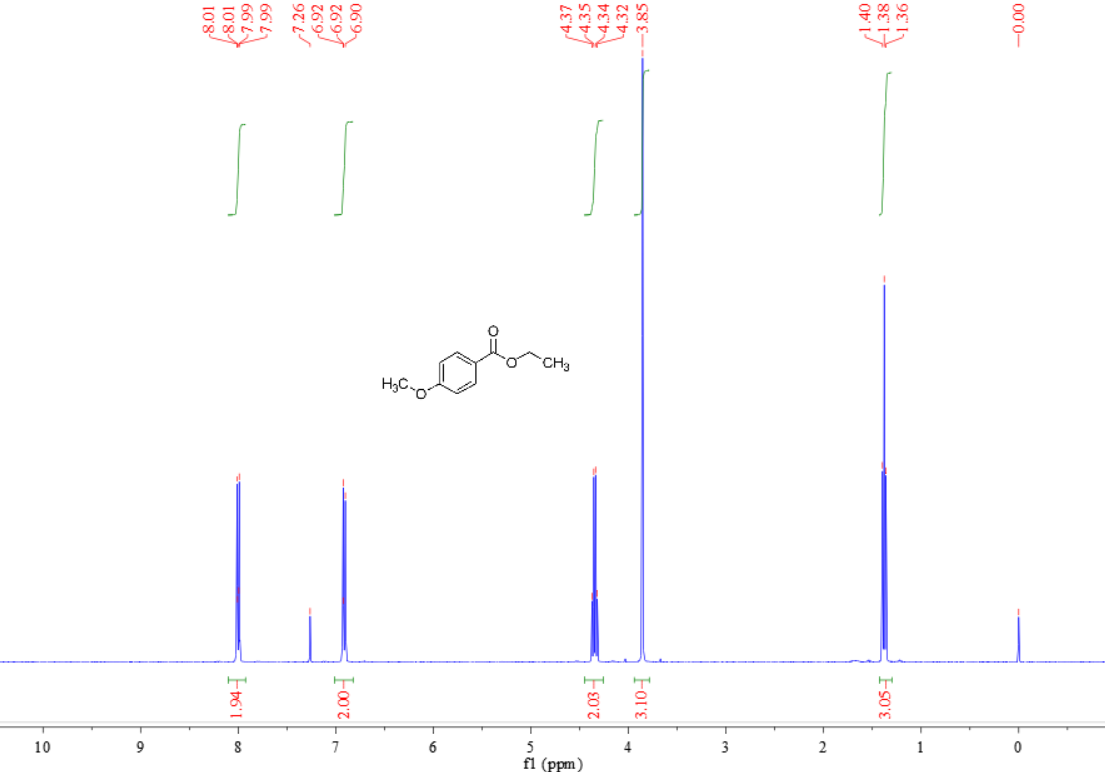


**Compound** **2bb** ^13^C{^1^H}NMR (100 MHz, CDCl_3_)


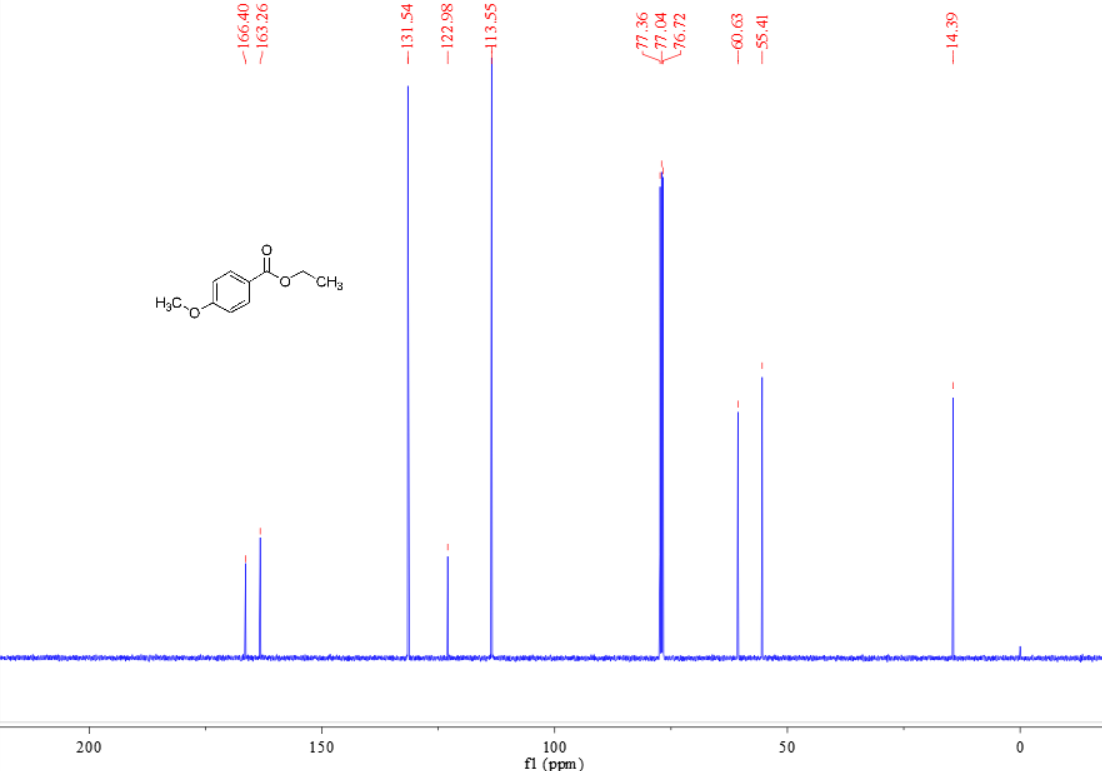


**Compound** **2bc** ^1^H NMR (400 MHz, DMSO-*d*_6_)


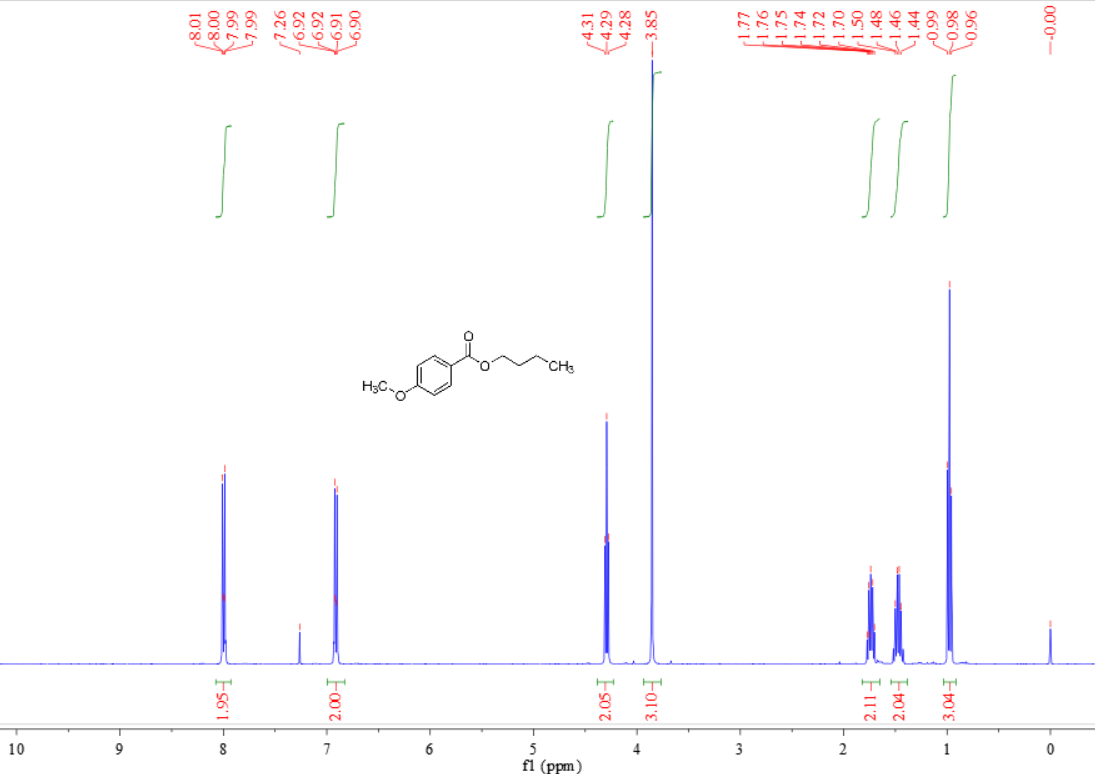


**Compound** **2bc** ^13^C{^1^H}NMR (100 MHz, DMSO-*d*_6_)


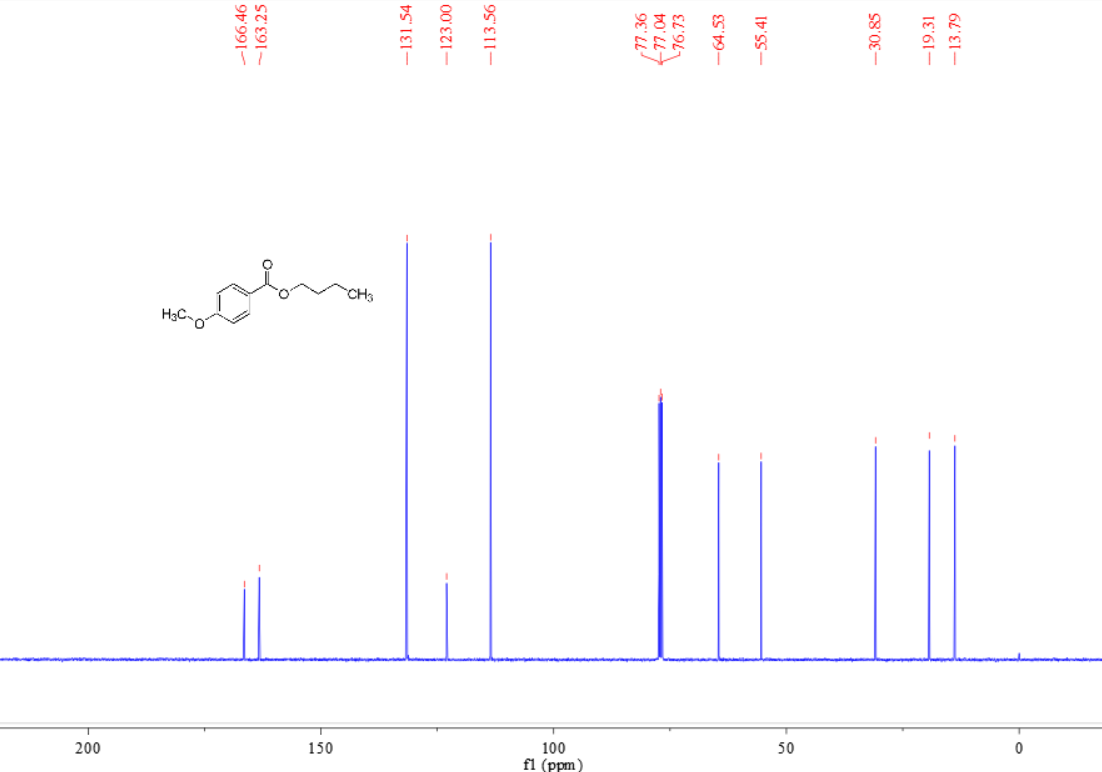


**Compound** **2c** ^1^H NMR (400 MHz, CDCl_3_)


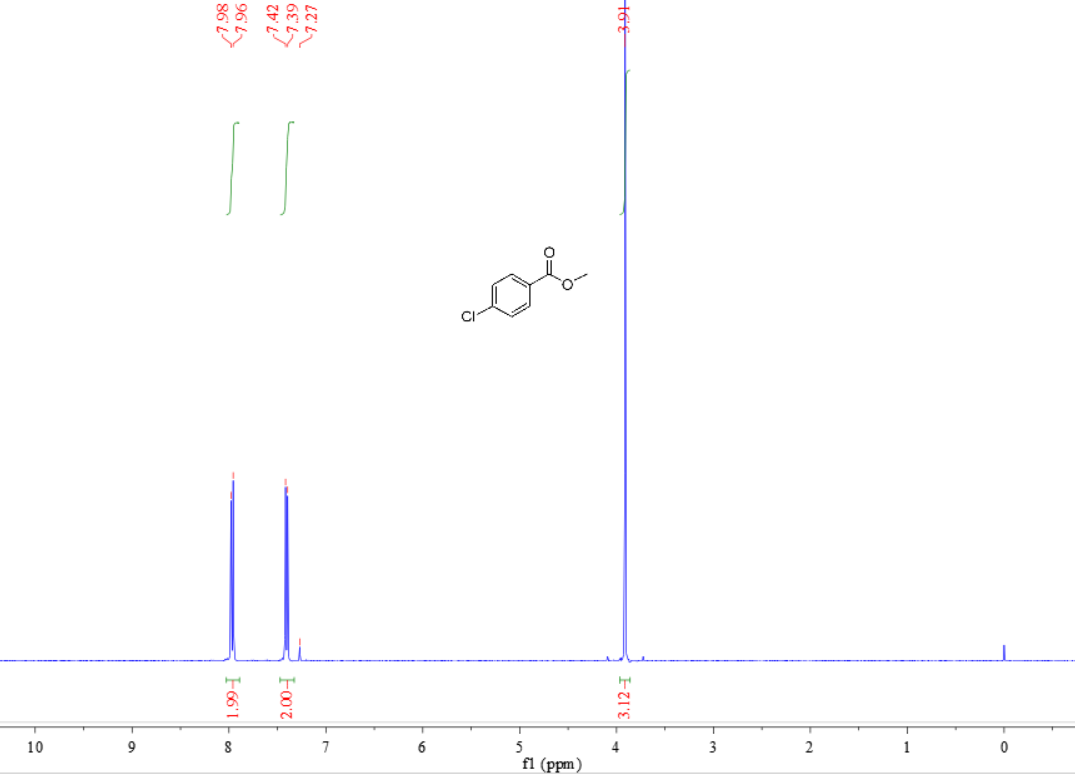


**Compound** **2c** ^13^C{^1^H}NMR (100 MHz, CDCl_3_)


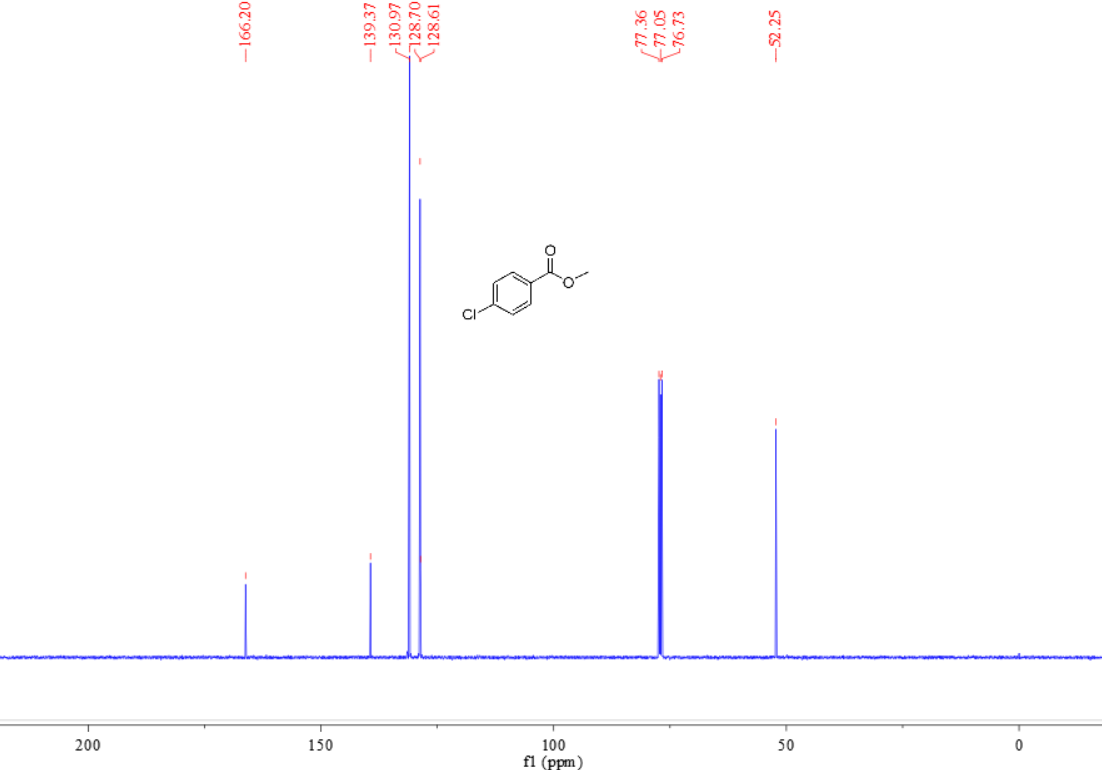


**Compound** **2cb** ^1^H NMR (400 MHz, DMSO-*d*_6_)


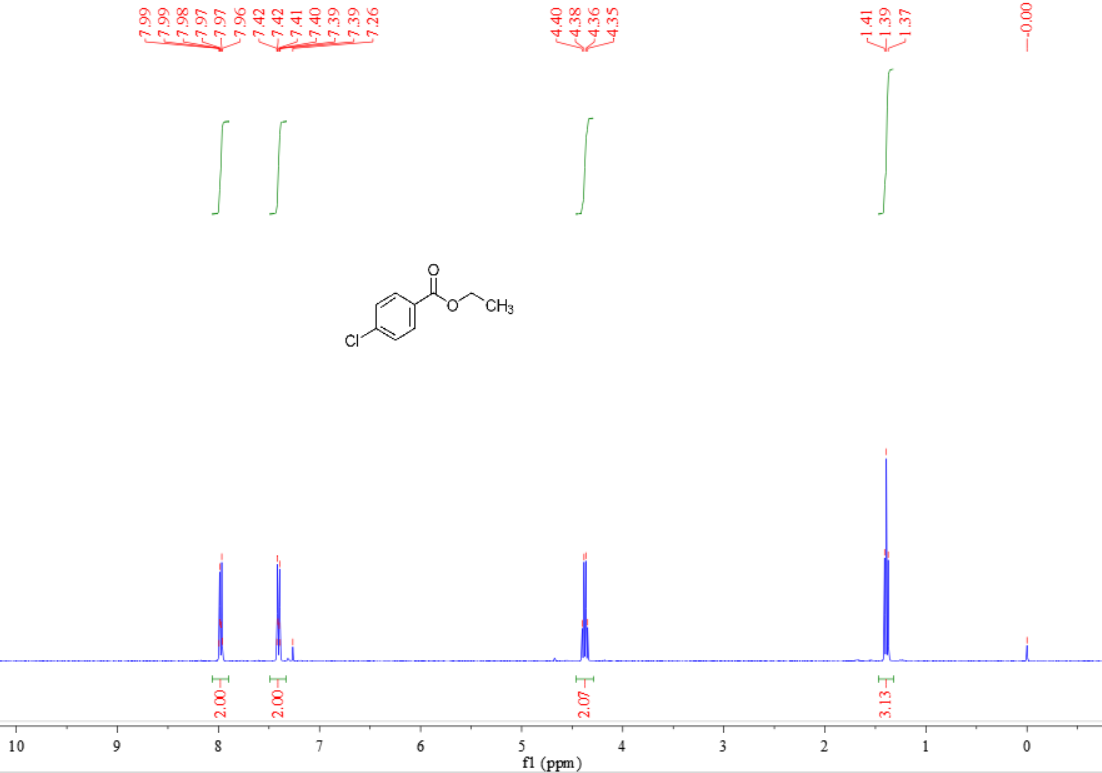


**Compound** **2cb**^13^C{^1^H}NMR (100 MHz, DMSO-*d*_6_)


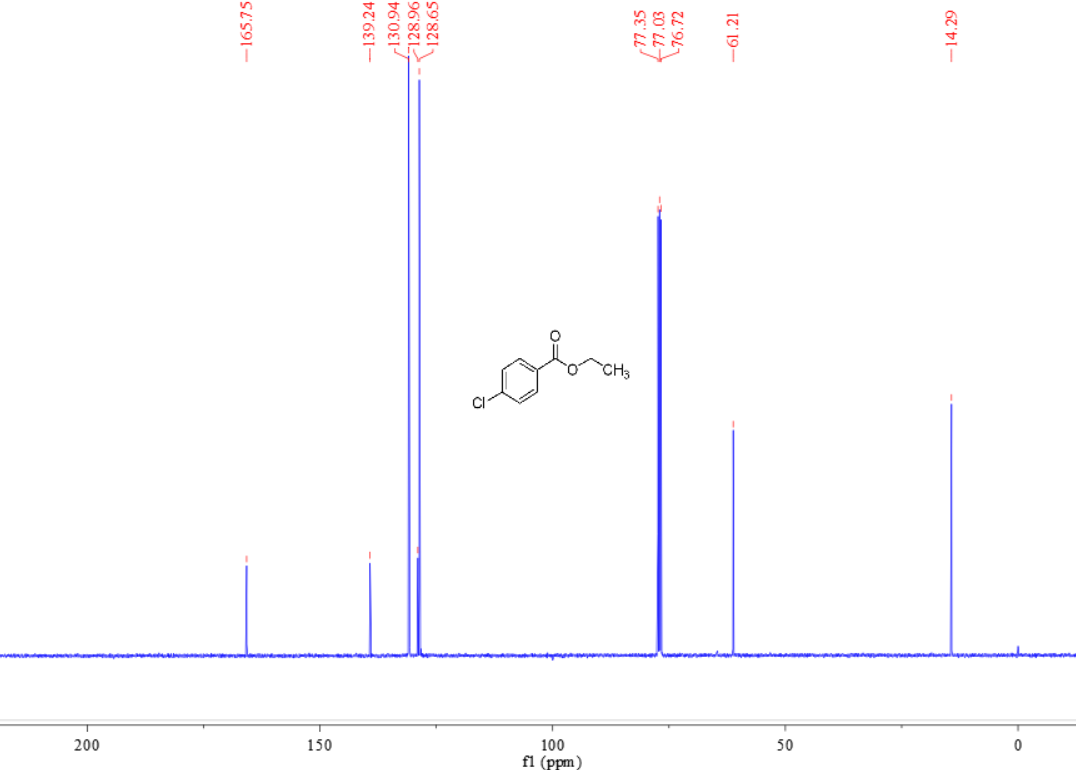


**Compound** **2cc**^1^H NMR (400 MHz, DMSO-*d*_6_)


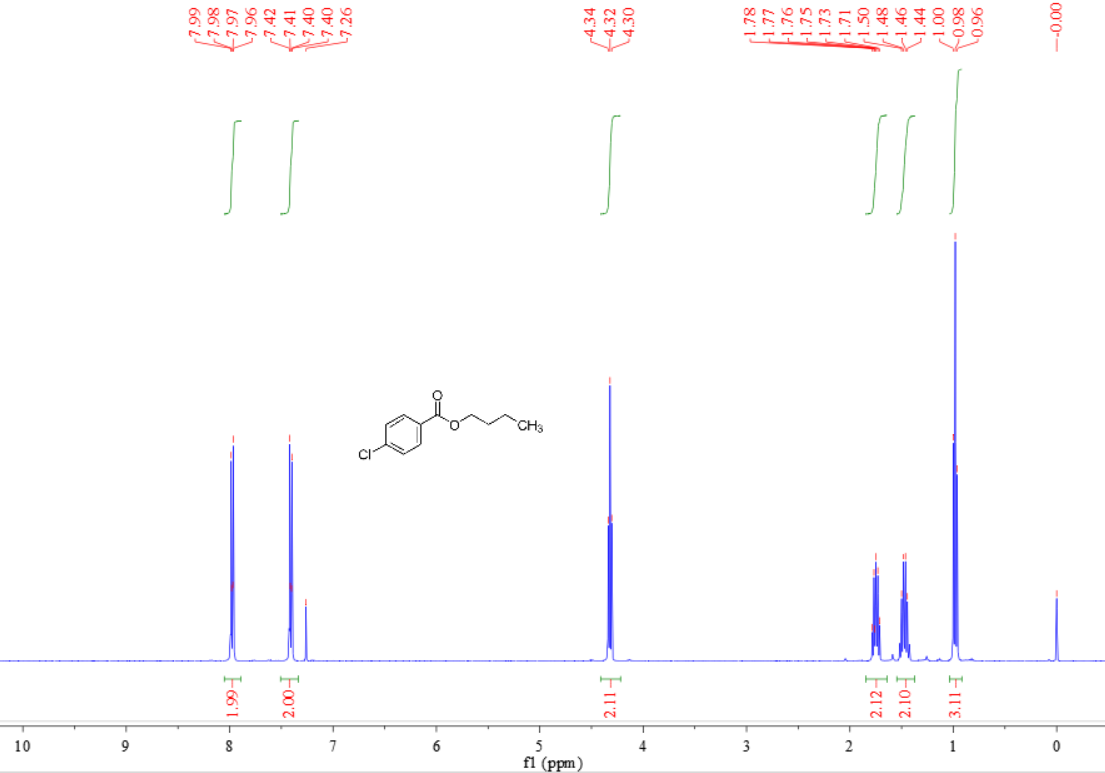


**Compound** **2cc** ^13^C{^1^H}NMR (100 MHz, DMSO-*d*_6_)


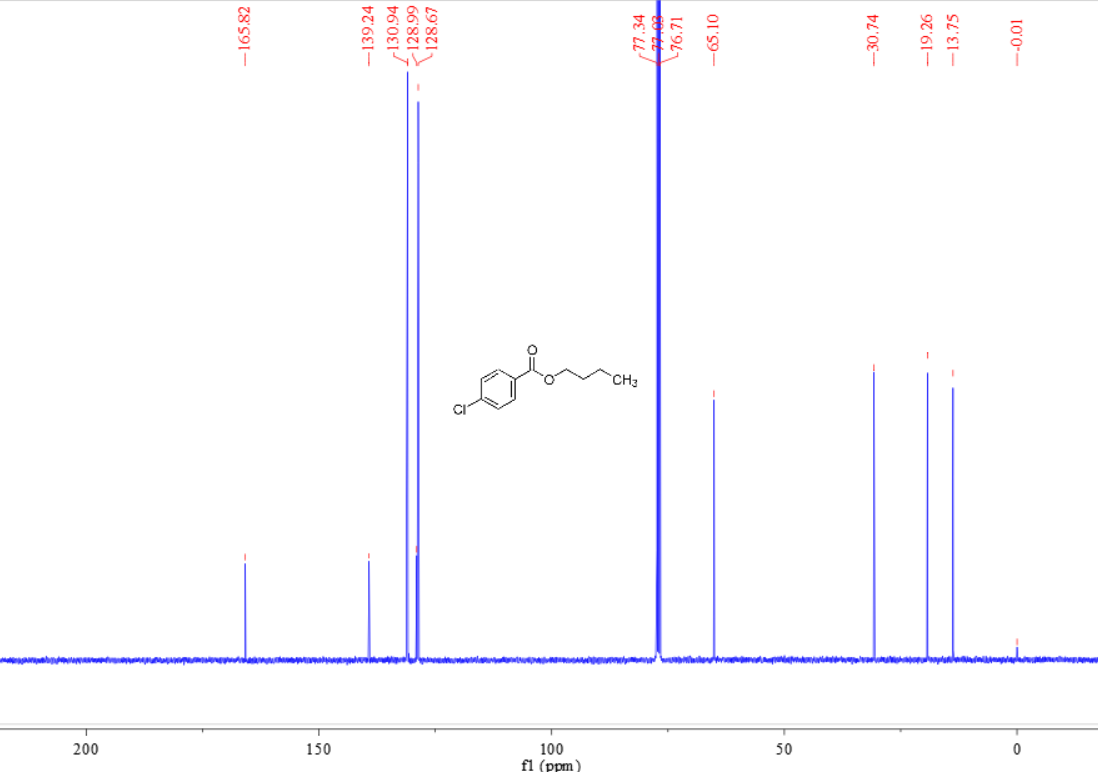


**Compound** **2d** ^1^H NMR (400 MHz, CDCl_3_)


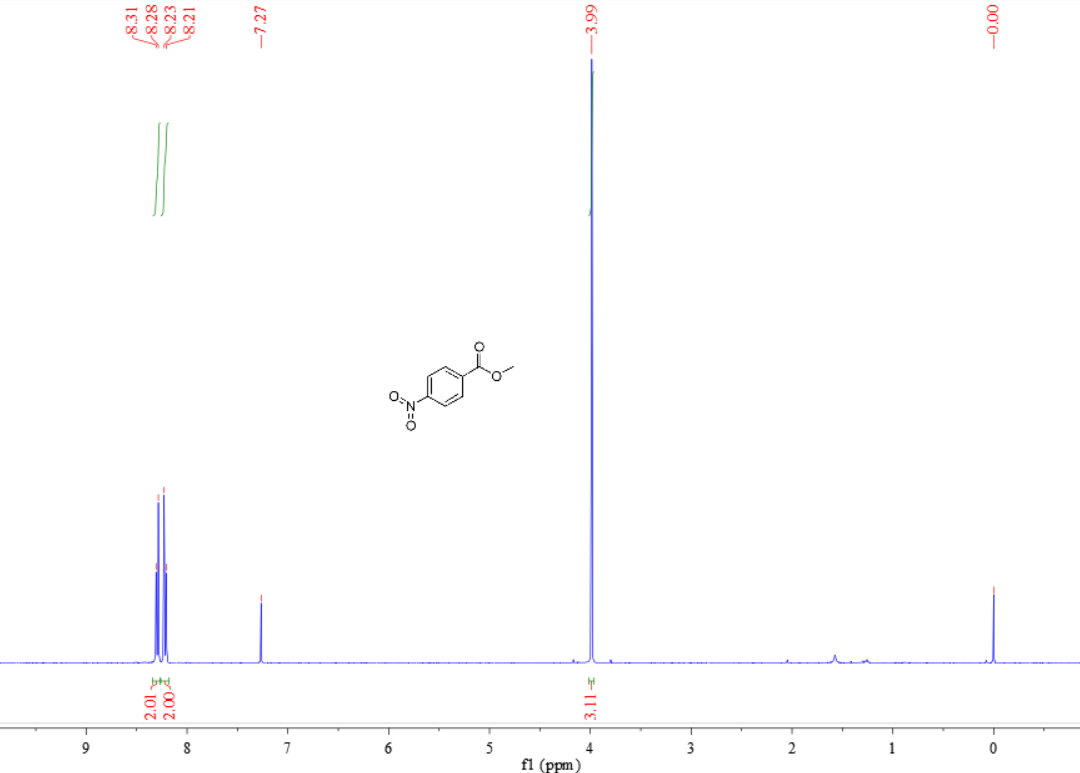


**Compound** **2d** ^13^C{^1^H}NMR (100 MHz, CDCl_3_)


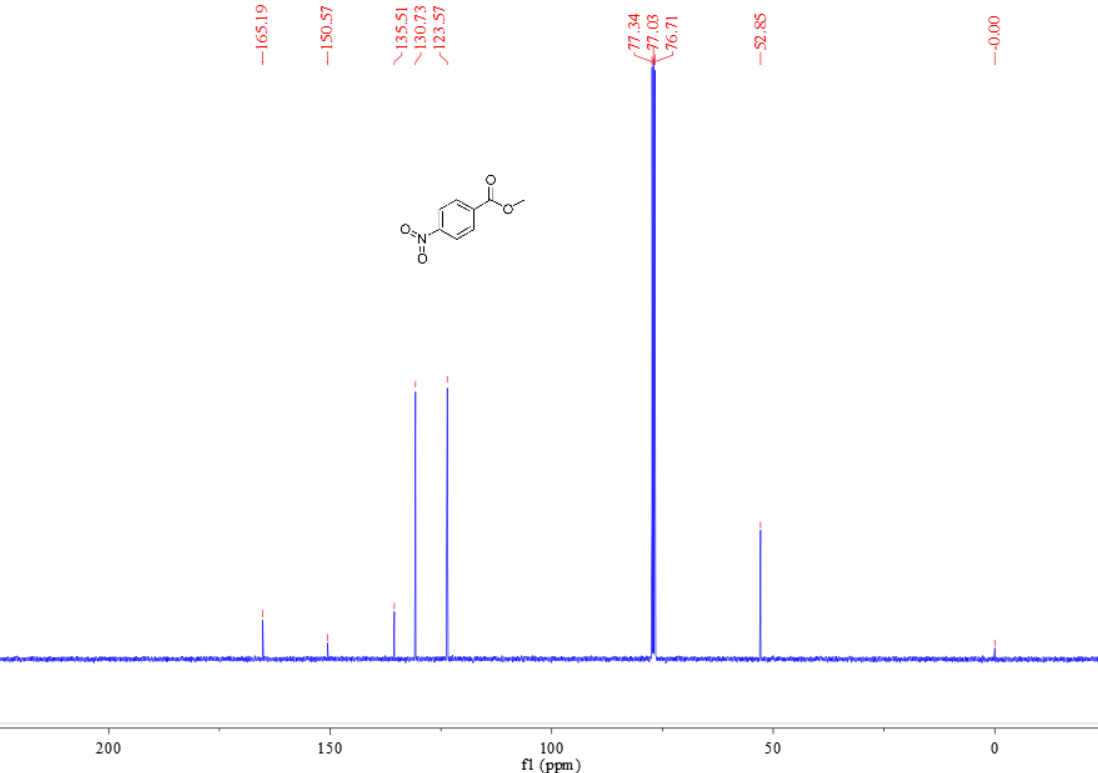


**Compound** **2db** ^1^H NMR (400 MHz, CDCl_3_)


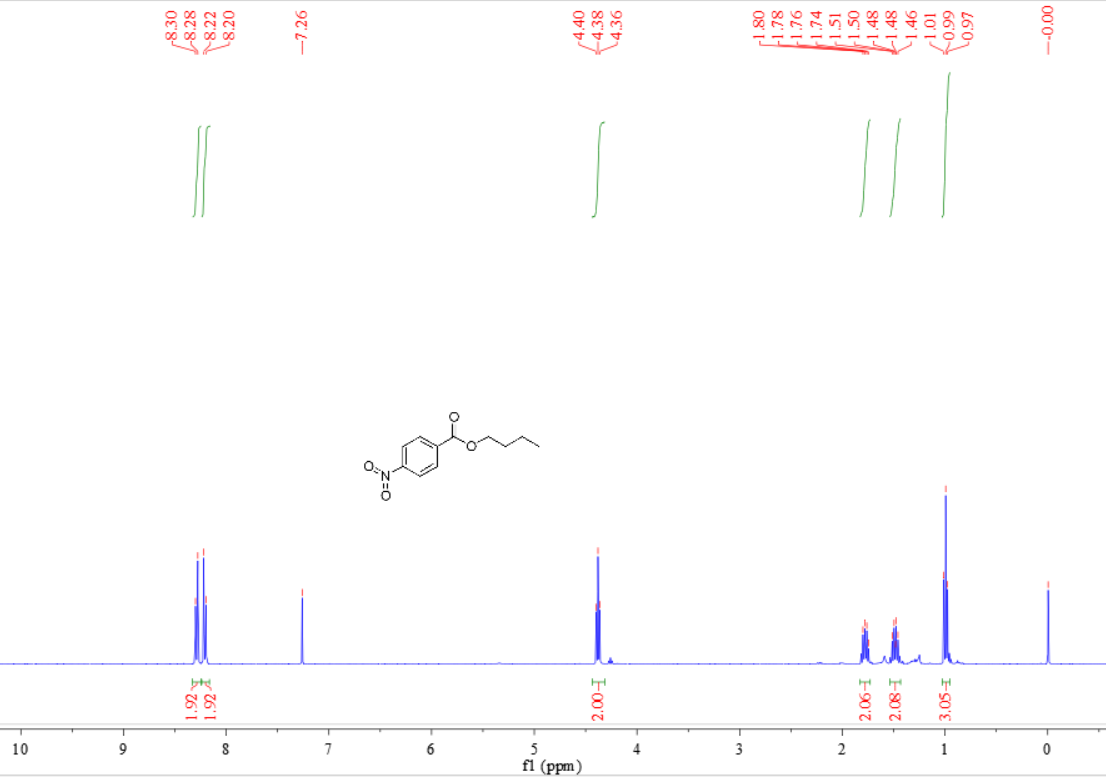


**Compound** **2db** ^13^C{^1^H}NMR (100 MHz, CDCl_3_)


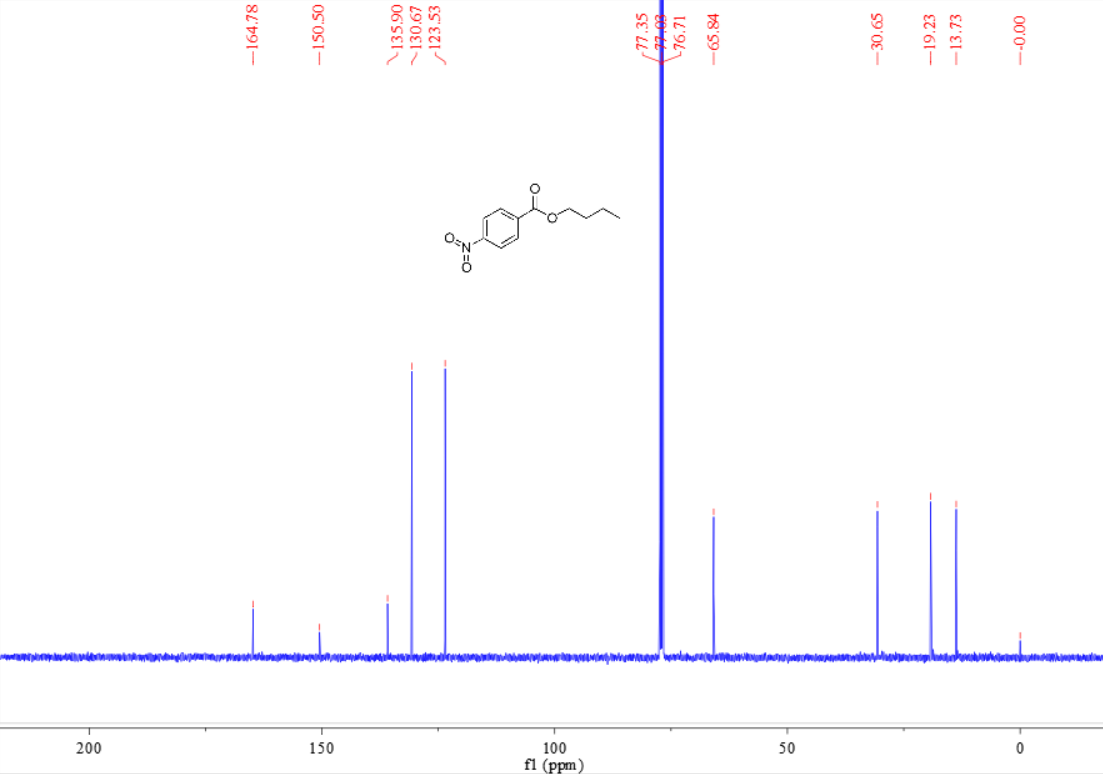


**Compound** **2e** ^1^H NMR (400 MHz, CDCl_3_)


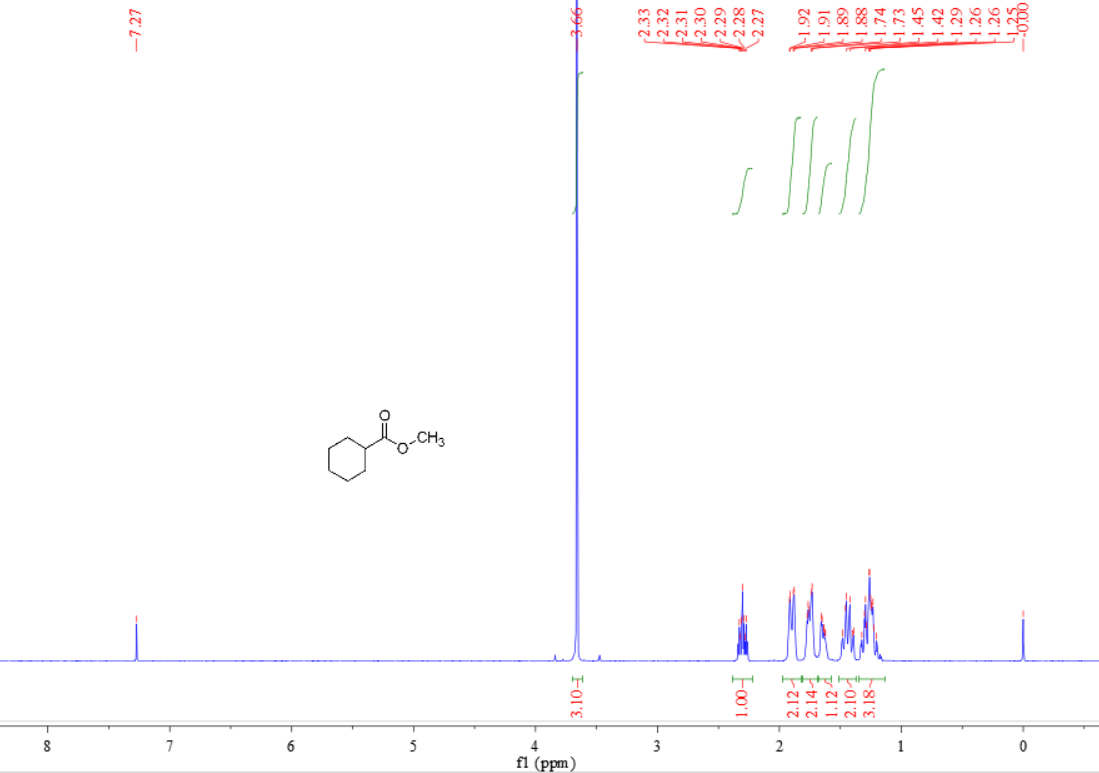


**Compound** **2e** ^13^C{^1^H}NMR (100 MHz, CDCl_3_)


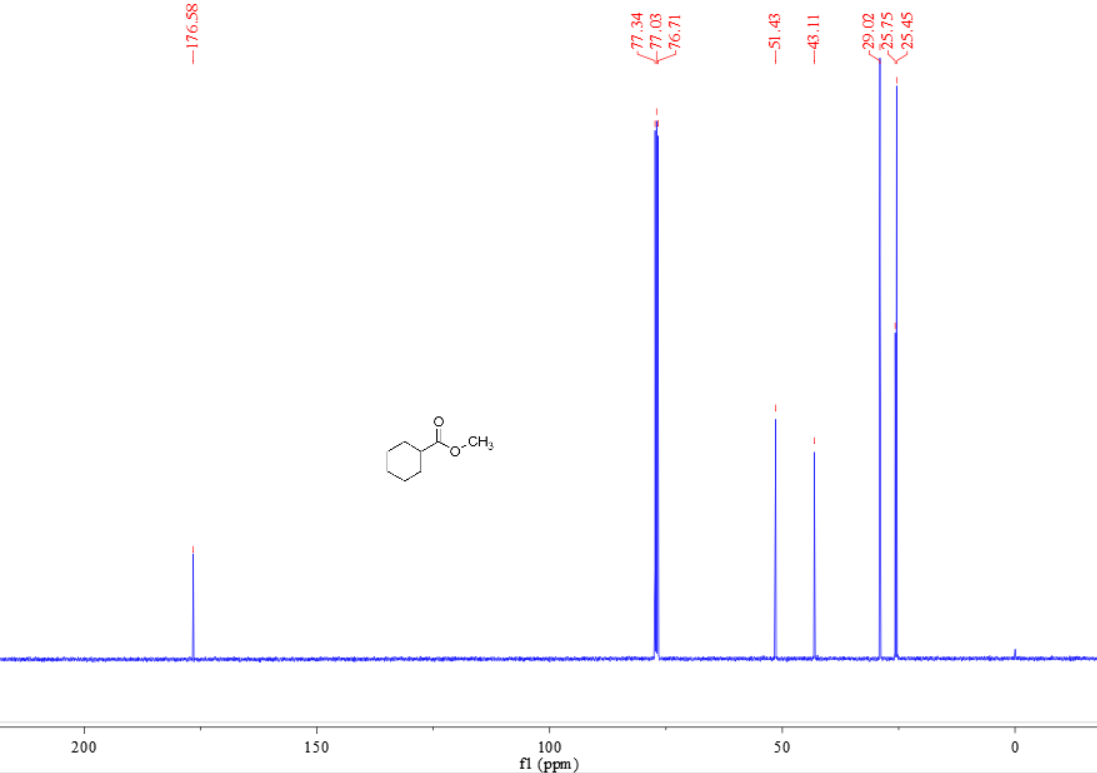


**Compound** **2eb** ^1^H NMR (400 MHz, CDCl_3_)


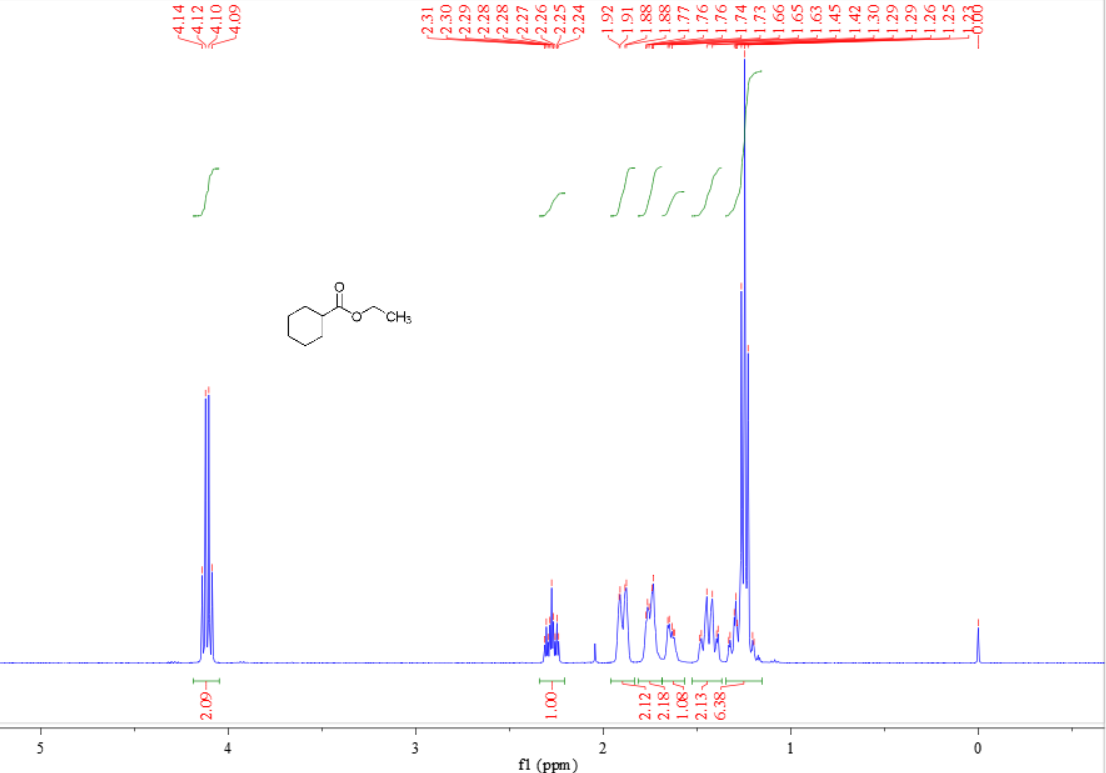


**Compound** **2eb**^13^C{^1^H}NMR (100 MHz, CDCl_3_)


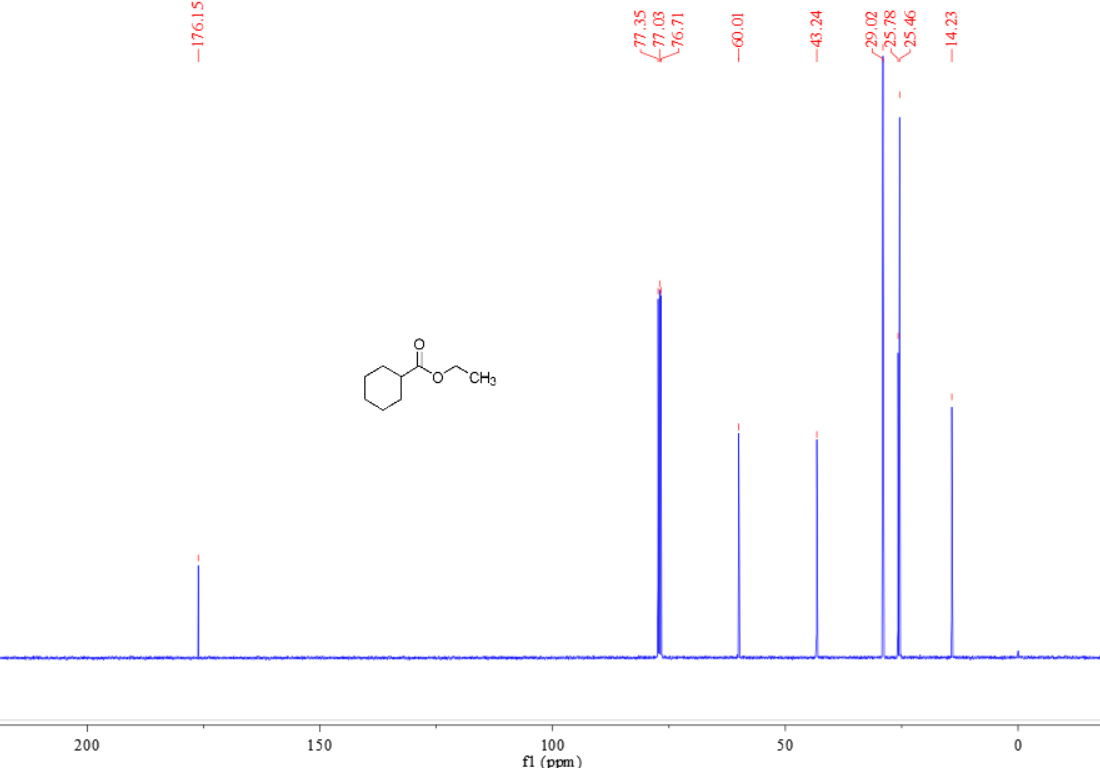


**Compound** **2ec** ^1^H NMR (400 MHz, CDCl_3_)


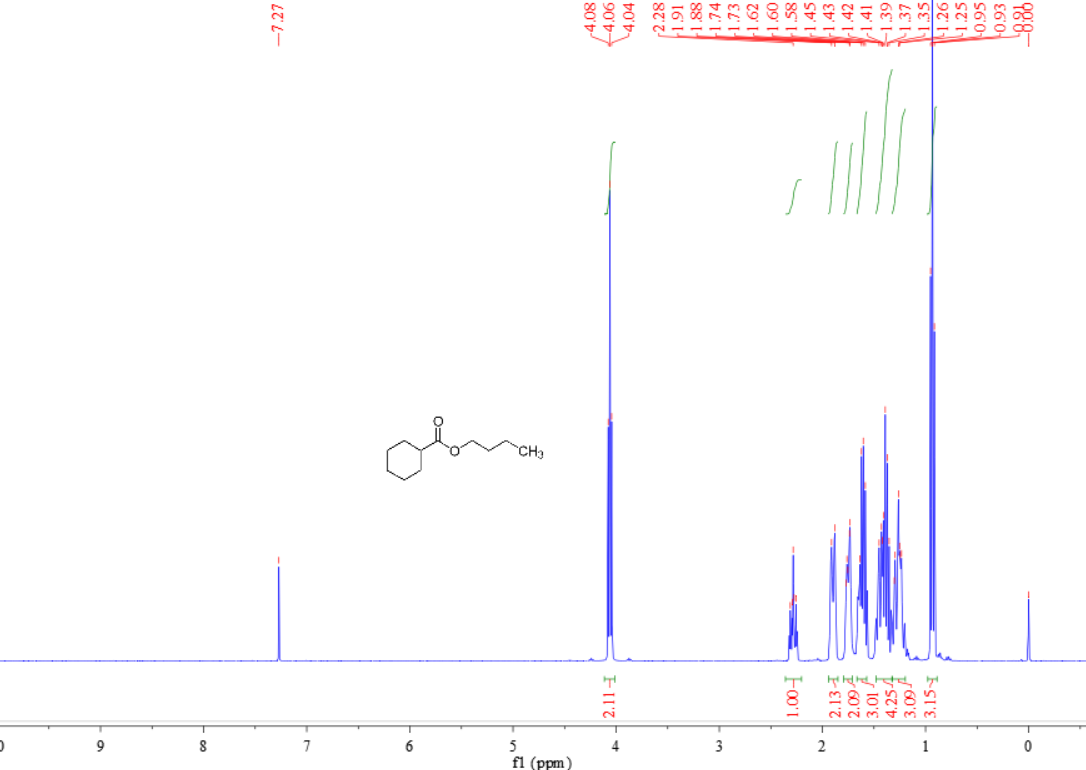


**Compound** **2ec** ^13^C{^1^H}NMR (100 MHz, CDCl_3_)


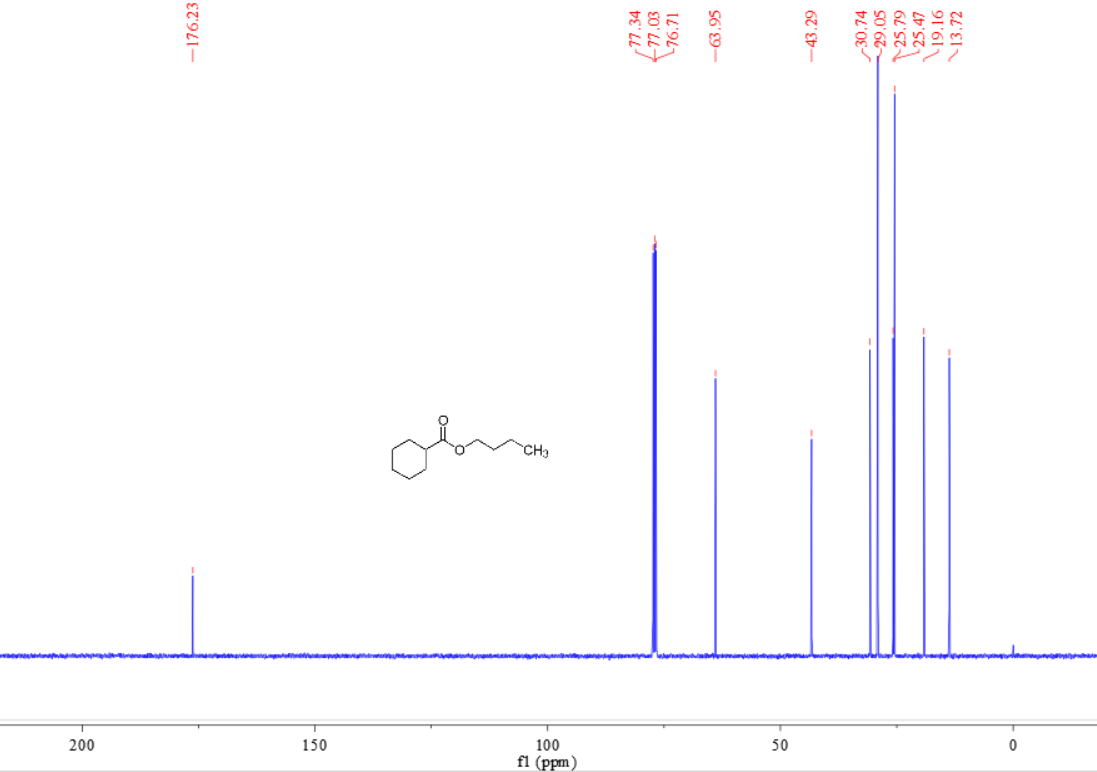


**Compound** **2f** ^1^H NMR (400 MHz, CDCl_3_)


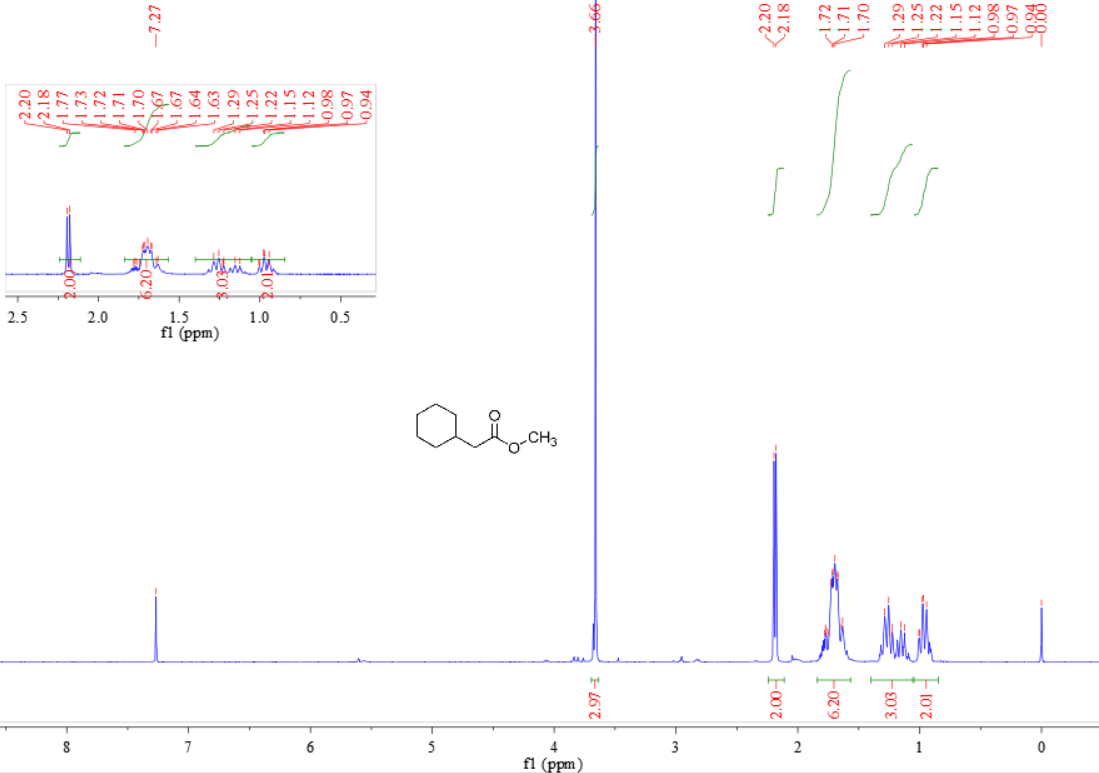


**Compound** **2f** ^13^C{^1^H}NMR (100 MHz, CDCl_3_)


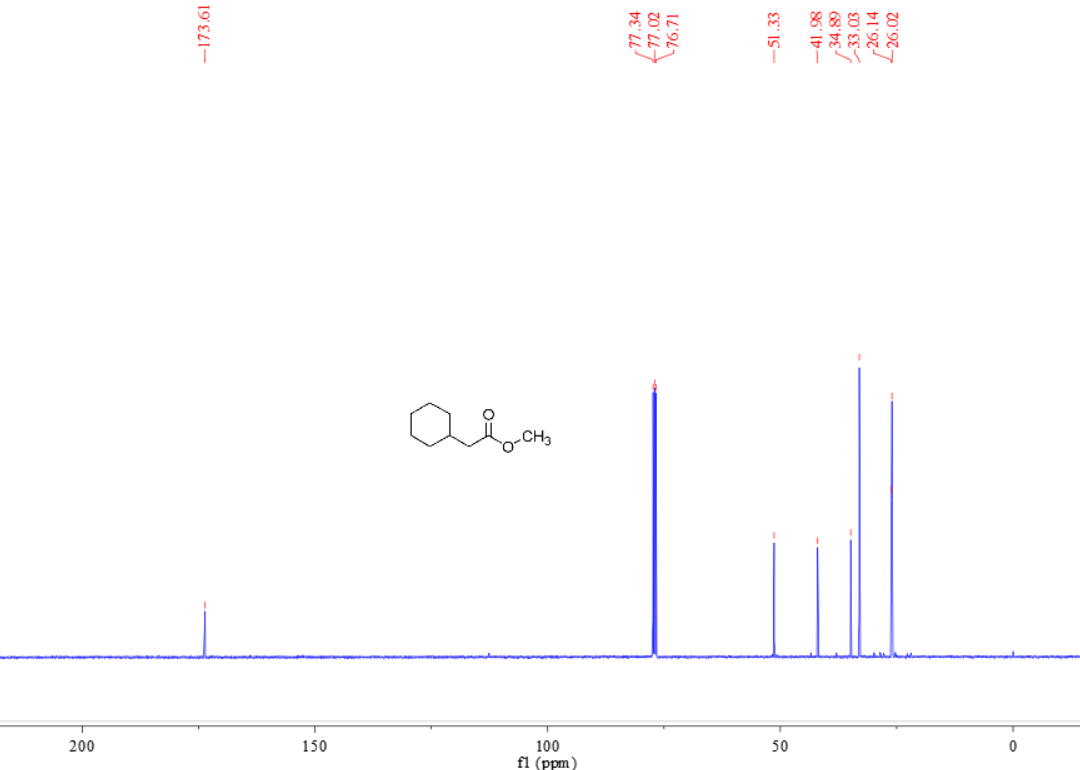


**Compound** **2fb** ^1^H NMR (400 MHz, CDCl_3_)


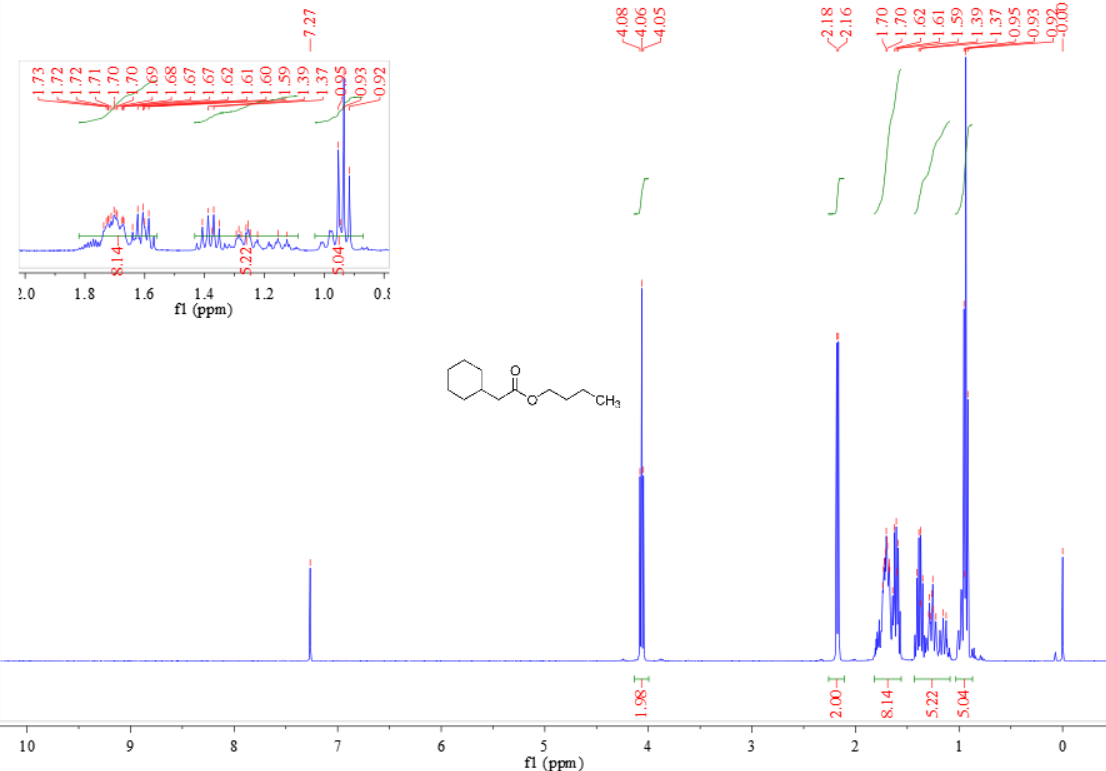


**Compound** **2fb** ^13^C{^1^H}NMR (100 MHz, CDCl_3_)


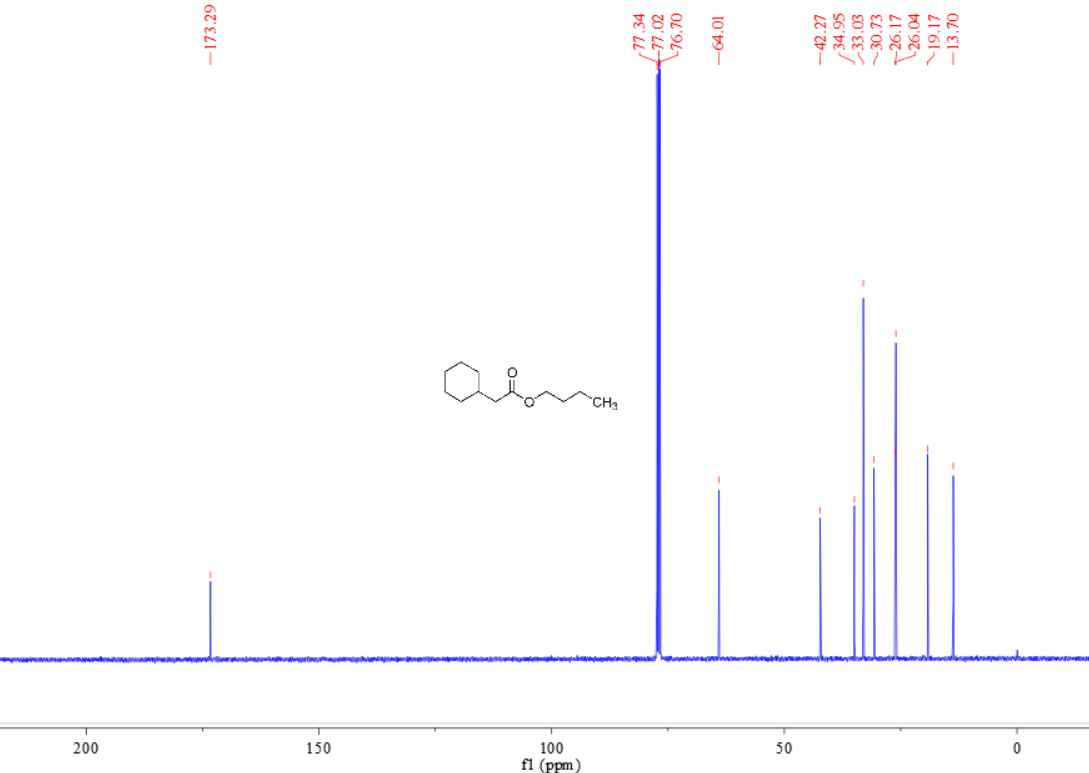


**Compound** **2g** ^1^H NMR (400 MHz, CDCl_3_)


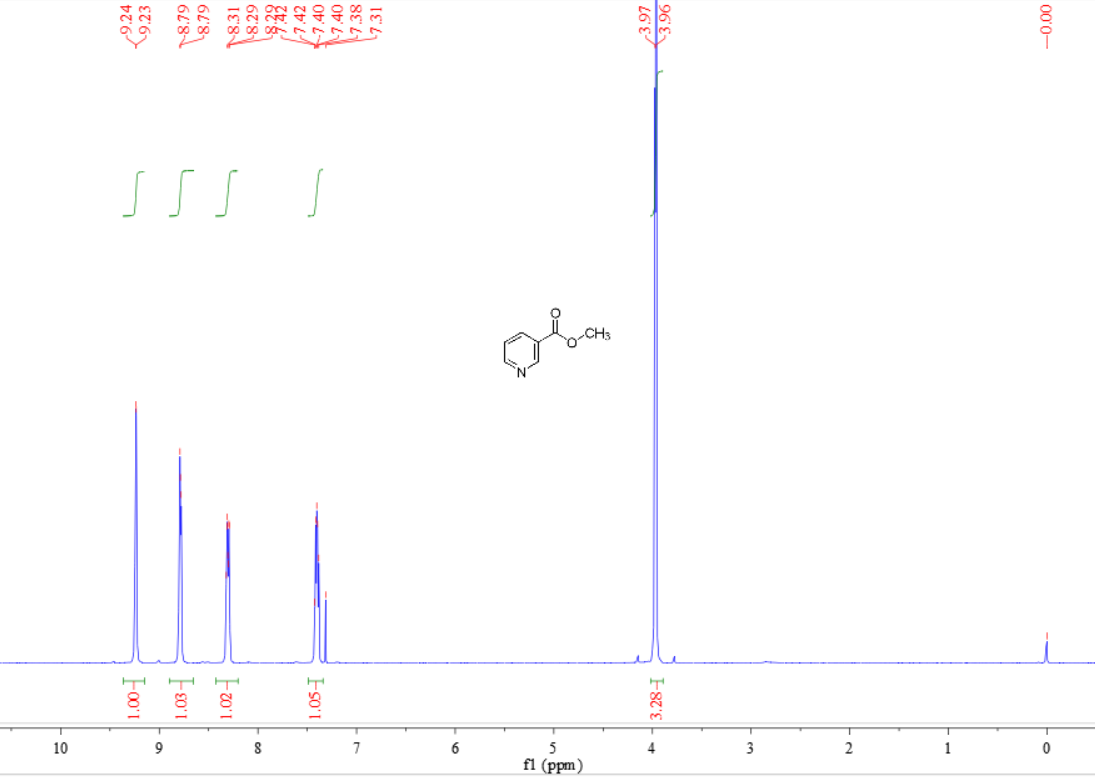


**Compound** **2g** ^13^C{^1^H}NMR (100 MHz, CDCl_3_)


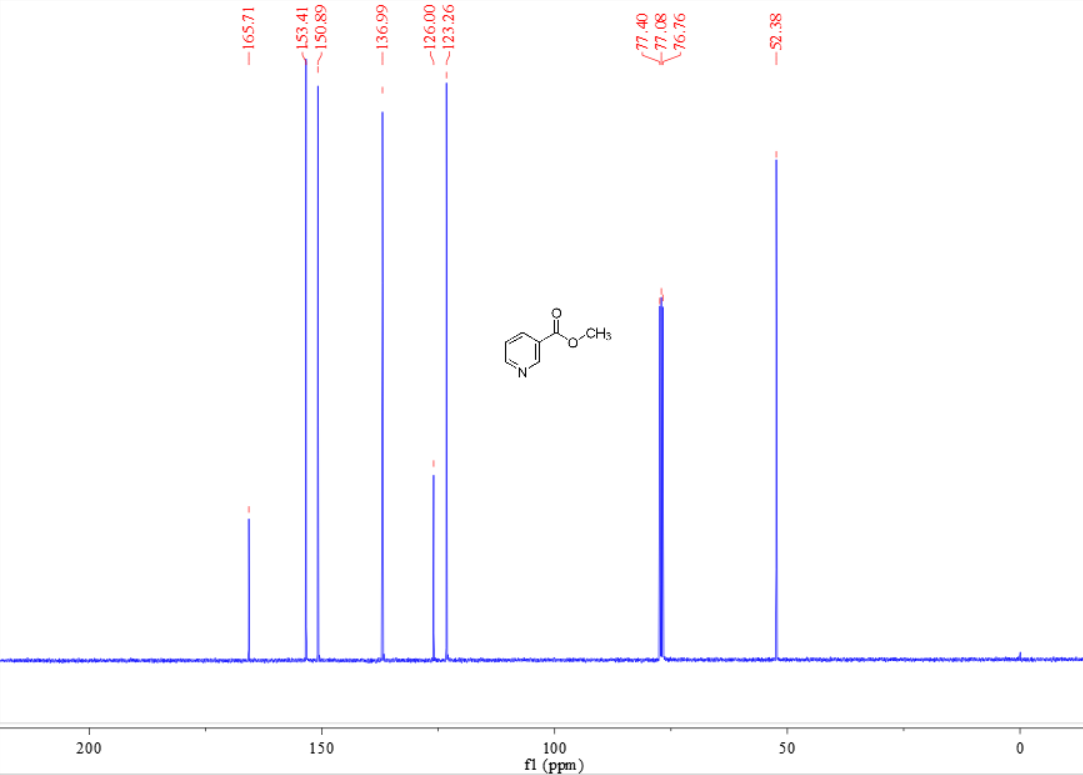


**Compound** **2gb** ^1^H NMR (400 MHz, CDCl_3_)


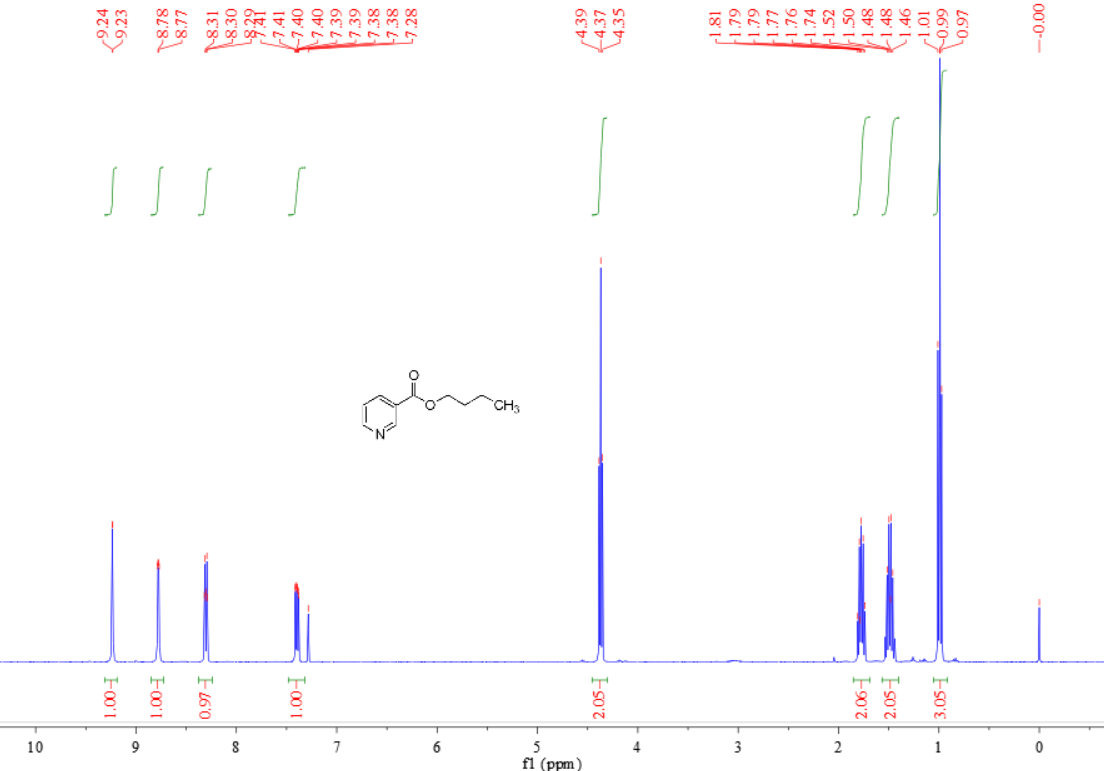


**Compound** **2gb** ^13^C{^1^H}NMR (100 MHz, CDCl_3_)


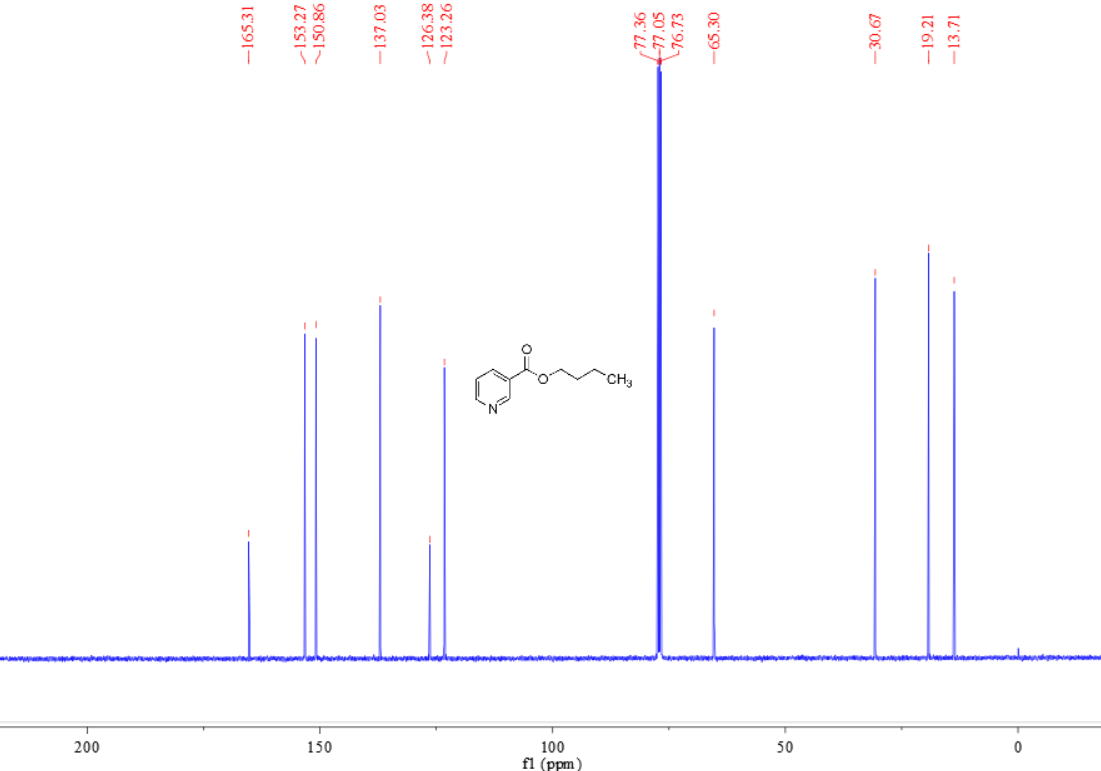


**Compound** **2h** ^1^H NMR (400 MHz, CDCl_3_)


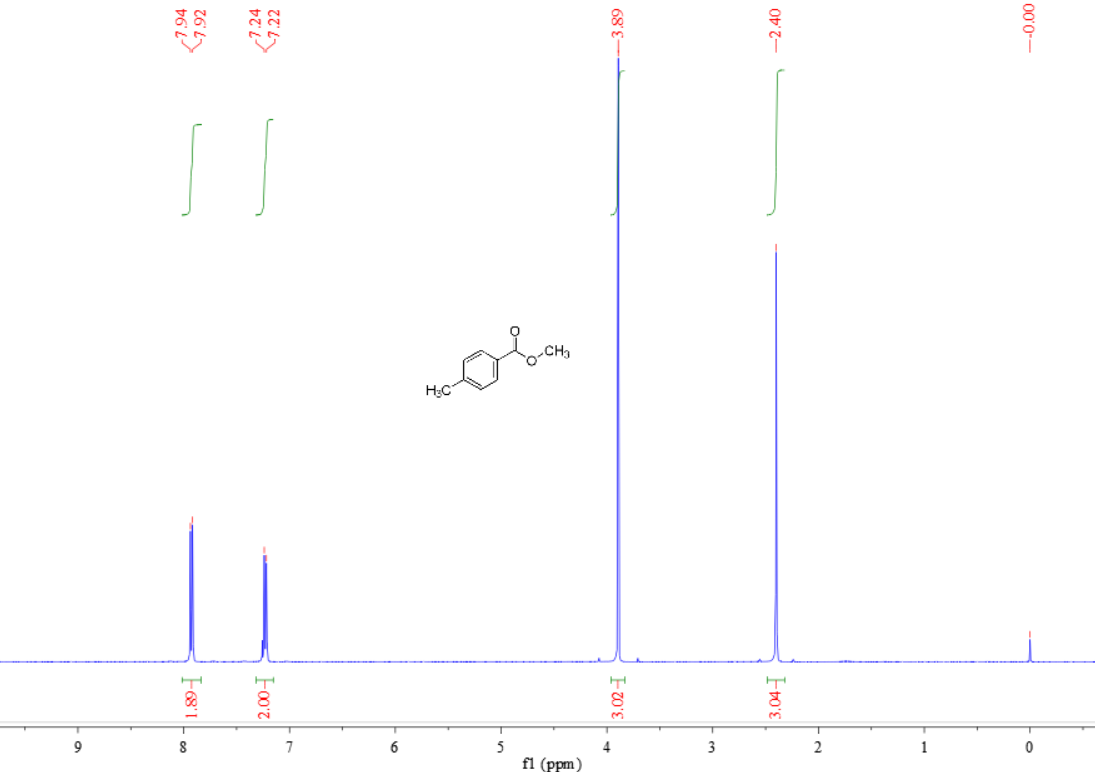


**Compound** **2h** ^13^C{^1^H}NMR (100 MHz, CDCl_3_)


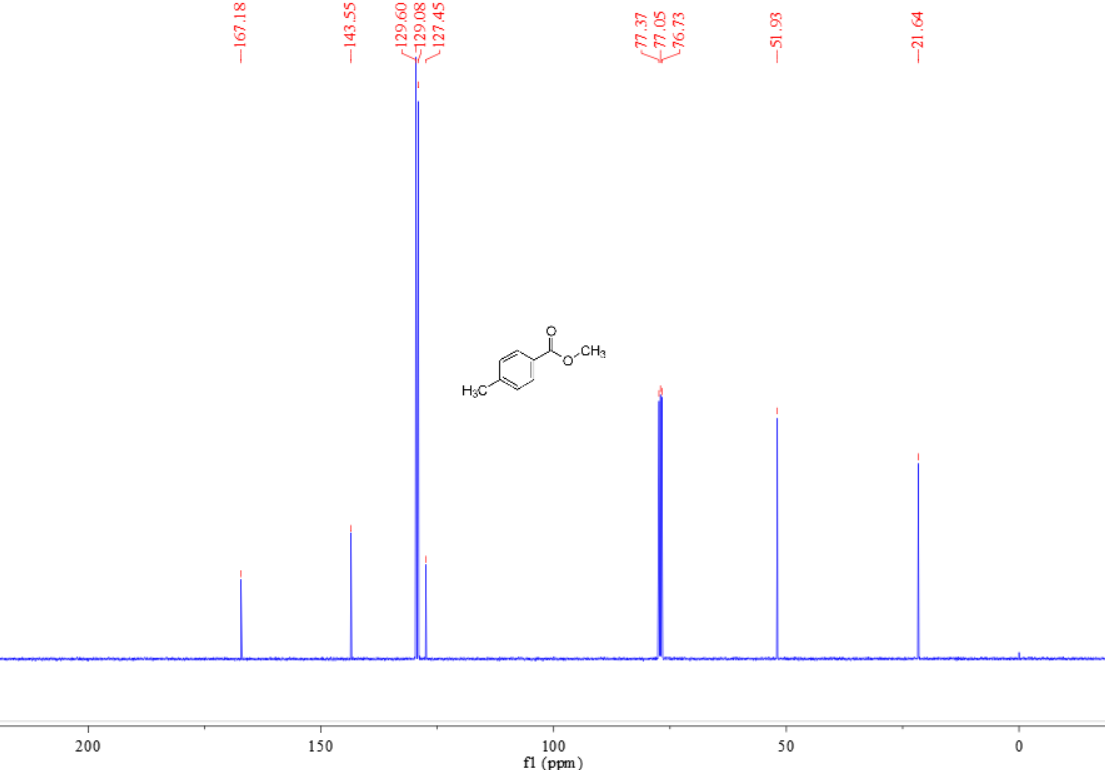


**Compound** **2hb** ^1^H NMR (400 MHz, CDCl_3_)


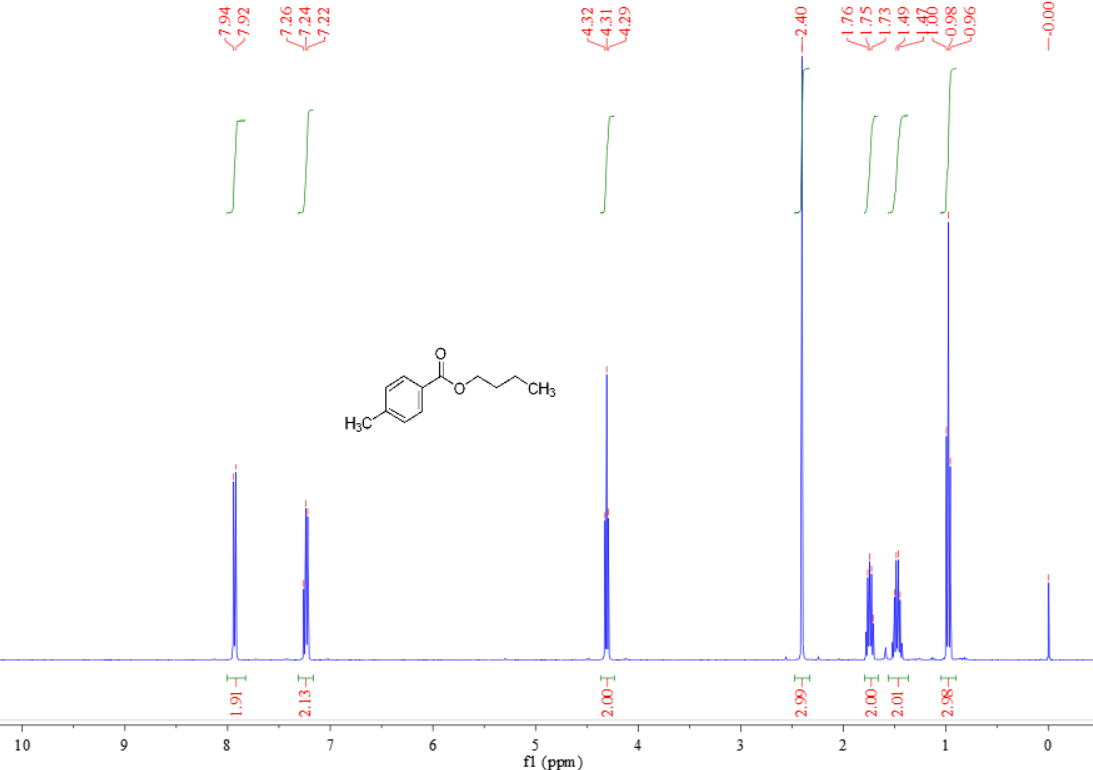


**Compound** **2hb** ^13^C{^1^H}NMR (100 MHz, CDCl_3_)


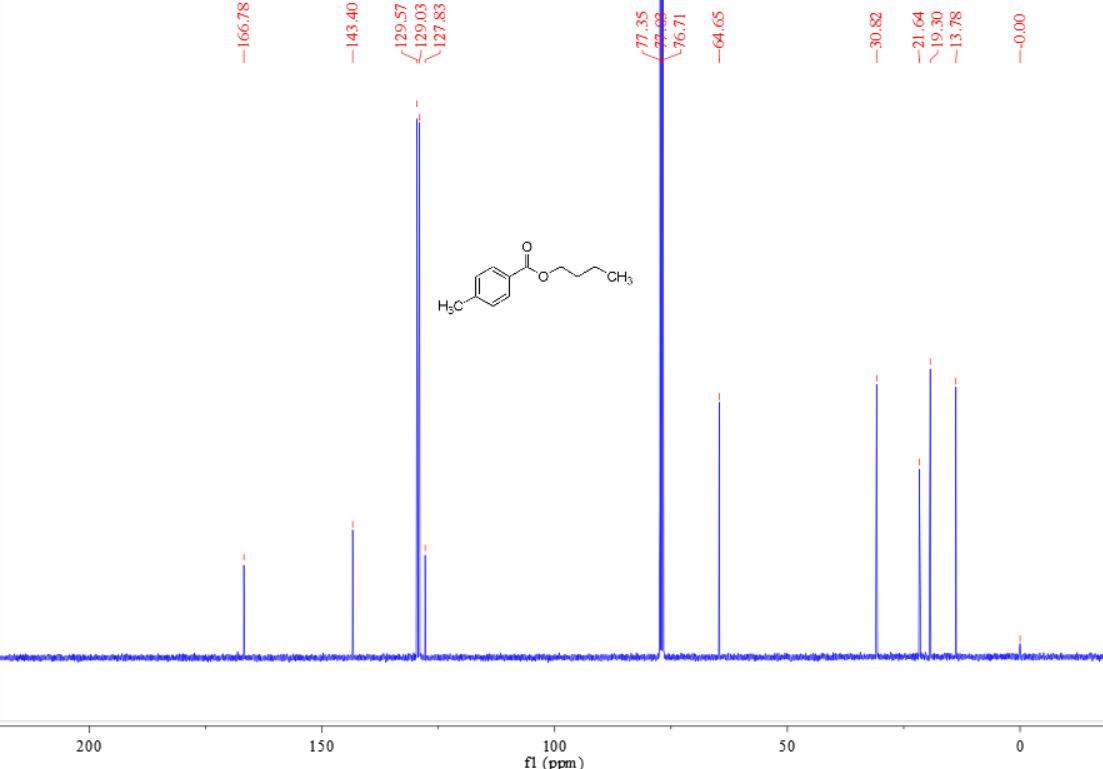


**Compound** **2i** ^1^H NMR (400 MHz, CDCl_3_)


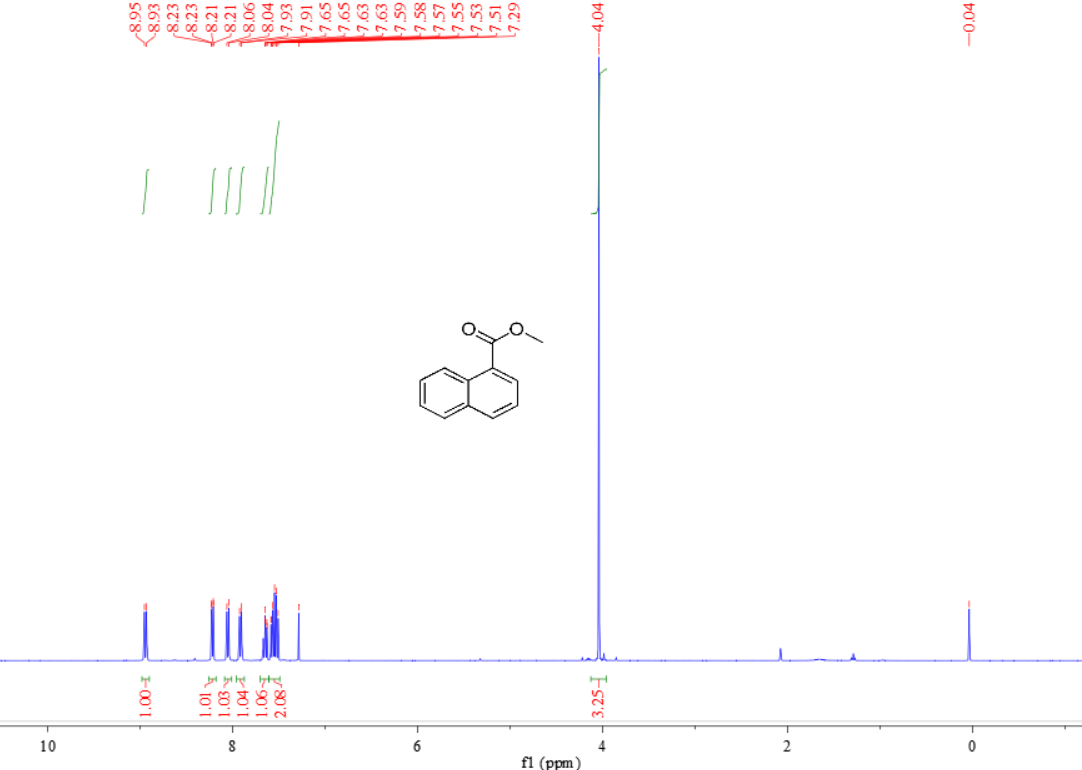


**Compound** **2i** ^13^C{^1^H}NMR (100 MHz, CDCl_3_)


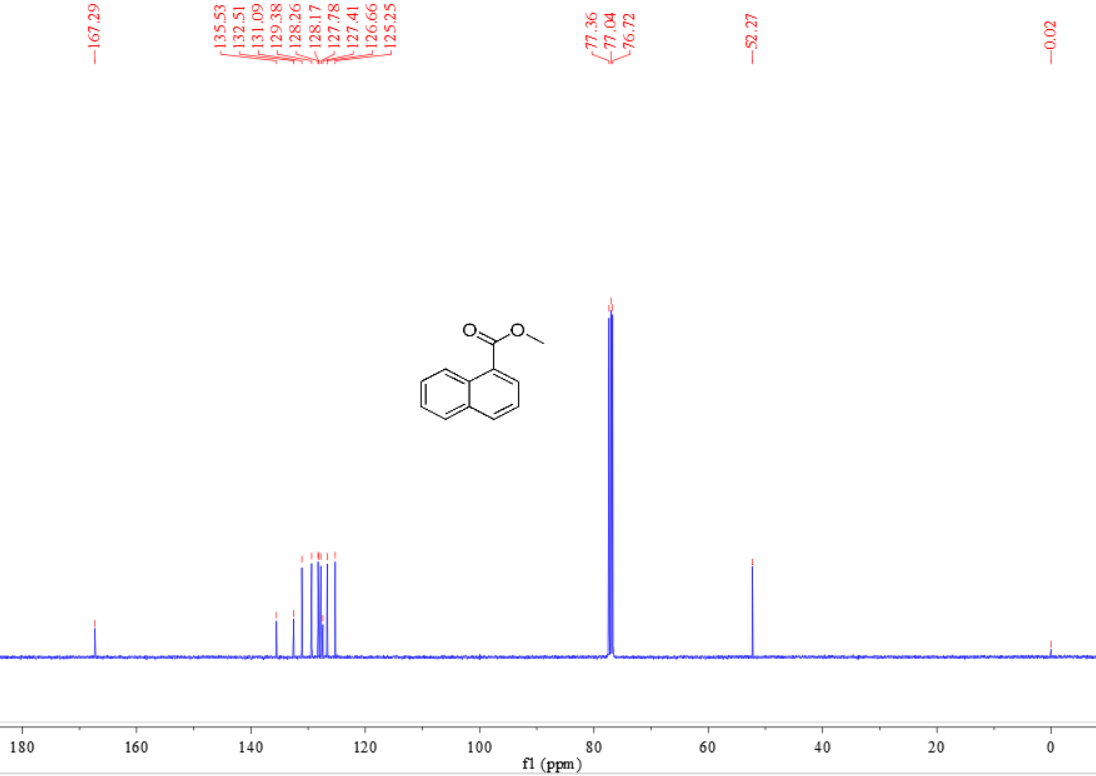


**Compound** **2ib** ^1^H NMR (400 MHz, DMSO-*d*_6_)


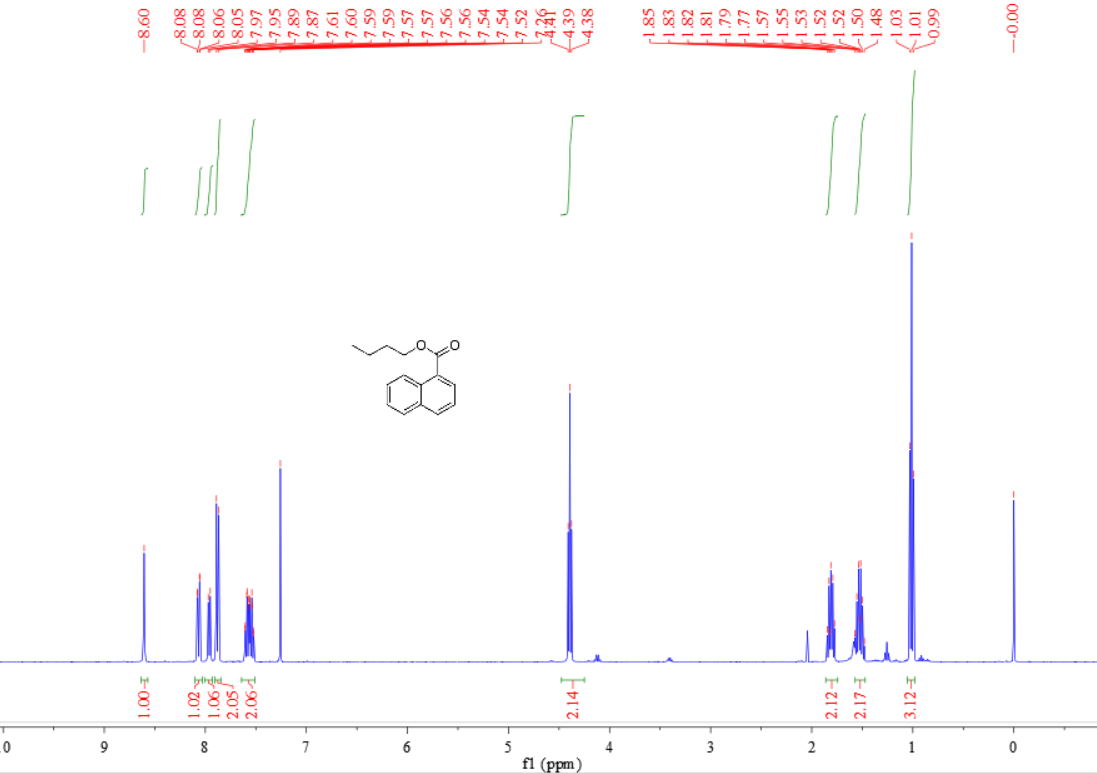


**Compound** **2ib** ^13^C{^1^H}NMR (100 MHz, DMSO-*d*_6_)


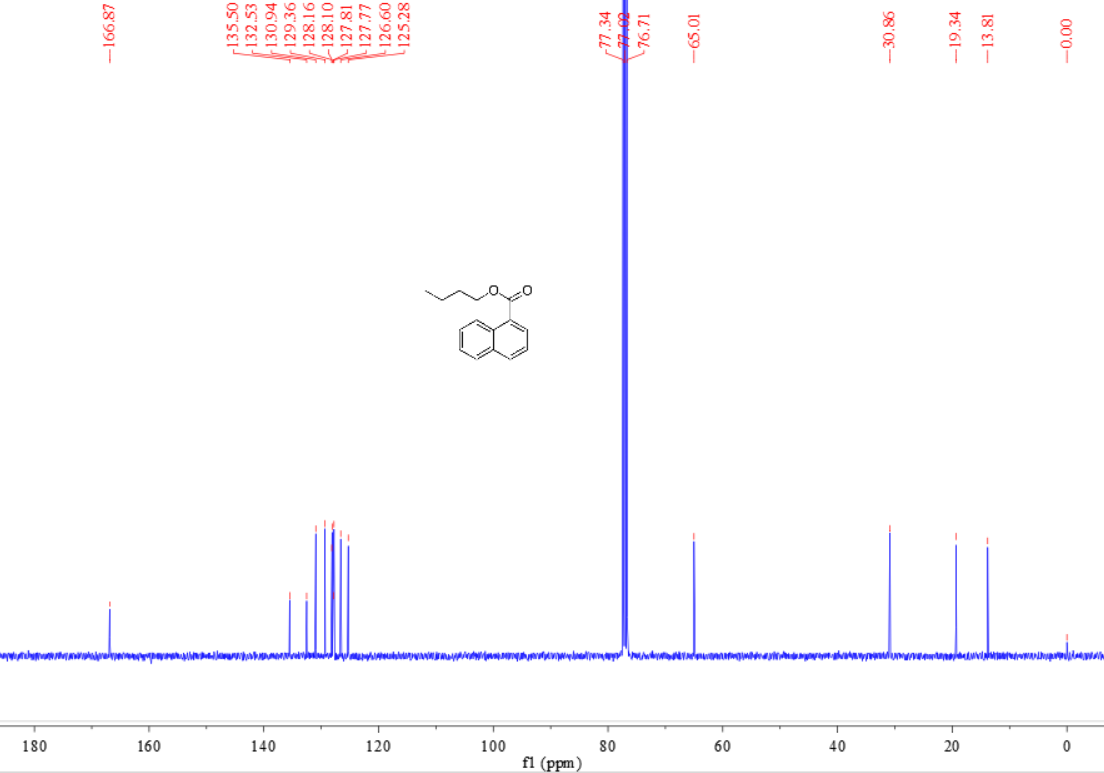


**Compound** **2j** ^1^H NMR (400 MHz, CDCl_3_)


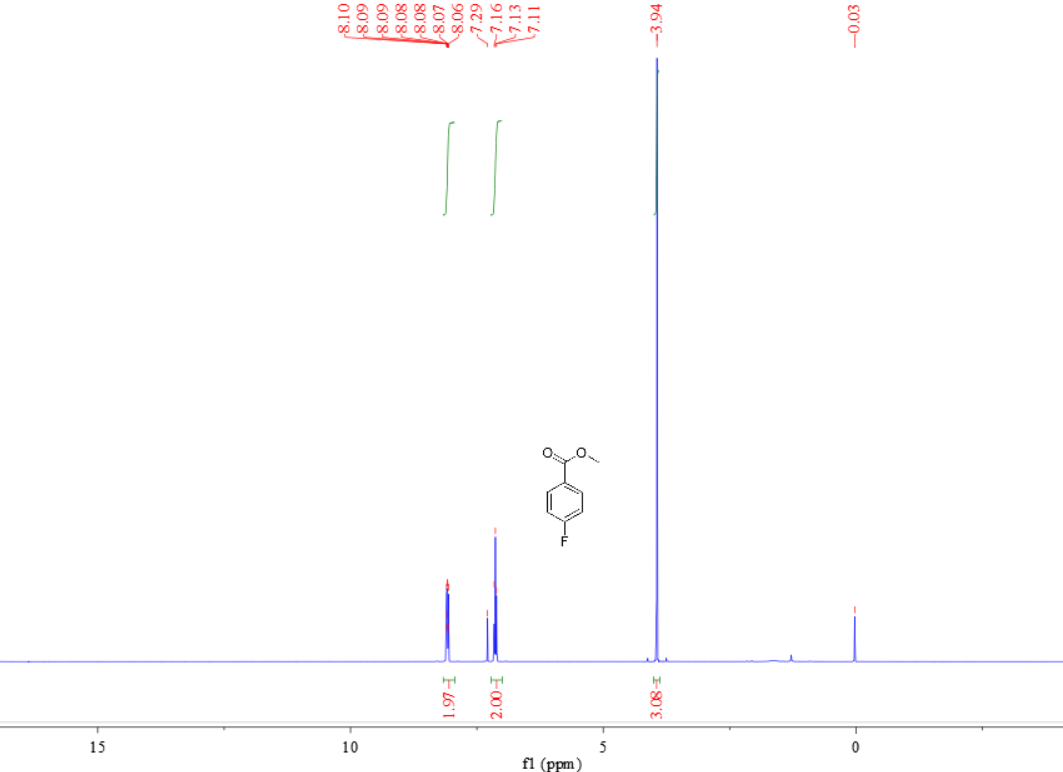


**Compound** **2j** ^13^C{^1^H}NMR (100 MHz, CDCl_3_)


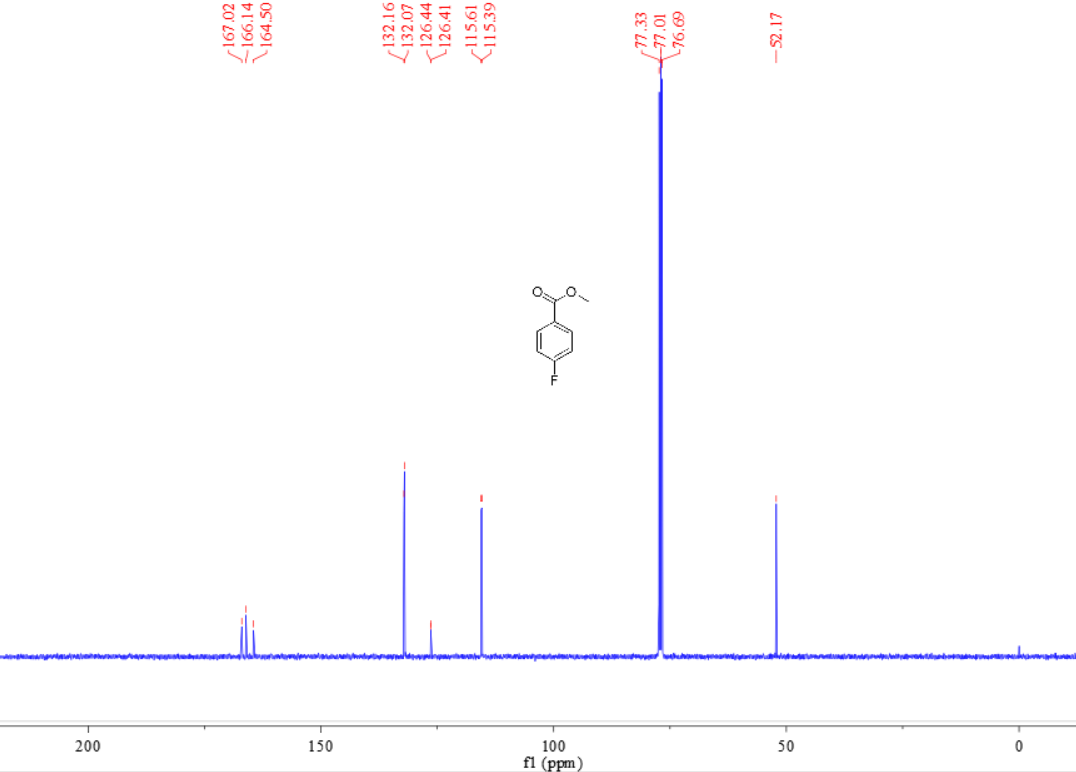


**Compound** **2jb** ^1^H NMR (400 MHz, CDCl_3_)


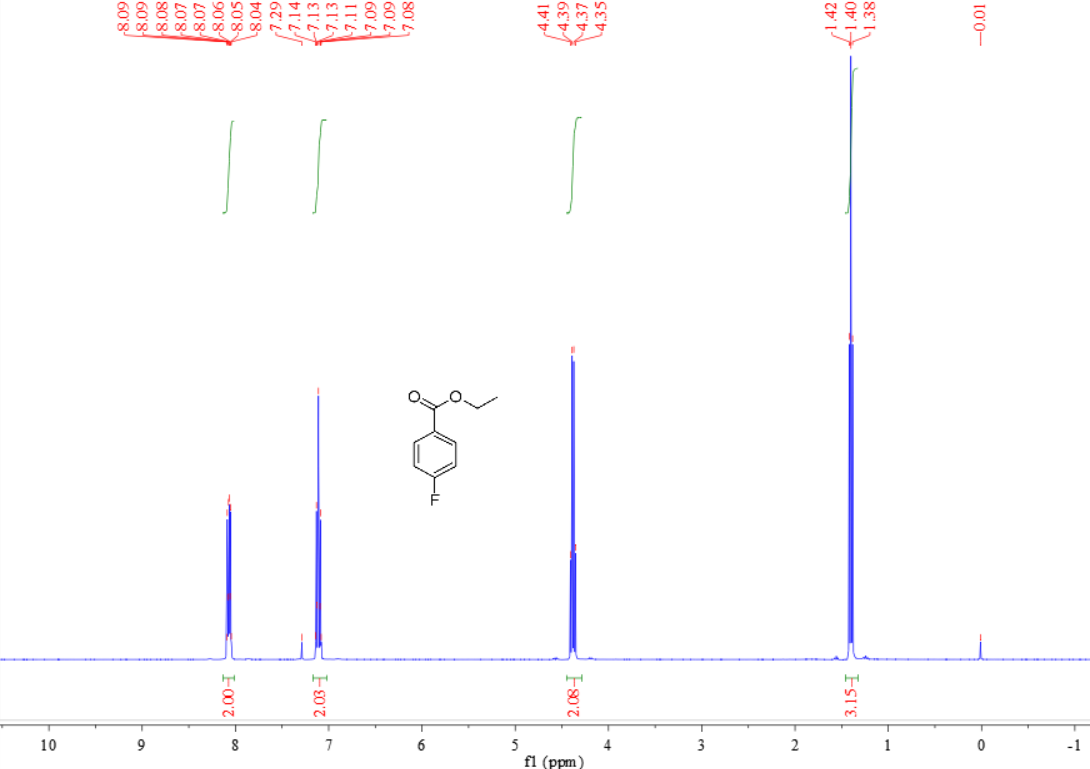


**Compound** **2jb** ^13^C{^1^H}NMR (100 MHz, CDCl_3_)


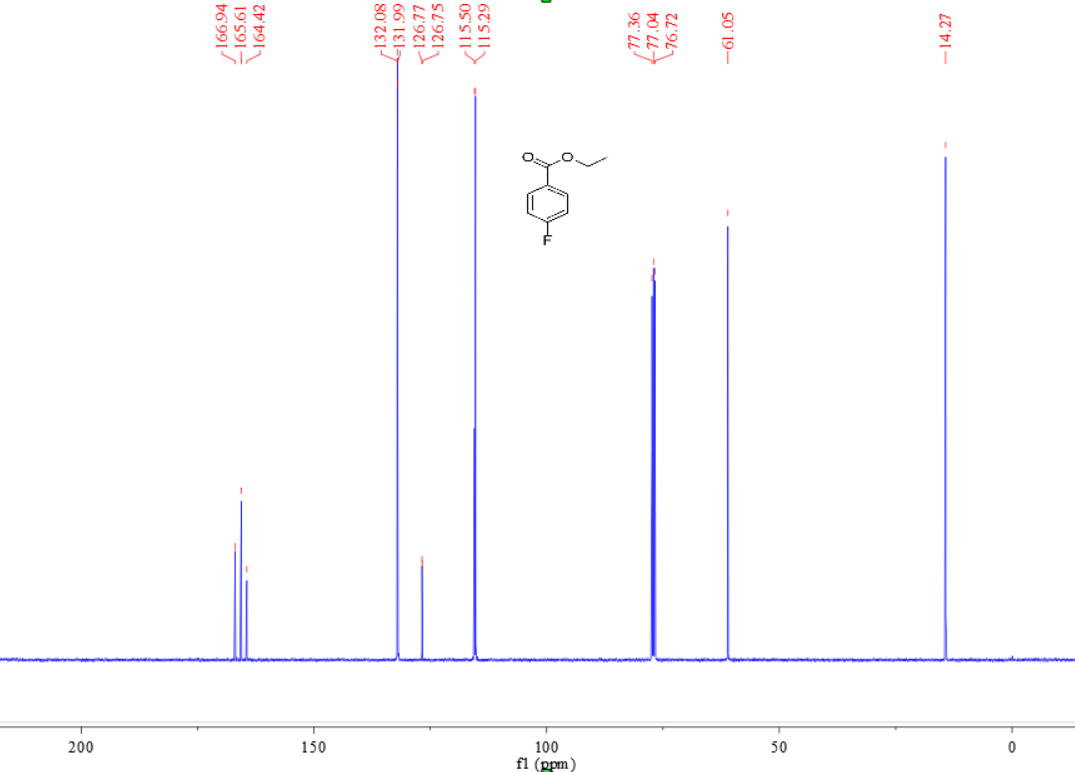


**Compound** **2jc** ^1^H NMR (400 MHz, CDCl_3_)


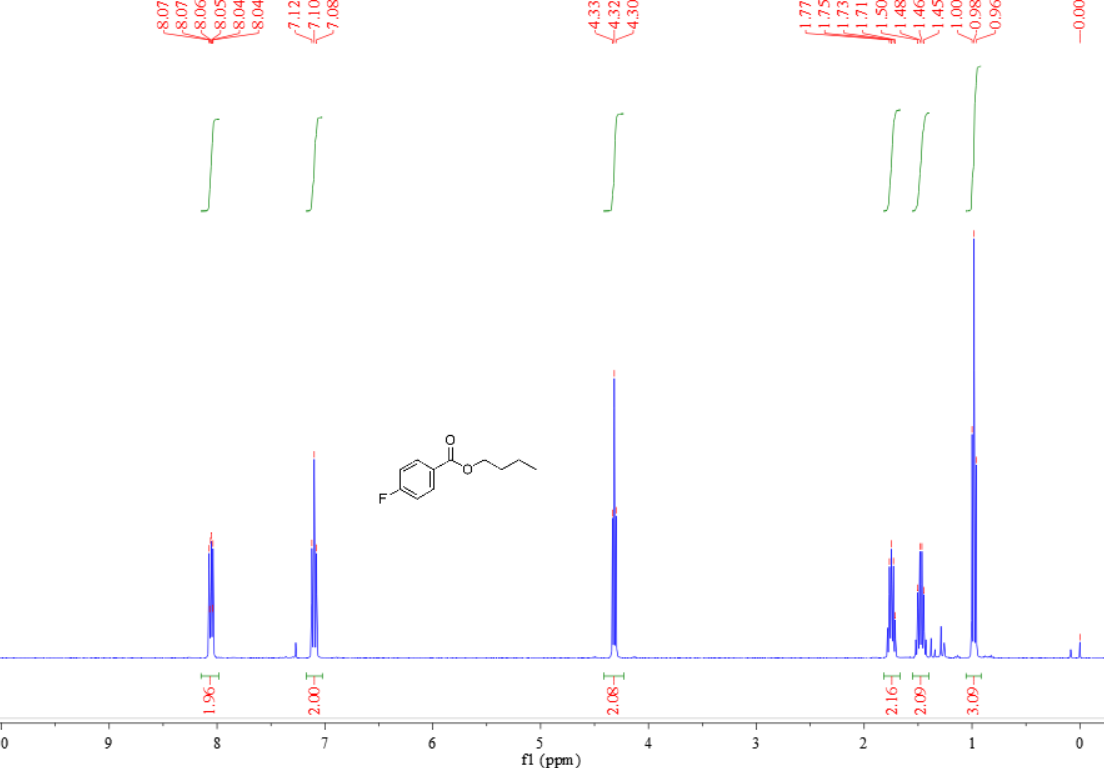


**Compound** **2jc** ^13^C{^1^H}NMR (100 MHz, CDCl_3_)


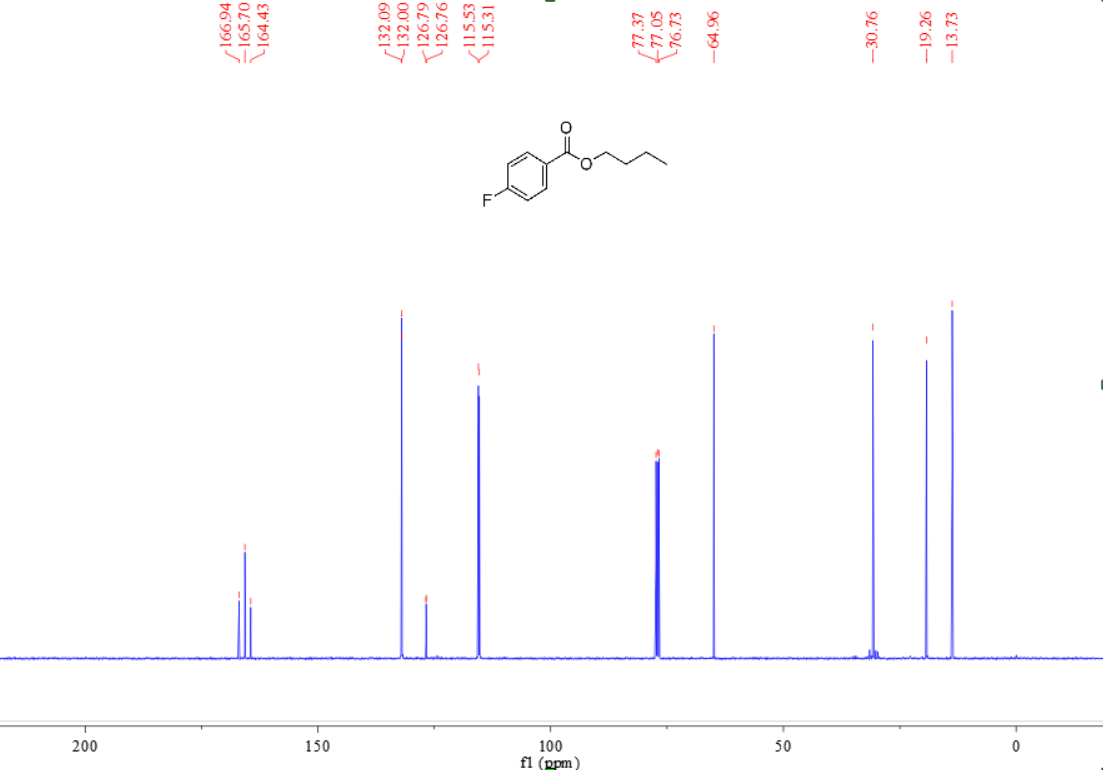


**Compound** **2k** ^1^H NMR (400 MHz, CDCl_3_)


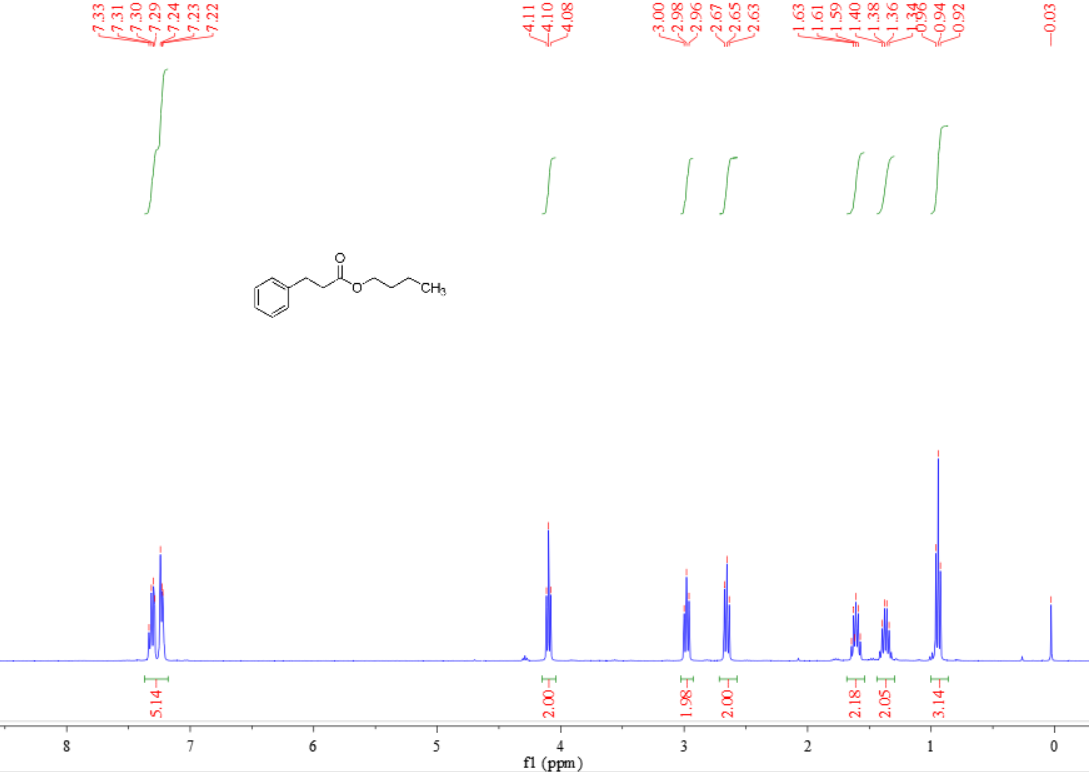


**Compound** **2k** ^13^C{^1^H}NMR (100 MHz, CDCl_3_)


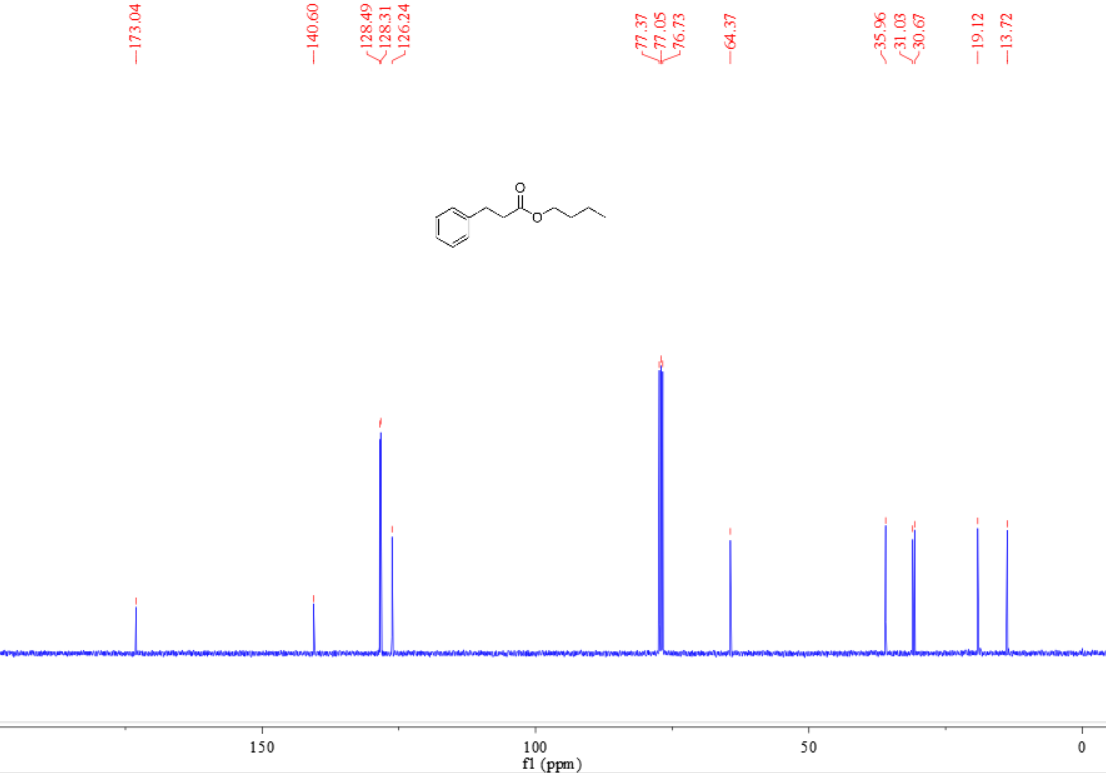


**Compound** **2l** ^1^H NMR (400 MHz, CDCl_3_)

**Compound** **2l** ^13^C{^1^H}NMR (100 MHz, CDCl_3_)

**Compound** **2m** ^1^H NMR (400 MHz, CDCl_3_)

**Compound** **2m** ^13^C{^1^H}NMR (100 MHz, CDCl_3_)

**Compound** **2mb** ^1^H NMR (400 MHz, CDCl_3_)

**Compound** **2mb** ^13^C{^1^H}NMR (100 MHz, CDCl_3_)

**Compound** **2n** ^1^H NMR (400 MHz, CDCl_3_)

**Compound** **2n** ^13^C{^1^H}NMR (100 MHz, CDCl_3_)

**Compound** **2nb** ^1^H NMR (400 MHz, CDCl_3_)

**Compound** **2nb** ^13^C{^1^H} NMR (100 MHz, CDCl_3_)

**Compound** **2nn** ^1^H NMR (400 MHz, CDCl_3_)

**Compound** **2nn** ^13^C{^1^H}NMR (100 MHz, CDCl_3_)

**Compound** **2nnb**^1^H NMR (400 MHz, CDCl_3_)

**Compound** **2nnb** ^13^C{^1^H}NMR (100 MHz, CDCl_3_)

**Compound** **2of** ^1^H NMR (400 MHz, CDCl_3_)

**Compound** **2of** ^13^C{^1^H}NMR (100 MHz, CDCl_3_)

**Compound** **2og** ^1^H NMR (400 MHz, CDCl_3_)

**Compound** **2og** ^13^C{^1^H}NMR (100 MHz, CDCl_3_)

**Compound** **2pp** ^1^H NMR (400 MHz, CDCl_3_)

**Compound** **2pp** ^13^C {^1^H}NMR (100 MHz, CDCl_3_)

**Compound** **2qq** ^1^H NMR (400 MHz, CDCl_3_)

**Compound** **2qq** ^13^C {^1^H}NMR (100 MHz, CDCl_3_)

**Compound** **2rr** ^1^H NMR (400 MHz, CDCl_3_)

**Compound** **2rr** ^13^C {^1^H}NMR (100 MHz, CDCl_3_)

**Compound** **INT 1z** ^1^H NMR (500 MHz, CDCl_3_)

**Compound** **INT 1z** ^13^C{^1^H}NMR (125 MHz, CDCl_3_)

**Compound** **INT 3z** ^1^H NMR (400 MHz, CDCl_3_)

**Compound** **INT 3z** ^13^C{^1^H}NMR (100 MHz, CDCl_3_)

**Compound** **2z** ^1^H NMR (400 MHz, CDCl_3_)

**Compound** **2z** ^13^C{^1^H}NMR (100 MHz, CDCl_3_)

**Compound** **3o** ^1^H NMR (400 MHz, CDCl_3_)

**Compound** **3o** ^13^C{^1^H}NMR (100 MHz, CDCl_3_)

**Compound** **3p**^1^H NMR (400 MHz, CDCl_3_)

**Compound** **3p** ^13^C{^1^H}NMR (100 MHz, CDCl_3_)

**Compound** **3q** ^1^H NMR (400 MHz, CDCl_3_)

**Compound** **3q** ^13^C{^1^H}NMR (100 MHz, CDCl_3_)

**Compound** **3r** ^1^H NMR (400 MHz, CD_3_OD)

**Compound** **3r** ^13^C{^1^H}NMR (100 MHz, CD_3_OD)

**Compound** **3s** ^1^H NMR (400 MHz, CDCl_3_)

**Compound** **3s** ^13^C{^1^H}NMR (100 MHz, CDCl_3_)

**Compound** **3t** ^1^H NMR (400 MHz, CDCl_3_)

**Compound** **3t** ^13^C{^1^H}NMR (100 MHz, CDCl_3_)

**Compound** **3u** ^1^H NMR (400 MHz, CDCl_3_)

**Compound** **3u** ^13^C{^1^H}NMR (100 MHz, CDCl_3_)

**Compound** **3v** ^1^H NMR (400 MHz, CDCl_3_)

**Compound** **3v** ^13^C{^1^H}NMR (100 MHz, CDCl_3_)

**Compound** **3w** ^1^H NMR (400 MHz, CD_3_OD)

**Compound** **3w** ^13^C{^1^H}NMR (100 MHz, CD_3_OD)

**Compound** **3x** ^1^H NMR (400 MHz, CD_3_OD)

**Compound** **3x** ^13^C{^1^H}NMR (100 MHz, CD_3_OD)

**Compound** **3y** ^1^H NMR (400 MHz, CD_3_OD)

**Compound** **3y** ^13^C{^1^H}NMR (100 MHz, CD_3_OD)

**Compound** **3wD** ^1^H NMR (400 MHz, CD_3_OD)

**Compound** **3wD** ^13^C{^1^H}NMR (100 MHz, CD_3_OD)

**Compound** **3xD** ^1^H NMR (400 MHz, CD_3_OD)

**Compound** **3xD** ^13^C{^1^H}NMR (100 MHz, CD_3_OD)

**Compound** **3yD** ^1^H NMR (400 MHz, CD_3_OD)

**Compound** **3yD** ^13^C{^1^H}NMR (100 MHz, CD_3_OD)
