# Supplementary material for: Direct esterification of amides by the dimethylsulfate-mediated activation of amide C–N bonds
Source: Commun Chem. 2024 Apr 27;7:93. doi: 10.1038/s42004-024-01180-9 (PMC11055851; doi:10.1038/s42004-024-01180-9)

**Supplementary Data 2**

**Direct esterification of amides by the dimethylsulfate-mediated activation of amide C–N bonds**

## DFT Calculations

**Energy values for the reported species and imaginary frequencies for the transition states**

**Table S1.** Zero-point energy (Hartree/Particle), energy corrections, enthalpy corrections and free energy corrections of the structures calculated at M06-2X PCM/6-31G(d), single point energies (Hartree) at the M06-2X SMD/6-311++G(d,p) level of theory and imaginary frequencies of the transition states.

| structures | ZPE | corr. to E | corr. to H | Corr. to G | SP | IF |
| --- | --- | --- | --- | --- | --- | --- |
| **1a+Me_2_SO_4_** | 0.256704 | 0.275653 | 0.276597 | 0.205513 | -1219.005 | — |
| **TS-1** | 0.255942 | 0.274053 | 0.274997 | 0.205782 | -1218.965 | -679.08 |
| **INT-1** | 0.258059 | 0.275665 | 0.276609 | 0.210001 | -1219.005 | — |
| **1-BuOH** | 0.138816 | 0.145692 | 0.146636 | 0.108618 | -233.631 | — |
| **TS-2** | 0.397906 | 0.423595 | 0.424539 | 0.338621 | -1452.613 | -638.99 |
| **BuOCH_3_** | 0.167723 | 0.175846 | 0.17679 | 0.1353 | -272.9172 | — |
| **INT-2** | 0.226826 | 0.243361 | 0.244305 | 0.180126 | -1179.735 | — |
| **INT-3** | 0.228479 | 0.245423 | 0.246367 | 0.180332 | -1179.734 | — |
| **TS-3** | 0.366889 | 0.38984 | 0.390785 | 0.313702 | -1413.347 | -672.66 |
| **INT-4** | 0.369771 | 0.39347 | 0.394414 | 0.313395 | -1413.348 | — |
| **INT-5** | 0.373352 | 0.396475 | 0.397419 | 0.319557 | -1413.384 | — |
| **TS-4** | 0.365917 | 0.390026 | 0.39097 | 0.310195 | -1413.358 | -108.51 |
| **[MeNH_3_] MeSO_4_** | 0.138614 | 0.149355 | 0.150299 | 0.100869 | -835.397 | — |
| **3ac** | 0.231724 | 0.244558 | 0.245502 | 0.190286 | -577.991 | — |

# Compound INT-1z X-single-crystal diffraction

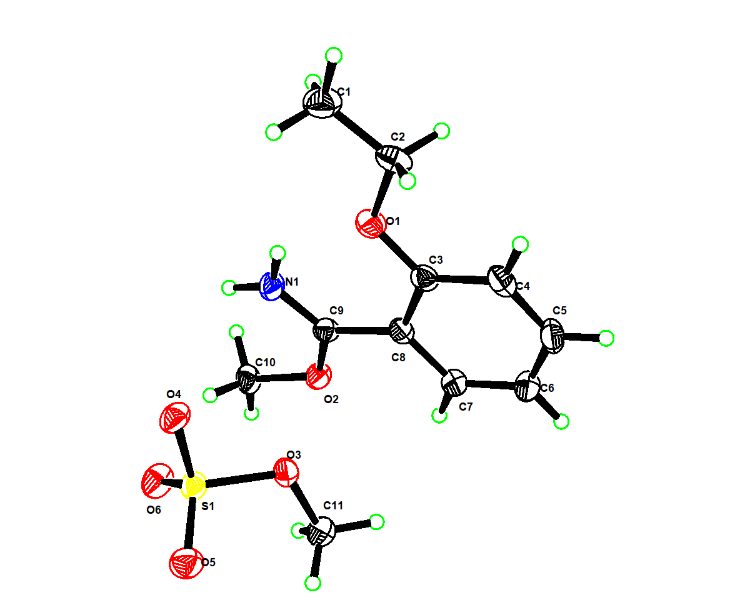


CheckCIF/PLATON report

Datablock: **compound INT-1z**


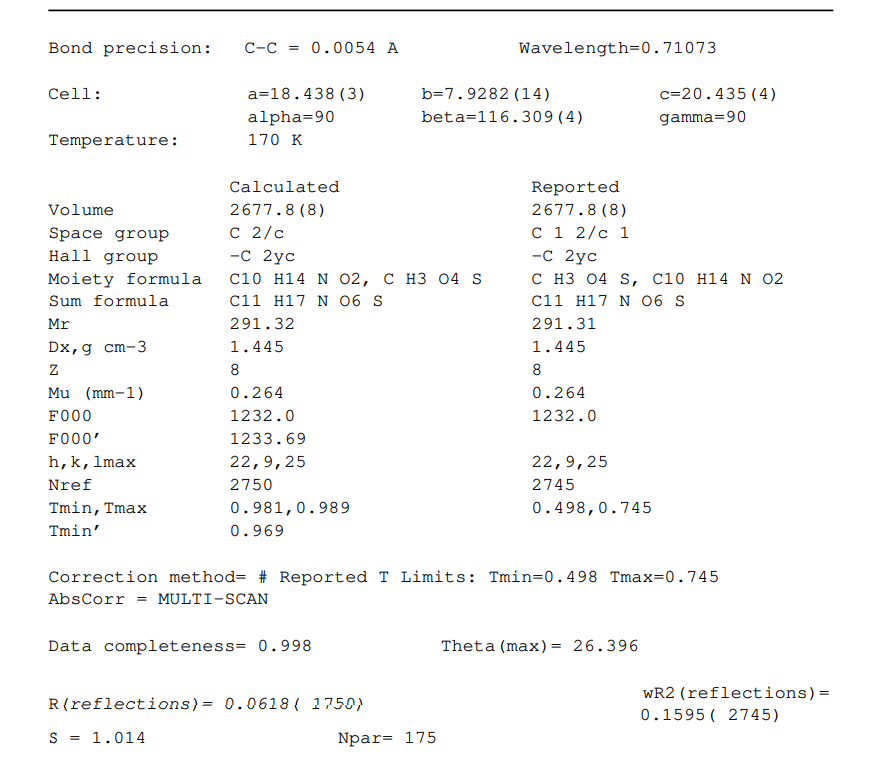


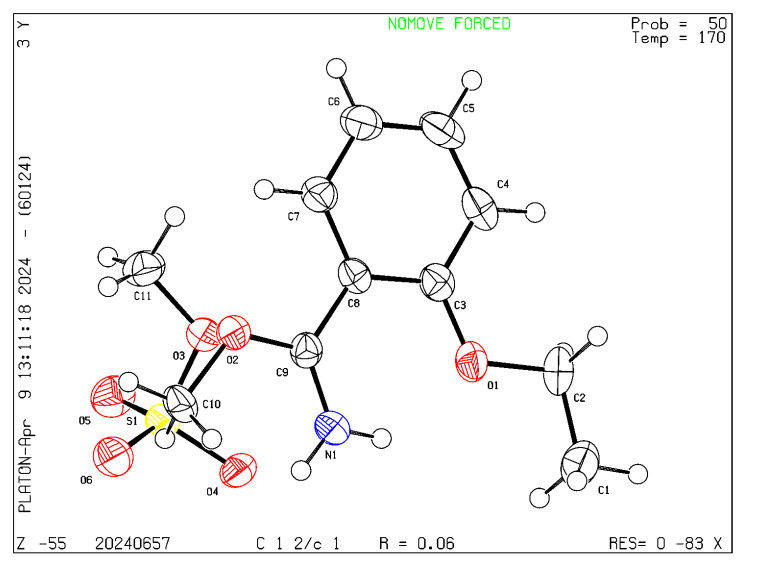


**Table S2.** Crystal data and structure refinement for **compound INT-1z**


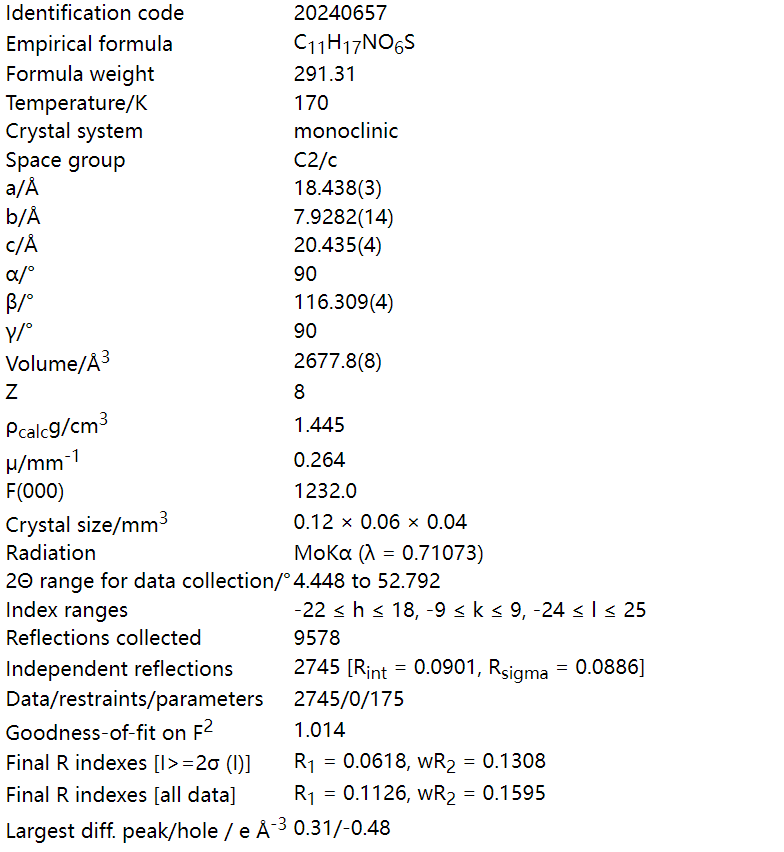


**Table S3.** Fractional Atomic Coordinates (×10^4^) and Equivalent Isotropic Displacement Parameters (Å^2^×10^3^) for **compound INT-1z** U_eq_ is defined as 1/3 of the trace of the orthogonalised U_IJ_ tensor.


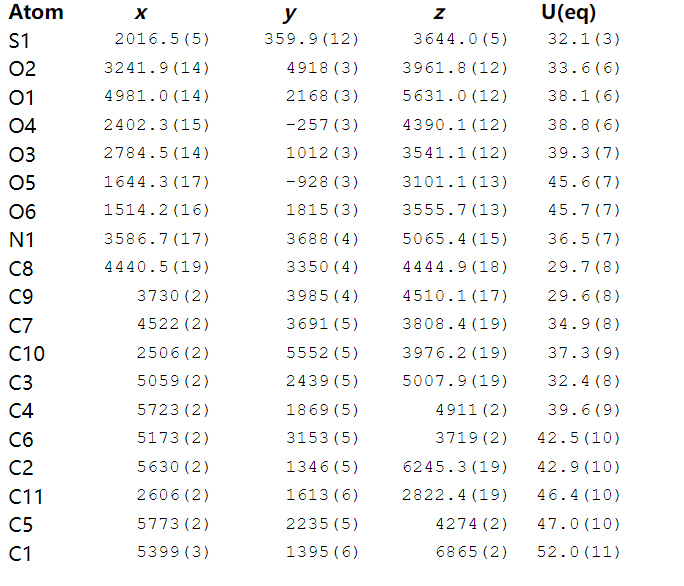


**Table S4**. Anisotropic Displacement Parameters (Å2×103) for **compound INT-1z**. The Anisotropic displacement factor exponent takes the form: -2π2[h2a*2U11+2hka*b*U12+…]


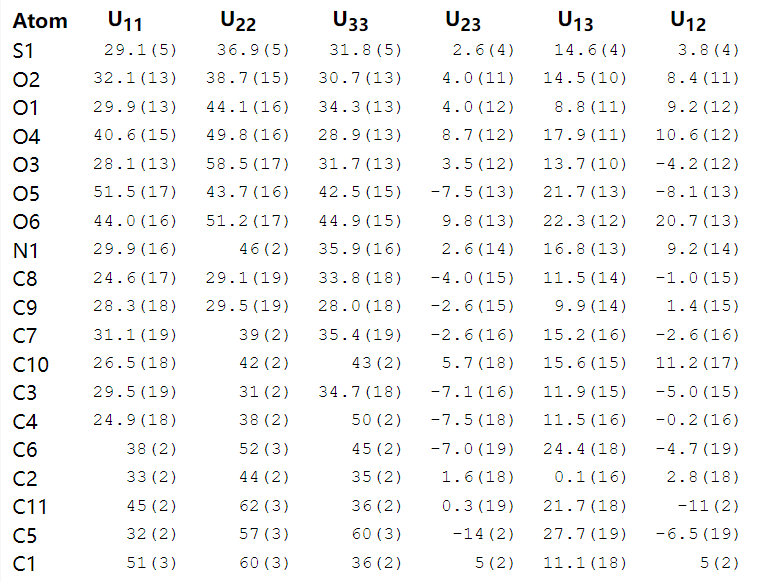


**Table S5.** Bond Lengths for **compound INT-1z**


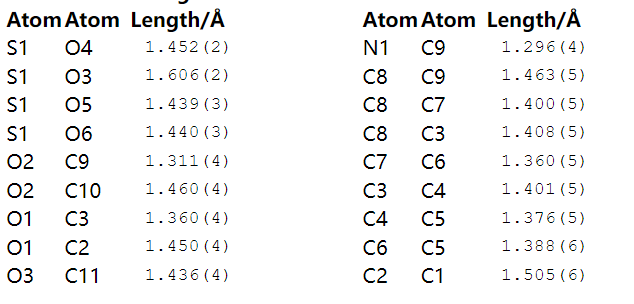


**Table S6.** Bond Angles for **compound INT-1z**


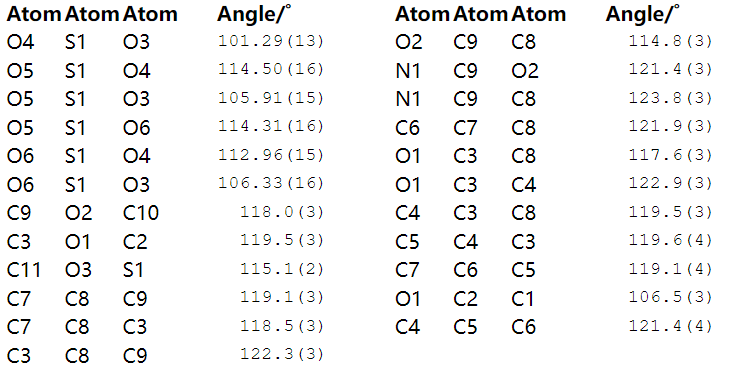


**Table S7**. Torsion Angles for **compound INT-1z**


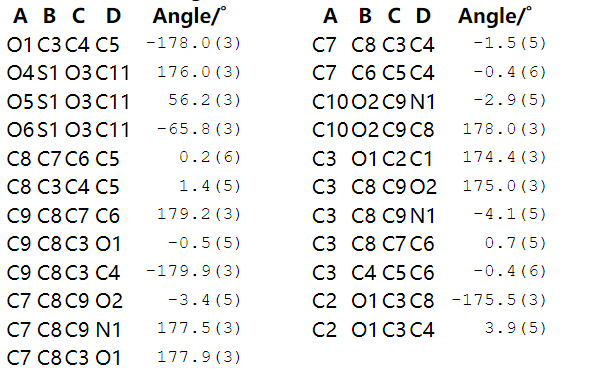


**Table S8.** Hydrogen Atom Coordinates (Å×104) and Isotropic Displacement Parameters (Å2×103) for **compound INT-1z**


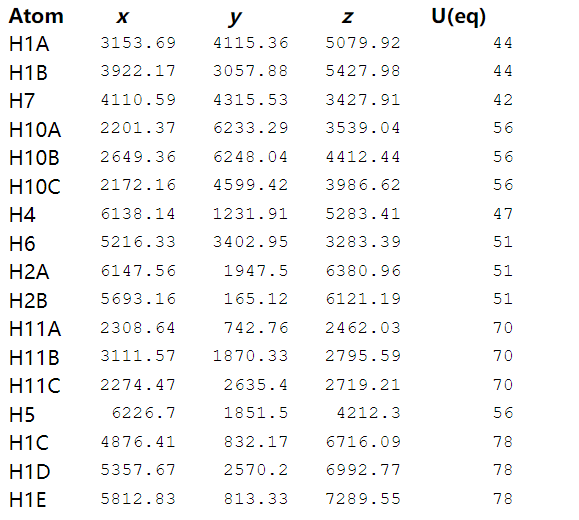

Supplement: Supplementary file 4 — Supplementary Data 2 file [file 42004_2024_1180_MOESM4_ESM.docx]
