# Supplementary material for: Direct esterification of amides by the dimethylsulfate-mediated activation of amide C–N bonds
Source: Commun Chem. 2024 Apr 27;7:93. doi: 10.1038/s42004-024-01180-9 (PMC11055851; doi:10.1038/s42004-024-01180-9)
Supplement: Supplementary file 5 — Supplementary Data 3 file [file 42004_2024_1180_MOESM5_ESM.docx]

**Supplementary Data 3**

**Direct esterification of amides by the dimethylsulfate-mediated activation of amide C–N bonds**

# Chromatograms

Synthesis of **2m** from esterification of **1m** undergo the dimethylsulphate-mediated in methanol

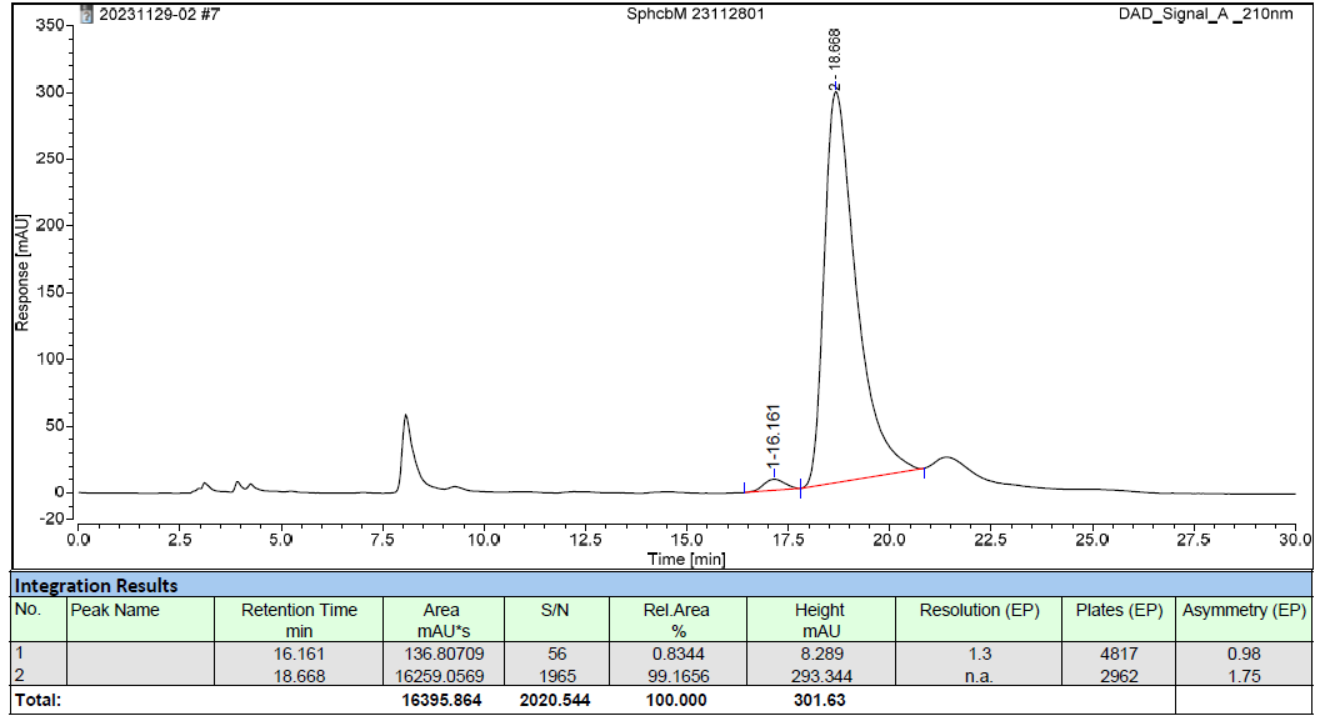


**Figure S1:** HPLC chromatogram of **2m** from esterification of **1m** undergo the dimethylsulphate-mediated in methanol (column: CHIRALCEL IG 250×4.6mm, 5μm, 30 °C, Hexane: Ethanol = 75:25, 1mL/min, λ =210 nm).

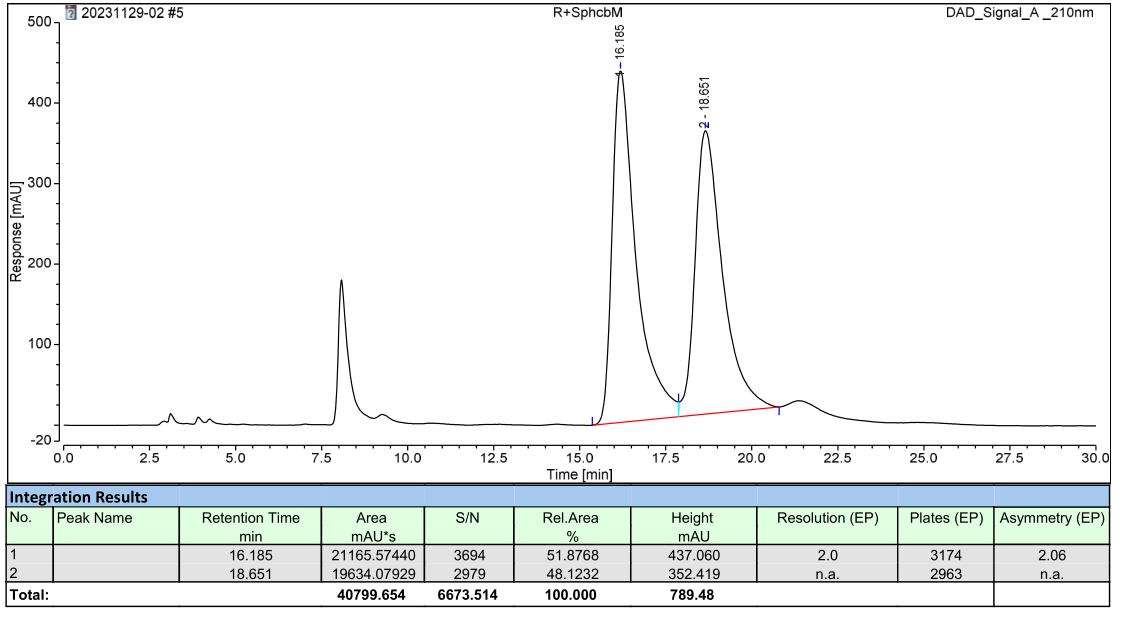


**Figure S2**: HPLC chromatogram of the mixture of **2m** and enantiomeric isomer of **2m** (column: CHIRALCEL IG 250×4.6mm, 5μm, 30 °C, Hexane: Ethanol = 75:25, 1mL/min, λ =210 nm).

Synthesis of **2mb** from esterification of **1m** undergo the dimethylsulphate-mediated in 1-butanol


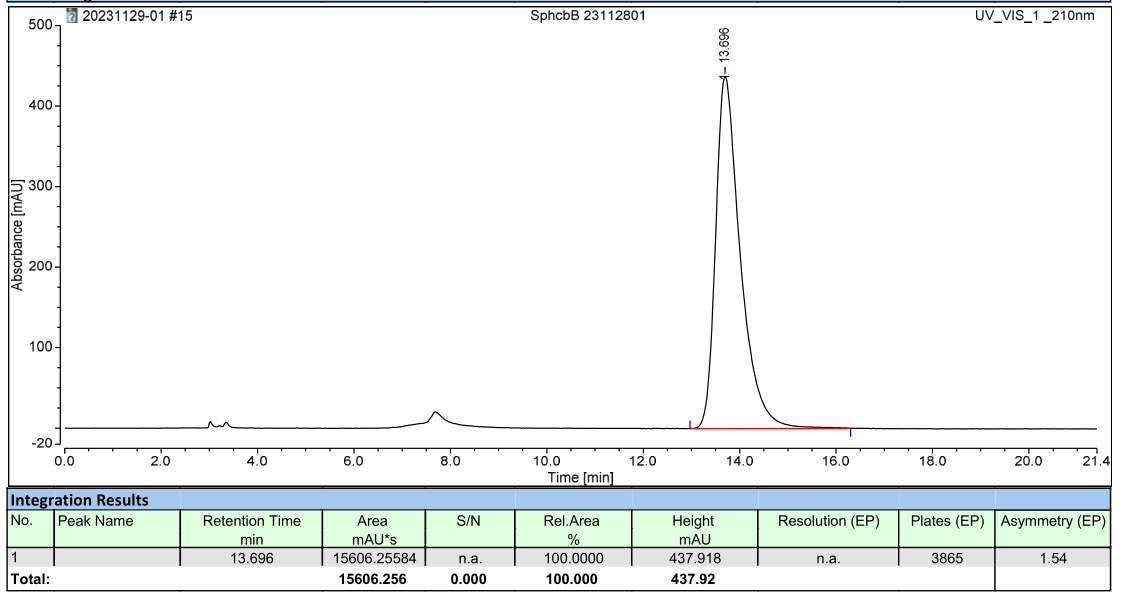


**Figure S3**: HPLC chromatogram of **2mb** from esterification of **1m** undergo the dimethylsulphate-mediated in 1-buthanol (column: CHIRALPAK AD-H 250×4.6mm, 5μm, 30 °C, Hexane: Ethanol = 85:15, 1mL/min, λ =210 nm).


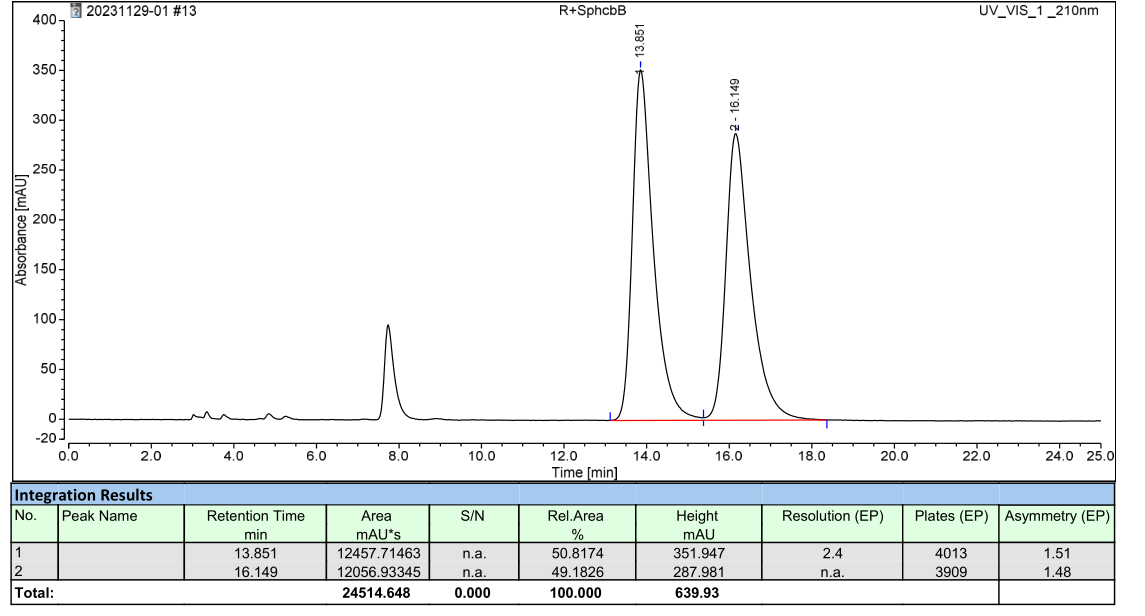


**Figure S4:** HPLC chromatogram of the mixture of **2mb** and enantiomeric isomer of **2mb** (column: CHIRALPAK AD-H 250×4.6mm, 5μm, 30 °C, Hexane: Ethanol = 85:15, 1mL/min, λ =210 nm).

Synthesis of **2n** from esterification of **1n** undergo the dimethylsulphate-mediated in methanol

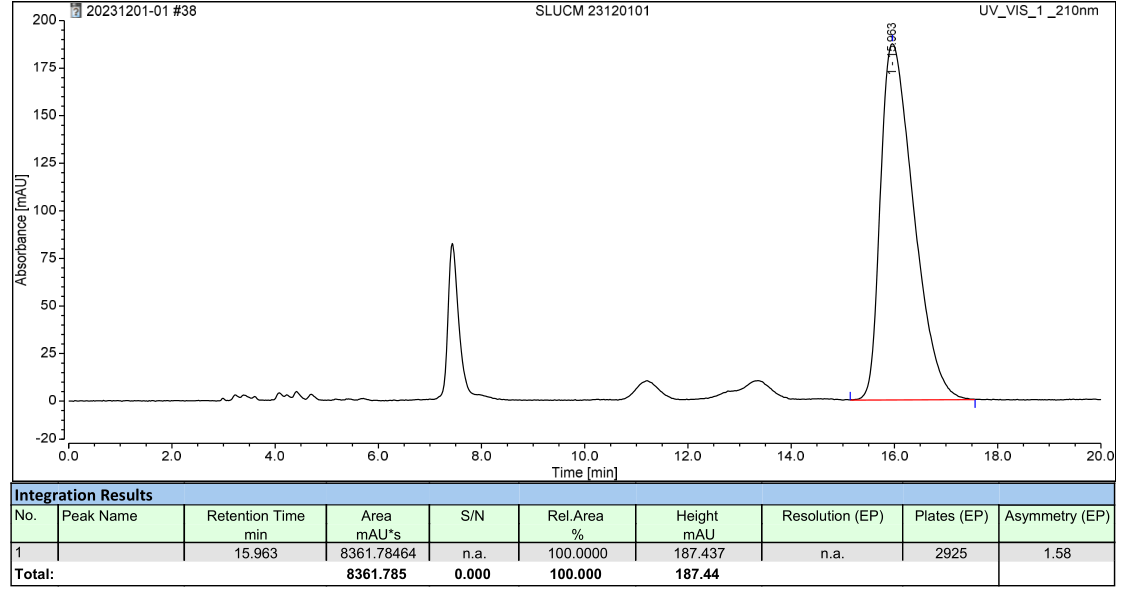


**Figure S5**: HPLC chromatogram of **2n** from esterification of **1n** undergo the dimethylsulphate-mediated in methanol (column: CHIRALCEL IG 250×4.6mm, 5μm, 30 °C, Hexane: Ethanol : Isopropanol= 70:15:15, 1mL/min, λ =210 nm).


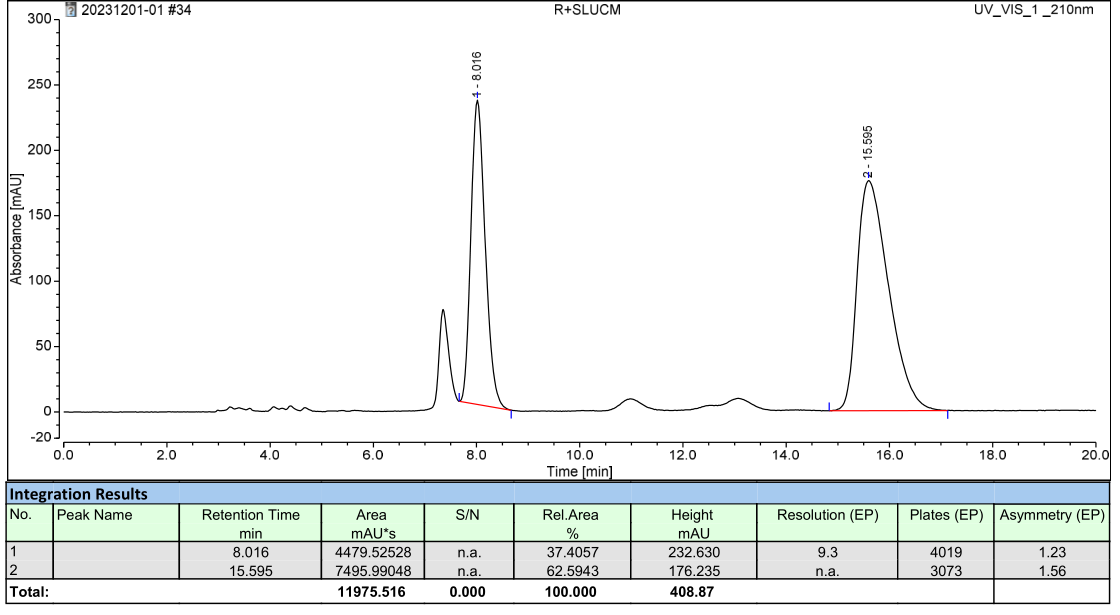


**Figure S6**: HPLC chromatogram of the mixture of **2n** and enantiomeric isomer of **2n** (column: CHIRALCEL IG 250×4.6mm, 5μm, 30 °C, Hexane: Ethanol : Isopropanol= 70:15:15, 1mL/min, λ =210 nm).

Synthesis of **2nb** from esterification of **1n** undergo the dimethylsulphate-mediated in 1-buthanol

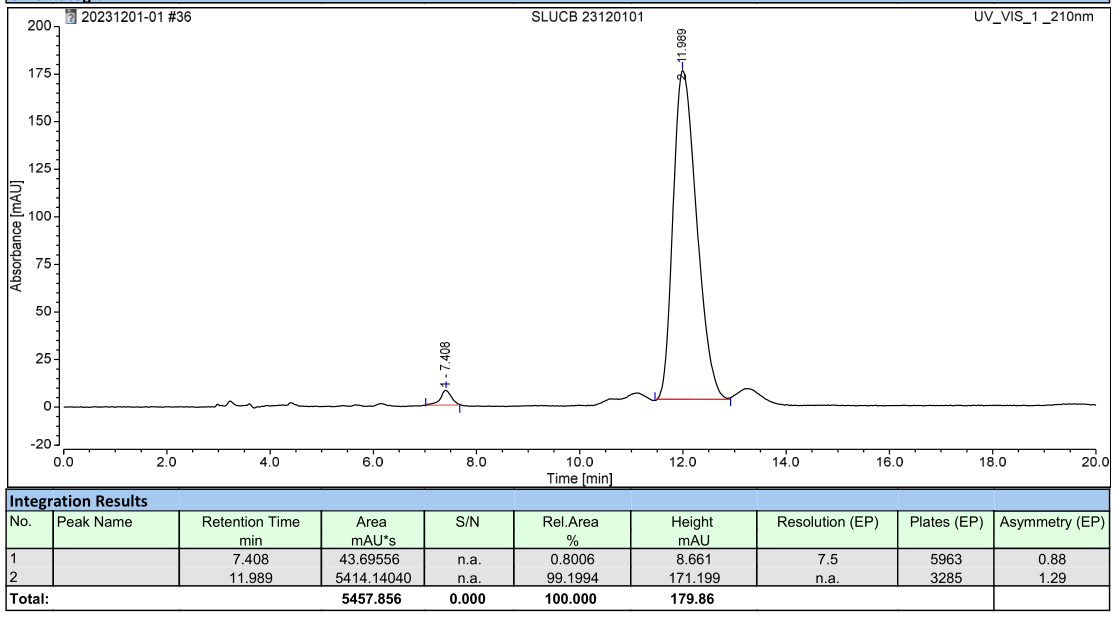


**Figure S7**: HPLC chromatogram of **2nb** from esterification of **1n** undergo the dimethylsulphate-mediated in 1-buthanol (column: CHIRALCEL IG 250×4.6mm, 5μm, 30 °C, Hexane: Ethanol : Isopropanol= 70:15:15, 1mL/min, λ =210 nm).


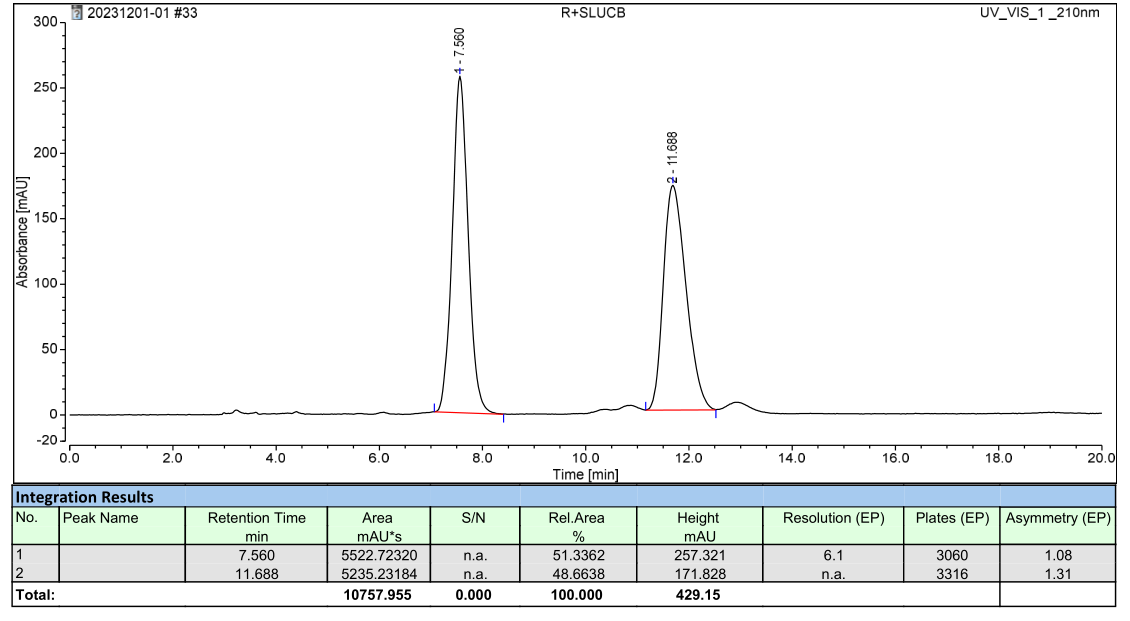


**Figure S8**: HPLC chromatogram of the mixture of **2nb** and enantiomeric isomer of **2nb** (column: CHIRALCEL IG 250×4.6mm, 5μm, 30 °C, Hexane: Ethanol : Isopropanol= 70:15:15, 1mL/min, λ =210 nm).

Synthesis of **2nn** from esterification of **1nn** undergo the dimethylsulphate-mediated in methanol

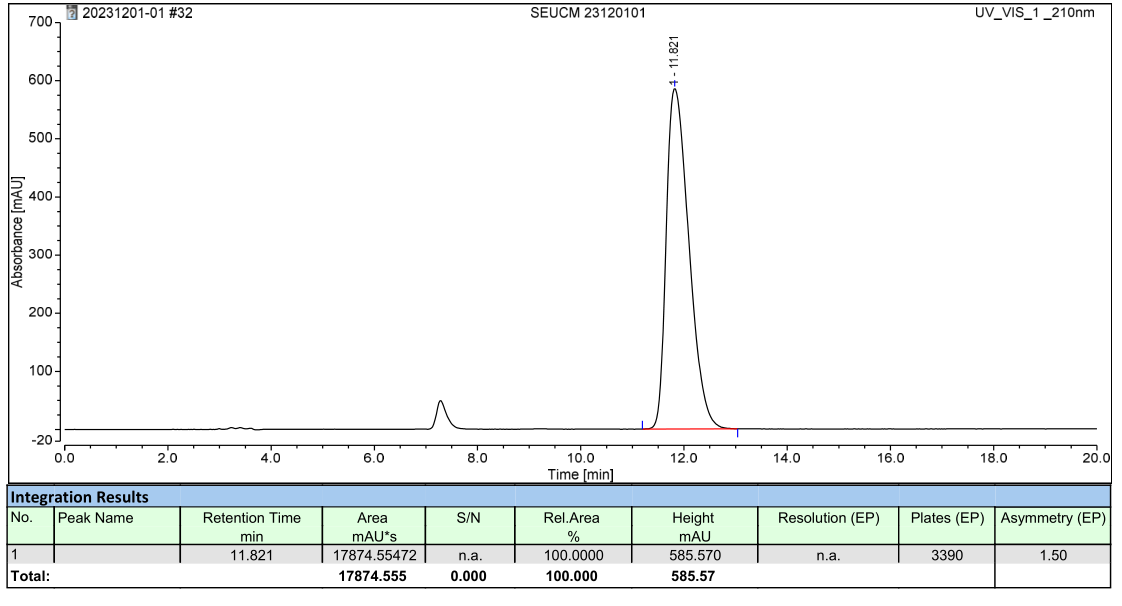


**Figure S9**: HPLC chromatogram of **2nn** from esterification of **1nn** undergo the dimethylsulphate-mediated in methanol (column: CHIRALCEL IG 250×4.6mm, 5μm, 30 °C, Hexane: Ethanol : Isopropanol= 70:15:15, 1mL/min, λ =210 nm).


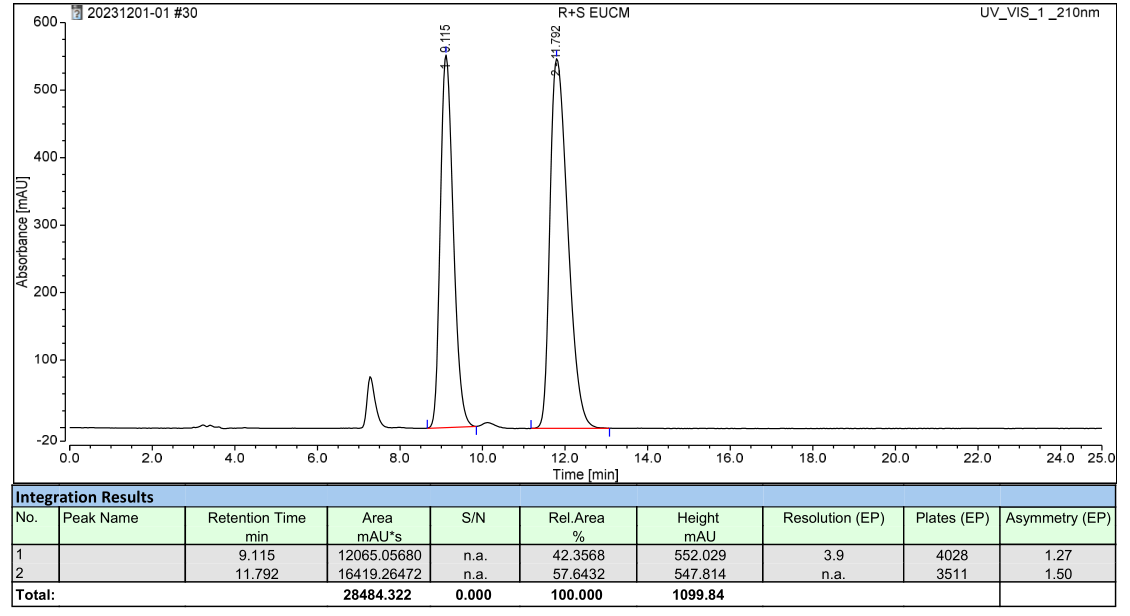


**Figure S10**: HPLC chromatogram of the mixture **of 2nn** and enantiomeric isomer of **2nn** (column: CHIRALCEL IG 250×4.6mm, 5μm, 30 °C, Hexane: Ethanol : Isopropanol= 70:15:15, 1mL/min, λ =210 nm).

Synthesis of **2nnb** from esterification of **1nn** undergo the dimethylsulphate-mediated in 1-buthanol

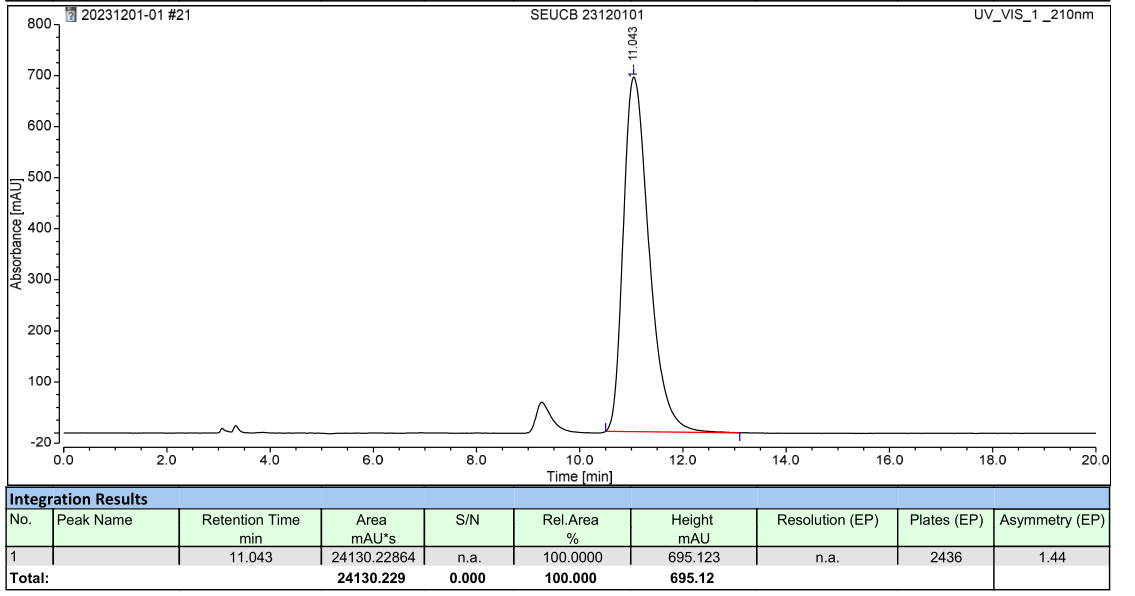


**Figure S11**: HPLC chromatogram of **2nnb** from esterification of **1nn** undergo the dimethylsulphate-mediated in 1-buthanol (column: CHIRALPAK AD-H 250×4.6mm, 5μm, 30 °C, Hexane: Ethanol : Isopropanol = 92:4:4, 1mL/min, λ =210 nm).


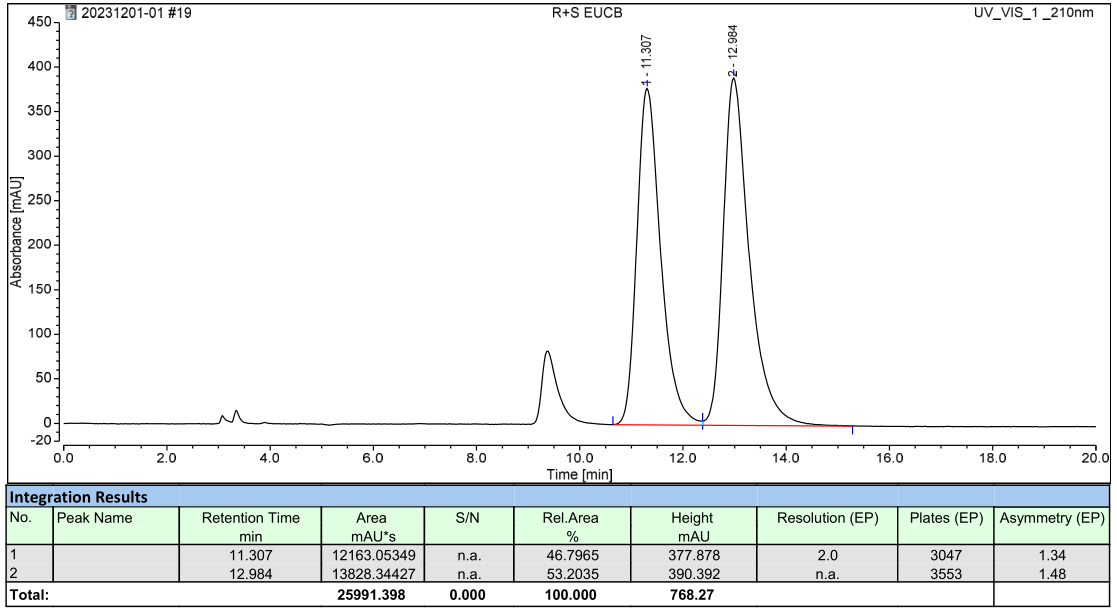


**Figure S12**: HPLC chromatogram of the mixture of **2nnb** and enantiomeric isomer of **2nnb** (column: CHIRALPAK AD-H 250×4.6mm, 5μm, 30 °C, Hexane: Ethanol : Isopropanol = 92:4:4, 1mL/min, λ =210 nm).

Synthesis of **3w** from cleavage of acyl protective group of **1w** undergo the dimethylsulphate-mediated in 1-buthanol

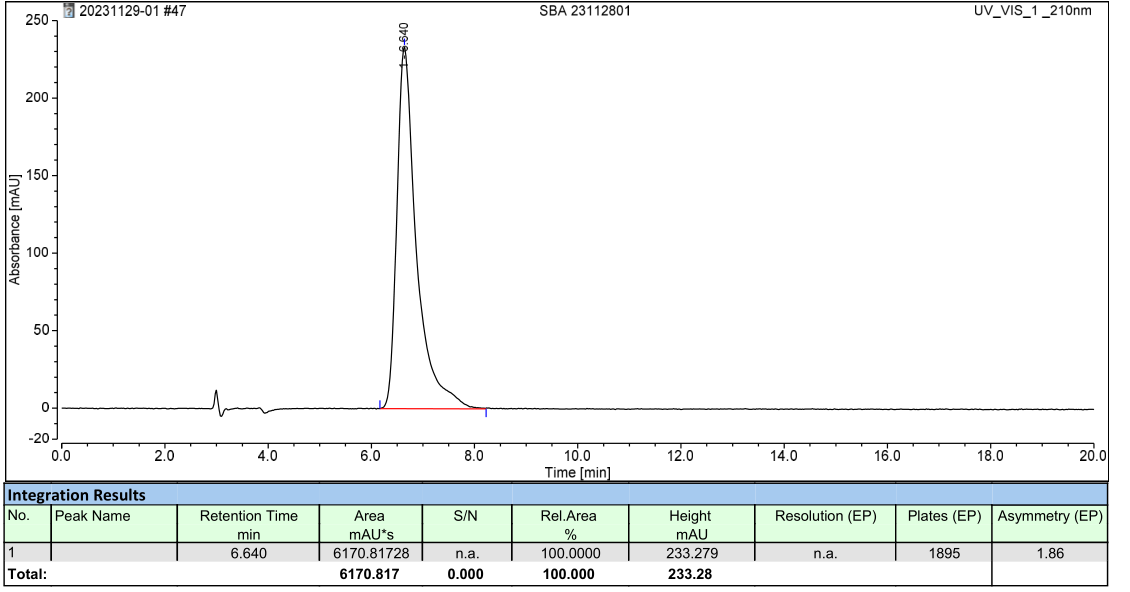


**Figure S13:** HPLC chromatogram of **3w** from cleavage of acyl protective group of **1wb** undergo the dimethylsulphate-mediated in 1-buthanol (column: CHIRALCEL IG 250×4.6mm, 5μm, 30 °C, Hexane: Ethanol = 80:20, 1mL/min, λ =210 nm).


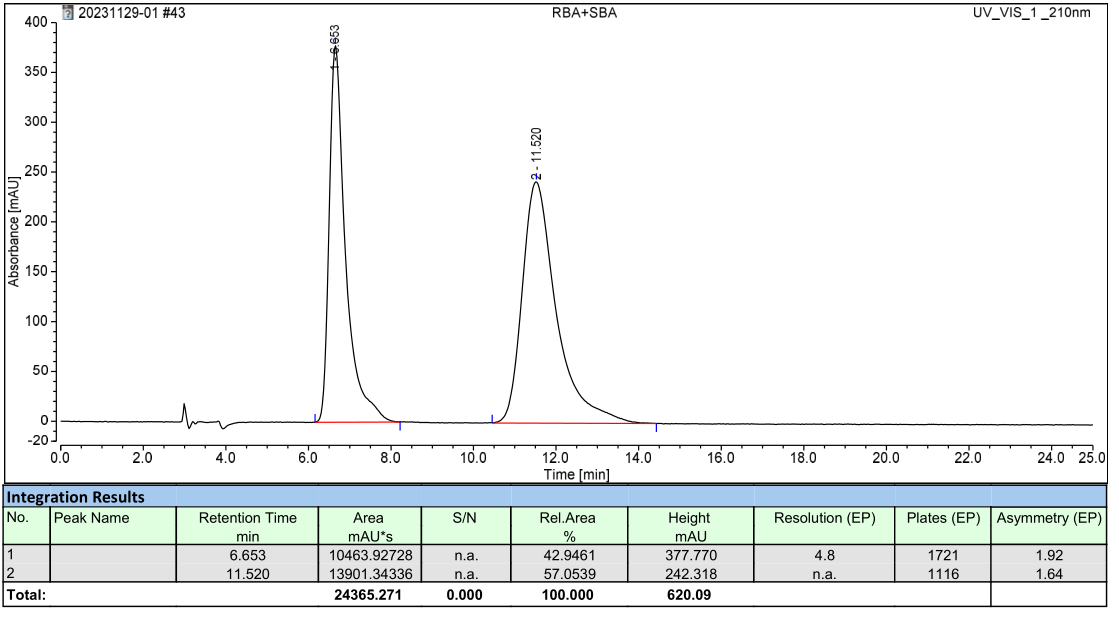


**Figure S14**: HPLC chromatogram of the mixture of **3w** and enantiomeric isomer of **3w** (**3wD**) (column: CHIRALCEL IG 250×4.6mm, 5μm, 30 °C, Hexane: Ethanol = 80:20, 1mL/min, λ =210 nm).

Synthesis of **3x** from cleavage of acyl protective group of **1xb** undergo the dimethylsulphate-mediated in 1-buthanol

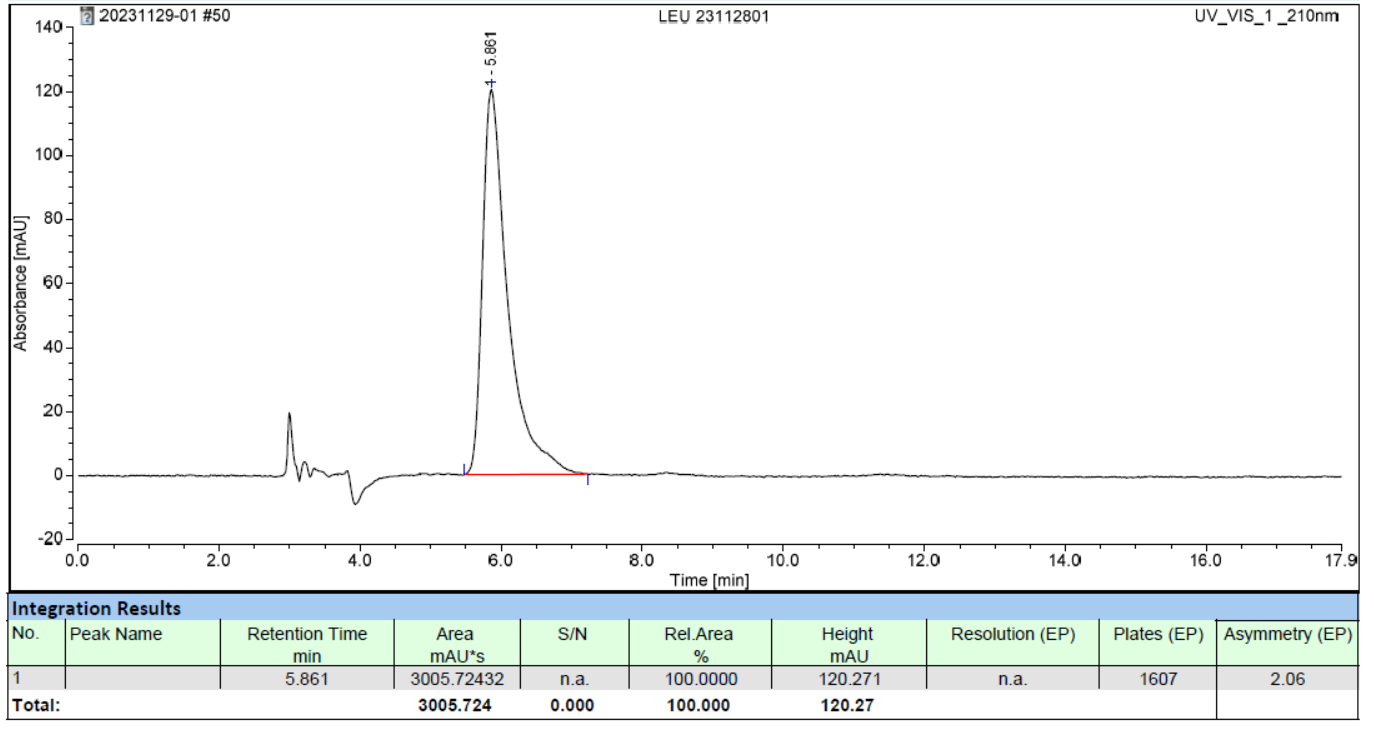


**Figure S15**: HPLC chromatogram of **3x** from cleavage of acyl protective group of **1xb** undergo the dimethylsulphate-mediated in 1-buthanol (column: CHIRALCEL IG 250×4.6mm, 5μm, 30 °C, Hexane: Ethanol = 80:20, 1mL/min, λ =210 nm).


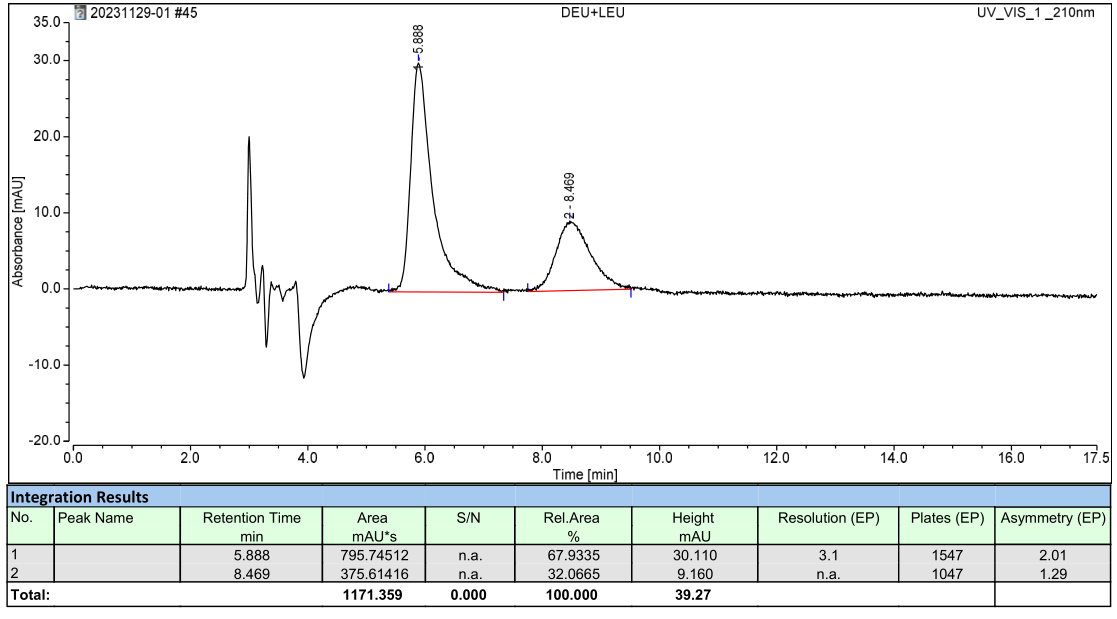


**Figure S14**: HPLC chromatogram of the mixture of **3x** and enantiomeric isomer of **3x** (**3xD**) (column: CHIRALCEL IG 250×4.6mm, 5μm, 30 °C, Hexane: Ethanol = 80:20, 1mL/min, λ =210 nm).

Synthesis of **3y** from cleavage of acyl protective group of **1yb** undergo the dimethylsulphate-mediated in 1-buthanol

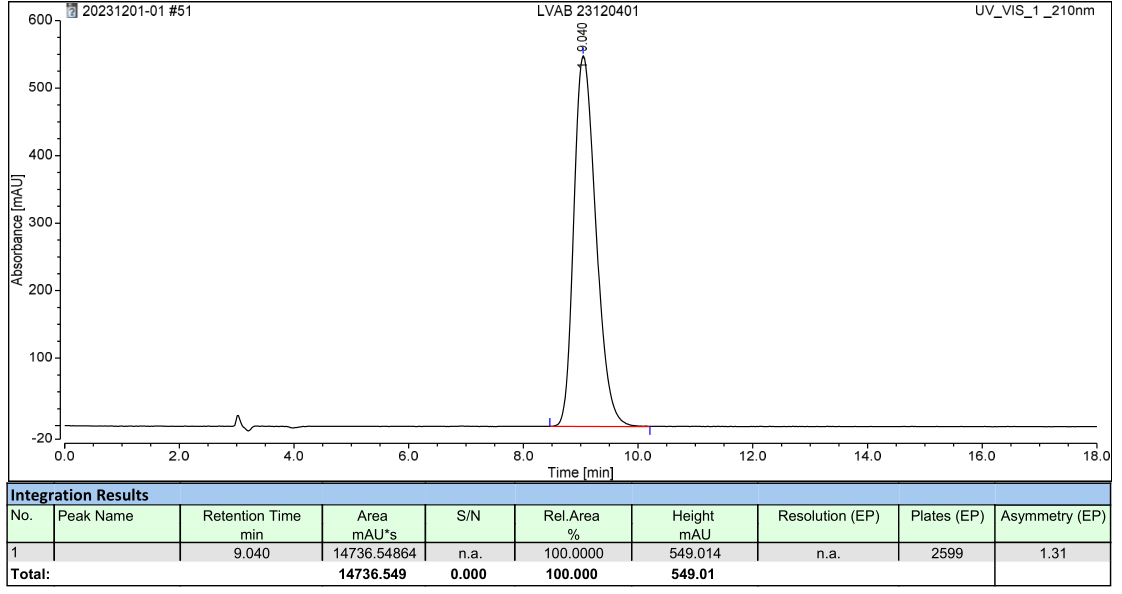


**Figure S17**: HPLC chromatogram of the mixture of **3y** and enantiomeric isomer of **1yb** (column: CHIRALCEL IG 250×4.6mm, 5μm, 30 °C, Hexane: Ethanol : Isopropanol= 70:15:15, 1mL/min, λ =210 nm).


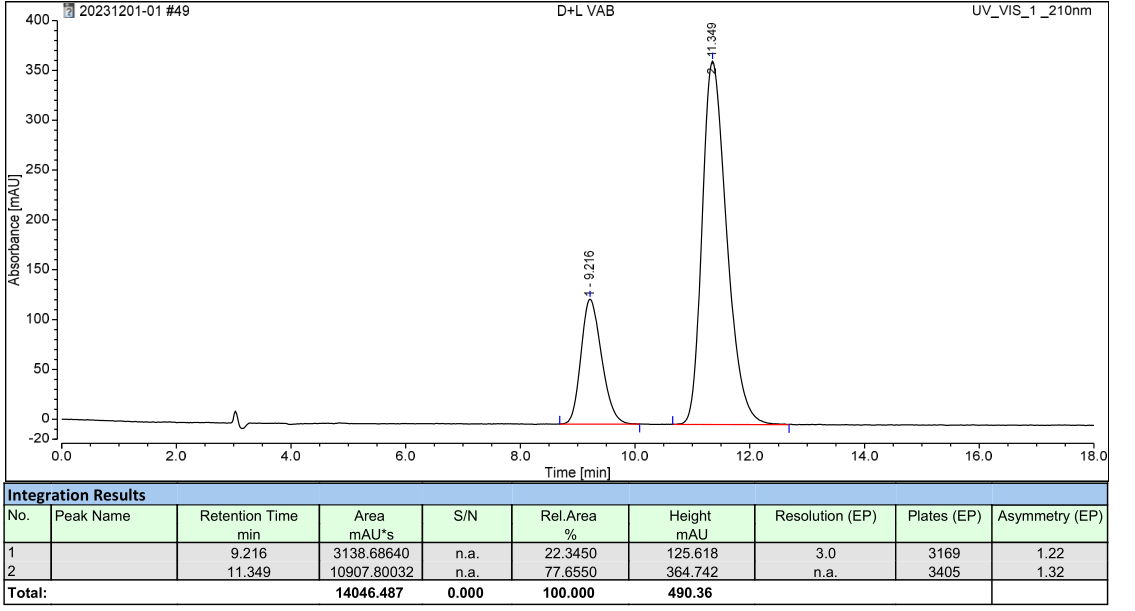


**Figure S18**: HPLC chromatogram of the mixture of **3y** and enantiomeric isomer of **3y** (**3yD**) (column: CHIRALCEL IG 250×4.6mm, 5μm, 30 °C, Hexane: Ethanol : Isopropanol= 70:15:15, 1mL/min, λ =210 nm).
